# Regioselective decarboxylative addition of malonic acid and its mono(thio)esters to 4-trifluoromethylpyrimidin-2(1*H*)-ones

**DOI:** 10.3762/bjoc.13.259

**Published:** 2017-12-07

**Authors:** Sergii V Melnykov, Andrii S Pataman, Yurii V Dmytriv, Svitlana V Shishkina, Mykhailo V Vovk, Volodymyr A Sukach

**Affiliations:** 1 Institute of Organic Chemistry, National Academy of Sciences of Ukraine, 5 Murmanska str., Kyiv 02660, Ukraine; 2 Enamine LTD, 78 Chervonotkats‘ka str., Kyiv 02094, Ukraine; 3 National Technical University of Ukraine “Igor Sikorsky Kyiv Polytechnic Institute”, 37 Peremohy ave., Kyiv 03056, Ukraine; 4 STC ‘‘Institute for Single Crystals’’, National Academy of Sciences of Ukraine, 60 Nauky ave., Kharkiv 61001, Ukraine; 5 Department of Inorganic Chemisrtry, V.M. Karasin Kharkiv National University, 4 Svobody sq, Kharkiv 61122, Ukraine

**Keywords:** ketimines, malonic acid, Michael- and Mannich-type decarboxylative addition, pyrimidin-2(1*H*)-ones, regioselectivity, trifluoromethyl group

## Abstract

**Background:** Due to the high reactivity towards various C-nucleophiles, trifluoromethylketimines are known to be useful reagents for the synthesis of α-trifluoromethylated amine derivatives. However, decarboxylative reactions with malonic acid and its mono(thio)esters have been poorly investigated so far despite the potential to become a convenient route to β-trifluoromethyl-β-amino acid derivatives and to their partially saturated heterocyclic analogues.

**Results:** In this paper we show that 4-trifluoromethylpyrimidin-2(1*H*)-ones, unique heterocyclic ketimines, react with malonic acid under organic base catalysis to regioselectively provide either Michael- or Mannich-type decarboxylative addition products depending on solvent polarity. Malonic mono(thio)esters give exclusively Michael-type products. The two regioisomeric products can be converted into saturated (2-oxohexahydropyrimidin-4-yl)acetic acid derivatives by mild hydrogenation of the endocyclic C=C double bond in the presence of Pd/C as catalyst. The *cis*-stereoisomers selectively formed upon reduction of the Michael-type products were structurally determined by X-ray diffraction. As a result of this study, a number of novel acetic acid derivatives containing trifluoromethylated, partially or fully saturated 2-oxopyrimidine cores were prepared and characterized as promising building blocks.

**Conclusions:** Regio- and stereoselective protocols have been developed for the synthesis of novel isomeric 4(6)-trifluoromethylated 1,2,3,4-tetrahydro- and perhydro-(2-oxopyrimidin-4-yl)acetic acid derivatives.

## Introduction

Organofluorine compounds now play an essential role in the development of new materials for solar cells [1–3], radiotracers for PET imaging [4], agrochemicals [5–6], sensitive chemical probes for ^19^F nuclear magnetic resonance investigation of biological experiments [7–8], and are most widely used in the modern drug discovery and development area [9–10]. As a result of intensive research efforts over the last decades, efficient fluorination and fluoroalkylation methods have emerged to prepare previously challenging molecules decorated with fluorine atoms or fluorinated groups which make them practically useful [11–14]. A building-block approach remains an alternative strategy to the synthesis of fluorine-containing compounds. This complementary method takes advantage of specific reagents featuring original fluorinated motives and/or functional groups which affords more complex derivatives via conventional functionalization or (hetero)cyclization [15–17]. Among these reagents, trifluoromethylketimines have drawn much research interest in recent years as key starting materials for the synthesis of trifluoromethyl-substituted amines [18–19], α-amino acids [20–23] as well as nitrogen-containing heterocyclic compounds [24–29]. It should be noted that the presence of a strong electron-withdrawing trifluoromethyl group is responsible for the sufficient reactivity of the electrophilic ketimine function with various carbon nucleophiles in these reactions.

Recently, the decarboxylative addition of malonic acid mono(thio)esters to aldehydes and imines has become an increasingly popular synthetic strategy [28,30–36]. However, the utility of trifluoromethylketimines as electrophilic substrates in this reaction remains underinvestigated. The only published work from the group of Ma described the development of a chiral thiourea-catalyzed enantioselective decarboxylative Mannich reaction of malonic acid monoesters with 4-trifluoromethylquinazolin-2(1*H*)-ones as heterocyclic trifluoromethylketimine substrates for the preparation of enantioenriched 3,4-dihydroquinazolin-2(1*H*)-ones and the anti-HIV drug DPC 083 [28]. No examples of any ketimines reacting directly with malonic acid have been reported so far.

**Figure 1:**
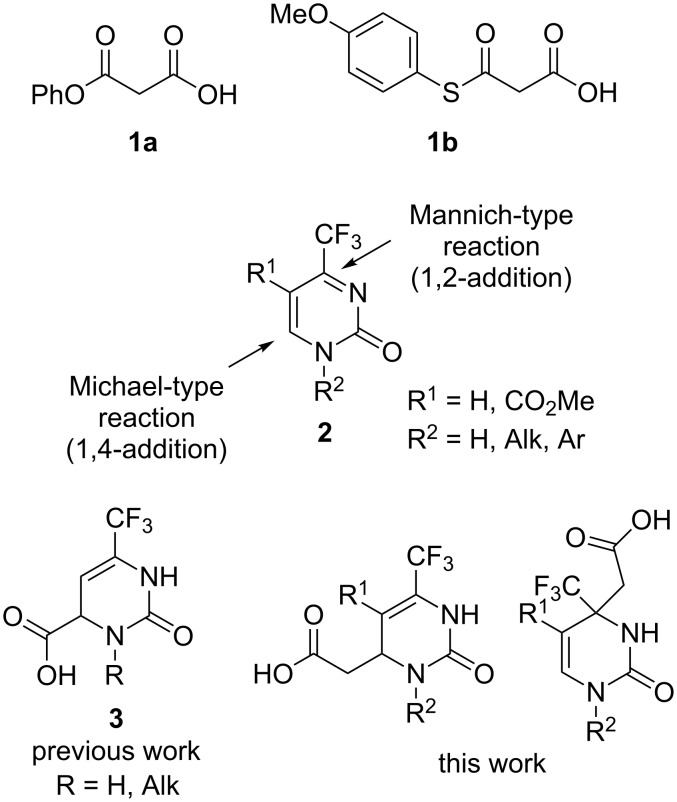
Summary of the present study.

Here we present the results of the decarboxylative addition of malonic acid, malonic monoester **1a** and thioester **1b** to 4-trifluoromethylpyrimidin-2(1*H*)-ones **2** (Figure 1). These compounds are unique heterocyclic conjugated trifluoromethylketimines with two competing electrophilic centers which can enable either Michael- or Mannich-type nucleophilic additions. As found in our previous studies, organocatalytic addition of acetone [37], nitromethane [38] and trimethylsilyl cyanide [39] in most cases can be performed regioselectively after optimization of the reaction conditions (temperature, solvent, time and catalyst nature). In general, under kinetic reaction control, the Michael-type 1,4-adducts are the predominant products while under thermodynamic control, the regioisomeric Mannich-type 1,2-adducts are more likely to be formed. These observations allowed us to develop selective methods for the synthesis of functionalized partially saturated 4-trifluoromethyl-substituted pyrimidin-2(1*H*)-ones, in particular, 4,5-dihydroorotic acid analogues **3** [39]. Here we report the preparation of acid **3** homologues (with a methylene linker between the carboxylic group and the pyrimidine ring) and their isomers resulting from two alternative regioselective pathways for the decarboxylative nucleophilic addition of malonic acid and its mono(thio)esters.

## Results and Discussion

We first screened organic base catalysts, solvents and temperature in the decarboxylative addition of malonic acid (nucleophilic component) to 1-methyl-4-trifluoromethylpyrimidin-2(1*H*)-one (**2a**, the simplest model substrate) aiming to find the optimal organocatalytic reaction conditions (Table 1). In the preliminary experiments, it was established that the reaction was quite slow; heating and a 5-fold excess of malonic acid were required to reach a reasonable conversion. Additionally, it was found that a stoichiometric amount of a model catalyst, triethylamine (TEA), was necessary for the reaction to proceed efficiently. Thus, heating the reaction mixture in toluene at 80 °C for 18 h in the presence of 1 equivalent of TEA resulted in a satisfactory 84% conversion and led to the Mannich-type product, (1-methyl-2-oxo-4-trifluoromethyl-1,2,3,4-tetrahydropyrimidin-4-yl)acetic acid (**4a**), along with a small amount of the Michael-type regioisomer **5a** (Table 1, entry 1). The reaction course and the ratio of the regioisomers formed were conveniently monitored by ^19^F NMR spectroscopy. Acid **4a** precipitated in pure form on evaporating toluene and treating the residue with diluted hydrochloric acid. Performing the reaction in a more polar solvent such as THF drastically shifted the regioselectivity to the Michael-type adduct formation (Table 1, entry 2). Using DMSO as solvent and heating the reaction at 80 °C provided exclusively (3-methyl-2-oxo-6-trifluoromethyl-1,2,3,4-tetrahydropyrimidin-4-yl)acetic acid (**5a**) in high isolated yield (Table 1, entry 3). Methanol proved to be an unsuitable solvent for this reaction in terms of both conversion and selectivity (Table 1, entry 4). Likewise, diisopropylethylamine (DIEA) and 1,8-diazabicyclo[5.4.0]undec-7-ene (DBU) were found to be not superior to TEA as catalysts (Table 1, entries 5 and 6). Unfortunately, quinine and the chiral quinine-derived thiourea organocatalyst QT in toluene led to a mixture of racemic products along with 19% and 38% of unreacted starting material, respectively (Table 1, entries 7 and 8).

**Table 1:** Screening of the reaction conditions for organic base-catalyzed malonic acid addition to 1-methyl-4-trifluoromethylpyrimidin-2(1*H*)-one (**2a**).

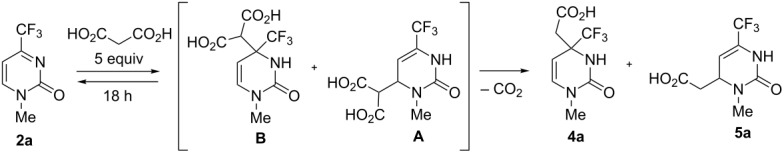						

entry	base (1 equiv)	solvent	temp. (°C)	conv. (%)	**4a**:**5a** ratio (%)	product (isolated yield, %)

1	TEA	toluene	80	84	92:8	**4a** (68)
2	TEA	THF	65	97	13:87	**5a** (63)
3	TEA	DMSO	80	94	1:99	**5a** (85)
4	TEA	MeOH	63	46	21:79	–
5	DIEA	toluene	80	83	88:12	**4a** (66)
6	DBU	toluene	80	80	85:15	–
7	quinine	toluene	80	81	47:53^a^	–
8	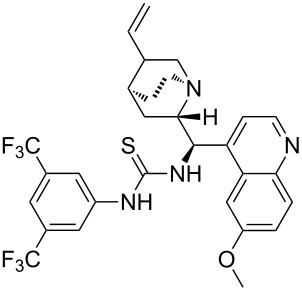 QT	toluene	80	62	34:66^a^	–

^a^The two regioisomers were racemic.

As seen from the screening results, the reaction regioselectivity is easily solvent controlled. Non-polar toluene is the preferential solvent for the Mannich-type decarboxylative addition to the C=N double bond while polar DMSO promotes the highly selective Michael-type addition to the C=C double bond. These observations are explained by the fact that the initially formed (kinetically controlled) Michael-type dicarboxylate adduct **A** is much more stable in a low-polar than in a high-polar solvent (Table 1). In the former case, the long-living intermediate **A** is gradually converted, via the reversible first reaction step, into the energetically advantageous (thermodynamically controlled) Mannich-type adduct **B**, followed by rapid irreversible decarboxylation of **B** into compound **4a**. Contrastingly, in a high-polar solvent, the intermediate **A** is so labile that it undergoes decarboxylation to product **5a** rather than rearrangement to **B**. The proposed reaction mechanism is supported by the known effect of solvent polarity on the decarboxylation rate of malonic acid derivatives which was claimed to be faster in polar media [40].

To study the substrate scope of the regioselective additions of malonic acid, we introduced substituted pyrimidones **2b**–**m** in the reaction and performed it under optimal conditions using toluene or DMSO as solvent and TEA (1 equiv) as catalyst (Table 2). The alkyl substituent at the nitrogen atom of the substrate had no significant effect on the reaction course. In all cases, both regioisomers, **4b**–**i** and **5b**–**i**, were isolated in modest to high yields (Table 2, entries 1–16). The presence of the ester functionality at position 5 of the heterocycle led to product mixtures if toluene was used as solvent so that products **4j**–**m** could not be obtained selectively and separated. In DMSO solution, the corresponding Michael-type adducts **5j**–**m** were smoothly formed and obtained in 75–83% isolated yields (Table 2, entries 17–20). 4-Trifluoromethylpyrimidin-2(1*H*)-ones **2** lacking a substituent at position 1 (structures not shown) were found to be completely unreactive in the decarboxylative reaction under study.

With the aim of preparing the corresponding *N*1(3)-unsubstituted products **4j** and **5n**,**o**, we utilized *N*1(3)-(4-methoxybenzyl) derivatives **4i**, **5i**, **5k** in trifluoroacetic acid (TFA); the resulting cleavage of the 4-methoxybenzyl (PMB) group afforded the target compounds in good yields (see Table 2).

**Table 2:** Regioselective decarboxylative addition of malonic acid to 4-trifluoromethylpyrimidin-2(1*H*)-ones **2b–m** and preparation of *N*1(3)-unsubstituted compounds **4j** and **5n,o**.

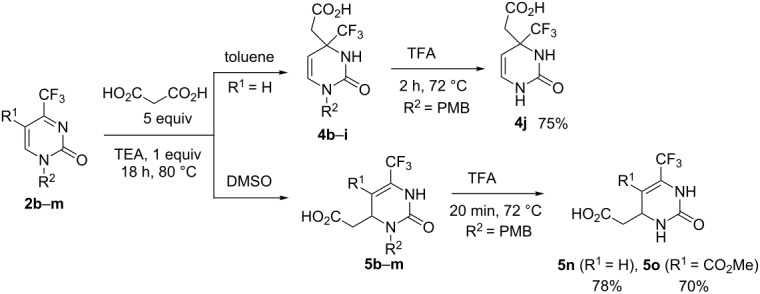					

entry	comp. **2**	R^1^	R^2^	isol. product	yield (%)

1	**b**	H	Et	**4b**	62
2	**b**	H	Et	**5b**	58
3	**c**	H	*n*-Bu	**4c**	67
4	**c**	H	*n*-Bu	**5c**	55
5	**d**	H	Me_2_CHCH_2_	**4d**	57
6	**d**	H	Me_2_CHCH_2_	**5d**	51
7	**e**	H	MeOCH_2_CH_2_	**4e**	59
8	**e**	H	MeOCH_2_CH_2_	**5e**	64
9	**f**	H	CH_2_=CHCH_2_	**4f**	73
10	**f**	H	CH_2_=CHCH_2_	**5f**	82
11	**g**	H	Bn	**4g**	68
12	**g**	H	Bn	**5g**	89
13	**h**	H	4-FC_6_H_4_CH_2_	**4h**	60
14	**h**	H	4-FC_6_H_4_CH_2_	**5h**	80
15	**i**	H	4-MeOC_6_H_4_CH_2_	**4i**	65
16	**i**	H	4-MeOC_6_H_4_CH_2_	**5i**	82
17	**j**	CO_2_Me	4-FC_6_H_4_CH_2_	**5j**	75
18	**k**	CO_2_Me	4-MeOC_6_H_4_CH_2_	**5k**	83
19	**l**	CO_2_Me	4-ClC_6_H_4_	**5l**	81
20	**m**	CO_2_Me	4-MeOC_6_H_4_	**5m**	81

Next we studied the decarboxylative addition of reagent **1a** to model substrate **2a** (Table 3) to compare the reactivity of malonic acid and its monophenyl ester **1a**. It was proved again that the reaction proceeded sufficiently fast in toluene only in the presence of a stoichiometric amount of TEA or DIEA (Table 3, entries 1 and 2). Under these conditions the reaction provided the Michael-type adduct, phenyl 2-(3-methyl-2-oxo-6-trifluoromethyl-1,2,3,4-tetrahydropyrimidin-4-yl)acetate (**6a**). The presence of DBU caused substantial decarboxylation of starting reagent **1a** (Table 3, entry 3). This unwanted process necessitated using of up to 6 equivalents of **1a** to reach a reasonable conversion with TEA as catalyst. Quinine and QT were again found to be ineffective to promote the enantioselective reaction (Table 3, entries 4 and 5). In contrast to the reaction with malonic acid under similar conditions, just trace amounts of regioisomeric Mannich-type adduct **7a** were detected. Presumably, in this case, the kinetically controlled Michael-type intermediate **C** is even far more prone to decarboxylation than the dicarboxylate intermediate **A** (Table 1) and hence, the reaction is sufficiently regioselective irrespective of the solvent polarity (Table 3, entries 1, and 6–9). Performing the reaction in toluene in the presence of TEA (1 equiv) at 80 °C for 4 hours gave the best result in terms of regioselectivity and yield of **6a** (Table 3, entry 1), virtually the only product formed in all the solvents used here (as evidenced by ^19^F NMR monitoring).

**Table 3:** Screening of the reaction conditions for organic base-catalyzed malonic acid monophenyl ester (**1a**) addition to 1-methyl-4-trifluoromethylpyrimidin-2(1*H*)-one (**2a**).

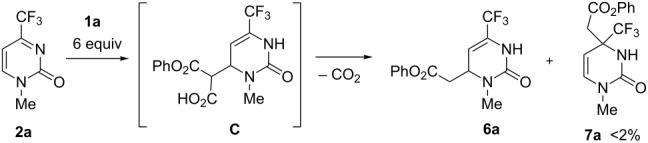						

entry	solvent	base	temp. (°C)	time (h)	conv.	yield **6a** (%)^a^

1	toluene	TEA	80	4	98	81
2	toluene	DIEA	80	4	96	77
3	toluene	DBU	80	4	10	–
4	toluene	quinine	80	4	92	68^b^
5	toluene	QT	80	4	78	55^b^
6	CH_2_Cl_2_	TEA	40	8	94	75
7	THF	TEA	66	8	90	74
8	dioxane	TEA	80	4	91	80
9	DMSO	TEA	80	4	93	81

^a^The regioisomeric product **7a** was formed in a negligible amount in all cases; ^b^Racemic product.

The addition of malonic acid monophenyl ester (**1a**) to substituted pyrimidones **2b**–**e** carried out in the presence of TEA in toluene for 8 h has shown that a substituent at position 1 of the pyrimidine ring can significantly influence the progress of the reaction (Table 4). Thus, *N*1-alkyl-substituted compounds **2b**–**e** exhibited a lower reactivity compared to **2a** and the corresponding products **6b**–**e** were not isolated due to low conversion and regioselectivity (Table 4, entries 1–4). These are likely caused by the enhanced steric hindrance around the neighboring electrophilic position 6 and also the lowered electrophilicity of the reaction center. Consequently, the nucleophilic attack on the C=N double bond becomes equally probable thus leading to the loss of regioselectivity. Fortunately, allyl and various benzyl or phenyl substituents in derivatives **2f**–**m** allowed the regioselective synthesis of products **6f**–**m** in high yields (Table 4, entries 5–12). We found that the ester group at position 5 significantly increases the electrophilicity of the endocyclic C=C double bond giving rise to faster addition of **1a** and higher regioselectivity of products **6j**–**m** (Table 4, entries 9–12). Like *N*3-unsubstituted compounds **5n**,**o**, their phenyl ester analogues **6n**,**o** were obtained by the cleavage of the *N*3-PMB substituent on short heating in TFA (see Table 4). It has been shown that acids **4a**,**f**–**m** can be synthesized alternatively by alkaline hydrolysis of esters **6a**,**f**–**m** (see Supporting Information File 1 for full experimental data). The ester group at position 5 remained intact during the hydrolysis.

**Table 4:** Regioselective decarboxylative addition of malonic acid monophenyl ester (**1a**) to 4-trifluoromethylpyrimidin-2(1*H*)-ones **2b–m** and preparation of *N*3-unsubstituted compounds **6n**,**o**.

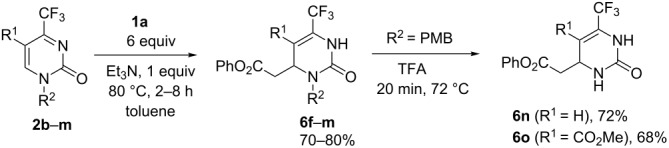						

entry	comp. **2**, **6**	R^1^	R^2^	time (h)	conv.	yield **6** (%)

1	**b**	H	Et	8	50	–
2	**c**	H	*n*-Bu	8	55	–
3	**d**	H	Me_2_CHCH_2_	8	49	–
4	**e**	H	MeOCH_2_CH_2_	8	44	–
5	**f**	H	CH_2_=CHCH_2_	4	97	75
6	**g**	H	Bn	4	99	70
7	**h**	H	4-FC_6_H_4_CH_2_	4	99	74
8	**i**	H	4-MeOC_6_H_4_CH_2_	4	98	69
9	**j**	CO_2_Me	4-FC_6_H_4_CH_2_	2	99	80
10	**k**	CO_2_Me	4-MeOC_6_H_4_CH_2_	2	97	71
11	**l**	CO_2_Me	4-ClC_6_H_4_	2	98	73
12	**m**	CO_2_Me	4-MeOC_6_H_4_	2	99	75

Malonic acid monothioesters are known to be more reactive C-nucleophiles than the corresponding esters [41]. Therefore, we studied the decarboxylative addition of compound **1b** as representative example to substrates **2a**–**m** (Table 5). They were found to furnish Michael-type addition products **8a**–**m** on heating in CH_2_Cl_2_ at 40 °C in excellent yields. Moreover, 3 equivalents excess of **1b** was sufficient for the reaction to be completed within 1–3 hours.

**Table 5:** Regioselective decarboxylative addition of malonic acid mono-4-methoxyphenyl thioester (**1b**) to 4-trifluoromethylpyrimidin-2(1*H*)-ones **2a–m**.

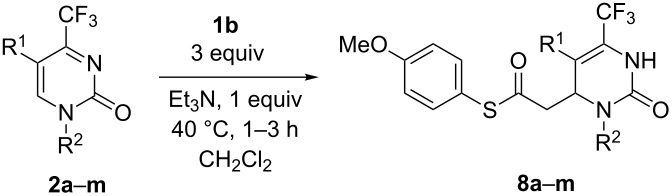					

entry	comp. **2**, **8**	R^1^	R^2^	time (h)	yield **8** (%)

1	**a**	H	Me	3	83
2	**b**	H	Et	3	71
3	**c**	H	*n*-Bu	3	77
4	**d**	H	Me_2_CHCH_2_	3	77
5	**e**	H	MeOCH_2_CH_2_	3	75
6	**f**	H	CH_2_=CHCH_2_	3	73
7	**g**	H	Bn	3	74
8	**h**	H	4-FC_6_H_4_CH_2_	3	71
9	**i**	H	4-MeOC_6_H_4_CH_2_	3	70
10	**j**	CO_2_Me	4-FC_6_H_4_CH_2_	1	77
11	**k**	CO_2_Me	4-MeOC_6_H_4_CH_2_	1	75
12	**l**	CO_2_Me	4-ClC_6_H_4_	1	81
13	**m**	CO_2_Me	4-MeOC_6_H_4_	1	72

Satisfactory conversion and regioselectivity were achieved even with substrates **2b**–**e** bearing ethyl, *n*-butyl, isobutyl and 2-methoxyethyl substituents which demonstrated low reactivity in the addition reaction with ester analogue **1a**. It can thus be inferred that the substituents R^1^ and R^2^ have almost no impact on the outcome of the decarboxylative addition provided a highly reactive nucleophilic component such as malonic acid monothioester **1b** is used.

Importantly, representative compounds **6a**,**j** and **8f** readily reacted with benzylamine thus showing the possibility of esters **6** and thioesters **8** to be convenient amine acylating agents [32] and, hence, building blocks for direct preparation of the amide derivatives (see Supporting Information File 1 for examples of the corresponding amide syntheses).

**Scheme 1:**
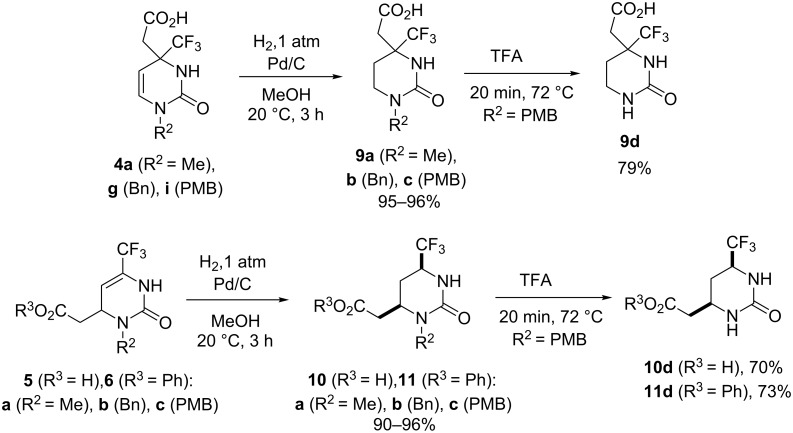
Hydrogenation of compounds **4–6** and preparation of *N*1(3)-unsubstituted compounds **9–11d**.

In a next set of experiments, the endocyclic C=C double bond of the decarboxylative adducts **4**–**6** were hydrogenated to prepare compounds with a saturated 3,4,5,6-tetrahydropyrimidin-2(1*H*)-one ring functionalized with an acetic acid moiety and a trifluoromethyl group. Thus, the acids **4a**,**g**,**i** quantitatively yielded reduced products **9a–c** under mild catalytic conditions (when reacted with hydrogen at atmospheric pressure and room temperature for 3 hours in the presence of 10% Pd/C catalyst) as shown in Scheme 1. The simplest acetic acid derivative **9d** was synthesized from **9c** in a good yield by using the general procedure for *N*1-PMB cleavage. Likewise, regioisomeric acids **5a**,**g**,**i** and their phenyl esters **6a**,**g**,**i** were reduced to the respective saturated compounds **10a**–**c** and **11a**–**c**. In this case a high hydrogenation *cis*-stereoselectivity is provided when the Pd/C catalyst loading is smaller than 20 weight % (otherwise the reaction proceeds too fast leading to diastereomeric mixtures with a *cis*- to *trans*-ratio of up to 3:1).

**Figure 2:**
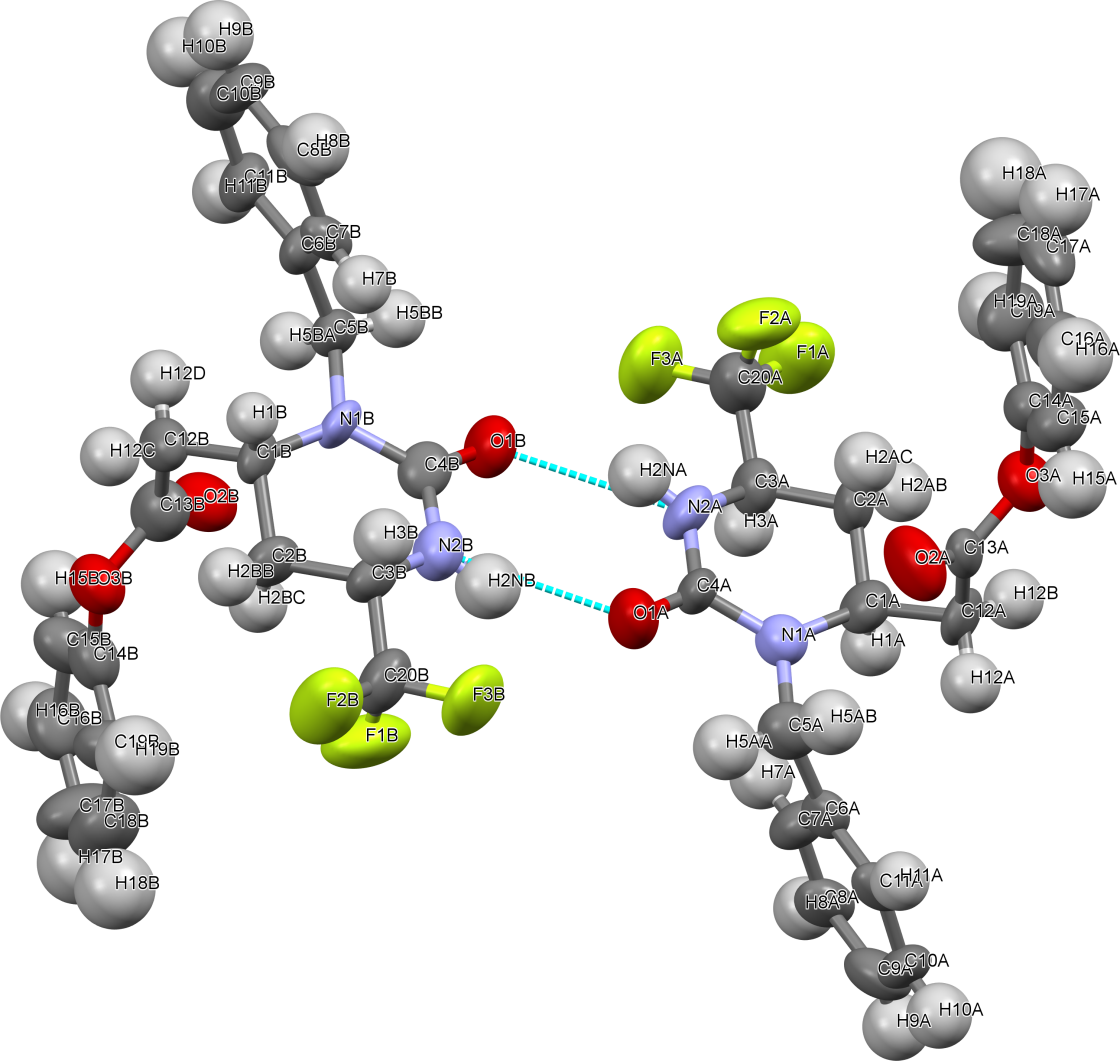
Molecular structure of compound **11b**. Two enantiomers form a heterochiral dimer in the crystal state. Intermolecular hydrogen bonds in the dimer are shown as dashed lines. Thermal ellipsoids are defined at 50% probability.

The relative *cis*-configuration of the CF_3_ and CH_2_COOPh substituents in the prepared phenyl (2-oxo-6-trifluoromethylhexahydropyrimidin-4-yl)acetates **11a**–**c** was unambiguously corroborated by a single-crystal X-ray diffraction study of compound **11b** (Figure 2, see Supporting Information File 1 for full structure description and experimental data). The configuration-preserving conversion of ester **11b** into acid **10b** by simple alkaline hydrolysis has also confirmed the *cis*-geometry for acids **10a**–**c** obtained by direct hydrogenation of compounds **5a**,**g**,**i** (see Supporting Information File 1). *N*3-Unsubstituted compounds **10d** and **11d** with the preserved *cis*-configuration of the substituents were readily prepared from the corresponding *N*3-PMB derivatives **10c** and **11c** by using the general procedure for *N*1(3)-PMB cleavage (see Scheme 1).

## Conclusion

In conclusion, it has been demonstrated that the efficient and highly regioselective organocatalytic decarboxylative addition of malonic acid or its derivatives to 4-trifluoromethylpyrimidin-2(1*H*)-ones **2** is perfectly feasible with a precise control of the reaction conditions. A remarkable solvent effect has been observed which governs the ratio of the resulting regioisomeric decarboxylated adducts **4** and **5** and allows their preparative selective isolation. This effect may well be attributed to a two-step mechanism of the decarboxylative nucleophilic addition which is characterized by a faster decarboxylation of kinetically-controlled Michael-type intermediates in high-polar solvents.

Though malonic monoester **1a** appears to be similar to malonic acid in reactivity towards compounds **2**, it produces exclusively Michael-type adducts **6** regardless of the reaction conditions used. Likewise, the more reactive malonic mono thioester **1b**, when reacted with a broader scope of substrates **2** under milder conditions, gives rise only to analogous Michael-type products **8**. In general, the reactivity of substrates **2** can be increased by introduction of the ester functionality at position 5 as well as allyl, various benzyl or phenyl substituents at position 1 of the pyrimidine core. Notably, esters **6** and thioesters **8** are remarkable for their potential application as smooth amine acylating agents.

It has been shown that the 3,4-dihydropyrimidin-2(1*H*)-one ring in both Mannich- and Michael-type products **4** and **5**, **6** can be readily hydrogenated under mild catalytic conditions to furnish saturated compounds **9** and **10**, **11**, respectively. Products **10** and **11** featuring two stereogenic centers were obtained only as *cis*-isomers.

*N*1(3)-Unsubstituted products **4j**, **5n**,**o**, **6n**,**o**, and **9**–**11d**, unavailable by direct decarboxylative addition, are readily accessible by TFA-mediated cleavage of the corresponding *N*1(3)-PMB substituted precursors.

## Supporting Information

File 1 Experimental procedures, characterization data and X-ray structure determination for compound **11b**.

File 2 Copies of the ^1^H, ^13^C, and ^19^F NMR spectra.
