# Supplementary material for: Regioselective decarboxylative addition of malonic acid and its mono(thio)esters to 4-trifluoromethylpyrimidin-2(1H)-ones
Source: Beilstein J Org Chem. 2017 Dec 7;13:2617–25. doi: 10.3762/bjoc.13.259 (PMC5727768; doi:10.3762/bjoc.13.259)
Supplement: File 1 — Experimental procedures, characterization data and X-ray structure determination for compound 11b. [file Beilstein_J_Org_Chem-13-2617-s001.pdf]

Supporting Information File 1

for

Regioselective decarboxylative addition of malonic acid and its mono(thio)esters to 4-trifluoromethylpyrimidin-2(1*H*)-ones

Sergii V. Melnykov[1], Andrii S. Pataman[2], Yurii V. Dmytriv[2,3], Svitlana V. Shishkina[4,5], Mykhailo V. Vovk[1] and Volodymyr A. Sukach*[1]

Address: [1]Institute of Organic Chemistry, National Academy of Sciences of Ukraine, 5 Murmanska str., Kyiv 02660, Ukraine, [2]Enamine LTD, 78 Chervonotkats‘ka str., Kyiv 02094, Ukraine, [3]National Technical University of Ukraine “Igor Sikorsky Kyiv Polytechnic Institute”, 37 Peremohy ave., Kyiv 03056, Ukraine, [4]STC ‘‘Institute for Single Crystals’’, National Academy of Sciences of Ukraine, 60 Nauky ave., Kharkiv 61001, Ukraine and [5]Department of Inorganic Chemisrtry, V.M. Karasin Kharkiv National University, 4 Svobody sq, Kharkiv 61122, Ukraine

Email: Volodymyr A. Sukach - vsukach@gmail.com

* Corresponding author

Experimental procedures, characterization data and X-ray structure determination for compound 11b

Content

1. General methods……………………………………………………………………......S2

2. General procedures for the synthesis of compounds 4–6,8–11 and characterization d…………………………………………………………………………S2

3. Representative procedure for the synthesis of 2-[2-oxo-6-(trifluoromethyl)-1,2,3,4-tetrahydropyrimidin-4-yl]acetamides……………………………………………………...S30

4. Representative procedure for the hydrolysis of compounds 6 and 11…………..S31

5. X-ray structure determination for compound 11b……………………………………S32

6. References………………………………..……………………………………………...S34

1. General methods

All chemicals were obtained from commercially available sources (Sigma-Aldrich, Enamine Ltd.) and used without further purification. All solvents were purified by standard methods. Melting points are uncorrected. ^{19}F NMR, ^{1}H NMR and ^{13}C NMR spectra were recorded on Varian VXR-300, Varian Mercury-400 or Bruker Avance DRX-500 spectrometers with TMS or CCl_3F as an internal standard. Multiplets were assigned as s (singlet), d (doublet), t (triplet), dd (doublet of doublet), q (quartet), m (multiplet) and br s (broad singlet). LC–MS spectra were recorded on an Agilent 1100 Series high performance liquid chromatograph equipped with a diode matrix with an Agilent LC\MSD SL selective mass detector. Chromatography was performed on a low-pressure (up to 3 atm) CombiFlash Companion apparatus equipped with a UV detector (eluent hexane/methyl *tert*-butyl ether). Microanalyses were performed in the Microanalytical Laboratory of the Institute of Organic Chemistry, National Academy of Sciences of Ukraine. Compounds **1a** [1], **1b** [2], **2a-i** [3,4], **2j–m** [5,6] were prepared according to literature procedures.

2. General procedures for the synthesis of compounds 4–6,8–11 and characterization data

2.1. General procedure for the synthesis of compounds 4a–m.

To the solution of compound **2a–i** (3 mmol) and malonic acid (1.56 g, 15 mmol) in toluene (10 mL) triethylamine (0.3 g, 3 mmol) was added. The mixture was stirred at 80 °C for 18 h. After completion of the reaction the mixture was cooled and washed with 0.4 M hydrochloric acid (2 × 20 mL) and brine (2 × 20 mL). The organic layer was dried over anhydrous sodium sulfate and the solvent was evaporated. The obtained residue was crystallized from hexane/methyl *tert*-butyl ether 3:1.

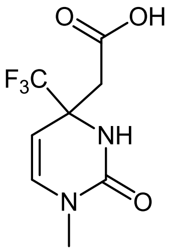

2-(1-Methyl-2-oxo-4-(trifluoromethyl)-1,2,3,4-tetrahydropyrimidin-4-yl)acetic acid (4a). In this case the solvent was evaporated after completion of the reaction and the residue was treated with 0.4 M hydrochloric acid (5 mL). After 2 h at 20 °C the precipitate formed was filtered, washed with water (5 mL) and dried on air. White solid, yield: 0.49 g (68%), m.p. 153–155 °C. ^{1}H NMR (400 MHz, DMSO-d$_6$): δ = 2.54–2.61 (m, 1H, CH_2), 2.61–2.74 (m, 1H, CH_2), 2.91 (s, 3H, CH_3), 4.69 (s, 1H, CH), 6.47 (d, *J* = 4.66 Hz, 1H, CH), 7.63 (s, 1H, NH), 12.52 (br s, 1H, COOH) ppm; ^{13}C NMR (101 MHz, DMSO-d$_6$): δ = 34.2, 39.2, 60.9 (q, *J* = 29.3 Hz, C-4), 94.0, 125.4 (q, *J* = 287.9 Hz, CF_3), 134.5, 152.4, 169.8 ppm; ^{19}F NMR (376 MHz, DMSO-d$_6$): δ = −82.44 (s, CF_3) ppm. $C_8H_9F_3N_2O_3$ (238.16). Found, %: C, 40.18; H, 3.82; N, 11.76. Calculated, %: C, 40.34; H, 3.81; N, 11.76. LCMS: $[MH]^+$ 239.

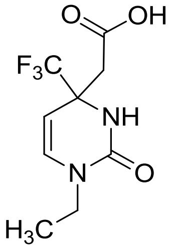

2-(1-Ethyl-2-oxo-4-(trifluoromethyl)-1,2,3,4-tetrahydropyrimidin-4-yl)acetic acid (4b). White solid, yield: 0.47 g (62%), m.p. 151–153 °C. ^{1}H NMR (400 MHz, DMSO-d$_6$): δ = 1.01 (t, *J* = 6.53 Hz, 3H, CH_3), 2.54–2.60 (m, 1H, CH_2), 2.66 (d, *J* = 16.79 Hz, 1H, CH_2), 3.22–3.52 (m, 2H, CH_2), 4.71 (d, *J* = 7.48 Hz, 1H, CH), 6.50 (d, *J* = 7.46 Hz, 1H, CH), 7.55 (s, 1H, NH), 12.46 (br s, 1H, COOH) ppm; ^{13}C NMR (101 MHz, DMSO-d$_6$): δ = 14.6, 39.2, 41.3, 60.7 (q, *J* = 28.6 Hz, C-4), 94.4, 125.1 (q, *J* = 286.8 Hz, CF_3), 133.2, 151.9, 169.9 ppm; ^{19}F NMR (376 MHz, DMSO-d$_6$): δ = −82.42 (s, CF_3) ppm. $C_9H_{11}F_3N_2O_3$ (252.19). Found, %: C, 42.75; H, 4.51; N, 11.17. Calculated, %: C, 42.86; H, 4.40; N, 11.11. LCMS: $[MH]^+$ 253.

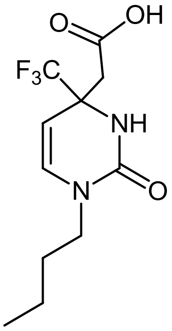

2-(1-Butyl-2-oxo-4-(trifluoromethyl)-1,2,3,4-tetrahydropyrimidin-4-yl)acetic acid (4c). White solid, yield: 0.56 g (67%), m.p. 103–105 °C. ^{1}H NMR (400 MHz, DMSO-d_6): δ = 0.85 (t, *J* =7.03 Hz, 3H, CH_3), 1.15–1.30 (m, 2H, CH_2), 1.32–1.49 (m, 2H, CH_2), 2.55–2.60 (m, 1H, CH_2), 2.65 (d, *J* =16 Hz, 1H, CH_2), 3.20–3.30 (m, 1H, CH_2), 3.38–3.47 (m, 1H, CH_2), 3.40 (br s, H_2O+COOH) 4.68 (d, *J* =8.03 Hz, 1H, CH), 6.48 (d, *J* =8.03 Hz, 1H, CH), 7.56 (br s, 1H, NH) ppm; ^{13}C NMR (101 MHz, DMSO-d_6): δ = 14.1, 19.4, 31.3, 39.1, 45.9, 60.7 (q, *J* = 29.3 Hz, C-4), 94.1, 125.5 (q, *J* = 286.8 Hz, CF_3), 133.6, 152.0, 169.8 ppm; ^{19}F NMR (376 MHz, DMSO-d_6): δ = −82.44 (s, CF_3) ppm. $C_{11}H_{15}F_3N_2O_3$ (280.24). Found, %: C, 47.43; H, 5.40; N, 10.00. Calculated, %: C, 47.14; H, 5.39; N, 10.00. LCMS: $[MH]^+$ 281.

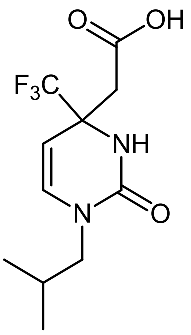

2-(1-Isobutyl-2-oxo-4-(trifluoromethyl)-1,2,3,4-tetrahydropyrimidin-4-yl)acetic acid (4d). White solid, yield: 0.5 g (57%), m.p. 155–157 °C. ^{1}H NMR (400 MHz, DMSO-d_6): δ = 0.80 (t, *J* = 5.6 Hz, 6H, CH_3), 1.73–1.91 (m, 1H, CH), 2.55 (d, *J* = 15.56 Hz, 1H, CH_2), 2.66 (d, *J* = 15.06 Hz, 1H, CH_2), 3.01 (dd, *J* = 13.55, 7.03 Hz, 1H, CH_2), 3.25–3.30 (m, 1H, CH_2), 4.66 (d, *J* = 8.03 Hz, 1H, CH), 6.47 (d, *J* = 8.03 Hz, 1H, CH), 7.58 (s, 1H, NH), 12.46 (br s, 1H, COOH) ppm; ^{13}C NMR (126 MHz, DMSO-d_6): δ = 19.9, 28.3, 39.2, 53.4, 60.7 (q, *J* = 28.9 Hz, C-4), 93.7, 125.14 (q, *J* = 286.4 Hz, CF_3), 134.1, 152.0, 169.8 ppm; ^{19}F NMR (376 MHz, DMSO-d_6): δ = −82.42 (s, CF_3) ppm. $C_{11}H_{15}F_3N_2O_3$ (280.24). Found, %: C, 47.12; H, 5.35; N, 10.09. Calculated, %: C, 47.14; H, 5.39; N, 10.00. LCMS: $[MH]^+$ 281.

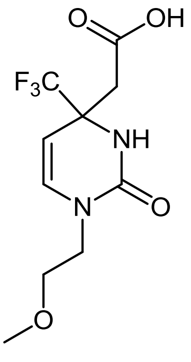

2-(1-(2-Methoxyethyl)-2-oxo-4-(trifluoromethyl)-1,2,3,4-tetrahydropyrimidin-4-yl)acetic acid (4e). White solid, yield: 0.5 g (59%), m.p. 105–107 °C. ^{1}H NMR (400 MHz, DMSO-d_6): δ = 2.56 (d, *J* = 14.92 Hz, 1H, CH_2), 2.66 (d, *J* = 13.99 Hz, 1H, CH_2), 3.23 (s, 3H, CH_3), 3.36–3.45 (m, 3H, CH_2), 3.54–3.62 (m, 1H, CH_2), 4.68 (d, *J* = 8.03 Hz, 1H, CH), 6.47 (d, *J* = 8.40 Hz, 1H, CH), 7.63 (s, 1H, NH), 12.48 (br s, 1H, COOH) ppm; ^{13}C NMR (126 MHz, DMSO-d_6): δ = 39.2, 46.0, 58.6, 60.6 (q, *J* = 28.8 Hz, C-4), 70.9, 93.8, 125.5 (q,

J = 286.2 Hz, CF_3), 134.1, 151.9, 169.8 ppm; ^{19}F NMR (376 MHz, DMSO-d_6): δ = −82.32 (s, CF_3) ppm. $C_{10}H_{13}F_3N_2O_4$ (282.22). Found, %: C, 42.40; H, 4.65; N, 9.93. Calculated, %: C, 42.56; H, 4.64; N, 9.93. LCMS: $[MH]^+$ 283.

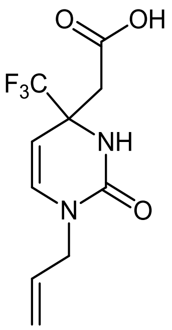

2-(1-Allyl-2-oxo-4-(trifluoromethyl)-1,2,3,4-tetrahydropyrimidin-4-yl)acetic acid (4f). White solid, yield: 0.58 g (73%), m.p. 160–163 °C. ^{1}H NMR (400 MHz, DMSO-d_6): δ = 2.57 (d, *J* = 14.56 Hz, 1H, CH_2), 2.68 (d, *J* = 14.56 Hz, 1H, CH_2), 3.91–4.06 (m, 2H, CH_2), 4.73 (d, J = 7.60 Hz, 1H, CH_2), 5.01–5.21 (m, 2H, CH, CH_2), 5.70–5.83 (m, 1H, CH), 6.39 (d, *J* = 7.96 Hz, 1H, CH), 7.68 (s, 1H, NH), 12.48 (br s, 1H, COOH) ppm; ^{13}C NMR (126 MHz, DMSO-d_6): δ = 39.1, 48.0, 60.9 (q, *J* = 29.0 Hz, C-4), 94.5, 116.0, 125.4 (q, *J* = 288.5 Hz, CF_3), 133.3, 134.6, 151.7, 169.8 ppm; ^{19}F NMR (376 MHz, DMSO-d_6): δ = −82.36 (s, CF_3) ppm. $C_{10}H_{11}F_3N_2O_3$ (264.20). Found, %: C, 45.60; H, 4.16; N, 10.56. Calculated, %: C, 45.46; H, 4.20; N, 10.60. LCMS: $[MH]^+$ 265.

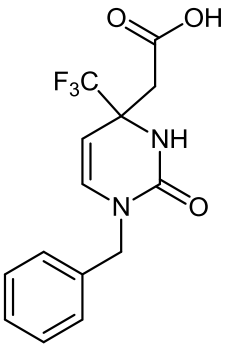

2-(1-Benzyl-2-oxo-4-(trifluoromethyl)-1,2,3,4-tetrahydropyrimidin-4-yl)acetic acid (4g). White solid, yield: 0.64 g (68%), m.p. 165–170 °C.^{1}H NMR (400 MHz, DMSO-d_6): δ = 2.61 (d, *J* = 14.92 Hz, 1H, CH_2), 2.72 (d, *J* = 14.92 Hz, 1H, CH_2), 4.55 (d, *J* = 15.86 Hz, 1H, CH_2), 4.65 (d, *J* = 15.86 Hz, 1H, CH_2), 4.76 (d, *J* = 6.40 Hz, 1H, CH), 6.53 (d, *J* = 7.60 Hz, 1H, CH), 7.20–7.35 (m, 5H, CH), 7.77 (s, 1H, NH), 12.53 (br s, 1H, COOH) ppm; ^{13}C NMR (126 MHz, DMSO-d_6): δ = 39.1, 49.1, 60.9 (q, *J* = 29.0 Hz, C-4), 94.8, 125.4 (q, *J* = 288.5 Hz, CF_3), 127.1, 127.4, 128.8, 133.6, 138.8, 152.1, 169.9 ppm; ^{19}F NMR (376 MHz, DMSO-d_6): δ = −82.29 (s, CF_3) ppm. $C_{14}H_{13}F_3N_2O_3$ (314.26). Found, %: C, 53.48; H, 4.15; N, 8.94. Calculated, %: C, 53.51; H, 4.17; N, 8.91. LCMS: $[MH]^+$ 315.

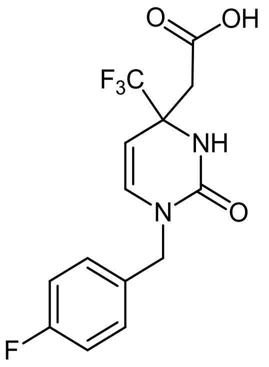

2-(1-(4-Fluorobenzyl)-2-oxo-4-(trifluoromethyl)-1,2,3,4-tetrahydropyrimidin-4-yl)acetic acid (4h). White solid, yield: 0.6 g (60%), m.p. 146–148 °C.^{1}H NMR (400 MHz, DMSO-d$_6$): δ = 2.63 (d, *J* = 15.06 Hz, 1H, CH_2), 2.74 (d, *J* = 15.06 Hz, 1H, CH_2), 4.56 (d, *J* = 15.06 Hz, 1H, CH_2), 4.64 (d, *J* = 16.06 Hz, 1H, CH_2), 4.78 (d, *J* = 8.03 Hz, 1H, CH), 6.56 (d, *J* = 8.03 Hz, 1H, CH), 7.16 (t, *J* = 9.03 Hz, 2H, CH), 7.23–7.38 (m, 2H, CH), 7.81 (s, 1H, NH), 12.74 (br s, 1H, COOH) ppm; ^{13}C NMR (101 MHz, DMSO-d$_6$): δ = 39.0, 48.5, 60.9 (q, *J* = 28.3 Hz, C-4), 94.9, 115.6 (d, *J* = 21.3 Hz), 125.4 (q, *J* = 287.6 Hz, CF_3), 129.3 (d, *J* = 8.1 Hz), 133.5, 135.0, 152.1, 161.8 (d, *J* = 242.1 Hz), 169.8 ppm; ^{19}F NMR (376 MHz, DMSO-d$_6$): δ = −82.33 (s, CF_3), −116.31 (s, CF) ppm. $C_{14}H_{12}F_4N_2O_3$ (332.25). Found, %: C, 50.56; H, 3.70; N, 8.37. Calculated, %: C, 50.61; H, 3.64; N, 8.43. LCMS: $[MH]^+$ 333.

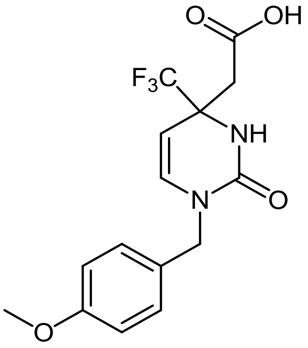

2-(1-(4-Methoxybenzyl)-2-oxo-4-(trifluoromethyl)-1,2,3,4-tetrahydropyrimidin-4-yl)acetic acid (4i). White solid, yield: 0.67 g (65%), m.p. 157–160 °C. ^{1}H NMR (400 MHz, DMSO-d$_6$): δ = 2.59 (d, *J* = 14.92 Hz, 1H, CH_2), 2.70 (d, *J* = 14.92 Hz, 1H, CH_2), 3.72 (s, 3H, CH_3), 4.45 (d, *J* = 14.92 Hz, 1H, CH_2), 4.57 (d, *J* = 15.86 Hz, 1H, CH_2), 4.74 (d, *J* = 7.46 Hz, 1H, CH), 6.50 (d, *J* = 7.46 Hz, 1H, CH), 6.87 (d, *J* = 8.39 Hz, 2H, CH), 7.17 (d, *J* = 8.39 Hz, 2H, CH), 7.71 (s, 1H, NH), 12.68 (br s, 1H, COOH) ppm; ^{13}C NMR (126 MHz, DMSO-d$_6$): δ = 39.1, 48.5, 55.5, 60.8 (q, *J* = 29.0 Hz, C-4), 94.7, 114.3, 125.4 (q, *J* = 288.5 Hz, CF_3), 128.7, 130.6, 133.4, 152.1, 158.9, 169.8 ppm; ^{19}F NMR (376 MHz, DMSO-d$_6$): δ = −82.29 (s, CF_3) ppm. $C_{15}H_{15}F_3N_2O_4$ (344.29). Found, %: C, 52.38; H, 4.40; N, 8.01. Calculated, %: C, 52.33; H, 4.39; N, 8.14. LCMS: $[MH]^+$ 345.

2.2. General procedure for the synthesis of compounds 5a–m.

To the solution of compound **2a–m** (3 mmol) and malonic acid (1.56 g, 15 mmol) in dimehtylsulfoxide (10 mL) triethylamine (0.3 g, 3 mmol) was added. The mixture was stirred at 80 °C for 18 h. After completion of the reaction the mixture was cooled, diluted with 0.4 M hydrochloric acid (20 mL) and the product was extracted with dichloromethane (3 × 10 mL). The organic layer was washed with brine (2 × 20 mL), dried over anhydrous sodium sulfate and the solvent was evaporated. The obtained residue was crystallized from hexane/methyl *tert*-butyl ether 1:1.

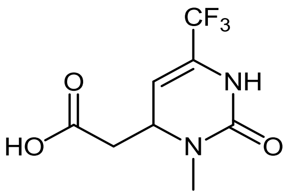

2-(3-Methyl-2-oxo-6-(trifluoromethyl)-1,2,3,4-tetrahydropyrimidin-4-yl)acetic acid (5a). White solid, yield: 0.61 g (85%), m.p. 152–154 °C.^{1}H NMR (400 MHz, DMSO-d$_6$): δ = 2.45–2.55 (m, 1H, CH$_2$), 2.65 (d, *J* = 19.59 Hz, 1H), 2.81 (s, 3H, CH$_3$), 4.35 (s, 1H, CH), 5.45 (s, 1H, CH), 9.35 (s, 1H, NH), 12.48 (br s, 1H, COOH) ppm; ^{13}C NMR (101 MHz, DMSO-d$_6$): δ = 32.7, 38.3, 54.6, 102.4, 120.3 (q, *J* = 272.7 Hz, CF$_3$), 127.6 (q, *J* = 35.4 Hz, C-4), 152.7, 171.9 ppm; ^{19}F NMR (376 MHz, DMSO-d$_6$): δ = −69.55 (s, CF$_3$) ppm. $C_8H_9F_3N_2O_3$ (238.16). Found, %: C, 40.24; H, 3.92; N, 11.75. Calculated, %: C, 40.34; H, 3.81; N, 11.76. LCMS: $[MH]^+$ 239.

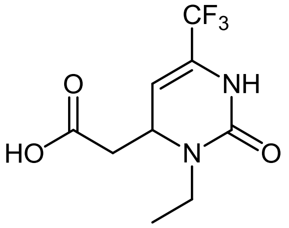

2-(3-Ethyl-2-oxo-6-(trifluoromethyl)-1,2,3,4-tetrahydropyrimidin-4-yl)acetic acid (5b). White solid, yield: 0.44 g (58%), m.p. 113–115 °C. ^{1}H NMR (400 MHz, DMSO-d$_6$): δ= 1.04 (br s, 3H, CH$_3$), 2.46–2.72 (m, 2H, CH$_2$), 2.90-3.10 (m, 1H, CH$_2$), 3.50-3.65 (m, 1H, CH$_2$), 4.38 (s, 1H, CH), 5.47 (s, 1H, CH), 9.31 (br s, 1H, NH), 12.51 (br s, 1H, COOH) ppm; ^{13}C NMR (126 MHz, DMSO-d$_6$): δ = 13.4, 39.2, 52.1, 55.3, 102.7, 120.2 (q, *J* = 272.2 Hz, CF$_3$), 127.7(q, *J* = 34.0 Hz, C-4), 152.4, 171.9 ppm; ^{19}F NMR (376 MHz, DMSO-d$_6$): δ= −69.55 (s, CF$_3$) ppm. $C_9H_{11}F_3N_2O_3$ (252.19). Found, %: C, 42.89; H, 4.40; N, 11.02. Calculated, %: C, 42.86; H, 4.40; N, 11.11. LCMS: $[MH]^+$ 253.

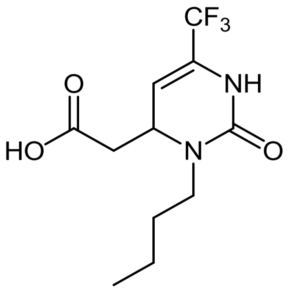

2-(3-Butyl-2-oxo-6-(trifluoromethyl)-1,2,3,4-tetrahydropyrimidin-4-yl)acetic acid (5c). White solid, yield: 0.46 g (55%), m.p. 111–113 °C. ^{1}H NMR (400 MHz, DMSO-d_6): δ = 0.86 (t, *J* = 7.53 Hz, 3H, CH_3), 1.23 (q, *J* = 7.03 Hz, 2H, CH_2), 1.31–1.57 (m, 2H, CH_2), 2.42–2.50 (m, 1H, CH_2), 2.63 (d, *J* = 15.56 Hz, 1H, CH_2), 2.79–2.96 (m, 1H, CH_2), 3.52–3.68 (m, 1H, CH_2), 4.34 (s, 1H, CH), 5.48 (s, 1H, CH), 9.33 (s, 1H, NH), 12.46 (br s, 1H, COOH) ppm; ^{13}C NMR (101 MHz, DMSO-d_6): δ = 14.1, 19.9, 29.8, 39.0, 44.3, 52.2, 102.7, 120.2 (q, *J* = 272.7 Hz, CF_3), 127.8 (q, *J* = 34.4 Hz, C-4), 152.7, 172.0 ppm; ^{19}F NMR (376 MHz, DMSO-d_6): −69.46 (s, CF_3) ppm. $C_{11}H_{15}F_3N_2O_3$ (280.24). Found, %: C, 47.12; H, 5.37; N, 10.04. Calculated, %: C, 47.14; H, 5.39; N, 10.00. LCMS: $[MH]^+$ 281.

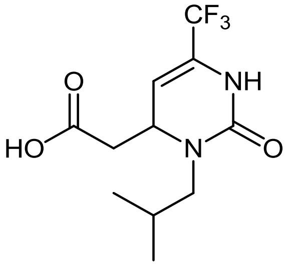

2-(3-Isobutyl-2-oxo-6-(trifluoromethyl)-1,2,3,4-tetrahydropyrimidin-4-yl)acetic acid (5d). White solid, yield: 0.43 g (51%), m.p. 111–113 °C. ^{1}H NMR (400 MHz, DMSO-d_6): δ = 0.77 (d, *J* = 6.53 Hz, 3H, CH_3), 0.85 (d, *J* = 6.53 Hz, 3H, CH_3), 1.75–1.97 (m, 1H, CH), 2.37–2.45 (m, 1H, CH_2), 2.55–2.69 (m, 2H, CH_2), 3.00–4.00 (br s, H_2O+COOH), 3.54 (dd, *J* = 13.55, 8.03 Hz, 1H, CH_2), 4.28 (s, 1H, CH), 5.54 (d, *J* = 4.02 Hz, 1H, CH), 9.38 (br s, 1H, NH) ppm; ^{13}C NMR (101 MHz, DMSO-d_6): δ = 20.07, 20.14, 26.5, 38.6, 51.4, 52.5, 102.9, 120.3 (q, *J* = 272.7 Hz, CF_3), 128.0 (q, *J* = 35.4 Hz, C-4), 153.1, 172.0 ppm; ^{19}F NMR (376 MHz, DMSO-d_6): δ= −69.34 (s, CF_3) ppm. $C_{11}H_{15}F_3N_2O_3$ (280.24). Found, %: C, 47.43; H, 5.46; N, 10.00. Calculated, %: C, 47.14; H, 5.39; N, 10.00. LCMS: $[MH]^+$ 281.

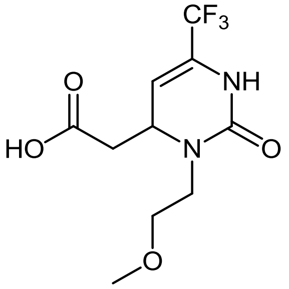

2-(3-(2-Methoxyethyl)-2-oxo-6-(trifluoromethyl)-1,2,3,4-tetrahydropyrimidin-4-yl)acetic acid (5e). White solid, yield: 0.54 g (64%), m.p. 116–118 °C.^{1}H NMR (400 MHz, DMSO-d$_6$): δ = 2.44–2.53 (m, *J* =8.00 Hz, 1H, CH_2), 2.67 (dd, *J* =15.81, 4.27 Hz, 1H, CH_2), 3.00–4.00 (br s, H_2O+COOH), 3.09–3.20 (m, 1H, CH_2), 3.23 (s, 3H, CH_3), 3.37–3.49 (m, 2H, CH_2), 3.65–3.75 (m, 1H, CH_2), 4.40 (s, 1H, CH), 5.49 (d, *J* = 5.02 Hz, 1H, CH), 9.38 (s, 1H, NH) ppm; ^{13}C NMR (101 MHz, DMSO-d$_6$): δ = 38.9, 44.5, 53.1, 58.5, 70.4, 102.9, 120.2 (q, *J* = 273.7 Hz, CF_3), 127.6 (q, *J* = 35.4 Hz, C-4), 152.8, 172.0 ppm; ^{19}F NMR (376 MHz, DMSO-d$_6$): δ = −69.49 (s, CF_3) ppm. $C_{10}H_{13}F_3N_2O_4$ (282.22). Found, %: C, 42.53; H, 4.66; N, 9.94. Calculated, %: C, 42.56; H, 4.64; N, 9.93. LCMS: $[MH]^+$ 283.

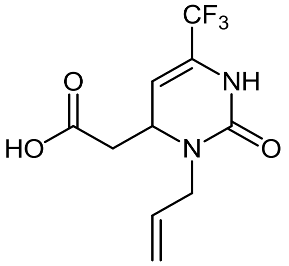

2-(3-Allyl-2-oxo-6-(trifluoromethyl)-1,2,3,4-tetrahydropyrimidin-4-yl)acetic acid (5f). White solid, yield: 0.65 g (82%), m.p. 105–107 °C. ^{1}H NMR (400 MHz, DMSO-d$_6$): δ = 2.32–2.47 (m, 1H, CH_2), 2.66 (dd, *J* = 15.39, 3.26 Hz, 1H, CH_2), 3.60 (dd, *J* = 15.86, 7.63 Hz, 1H, CH_2), 4.13–4.41 (m, 2H, CH_2, CH), 5.00–5.29 (m, 2H, CH_2), 5.50 (s, 1H, CH), 5.64–5.91 (m, 1H, CH), 9.41 (s, 1H, NH), 12.36 (br s, 1H, COOH) ppm; ^{13}C NMR (126 MHz, DMSO-d$_6$): δ = 38.8, 46.7, 51.8, 102.7, 117.4, 120.4 (q, *J* = 272.2 Hz, CF_3), 127.8 (q, *J* = 34.0 Hz, C-4), 134.2, 152.5, 171.9 ppm; ^{19}F NMR (376 MHz, DMSO-d$_6$): δ= −69.49 (s, CF_3) ppm. $C_{10}H_{11}F_3N_2O_3$ (264.20). Found, %: C, 45.43; H, 4.18; N, 10.63. Calculated, %: C, 45.46; H, 4.20; N, 10.60. LCMS: $[MH]^+$ 265.

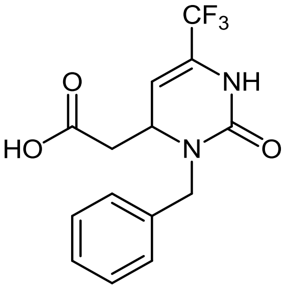

2-(3-Benzyl-2-oxo-6-(trifluoromethyl)-1,2,3,4-tetrahydropyrimidin-4-yl)acetic acid (5g). White solid, yield: 0.84 g (89%), m.p. 135–137 °C. ^{1}H NMR (400 MHz, DMSO-d$_6$): δ = 2.45–2.55 (m, 1H, CH_2), 2.65 (d, *J* = 14.92 Hz, 1H, CH_2), 4.15 (d, *J* = 14.92 Hz, 1H, CH_2), 4.23 (br s, 1H, CH), 4.97 (d, *J* = 15.86 Hz, 1H, CH_2), 5.48 (s, 1H, CH), 7.27 (s, 3H, CH), 7.34 (s, 2H, CH), 9.54 (s, 1H, NH), 12.56 (br s, 1H, COOH) ppm; ^{13}C NMR (101 MHz, DMSO-d$_6$): δ = 38.5, 47.2, 51.9, 102.7, 120.2 (q, *J* = 273.7 Hz, CF_3), 127.7, 127.8 (q, *J* = 34.5, C-4), 127.9, 129.1, 138.1, 153.0, 171.9 ppm; ^{19}F NMR (376 MHz, DMSO-d$_6$): δ = −69.53 (s, CF_3) ppm. $C_{14}H_{13}F_3N_2O_3$ (314.26). Found, %: C, 53.48; H, 4.15; N, 8.94. Calculated, %: C, 53.51; H, 4.17; N, 8.91. LCMS: $[MH]^+$ 315.

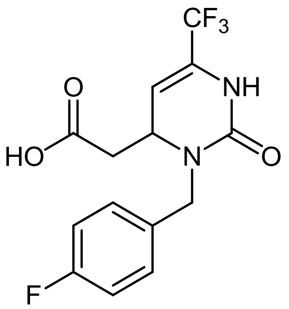

2-(3-(4-Fluorobenzyl)-2-oxo-6-(trifluoromethyl)-1,2,3,4-tetrahydropyrimidin-4-yl)acetic acid (5h). White solid, yield: 0.8 g (80%), m.p. 177–179 °C. ^{1}H NMR (400 MHz, DMSO-d$_6$): δ = 2.40–2.51 (m, 1H, CH$_2$), 2.65 (d, *J* = 14.82 Hz, 1H, CH$_2$), 4.16 (d, *J* = 14.92 Hz, 1H, CH$_2$), 4.24 (br s, 1H, CH), 4.91 (d, *J* = 14.92 Hz, 1H, CH$_2$), 5.47 (s, 1H, CH), 7.17 (t, *J* = 8.86 Hz, 2H, CH), 7.25–7.38 (m, 2H, CH), 9.57 (br s, 1H, NH), 12.47 (br s, 1H, COOH) ppm; ^{13}C NMR (126 MHz, DMSO-d$_6$): δ = 38.6, 46.6, 52.0, 102.7, 115.8 (d, *J* = 20.9 Hz), 120.2 (q, *J* = 272.2 Hz, CF$_3$), 127.7 (q, *J* = 35.3 Hz, C-4), 129.9 (d, *J* = 8.0 Hz), 134.3, 152.9, 161.9 (d, *J* = 242.8 Hz), 171.8 ppm; ^{19}F NMR (376 MHz, DMSO-d$_6$): δ= −69.45 (s, CF$_3$), −115.96 (s, CF) ppm. $C_{14}H_{12}F_4N_2O_3$ (332.25). Found, %: C, 50.76; H, 3.65; N, 8.42. Calculated, %: C, 50.61; H, 3.64; N, 8.43. LCMS: $[MH]^+$ 333.

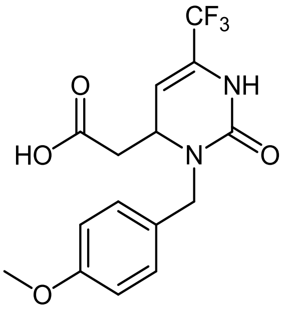

2-(3-(4-Methoxybenzyl)-2-oxo-6-(trifluoromethyl)-1,2,3,4-tetrahydropyrimidin-4-yl)acetic acid (5i). White solid, yield: 0.85 g (82%), m.p. 128–130 °C.^{1}H NMR (400 MHz, DMSO-d$_6$): δ = 2.45–2.55 (m, 1H, CH$_2$), 2.66 (dd, *J* = 14.03, 3.73 Hz, 1H, CH$_2$), 3.74 (s, 3H, CH$_3$), 4.06 (d, *J* = 15.37 Hz, 1H, CH$_2$), 4.21 (s, 1H, CH), 4.93 (d, *J* = 14.82 Hz, 1H, CH$_2$), 5.46 (s, 1H, CH), 6.92 (d, *J* = 8.23 Hz, 2H, CH), 7.22 (d, *J* = 7.68 Hz, 2H, CH), 9.52 (s, 1H, NH), 12.46 (br s, 1H, COOH) ppm; ^{13}C NMR (126 MHz, DMSO-d$_6$): δ = 38.5, 46.5, 51.4, 55.5, 102.6, 114.5, 120.2 (q, *J* = 273.4 Hz, CF$_3$), 127.8 (q, *J* = 35.3 Hz, CF$_3$), 129.4, 129.8, 152.9, 159.1, 171.9 ppm; ^{19}F NMR (376 MHz, DMSO-d$_6$): δ = −69.44 (s, CF$_3$) ppm. $C_{15}H_{15}F_3N_2O_4$ (344.29). Found, %: C, 52.32; H, 4.37; N, 8.10. Calculated, %: C, 52.33; H, 4.39; N, 8.14. LCMS: $[MH]^+$ 345.

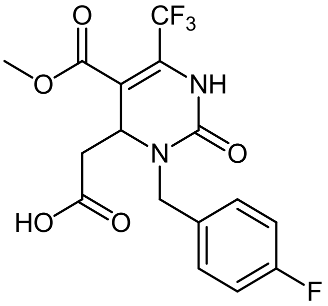

2-(3-(4-Fluorobenzyl)-5-(methoxycarbonyl)-2-oxo-6-(trifluoromethyl)-1,2,3,4-tetrahydropyrimidin-4-yl)acetic acid (5j). White solid, yield: 0.88 g (75%), m.p. 158–160 °C.^{1}H NMR (400 MHz, DMSO-d_6): δ = 2.47–2.63 (m, 2H, CH_2), 3.63 (s, 3H, CH_3), 4.21 (d, *J* = 15.86 Hz, 1H, CH_2), 4.47–4.62 (m, 1H, CH), 4.92 (d, *J* = 14.92 Hz, 1H, CH_2), 7.18 (t, *J* = 8.86 Hz, 2H, CH), 7.28–7.43 (m, 2H, CH), 10.06 (s, 1H, NH), 12.53 (br s, 1H, COOH) ppm; ^{13}C NMR (126 MHz, DMSO-d_6): δ = 37.8, 47.5, 52.7, 53.3, 106.6, 115.8 (d, *J* = 21.4 Hz), 119.7 (q, *J* = 275.3 Hz, CF_3), 130.0 (d, *J* = 8.0 Hz), 133.0 (q, *J* = 35.9 Hz, C-4), 133.8, 152.2, 162.0 (d, *J* = 243.3 Hz), 163.8, 171.7 ppm; ^{19}F NMR (376 MHz, DMSO-d_6): δ = −63.51 (s, CF_3) ppm, −115.70 (s, CF). $C_{16}H_{14}F_4N_2O_5$ (390.29). Found, %: C, 49.26; H, 3.62; N, 7.16. Calculated, %: C, 49.24; H, 3.62; N, 7.18. LCMS: $[MH]^+$ 391.

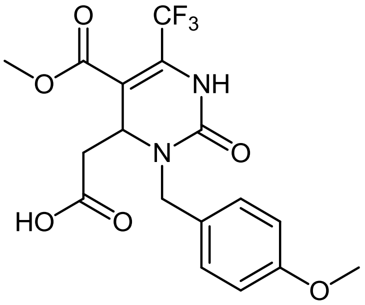

2-(3-(4-Methoxybenzyl)-5-(methoxycarbonyl)-2-oxo-6-(trifluoromethyl)-1,2,3,4-tetrahydropyrimidin-4-yl)acetic acid (5k). White solid, yield: 1 g (83%), m.p. 128–130 °C.^{1}H NMR (400 MHz, DMSO-d_6): δ = 2.45–2.60 (m, 2H, CH_2), 3.41 (br s, H_2O+COOH), 3.62 (s, 3H, CH_3), 3.73 (s, 3H, CH_3), 4.10 (d, *J* = 14.92 Hz, 1H, CH_2), 4.45–4.58 (m, 1H, CH), 4.90 (d, *J* = 14.92 Hz, 1H, CH_2), 6.91 (d, *J* = 8.39 Hz, 2H, CH), 7.19 (d, *J* = 8.39 Hz, 2H, CH), 10.02 (br s, 1H, NH) ppm; ^{13}C NMR (126 MHz, DMSO-d_6): δ = 37.8, 47.4, 52.7, 52.9, 55.5, 106.5, 114.5, 119.7 (q, J = 275.8 Hz, CF_3), 129.3, 129.4, 132.9 (q, *J* = 35.9 Hz, C-4), 152.1, 159.1, 163.8, 171.8 ppm; ^{19}F NMR (376 MHz, DMSO-d_6): δ = −63.52 (s, CF_3) ppm. $C_{17}H_{17}F_3N_2O_6$ (402.32). Found, %: C, 50.78; H, 4.28; N, 6.87. Calculated, %: C, 50.75; H, 4.26; N, 6.96. LCMS: $[MH]^+$ 403.

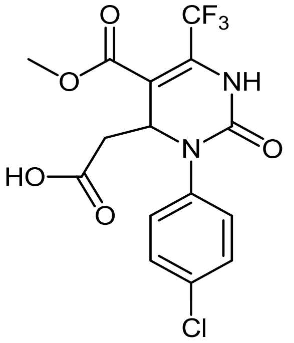

2-(3-(4-Chlorophenyl)-5-(methoxycarbonyl)-2-oxo-6-(trifluoromethyl)-1,2,3,4-tetrahydropyrimidin-4-yl)acetic acid (5l). White solid, yield: 0.95 g (81%), m.p. 177–179 °C. ^{1}H NMR (400 MHz, DMSO-d$_6$): δ = 2.45–2.56 (m, 1H, CH_2) 2.58–2.69 (m, 1H, CH_2), 3.70 (s, 3H, CH_3), 5.00 (s, 1H, CH), 7.47 (s, 4H, CH), 10.23 (br s, 1H, NH), 12.47 (br s, 1H, COOH) ppm; ^{13}C NMR (101 MHz, DMSO-d$_6$): δ = 38.0, 52.8, 57.6, 107.2, 119.7 (q, *J* = 276.7 Hz, CF_3), 129.4, 129.7, 131.7, 133.3 (q, *J* = 36.4 Hz, C-4), 139.3, 150.8, 163.6, 171.4 ppm; ^{19}F NMR (376 MHz, DMSO-d$_6$): δ = −63.51 (s, CF_3) ppm. $C_{15}H_{12}ClF_3N_2O_5$ (392.71). Found, %: C, 45.88; H, 3.11; N, 7.10. Calculated, %: C, 45.88; H, 3.08; N, 7.13. LCMS: $[MH]^+$ 393.

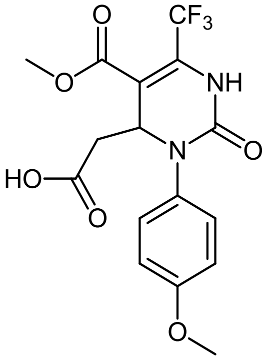

2-(5-(Methoxycarbonyl)-3-(4-methoxyphenyl)-2-oxo-6-(trifluoromethyl)-1,2,3,4-tetrahydropyrimidin-4-yl)acetic acid (5m). White solid, yield: 0.94 g (81%), m.p. 195–197 °C. ^{1}H NMR (400 MHz, DMSO-d$_6$): δ = 2.45–2.56 (m, 1H, CH_2), 2.58 (d, *J* = 4.66 Hz, 1H, CH_2), 3.70 (s, 3H, CH_3), 3.77 (s, 3H, CH_3), 4.81–4.95 (m, 1H, CH), 6.96 (d, *J* = 9.33 Hz, 2H, CH), 7.32 (d, *J* = 8.39 Hz, 2H, CH), 10.09 (br s, 1H, NH), 12.46 (br s, 1H, COOH) ppm; ^{13}C NMR (101 MHz, DMSO-d$_6$): δ = 37.9, 52.7, 55.7, 58.1, 106.8, 114.7, 119.8 (q, *J* = 276.4 Hz, CF_3), 129.4, 133.0, 133.5 (q, *J* = 36.4 Hz, C-4), 151.1, 158.5, 163.7, 171.5 ppm; ^{19}F NMR (376 MHz, DMSO-d$_6$): δ = −63.56 (s, CF_3) ppm. $C_{16}H_{15}F_3N_2O_6$ (388.30). Found, %: C, 49.49; H, 3.82; N, 7.24. Calculated, %: C, 49.49; H, 3.89; N, 7.21. LCMS: $[MH]^+$ 389.

2.3. General procedure for the synthesis of compounds 6a,f–m.

To the solution of compound **2a,f–m** (3 mmol) and compound **1a** (3.24 g, 18 mmol) in toluene (20 mL) triethylamine (0.3 g, 3 mmol) was added. The mixture was stirred at 80 °C for 2–4 h. The reaction was monitored by ^{19}F NMR spectroscopy. After completion of the

reaction the mixture was cooled and washed with 0.4 M hydrochloric acid (2 × 20 mL) then brine (2 × 20 mL) and 0.2 M potassium carbonate solution (2 × 20 mL). The organic layer was separated, dried over anhydrous sodium sulfate and evaporated. The obtained residue was crystallized from hexane/toluene 1:1.

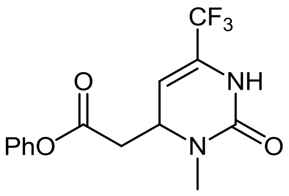

Phenyl 2-(3-methyl-2-oxo-6-(trifluoromethyl)-1,2,3,4-tetrahydropyrimidin-4-yl)acetate (6a). White solid, yield: 0.76 g (81%), m.p. 140–142 °C. ^{1}H NMR (400 MHz, DMSO-d$_6$): δ = 2.85 (dd, *J* = 14.92, 3.73 Hz, 1H, CH$_2$), 2.90 (s, 3H, CH$_3$), 3.00 (dd, *J* = 14.92, 6.53 Hz, 1H, CH$_2$), 4.56 (br s, 1H, CH), 5.57 (d, *J* = 4.66 Hz, 1H, CH), 7.09 (d, *J* = 7.46 Hz, 2H, CH), 7.27 (t, *J* = 7.46 Hz, 1H, CH), 7.42 (t, *J* = 7.46 Hz, 2H, CH), 9.48 (br s, 1H, NH) ppm; ^{13}C NMR (126 MHz, DMSO-d$_6$): δ = 32.8, 38.3, 55.0, 101.8, 120.4 (q, *J* = 272.3 Hz, CF$_3$), 122.2, 126.5, 128.1 (q, *J* = 36.9 Hz, C-4), 130.1, 150.8, 152.8, 169.2 ppm; ^{19}F NMR (376 MHz, DMSO-d$_6$): δ = −70.48 (s, CF$_3$) ppm. $C_{14}H_{13}F_3N_2O_3$ (314.26). Found, %: C, 53.35; H, 4.13; N, 8.94. Calculated, %: C, 53.51; H, 4.17; N, 8.91. LCMS: $[MH]^+$ 315.

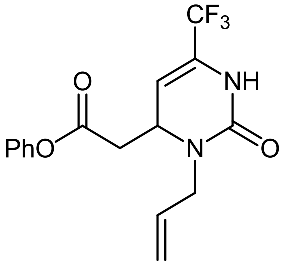

Phenyl 2-(3-allyl-2-oxo-6-(trifluoromethyl)-1,2,3,4-tetrahydropyrimidin-4-yl)acetate (6f). White solid, yield: 0.77 g (75%), m.p. 101–103 °C. ^{1}H NMR (400 MHz, DMSO-d$_6$): δ = 2.87 (dd, *J* = 15.86, 3.73 Hz, 1H, CH$_2$), 2.99 (dd, *J* = 14.92, 6.53 Hz, 1H, CH$_2$), 3.70 (dd, *J* = 15.86, 6.53 Hz, 1H, CH$_2$), 4.37 (dd, *J* = 15.86, 3.73 Hz, 1H, CH$_2$), 4.49 (br s, 1H, CH), 5.13–5.32 (m, 2H, CH$_2$), 5.60 (d, 1H, *J* = 4 Hz, CH), 5.78–5.89 (m, 1H, CH), 7.09 (d, *J* = 7.46 Hz, 2H, CH), 7.21–7.32 (m, 1H, CH), 7.35–7.52 (m, 2H, CH), 9.57 (s, 1H, NH) ppm; ^{13}C NMR (126 MHz, DMSO-d$_6$): δ = 38.5, 46.7, 52.3, 102.0, 117.6, 120.2 (q, *J* = 272.2 Hz, CF$_3$), 122.1, 126.4, 128.1 (q, *J* = 35.3 Hz, C-4), 130.0, 134.2, 150.7, 152.4, 169.0 ppm; ^{19}F NMR (376 MHz, DMSO-d$_6$): δ = −69.47 (s, CF$_3$) ppm. $C_{16}H_{15}F_3N_2O_3$ (340.30). Found, %: C, 56.63; H, 4.45; N, 8.23. Calculated, %: C, 56.47; H, 4.44; N, 8.23. LCMS: $[MH]^+$ 341.

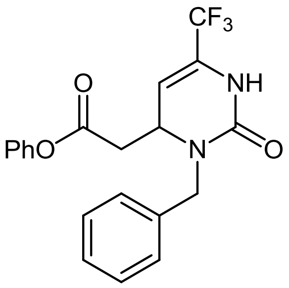

Phenyl 2-(3-benzyl-2-oxo-6-(trifluoromethyl)-1,2,3,4-tetrahydropyrimidin-4-yl)acetate (6g). White solid, yield: 0.82 g (70%), m.p. 130–132 °C. ^{1}H NMR (400 MHz, DMSO-d$_6$): δ = 2.85 (dd, *J* = 14.92, 2.80 Hz, 1 H), 3.03 (dd, *J* = 15.38, 6.99 Hz, 1H, CH$_2$), 4.25 (d, *J* = 15.85 Hz, 1H, CH$_2$), 4.41 (br s, 1H, CH), 5.04 (d, *J* = 15.85 Hz, 1H, CH$_2$), 5.58 (d, *J* = 3.73 Hz, 1 H), 7.08 (d, *J* = 8.39 Hz, 2H, CH), 7.19–7.47 (m, 8H, CH), 9.66 (br s, 1H, NH) ppm; ^{13}C NMR (126 MHz, DMSO-d$_6$): δ = 38.5, 47.3, 52.5, 102.2, 120.3 (q, *J* = 272.2 Hz, CF$_3$), 122.2, 126.5, 127.9, 128.0 (q, *J* = 35.4 Hz, C-4), 128.1, 129.2, 130.1, 138.1, 150.8, 153.0, 169.1 ppm; ^{19}F NMR (376 MHz, DMSO-d$_6$): δ = −69.15 (s, CF$_3$) ppm. $C_{20}H_{17}F_3N_2O_3$ (390.36). Found, %: C, 61.53; H, 4.41; N, 7.20. Calculated, %: C, 61.54; H, 4.39; N, 7.18. LCMS: $[MH]^+$ 391.

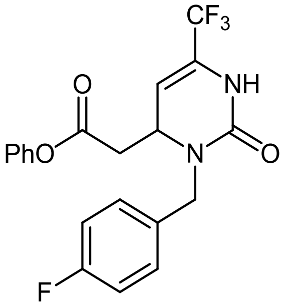

Phenyl 2-(3-(4-fluorobenzyl)-2-oxo-6-(trifluoromethyl)-1,2,3,4-tetrahydropyrimidin-4-yl)acetate (6h). White solid, yield: 0.91 g (74%), m.p. 133–135 °C. ^{1}H NMR (400 MHz, DMSO-d$_6$): δ = 2.84 (dd, *J* = 15.38, 3.26 Hz, 1H, CH$_2$), 3.04 (dd, *J* = 15.85, 6.53 Hz, 1H, CH$_2$), 4.25 (d, *J* = 15.85 Hz, 1H, CH$_2$), 4.42 (br s, 1H, CH), 4.97 (d, *J* = 14.92 Hz, 1H, CH$_2$), 5.57 (s, 1H, CH), 7.07 (d, *J* = 7.46 Hz, 2H, CH), 7.18 (t, *J* = 8.39 Hz, 2H, CH), 7.25 (t, *J* = 7.46 Hz, 1H, CH), 7.34–7.47 (m, 4H, CH), 9.66 (br s, 1H, NH) ppm; ^{13}C NMR (126 MHz, DMSO-d$_6$): δ = 38.4, 46.6, 52.4, 102.1, 115.8 (d, *J* = 20.9 Hz), 120.2 (q, *J* = 273.4 Hz, CF$_3$), 122.1, 126.4, 128.0 (q, *J* = 35.4 Hz, C-4), 129.9, 130.0 (d, *J* = 8.0 Hz), 134.3, 150.7, 152.9, 161.9 (d, *J* = 243.3 Hz), 169.0 ppm; ^{19}F NMR (376 MHz, DMSO-d$_6$): δ = −69.28 (s, CF$_3$), −115.71 (s, CF) ppm. $C_{20}H_{16}F_4N_2O_3$ (408.35). Found, %: C, 58.72; H, 3.83; N, 6.89. Calculated, %: C, 58.83; H, 3.95; N, 6.86. LCMS: $[MH]^+$ 409.

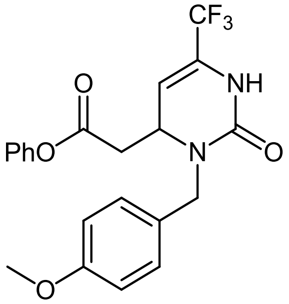

Phenyl 2-(3-(4-methoxybenzyl)-2-oxo-6-(trifluoromethyl)-1,2,3,4-tetrahydropyrimidin-4-yl)acetate (6i). White solid, yield: 0.87 g (69%), m.p. 115–117 °C. ^{1}H NMR (300 MHz, DMSO-d$_6$): δ = 2.85 (dd, *J* = 15.23, 2.59 Hz, 1H, CH_2), 3.03 (dd, *J* = 14.90, 6.48 Hz, 1H, CH_2), 3.75 (s, 3H, CH_3), 4.16 (d, *J* = 15.23 Hz, 1H, CH_2), 4.39 (br s, 1H, CH), 5.01 (d, *J* = 15.23 Hz, 1H, CH_2), 5.57 (d, *J* = 4.21 Hz, 1H, CH), 6.94 (d, *J* = 8.10 Hz, 2H, CH), 7.09 (d, *J* = 7.78 Hz, 2H, CH), 7.27 (d, *J* = 8.10 Hz, 3H, CH), 7.43 (t, *J* = 7.45 Hz, 2H, CH), 9.64 (br s, 1H, NH) ppm;^{13}C NMR (126 MHz, DMSO-d$_6$): δ = 38.2, 46.5, 51.9, 55.5, 102.0, 114.5, 120.2 (q, *J* = 272.2 Hz, CF_3), 122.1, 126.4, 128.0 (q, *J* = 35.4 Hz, C-4), 129.5, 129.7, 129.9, 150.7, 152.9, 159.1, 169.0 ppm; ^{19}F NMR (376 MHz, DMSO-d$_6$): δ = −69.37 (s, CF_3) ppm. $C_{21}H_{19}F_3N_2O_4$ (420.38). Found, %: C, 60.29; H, 4.63; N, 6.56. Calculated, %: C, 60.00; H, 4.56; N, 6.66. LCMS: $[MH]^+$ 421.

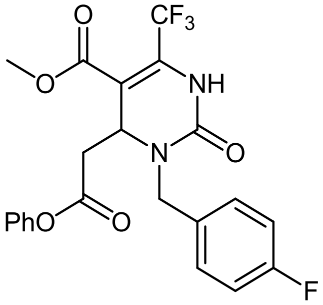

Methyl 3-(4-fluorobenzyl)-2-oxo-4-(2-oxo-2-phenoxyethyl)-6-(trifluoromethyl)-1,2,3,4-tetrahydropyrimidine-5-carboxylate (6j). White solid, yield: 1.12 g (80%), m.p. 122–125 °C. ^{1}H NMR (400 MHz, DMSO-d$_6$): δ = 2.87 (dd, *J* = 14.46, 3.26 Hz, 1H, CH_2), 3.04 (dd, *J* = 14.92, 5.60 Hz, 1H, CH_2), 3.67 (s, 3H, CH_3), 4.36 (d, *J* = 14.92 Hz, 1H, CH_2), 4.74 (s, 1H, CH), 4.95 (d, *J* = 14.92 Hz, 1H, CH_2), 7.09 (d, *J* = 7.46 Hz, 2H, CH), 7.19 (t, *J* = 8.39 Hz, 2H, CH), 7.27 (t, *J* = 7.46 Hz, 1H, CH), 7.34–7.48 (m, 4H, CH), 10.21 (br s, 1H, NH) ppm; ^{13}C NMR (126 MHz, DMSO-d$_6$): δ = 37.8, 47.6, 52.8, 53.7, 105.2, 115.9 (d, J=21.4 Hz), 119.6 (q, *J* = 275.9 Hz, CF_3), 122.0, 126.4, 130.0, 130.2 (d, *J* = 8.5 Hz), 133.8 (d, *J* = 3.0 Hz), 134.0 (q, *J* = 36.5 Hz, C-4), 150.7, 152.1, 162.4 (d, *J* = 243.8 Hz), 163.0, 168.8 ppm; ^{19}F NMR (376 MHz, DMSO-d$_6$): δ = −63.48 (s, CF_3), −115.59 (s, CF) ppm. $C_{22}H_{18}F_4N_2O_5$ (466.38). Found, %: C, 56.62; H, 3.88; N, 5.97. Calculated, %: C, 56.66; H, 3.89; N, 6.01. LCMS: $[MH]^+$ 467.

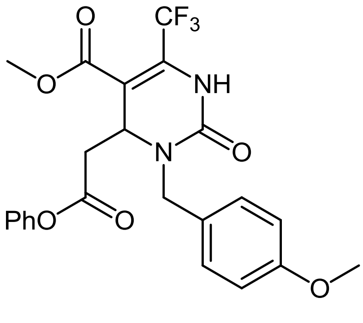

Methyl 3-(4-methoxybenzyl)-2-oxo-4-(2-oxo-2-phenoxyethyl)-6-(trifluoromethyl)-1,2,3,4-tetrahydropyrimidine-5-carboxylate (6k). White solid, yield: 1.02 g (71%), m.p. 125–127 °C. ^{1}H NMR (400 MHz, DMSO-d$_6$): δ = 2.85 (dd, *J* = 14.92, 3.73 Hz, 1H, CH_2), 3.03 (dd, *J* = 14.46, 6.06 Hz, 1H, CH_2), 3.66 (s, 3H, CH_3), 3.74 (s, 3H, CH_3), 4.27 (d, *J* =

14.92 Hz, 1H, CH_2), 4.69 (s, 1H, CH), 4.94 (d, J = 14.92 Hz, 1H, CH_2), 6.94 (d, J = 8.39 Hz, 2H, CH), 7.09 (d, J = 7.46 Hz, 2H, CH), 7.23–7.32 (m, 3H, CH), 7.43 (t, J = 8.39 Hz, 2H, CH), 10.21 (s, 1H, NH) ppm; ^{13}C NMR (101 MHz, DMSO-d_6): δ = 37.8, 47.5, 52.9, 53.3, 55.5, 105.0, 114.6, 119.7 (q, J = 276.7 Hz, CF_3), 122.0, 126.5, 129.3, 129.6, 130.0, 134.1 (q, J = 37.4 Hz, C-4), 150.7, 152.0, 159.2, 163.8, 168.8 ppm; ^{19}F NMR (376 MHz, DMSO-d_6): δ = −63.50 (s, CF_3) ppm. $C_{23}H_{21}F_3N_2O_6$ (478.42). Found, %: C, 57.88; H, 4.42; N, 5.84. Calculated, %: C, 57.74; H, 4.42; N, 5.86. LCMS: $[MH]^+$ 479.

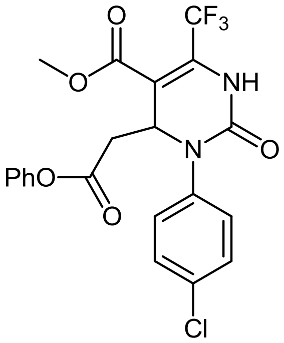

Methyl 3-(4-chlorophenyl)-2-oxo-4-(2-oxo-2-phenoxyethyl)-6-(trifluoromethyl)-1,2,3,4-tetrahydropyrimidine-5-carboxylate (6l). White solid, yield: 1.03 g (73%), m.p. 113–115 °C. ^{1}H NMR (400 MHz, DMSO-d_6): δ = 2.86–3.00 (m, 2H, CH_2), 3.74 (s, 3H, CH_3), 5.19 (br s, 1H, CH), 6.98 (d, J = 7.46 Hz, 2H, CH), 7.21–7.32 (m, 1H, CH), 7.35–7.45 (m, 2H, CH), 7.46–7.61 (m, 4H, CH), 10.41 (br s, 1H, NH) ppm; ^{13}C NMR (126 MHz, DMSO-d_6): δ = 38.2, 52.9, 57.8, 106.2, 119.6 (q, J = 277.3 Hz, CF_3), 121.9, 126.5, 129.6, 129.9, 130.0, 132.1, 133.9 (q, J = 35.3 Hz, C-4), 139.0, 150.6, 150.7, 163.5, 168.5 ppm; ^{19}F NMR (376 MHz, DMSO-d_6): δ = −63.52 (s, CF_3) ppm. $C_{21}H_{16}ClF_3N_2O_5$ (468.81). Found, %: C, 53.84; H, 3.47; N, 6.12. Calculated, %: C, 53.80; H, 3.44; N, 5.98. LCMS: $[MH]^+$ 469.

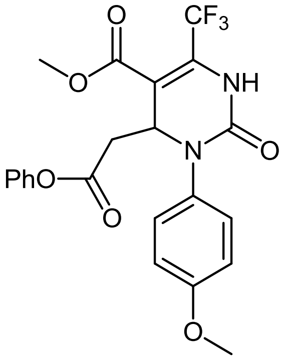

Methyl 3-(4-methoxyphenyl)-2-oxo-4-(2-oxo-2-phenoxyethyl)-6-(trifluoromethyl)-1,2,3,4-tetrahydropyrimidine-5-carboxylate (6m). White solid, yield: 1.04 g (75%), m.p. 109–111 °C. ^{1}H NMR (400 MHz, DMSO-d_6): δ = 2.84–2.95 (m, 2H, CH_2), 3.73 (s, 3H, CH_3), 3.78 (s, 3H, CH_3), 5.08 (s, 1H, CH), 6.88–7.08 (m, 4H, CH), 7.26 (t, J = 7.46 Hz, 1H, CH), 7.34–7.47 (m, 4H, CH), 10.25 (s, 1H, NH) ppm; ^{13}C NMR (101 MHz, DMSO-d_6): δ = 38.2, 52.9, 55.8, 58.3, 105.7, 114.8, 119.7 (q, J = 276.6 Hz, CF_3), 122.0, 126.5, 129.6, 129.9, 132.7, 134.2 (q, J = 36.4 Hz, C-4), 150.6, 151.0, 158.7, 163.7, 168.6 ppm; ^{19}F NMR (376 MHz, DMSO-d_6): δ = −63.53 (s, CF_3) ppm. $C_{22}H_{19}F_3N_2O_6$ (464.39). Found, %: C, 56.84; H, 4.14; N, 5.96. Calculated, %: C, 56.90; H, 4.12; N, 6.03. LCMS: $[MH]^+$ 465.

2.4. General procedure for the cleavage of the *N*1(3)-4-methoxybenzyl substituent. Synthesis of compounds 4j,5n,5o,6n,6o.

Compound **4–6i,6k** (2 mmol) was dissolved in trifluoroacetic acid (10 ml). The solution was stirred at 80 °C for 20 minutes (2 h for compound **4i**). Trifluoroacetic acid was evaporated and the residue was treated with dichloromethane (10 mL). The white precipitate formed was collected by filtration and recrystallized from toluene/ hexane 2:1.

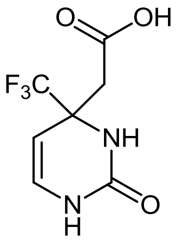

2-(2-Oxo-4-(trifluoromethyl)-1,2,3,4-tetrahydropyrimidin-4-yl)acetic acid (4j). White solid, yield: 0.34 g (75%), m.p. 205–207 °C. ^{1}H NMR (400 MHz, DMSO-d$_6$): δ = 2.56–2.60 (m, 1H, CH_2), 2.66 (d, *J* = 14.92 Hz, 1H, CH_2), 4.60 (d, *J* = 7.46 Hz, 1H, CH), 6.31 (s, 1H, CH), 7.46 (s, 1H, NH), 8.55 (s, 1H, NH), 12.39 (br s, 1H, COOH) ppm; ^{13}C NMR (126 MHz, DMSO-d$_6$): δ = 39.3, 60.7 (q, *J* = 29.0 Hz, C-4), 93.1, 125.6 (q, *J* = 287.3 Hz, CF_3), 129.7, 152.2, 169.9 ppm; ^{19}F NMR (376 MHz, DMSO-d$_6$): δ = −82.37 (s, CF_3) ppm. $C_7H_7F_3N_2O_3$ (224.14). Found, %: C, 37.46; H, 3.15; N, 12.51. Calculated, %: C, 37.51; H, 3.15; N, 12.50. LCMS: $[MH]^+$ 225.

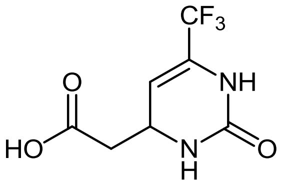

2-(2-Oxo-6-(trifluoromethyl)-1,2,3,4-tetrahydropyrimidin-4-yl)acetic acid (5n). White solid, yield: 0.35 g (78%), m.p. 200–202 °C. ^{1}H NMR (400 MHz, DMSO-d$_6$): δ = 2.40-2.55 (m, 2H, CH_2), 4.35 (s, 1H, CH), 5.39 (s, 1H, CH), 6.85 (s, 1H, NH), 9.20 (s, 1H, NH), 12.43 (br s, 1H, COOH) ppm; ^{13}C NMR (101 MHz, DMSO-d$_6$): δ = 42.0, 48.2, 102.6, 120.4 (q, *J* = 272.7 Hz, CF_3), 127.6 (q, *J* = 35.4 Hz, C-4), 152.9, 171.9 ppm; ^{19}F NMR (376 MHz, DMSO-d$_6$): δ = −69.73 (s, CF_3) ppm. $C_7H_7F_3N_2O_3$ (224.14). Found, %: C, 37.54; H, 3.07; N, 12.37. Calculated, %: C, 37.51; H, 3.15; N, 12.50. LCMS: $[MH]^+$ 225.

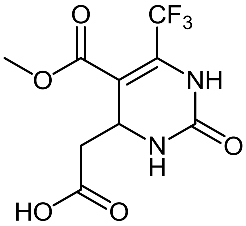

2-(5-(Methoxycarbonyl)-2-oxo-6-(trifluoromethyl)-1,2,3,4-tetrahydropyrimidin-4-yl)acetic acid (5o). White solid, yield: 0.4 g (70%), m.p. 209–211 °C. ^{1}H NMR (400 MHz, DMSO-d_6): δ = 2.40-2.55 (m, 2H, CH_2), 3.67 (s, 3H, CH_3), 4.46 (s, 1H, CH), 7.55 (s, 1H, NH), 9.68 (s, 1H, NH), 12.38 (br s, 1H, COOH) ppm; ^{13}C NMR (101 MHz, DMSO-d_6): δ = 40.8, 49.1, 52.7, 106.5, 119.8, (q, *J* = 277.8 Hz, CF_3), 133.0 (q, *J* = 35.4 Hz, C-4), 152.2, 164.3, 171.5 ppm; ^{19}F NMR (376 MHz, DMSO-d_6): δ = −63.96 (s, CF_3) ppm. $C_9H_9F_3N_2O_5$ (282.17). Found, %: C, 38.09; H, 3.23; N, 9.89. Calculated, %: C, 38.31; H, 3.21; N, 9.93. LCMS: $[MH]^+$ 283.

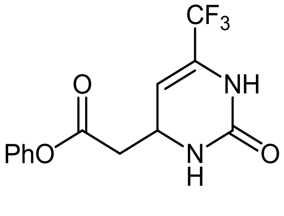

Phenyl 2-(2-oxo-6-(trifluoromethyl)-1,2,3,4-tetrahydropyrimidin-4-yl)acetate (6n). White solid, yield: 0.43 g (72%), m.p. 190–192 °C. ^{1}H NMR (400 MHz, DMSO-d_6): δ = 2.77–2.92 (m, 2H, CH_2), 4.53 (br s, 1H, CH), 5.50 (s, 1H, CH), 7.09–7.18 (m, 3H, CH), 7.20–7.32 (m, 1H, NH), 7.37–7.47 (m, 2H, CH), 9.29 (br s, 1H, NH) ppm; ^{13}C NMR (126 MHz, DMSO-d_6): δ = 42.0, 48.4, 102.1, 120.4 (q, *J* = 273.4 Hz, CF_3), 122.2, 126.4, 127.9 (q, *J* = 34.9 Hz, C-4), 129.9, 150.8, 152.8, 169.0 ppm; ^{19}F NMR (376 MHz, DMSO-d_6): δ = −69.59 (s, CF_3) ppm. $C_{13}H_{11}F_3N_2O_3$ (300.23). Found, %: C, 52.17; H, 3.66; N, 9.33. Calculated, %: C, 52.01; H, 3.69; N, 9.33. LCMS: $[MH]^+$ 301.

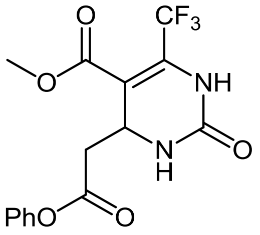

Methyl 2-oxo-4-(2-oxo-2-phenoxyethyl)-6-(trifluoromethyl)-1,2,3,4-tetrahydropyrimidine-5-carboxylate (6o). White solid, yield: 0.49 g (68%), m.p. 145–147 °C. ^{1}H NMR (400 MHz, DMSO-d_6): δ = 2.80–2.93 (m, 2H, CH_2), 3.72 (s, 3H, CH_3), 4.66 (s, 1H, CH), 7.16 (d, *J* = 7.68 Hz, 2H, CH), 7.28 (t, *J* = 7.68 Hz, 1H, CH), 7.43 (t, *J* = 7.68 Hz, 2H, CH), 7.85 (br s, 1H, NH), 9.86 (br s, 1H, NH) ppm; ^{13}C NMR (126 MHz, DMSO-d_6): δ = 41.2, 49.4, 52.8, 105.6, 119.8 (q, *J* = 277.2 Hz, CF_3), 122.2, 126.4, 129.9, 133.8 (q, *J* = 35.3 Hz, C-4), 150.8, 152.1, 164.2, 168.7 ppm; ^{19}F NMR (376 MHz, DMSO-d_6): δ = −63.88 (s, CF_3) ppm. $C_{15}H_{13}F_3N_2O_5$ (358.27). Found, %: C, 50.22; H, 3.74; N, 7.71. Calculated, %: C, 50.29; H, 3.66; N, 7.82. LCMS: $[MH]^+$ 359.

2.5. Procedure for the synthesis of compound 8a.

To the solution of compound **2a** (0.53 g, 3 mmol) and **1b** (2.04 g, 9 mmol) in dichloromethane (10 mL) triethylamine (0.3 g, 3 mmol) was added. The solution was

stirred at 40 °C for 3 hours. The white precipitate formed after reaction mixture cooling was collected by filtration and washed with dichloromethane (2 × 10 mL).

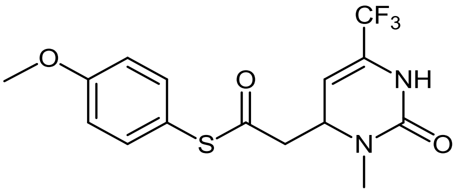

***S*-(4-methoxyphenyl) 2-(3-methyl-2-oxo-6-(trifluoromethyl)-1,2,3,4-tetrahydropyrimidin-4-yl)ethanethioate (8a).** White solid, yield: 0.9 g (83%), m.p. 150–152 °C. ^{1}H NMR (500 MHz, DMSO-d$_6$): δ = 2.84 (s, 3H, CH_3), 2.96 (dd, *J* = 14.82, 3.84 Hz, 1H, CH_2), 3.04 (dd, *J* = 14.82, 6.04 Hz, 1H, CH_2), 3.79 (s, 3H, CH_3), 4.47 (br s, 1H, CH), 5.49 (s, 1H, CH), 7.02 (d, *J* = 8.78 Hz, 2H, CH), 7.28 (d, *J* = 8.78 Hz, 2H, CH), 9.42 (br s, 1H) ppm; ^{13}C NMR (126 MHz, DMSO-d$_6$): δ = 33.1, 46.5, 55.4, 55.9, 101.8, 115.6, 118.4, 120.3 (q, *J* = 273.3 Hz, CF_3), 128.1 (q, *J* = 35.3 Hz, C-4), 136.5, 152.7, 161.0, 195.1 ppm; ^{19}F NMR (376 MHz, DMSO-d$_6$): δ = −69.39 (s, CF_3) ppm. $C_{15}H_{15}F_3N_2O_3S$ (360.35). Found, %: C, 49.98; H, 4.20; N, 7.86. Calculated, %: C, 50.00; H, 4.20; N, 7.77. LCMS: $[MH]^+$ 361.

2.6. General procedure for the synthesis of compounds 8b–m.

To the solution of compound **2b–m** (3 mmol) and **1b** (2.04 g, 9 mmol) in dichloromethane (20 mL) triethylamine (0.3 g, 3 mmol) was added. The solution was stirred at 40 °C for 1-3 hours. The reaction was monitored by ^{19}F NMR spectroscopy. After completion of the reaction the mixture was cooled and washed with 0.4 M hydrochloric acid (20 mL) and brine (20 mL). The organic layer was separated, dried over anhydrous sodium sulfate and evaporated. The obtained residue was crystallized from hexane/methyl *tert*-butyl ether 2:1 or purified by low pressure preparative chromatography.

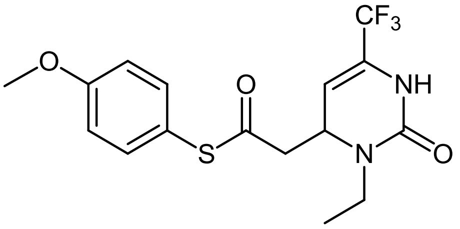

***S*-(4-Methoxyphenyl) 2-(3-ethyl-2-oxo-6-(trifluoromethyl)-1,2,3,4-tetrahydropyrimidin-4-yl)ethanethioate (8b).** Yellow solid, yield: 0.8 g (71%), m.p. 128–130 °C. ^{1}H NMR (500 MHz, DMSO-d$_6$): δ = 1.07 (t, *J* = 7.14 Hz, 3H, CH_3), 2.90–3.08 (m, 3H, CH_2), 3.57–3.71 (m, 1H, CH_2), 3.80 (s, 3H, CH_3), 4.51 (s, 1H, CH), 5.50 (s, 1H, CH), 7.03 (d, *J* = 8.78 Hz, 2H, CH), 7.29 (d, *J* = 8.78 Hz, 2H, CH), 9.38 (br s, 1H, NH) ppm; ^{13}C NMR (126 MHz, DMSO-d$_6$): δ = 13.3, 39.9, 47.1, 52.7, 55.8, 102.0, 115.5, 118.2, 120.2(q, *J* = 272.8 Hz, CF_3), 128.0 (q, *J* = 33.9 Hz, C-4), 136.4, 152.3, 160.9, 195.1 ppm; ^{19}F NMR (376 MHz, DMSO-d$_6$): δ = −69.53 (s, CF_3) ppm. $C_{16}H_{17}F_3N_2O_3S$ (374.38). Found, %: C, 51.34; H, 4.47; N, 7.42. Calculated, %: C, 51.33; H, 4.58; N, 7.48. LCMS: $[MH]^+$ 375.

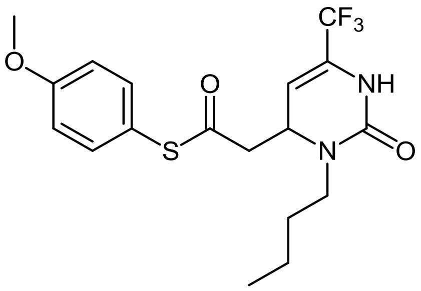

***S*-(4-Methoxyphenyl) 2-(3-butyl-2-oxo-6-(trifluoromethyl)-1,2,3,4-tetrahydropyrimidin-4-yl)ethanethioate (8c).** Yellow solid, yield: 0.93 g (77%), m.p. 95–98 °C. ^{1}H NMR (400 MHz, DMSO-d_6): δ = 0.87 (t, *J* = 7.28 Hz, 3H, CH_3), 1.15–1.31 (m, 2H, CH_2), 1.35–1.56 (m, 2H, CH_2), 2.81–3.04 (m, 3H, CH_2), 3.60–3.71 (m, 1H, CH_2), 3.78 (s, 3H, CH_3), 4.38–4.52 (m, 1H, CH), 5.50 (d, *J* = 5.02 Hz, 1H, CH), 7.01 (d, *J* = 9.03 Hz, 2H, CH), 7.27 (d, *J* = 9.03 Hz, 2H, CH), 9.40 (s, 1H, NH) ppm; ^{13}C NMR (101 MHz, DMSO-d_6): δ = 14.2, 19.9, 29.6, 44.4, 46.9, 52.9, 55.8, 102.0, 115.5, 118.2, 120.2 (q, *J* = 272.2 Hz, CF_3), 128.1 (q, *J* = 35.4 Hz, C-4), 136.4, 152.6, 160.9, 195.1 ppm; ^{19}F NMR (376 MHz, DMSO-d_6): δ = −69.48 (s, CF_3) ppm. $C_{18}H_{21}F_3N_2O_3S$ (402.43). Found, %: C, 53.66; H, 50.23; N, 6.99. Calculated, %: C, 53.72; H, 5.26; N, 6.96. LCMS: $[MH]^+$ 403.

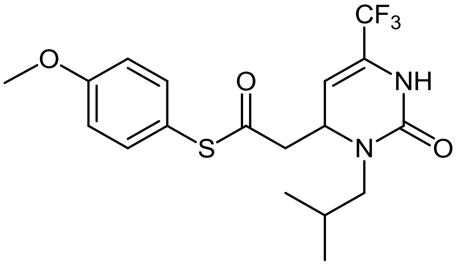

***S*-(4-Methoxyphenyl) 2-(3-isobutyl-2-oxo-6-(trifluoromethyl)-1,2,3,4-tetrahydropyrimidin-4-yl)ethanethioate (8d).** Yellow solid, yield: 0.93 g (77%), m.p. 120–122 °C.^{1}H NMR (400 MHz, DMSO-d_6): δ = 0.79 (d, *J* = 6.53 Hz, 3H, CH_3), 0.86 (d, *J* = 6.53 Hz, 3H, CH_3), 1.80–1.97 (m, 1H, CH), 2.62 (dd, *J* = 13.52, 6.99 Hz, 1H, CH_2), 2.92 (dd, *J* = 14.92, 4.66 Hz, 1H, CH_2), 3.00 (dd, *J* = 14.92, 6.53 Hz, 1H, CH_2), 3.61 (dd, *J* = 13.52, 7.93 Hz, 1H, CH_2), 3.79 (s, 3H, CH_3), 4.40 (d, *J* = 4.66 Hz, 1H, CH), 5.54 (d, *J* = 4.66 Hz, 1H, CH), 7.02 (d, *J* = 9.33 Hz, 2H, CH), 7.29 (d, *J* = 8.39 Hz, 2H, CH), 9.44 (s, 1H, NH) ppm; ^{13}C NMR (126 MHz, DMSO-d_6): δ = 20.1, 26.4, 46.5, 51.6, 53.2, 55.8, 79.6, 102.0, 115.5, 118.2, 119.8 (q, *J* = 273.4 Hz, CF_3), 128.4 (q, *J* = 35.3 Hz, C-4), 136.4, 152.9, 160.9, 195.1 ppm; ^{19}F NMR (376 MHz, DMSO-d_6): δ = −69.39 (s, CF_3) ppm. $C_{18}H_{21}F_3N_2O_3S$ (402.43). Found, %: C, 53.74; H, 5.26; N, 6.94.Calculated, %: C, 53.72; H, 5.26; N, 6.96. LCMS: $[MH]^+$ 403.

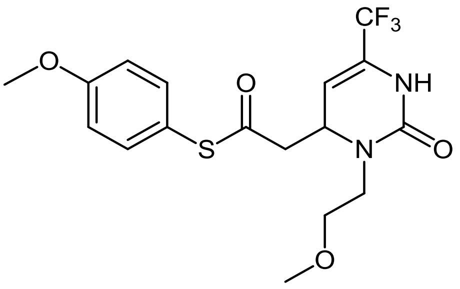

***S*-(4-Methoxyphenyl) 2-(3-(2-methoxyethyl)-2-oxo-6-(trifluoromethyl)-1,2,3,4-tetrahydropyrimidin-4-yl)ethanethioate (8e).** Yellow solid, yield: 0.91 g (75%), m.p. 95–97 °C.^{1}H NMR (400 MHz, DMSO-d_6): δ = 2.93–3.11 (m, 2H, CH_2), 3.13–3.22 (m, 1H, CH_2), 3.25 (s, 3H, CH_3), 3.40–3.51 (m, 2H, CH_2), 3.70–3.76 (m, 1H, CH_2), 3.79 (s, 3H, CH_3), 4.52 (s, 1H, CH), 5.51 (s, 1H, CH), 7.02 (d, *J* = 7.46 Hz, 2H, CH), 7.29 (d, *J* = 7.46 Hz, 2H, CH), 9.44 (s, 1H, NH) ppm; ^{13}C NMR (101 MHz, DMSO-d_6): δ = 44.7, 46.9, 54.0, 55.8, 58.6, 70.4, 102.2, 115.5, 118.2, 120.2 (q, *J* = 273.7 Hz, CF_3), 128.0 (q, *J* = 34.3 Hz, C-4), 136.4, 152.6, 160.9, 195.1 ppm; ^{19}F NMR (376 MHz, DMSO-d_6): δ = −69.48 (s, CF_3) ppm. $C_{17}H_{19}F_3N_2O_4S$ (404.40). Found, %: C, 50.48; H, 4.61; N, 6.98.Calculated, %: C, 50.49; H, 4.74; N, 6.93. LCMS: $[MH]^+$ 405.

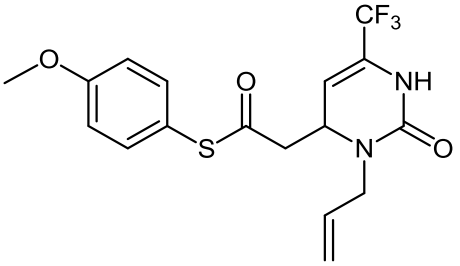

***S*-(4-Methoxyphenyl) 2-(3-allyl-2-oxo-6-(trifluoromethyl)-1,2,3,4-tetrahydropyrimidin-4-yl)ethanethioate (8f).** Yellow solid, yield: 0.85 g (73%), m.p. 112–114 °C.^{1}H NMR (400 MHz, DMSO-d_6): δ = 2.90–3.08 (m, 2H, CH_2), 3.60 (dd, *J* = 15.56, 6.53 Hz, 1H, CH_2), 3.78 (s, 3H, CH_3), 4.28–4.46 (m, 2H, CH_2, CH), 5.10–5.25 (m, 2H, CH_2), 5.52 (d, *J* = 4.52 Hz, 1H, CH), 5.69–5.84 (m, 1H, CH), 7.01 (d, *J* = 8.53 Hz, 2H, CH), 7.28 (d, *J* = 9.03 Hz, 2H, CH), 9.54 (s, 1H, NH) ppm; ^{13}C NMR (101 MHz, DMSO-d_6): δ = 46.6, 46.7, 52.5, 55.8, 101.9, 115.5, 117.6, 118.2, 120.2 (q, *J* = 273.5 Hz, CF_3), 128.1 (q, *J* =35.2 Hz, C-4), 134.0, 136.4, 152.4, 160.9, 195.1 ppm; ^{19}F NMR (376 MHz, DMSO-d_6): δ = −69.56 (s, CF_3) ppm. $C_{17}H_{17}F_3N_2O_3S$ (386.39). Found, %: C, 52.89; H, 4.43; N, 7.13. Calculated, %: C, 52.84; H, 4.43; N, 7.25. LCMS: $[MH]^+$ 387.

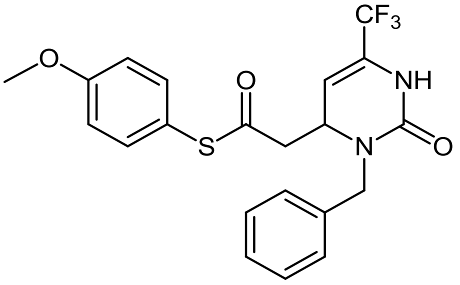

***S*-(4-Methoxyphenyl) 2-(3-benzyl-2-oxo-6-(trifluoromethyl)-1,2,3,4-tetrahydropyrimidin-4-yl)ethanethioate (8g).** Yellow solid, yield: 0.97 g (74%), m.p. 125–127 °C. ^{1}H NMR (400 MHz, DMSO-d$_6$): δ = 2.91–3.00 (m, 1H, CH$_2$), 3.08 (dd, *J* = 14.82, 5.49 Hz, 1H, CH$_2$), 3.80 (s, 3H, CH$_3$), 4.18 (d, *J* = 15.37 Hz, 1H, CH$_2$), 4.35 (s, 1H, CH), 5.03 (d, *J* = 15.37 Hz, 1H, CH$_2$), 5.50 (s, 1H, CH), 7.03 (d, *J* = 7.14 Hz, 2H, CH), 7.29 (d, *J* = 6.59 Hz, 5H, CH), 7.32–7.41 (m, 2H, CH), 9.59 (s, 1H, NH) ppm; ^{13}C NMR (101 MHz, DMSO-d$_6$): δ = 46.4, 47.3, 52.6, 55.8, 102.0, 115.5, 118.1, 120.2 (q, *J* = 272.2 Hz, CF$_3$), 127.8, 128.0, 128.1 (q, *J* = 35.2 Hz, C-4), 129.1, 136.5, 137.9, 152.9, 160.9, 195.1 ppm; ^{19}F NMR (376 MHz, DMSO-d$_6$): δ = −69.44 (s, CF$_3$) ppm. $C_{21}H_{19}F_3N_2O_3S$ (436.45). Found, %: C, 57.79; H, 4.39; N, 6.42. Calculated, %: C, 57.79; H, 4.39; N, 6.42. LCMS: [MH]$^+$ 437.

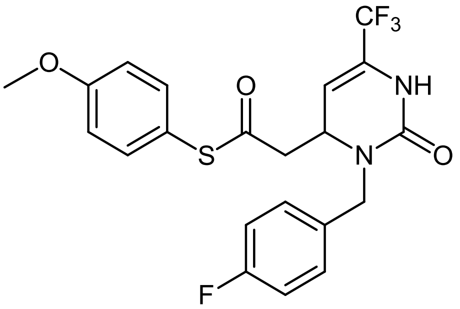

***S*-(4-Methoxyphenyl) 2-(3-(4-fluorobenzyl)-2-oxo-6-(trifluoromethyl)-1,2,3,4-tetrahydropyrimidin-4-yl)ethanethioate (8h).** Yellow solid, yield: 0.97 g (71%), m.p. 140–142 °C. ^{1}H NMR (400 MHz, DMSO-d$_6$): δ = 2.94 (d, *J* = 14.56 Hz, 1H, CH$_2$), 3.08 (dd, *J* = 14.56, 6.02 Hz, 1H, CH$_2$), 3.78 (s, 3H, CH$_3$), 4.17 (d, *J* = 15.06 Hz, 1H, CH$_2$), 4.34 (s, 1H, CH), 4.94 (d, *J* = 15.06 Hz, 1H, CH$_2$), 5.50 (s, 1H, CH), 7.01 (d, *J* = 8.03 Hz, 2H, CH), 7.10–7.22 (m, 2H, CH), 7.22–7.39 (m, 4H, CH), 9.62 (br s, 1H, NH) ppm; ^{13}C NMR (101 MHz, DMSO-d$_6$): δ = 46.4, 46.7, 52.7, 55.8, 102.0, 115.5, 115.8 (d, *J* = 22.0 Hz), 118.1, 120.6(q, *J* = 272.2 Hz, CF$_3$), 128.0 (q, *J* =35.2 Hz, C-4), 130.1 (d, *J* = 8.1 Hz), 134.2, 136.4, 152.8, 160.9, 162.0 (d, *J* = 243.4 Hz), 195.0 ppm; ^{19}F NMR (376 MHz, DMSO-d$_6$): δ = −69.48 (s, CF$_3$), −115.86 (s, CF) ppm. $C_{21}H_{18}F_4N_2O_3S$ (454.44). Found, %: C, 55.56; H, 4.09; N, 6.07. Calculated, %: C, 55.50; H, 3.99; N, 6.16. LCMS: [MH]$^+$ 455.

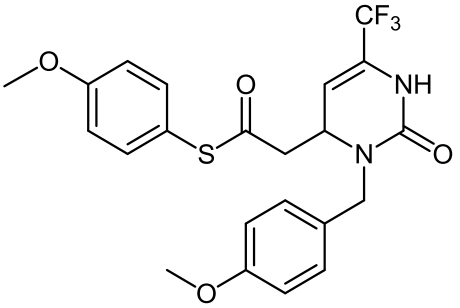

***S*-(4-Methoxyphenyl) 2-(3-(4-methoxybenzyl)-2-oxo-6-(trifluoromethyl)-1,2,3,4-tetrahydropyrimidin-4-yl)ethanethioate (8i).** Yellow solid, yield: 0.98 g (70%), m.p. 150–152 °C. ^{1}H NMR (400 MHz, DMSO-d$_6$): δ = 2.93 (d, *J* = 13.99 Hz, 1H, CH$_2$), 3.01–3.15 (m,

1H, CH_2), 3.73 (s, 3H, CH_3), 3.79 (s, 3H, CH_3), 4.05 (d, J = 14.92 Hz, 1H, CH_2), 4.30 (s, 1H, CH), 4.95 (d, J = 14.92 Hz, 1H, CH_2), 5.47 (s, 1H, CH), 6.91 (d, J = 6.53 Hz, 2H, CH), 7.02 (d, J = 7.46 Hz, 2H, CH), 7.21 (d, J = 7.46 Hz, 2H, CH), 7.28 (d, J = 7.46 Hz, 2H, CH), 9.57 (br s, 1H, NH) ppm; ^{13}C NMR (101 MHz, DMSO-d_6): δ = 46.4, 46.6, 52.2, 55.5, 55.8, 102.0, 114.5, 115.5, 118.2, 120.2 (q, J = 272.9 Hz, CF_3), 128.0 (q, J = 35.4 Hz, CF_3), 129.5, 129.6, 136.4, 152.8, 159.1, 160.9, 195.0 ppm; ^{19}F NMR (376 MHz, DMSO-d_6): δ = −69.45 (s, CF_3) ppm. $C_{22}H_{21}F_3N_2O_4S$ (466.47). Found, %: C, 56.67; H, 4.41; N, 5.92. Calculated, %: C, 56.65; H, 4.54; N, 6.01. LCMS: $[MH]^+$ 467.

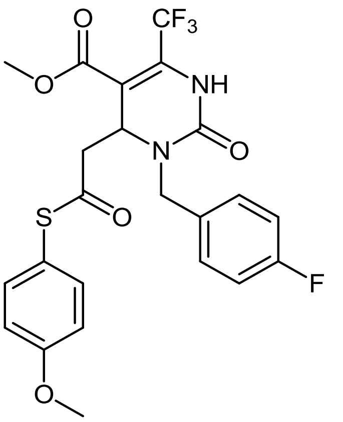

Methyl 3-(4-fluorobenzyl)-4-(2-((4-methoxyphenyl)thio)-2-oxoethyl)-2-oxo-6-(trifluoromethyl)-1,2,3,4-tetrahydropyrimidine-5-carboxylate (8j). White solid, yield: 1.18 g (77%), m.p. 145–146 °C. ^{1}H NMR (400 MHz, DMSO-d_6): δ = 2.95 (dd, J = 14.92, 4.66 Hz, 1H, CH_2), 3.08 (dd, J = 13.99, 5.60 Hz, 1H, CH_2), 3.66 (s, 3H, CH_3), 3.80 (s, 3H, CH_3), 4.26 (d, J = 14.92 Hz, 1H, CH_2), 4.64 (s, 1H, CH), 4.91 (d, J = 14.92 Hz, 1H, CH_2), 7.03 (d, J = 8.39 Hz, 2H, CH), 7.18 (t, J = 8.39 Hz, 2H, CH), 7.24–7.40 (m, 4H, CH), 10.17 (s, 1H, NH) ppm; ^{13}C NMR (101 MHz, DMSO-d_6): δ = 45.7, 47.6, 52.8, 54.0, 55.8, 104.7, 115.5, 116.0, 118.1, 119.1 (q, J = 273.3 Hz, CF_3), 130.1, 133.7, 134.4 (q, J = 35.2 Hz, C-4), 136.4, 152.0, 160.9, 162.1 (d, J = 242.0 Hz), 163.6, 194.7 ppm; ^{19}F NMR (376 MHz, DMSO-d_6): δ = −63.47 (s, CF_3), −115.56 (s, CF) ppm. $C_{23}H_{20}F_4N_2O_5S$ (512.47). Found, %: C, 53.73; H, 3.95; N, 5.44. Calculated, %: C, 53.90; H, 3.93; N, 5.47. LCMS: $[MH]^+$ 513.

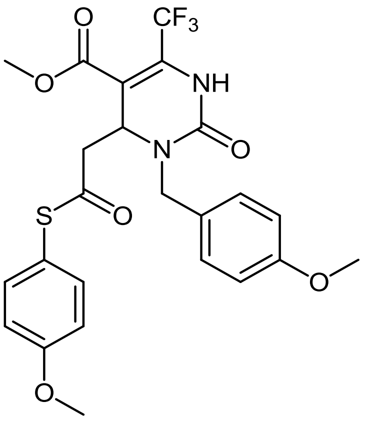

Methyl 3-(4-methoxybenzyl)-4-(2-((4-methoxyphenyl)thio)-2-oxoethyl)-2-oxo-6-(trifluoromethyl)-1,2,3,4-tetrahydropyrimidine-5-carboxylate (8k). White solid, yield: 1.18 g (75%), m.p. 145–157 °C. ^{1}H NMR (400 MHz, DMSO-d$_6$): δ = 2.93 (d, *J* = 12.12 Hz, 1H, CH_2), 3.07 (d, *J* = 10.26 Hz, 1H, CH_2), 3.65 (s, 3H, CH_3), 3.73 (s, 3H, CH_3), 3.80 (s, 3H, CH_3), 4.16 (d, *J* = 14.92 Hz, 1H, CH_2), 4.60 (s, 1H, CH), 4.91 (d, *J* = 14.92 Hz, 1H, CH_2), 6.92 (d, *J* = 7.46 Hz, 2H, CH), 7.03 (d, *J* = 7.46 Hz, 2H, CH), 7.14–7.37 (m, 4H, CH), 10.15 (s, 1H, NH) ppm; ^{13}C NMR (126 MHz, DMSO-d$_6$): δ = 45.6, 47.5, 52.8, 53.6, 55.5, 55.8, 104.6, 114.5, 115.5, 118.1, 119.6 (q, *J* = 278.3 Hz, CF_3), 129.1, 129.6, 134.5 (q, *J* = 35.3 Hz), 136.3, 151.9, 159.2, 160.9, 163.6, 194.7 ppm; ^{19}F NMR (376 MHz, DMSO-d$_6$): δ = −63.27 (s, CF_3) ppm. $C_{24}H_{23}F_3N_2O_6S$ (524.51), Found, %: C, 54.88; H, 4.40; N, 5.32. Calculated, %: C, 54.96; H, 4.42; N, 5.34. LCMS: $[MH]^+$ 525.

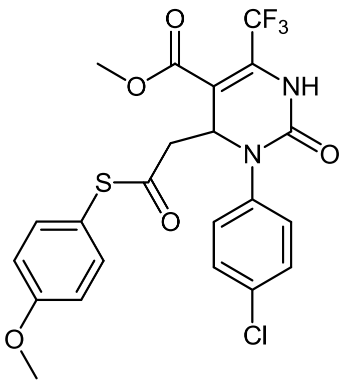

Methyl 3-(4-chlorophenyl)-4-(2-((4-methoxyphenyl)thio)-2-oxoethyl)-2-oxo-6-(trifluoromethyl)-1,2,3,4-tetrahydropyrimidine-5-carboxylate (8l). White solid, yield: 1.25 g (81%), m.p. 133–135 °C. ^{1}H NMR (400 MHz, DMSO-d$_6$): δ = 2.92 (dd, *J* = 13.99, 4.66 Hz, 1H, CH_2), 3.08 (dd, *J* = 14.46, 5.13 Hz, 1H, CH_2), 3.74 (s, 3H, CH_3), 3.80 (s, 3H, CH_3), 5.11 (br s, 1H, CH), 7.00 (d, *J* = 8.39 Hz, 2H, CH), 7.20 (d, *J* = 8.39 Hz, 2H, CH), 7.51 (q, *J* = 8.39 Hz, 4H, CH), 10.29 (br s, 1H, NH) ppm; ^{13}C NMR (101 MHz, DMSO-d$_6$): δ = 45.9, 52.9, 55.8, 58.1, 105.8, 115.5, 117.9, 119.6 (q, *J* = 276.6 Hz, CF_3), 129.4, 130.0, 131.9, 134.3 (q, *J* = 36.7 Hz, C-4), 136.3, 139.1, 150.6, 161.0, 163.4, 194.5 ppm; ^{19}F NMR (376 MHz, DMSO-d$_6$): δ = −63.51 (s, CF_3) ppm. $C_{22}H_{18}ClF_3N_2O_5S$ (514.90). Found, %: C, 51.45; H, 3.51; N, 5.47. Calculated, %: C, 51.32; H, 3.52; N, 5.44. LCMS: $[MH]^+$ 515.

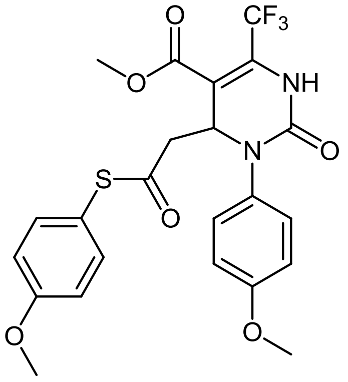

Methyl 3-(4-methoxyphenyl)-4-(2-((4-methoxyphenyl)thio)-2-oxoethyl)-2-oxo-6-(trifluoromethyl)-1,2,3,4-tetrahydropyrimidine-5-carboxylate (8m). White solid, yield: 1.1 g (72%), m.p. 138–140 °C.^{1}H NMR (400 MHz, DMSO-d_6): δ = 2.91 (dd, *J* = 13.99, 4.66 Hz, 1H, CH_2), 3.04 (dd, *J* = 13.99, 4.66 Hz, 1H, CH_2), 3.73 (s, 3H, CH_3), 3.78 (s, 3H, CH_3), 3.79 (s, 3H, CH_3), 4.99 (s, 1H, CH), 6.99 (t, *J* = 9.79 Hz, 4H, CH), 7.22 (d, *J* = 8.39 Hz, 2H, CH), 7.38 (d, *J* = 8.39 Hz, 2H, CH), 10.18 (s, 1H, NH) ppm; ^{13}C NMR (126 MHz, DMSO-d_6): δ = 45.8, 52.8, 55.81, 55.82, 58.5, 105.4, 114.7, 115.4, 118.0, 119.7 (q, *J* = 276.3 Hz, CF_3), 129.5, 132.8, 134.5 (q, *J* =36.9 Hz, C-4), 136.3, 150.8, 158.6, 160.9, 163.5, 194.4 ppm; ^{19}F NMR (376 MHz, DMSO-d_6): δ = −63.52 (s, CF_3) ppm. $C_{23}H_{21}F_3N_2O_6S$ (510.48). Found, %: C, 54.36; H, 4.20; N, 5.47. Calculated, %: C, 54.11; H, 4.15; N, 5.49. LCMS: $[MH]^+$ 511.

2.7. General procedure for the synthesis of compounds 9,10,11a–c.

Compound **4–6a,g,i** (2 mmol) was dissolved in methanol (25 mL) and 10% Pd/C as catalyst (20 wt %) was added. Hydrogen gas was bubbled at atmospheric pressure into the resulting suspension while stirring. The reaction was monitored by ^{19}F NMR spectroscopy. After 3 h the mixture was filtered and the solvent was evaporated. The resulting white solid residue was recrystallized from methanol/water 1:1.

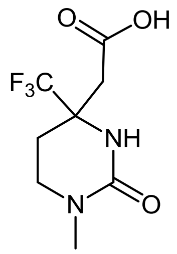

2-(1-Methyl-2-oxo-4-(trifluoromethyl)hexahydropyrimidin-4-yl)acetic acid (9a). White solid, yield: 0.45 g (95%), m.p.163–165 °C. ^{1}H NMR (400 MHz, DMSO-d_6): δ = 2.14 (d, *J* = 13.99 Hz, 1H, CH_2), 2.21–2.37 (m, 1H, CH_2), 2.58–2.70 (m, 2H, CH_2), 2.77 (s, 3H, CH_3), 3.13–3.26 (m, 2H, CH_2), 6.89 (s, 1H, NH) ppm; ^{13}C NMR (126 MHz, DMSO-d_6): δ = 25.1, 34.6, 39.0, 43.7, 57.7 (q, *J* = 27.9 Hz, C-4), 126.7 (q, *J* = 286.8 Hz, CF_3), 154.7, 170.4 ppm; ^{19}F NMR (376 MHz, DMSO-d_6): δ = −77.91 (s, CF_3) ppm. $C_8H_{11}F_3N_2O_3$ (240.18). Found, %: C, 40.19; H, 4.67; N, 11.73. Calculated, %: C, 40.01; H, 4.62; N, 11.66. LCMS: $[MH]^+$ 241.

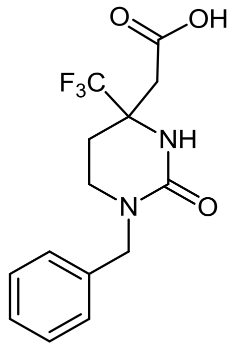

2-(1-Benzyl-2-oxo-4-(trifluoromethyl)hexahydropyrimidin-4-yl)acetic acid (9b). White solid, yield: 0.61 g (96%), m.p. 152–154 C.^{1}H NMR (400 MHz, DMSO-d_6): δ = 2.10–2.30 (d, *J* = 14.56 Hz, 1H, CH_2), 2.23–2.37 (m, 1H, CH_2), 2.68 (d, *J* = 4.02 Hz, 2H, CH_2), 3.07–3.19 (m, 2H, CH_2), 4.38 (d, *J* = 15.06 Hz, 1H, CH_2), 4.54 (d, *J* = 15.56 Hz, 1H, CH_2), 7.09 (s, 1H, NH), 7.16–7.27 (m, 3H, CH), 7.32 (t, *J* = 7.03 Hz, 2H, CH) ppm; ^{13}C NMR (101 MHz, DMSO-d_6): δ = 25.2, 38.9, 41.6, 50.0, 57.7 (q, *J* = 26.4 Hz, C-4), 126.7 (q, *J* = 286.1 Hz, CF_3), 127.4, 127.7, 128.9, 138.6, 154.7, 170.5 ppm; ^{19}F NMR (376 MHz, DMSO-d_6): δ = −77.76 (s, CF_3) ppm. $C_{14}H_{15}F_3N_2O_3$ (316.28). Found, %: C, 53.21; H, 4.73; N, 9.02. Calculated, %: C, 53.17; H, 4.78; N, 8.86. LCMS: $[MH]^+$ 317.

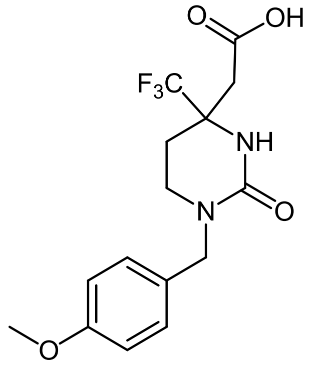

2-(1-(4-Methoxybenzyl)-2-oxo-4-(trifluoromethyl)hexahydropyrimidin-4-yl)acetic acid (9c). White solid, yield: 0.66 g (95%), m.p. 145–147 °C.^{1}H NMR (400 MHz, DMSO-d_6): δ = 2.08–2.18 (m, 1H, CH_2), 2.20–2.32 (m, 1H, CH_2), 2.61–2.73 (m, 2H, CH_2), 3.10 (s, 2H, CH_2), 3.73 (s, 3H, CH_3), 4.30 (d, *J* = 14.92 Hz, 1H, CH_2), 4.48 (d, *J* = 14.92 Hz, 1H, CH_2), 6.80–6.95 (m, 2H, CH), 7.03 (s, 1H, NH), 7.10–7.23 (m, 2H, CH), 12.64 (br s, 1H, COOH) ppm; ^{13}C NMR (126 MHz, DMSO-d_6): δ = 25.2, 38.9, 41.2, 49.4, 55.5, 57.6 (q, *J* = 26.9 Hz, C-4), 114.3, 126.6 (q, *J* = 287.7 Hz, CF_3), 129.2, 130.4, 154.6, 158.8, 170.4 ppm; ^{19}F NMR (376 MHz, DMSO-d_6): δ = −77.75 (s, CF_3) ppm. $C_{15}H_{17}F_3N_2O_4$ (346.30). Found, %: C, 52.02; H, 4.94; N, 8.06. Calculated, %: C, 52.02; H, 4.95; N, 8.09. LCMS: $[MH]^+$ 347.

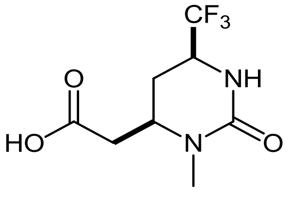

2-(3-Methyl-2-oxo-6-(trifluoromethyl)hexahydropyrimidin-4-yl)acetic acid (10a). White solid, yield: 0.45 g (93%), m.p. 173–175 °C. ^{1}H NMR (400 MHz, DMSO-d_6): δ = 1.75–1.93

(m, 1H, CH_2), 2.16–2.30 (m, 1H, CH_2), 2.40–2.60 (m, 1H, CH_2), 2.67–2.87 (m, 4H, CH_2, CH_3), 3.73 (br s, 1H, CH), 4.14 (br s, 1H, CH), 6.99 (s, 1H, NH), 12.47 (br s, 1H, COOH) ppm. ^{13}C NMR (101 MHz, DMSO-d_6): δ = 26.3, 31.6, 38.2, 50.6 (q, *J* = 31.3 Hz, C-4), 51.1, 125.3 (q, *J* = 280.2 Hz, CF_3), 156.0, 172.9 ppm; ^{19}F NMR (376 MHz, DMSO-d_6): δ = −76.52 (s, CF_3) ppm. $C_8H_{11}F_3N_2O_3$ (240.18). Found, %: C, 40.05; H, 4.59; N, 11.64. Calculated, %: C, 40.01; H, 4.62; N, 11.66. LCMS: $[MH]^+$ 241.

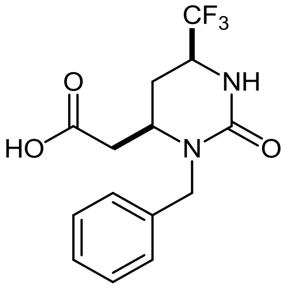

2-(3-Benzyl-2-oxo-6-(trifluoromethyl)hexahydropyrimidin-4-yl)acetic acid (10b). White solid, yield: 0.6 g (95%), m.p. 168–170 °C. ^{1}H NMR (500 MHz, DMSO-d_6): δ = 1.87–2.01 (m, 1H, CH_2), 2.16–2.29 (m, 1H, CH_2), 2.37 (dd, *J* = 16.19, 9.06 Hz, 1H, CH_2), 2.71 (dd, *J* = 15.92, 4.39 Hz, 1H, CH_2), 3.66–3.79 (m, 1H, CH), 4.16–4.31 (m, 2H, CH, CH_2), 4.88 (d, *J* = 15.92 Hz, 1H, CH_2), 7.16–7.28 (m, 4H, CH, NH), 7.34 (t, *J* = 7.68 Hz, 2H, CH), 12.45 (br s, 1H, COOH) ppm; ^{13}C NMR (126 MHz, DMSO-d_6): δ = 25.9, 37.8, 46.9, 49.4, 50.6 (q, *J* = 30.9 Hz, C-4), 125.4 (q, *J* = 281.7 Hz, CF_3), 127.3, 127.4, 128.9, 139.1, 156.0, 172.5 ppm; ^{19}F NMR (188 MHz, DMSO-d_6): δ = −76.06 (s, CF_3) ppm. $C_{14}H_{15}F_3N_2O_3$ (316.28). Found, %: C, 53.22; H, 4.76; N, 8.84. Calculated, %: C, 53.17; H, 4.78; N, 8.86. LCMS: $[MH]^+$ 317.

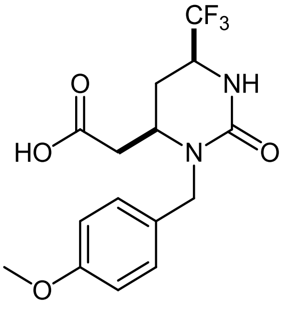

2-(3-(4-Methoxybenzyl)-2-oxo-6-(trifluoromethyl)hexahydropyrimidin-4-yl)acetic acid (10c). White solid, yield: 0.66 g (95%), m.p. 153–155 °C. ^{1}H NMR (400 MHz, DMSO-d_6): δ = 1.80–2.00 (m, 1H, CH_2), 2.10–2.25 (m, 1H, CH_2), 2.27–2.45 (m, 1H, CH_2), 2.60-2.80 (br s, 2H, CH_2), 3.60–3.83 (m, 4H, CH_3, CH), 4.05–4.23 (m, 2H, CH, CH_2), 4.83 (d, *J* = 14.90 Hz, 1H, CH_2), 6.89 (s, 2H, CH), 7.17 (s, 3H, CH, NH), 12.45 (br s, 1H, COOH) ppm; ^{13}C NMR (126 MHz, DMSO-d_6): δ = 25.8, 37.7, 46.2, 49.0, 50.6 (q, *J* = 30.4 Hz, C-4), 55.5, 114.3, 125.4 (q, *J* = 281.2 Hz, CF_3), 128.9, 130.9, 156.0, 158.7, 172.6 ppm; ^{19}F NMR (376 MHz, DMSO-d_6): δ = −76.04 (s, CF_3) ppm. $C_{15}H_{17}F_3N_2O_4$ (346.30). Found, %: C, 52.12; H, 4.84; N, 8.11. Calculated, %: C, 52.02; H, 4.95; N, 8.09. LCMS: $[MH]^+$ 347.

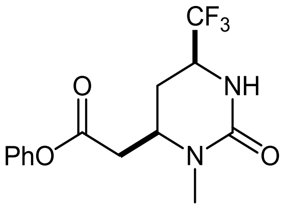

Phenyl 2-(3-methyl-2-oxo-6-(trifluoromethyl)hexahydropyrimidin-4-yl)acetate (11a). White solid, yield: 0.59 g (94%), m.p. 157–159 °C. ^{1}H NMR (400 MHz, DMSO-d$_6$): δ = 1.90–2.02 (m, 1H, CH$_2$), 2.28–2.38 (m, 1H, CH$_2$), 2.81–2.93 (m, 4H, CH$_3$, CH$_2$), 3.12 (dd, *J* = 16.00, 4.00 Hz, 1H, CH$_2$), 3.89 (br s, 1H, CH), 4.19 (d, *J* = 5.60 Hz, 1H, CH), 7.05–7.17 (m, 3H, CH, NH), 7.28 (t, *J* = 7.46 Hz, 1H, CH), 7.44 (t, *J* = 7.93 Hz, 2H, CH) ppm; ^{13}C NMR (126 MHz, DMSO-d$_6$): δ = 26.3, 31.8, 38.0, 50.6 (q, *J* = 30.9 Hz, C-4), 51.1, 122.1, 125.4 (q, *J* = 280.2 Hz, CF$_3$), 126.5, 130.1, 150.7, 156.0, 170.2 ppm; ^{19}F NMR (376 MHz, DMSO-d$_6$): δ = −78.56 (s, CF$_3$) ppm. $C_{14}H_{15}F_3N_2O_3$ (316.28). Found, %: C, 53.31; H, 4.91; N, 8.74. Calculated, %: C, 53.17; H, 4.78; N, 8.86. LCMS: [MH]$^+$ 317.

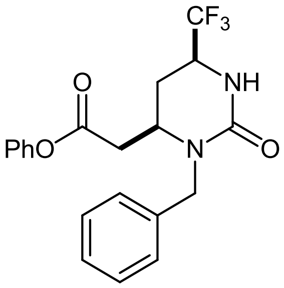

Phenyl 2-(3-benzyl-2-oxo-6-(trifluoromethyl)hexahydropyrimidin-4-yl)acetate (11b). White solid, yield: 0.71 g (90%), m.p. 125–127 °C. ^{1}H NMR (500 MHz, DMSO-d$_6$): δ = 2.01–2.12 (m, 1H, CH$_2$), 2.27–2.38 (m, 1H, CH$_2$), 2.79 (dd, *J* = 16.47, 8.23 Hz, 1H, CH$_2$), 3.08 (dd, *J* = 16.47, 4.39 Hz, 1H, CH$_2$), 3.89 (br s, 1H, CH), 4.29 (d, *J* = 6.59 Hz, 1H, CH), 4.35 (d, *J* = 16.47 Hz, 1H, CH$_2$), 4.91 (d, *J* = 15.92 Hz, 1H, CH$_2$), 7.05 (d, *J* = 7.14 Hz, 2H, CH), 7.21–7.32 (m, 5H, CH, NH), 7.35 (d, *J* = 7.68 Hz, 2H, CH), 7.42 (t, *J* = 7.68 Hz, 2H, CH) ppm; ^{13}C NMR (101 MHz, DMSO-d$_6$): δ = 25.8, 37.7, 47.3, 49.4, 50.5 (q, *J* = 30.2 Hz, C-4), 122.1, 125.4 (q, *J* = 281.0 Hz, CF$_3$), 126.5, 127.3, 127.4, 128.9, 130.0, 139.1, 150.5, 155.9, 169.9 ppm; ^{19}F NMR (188 MHz, DMSO-d$_6$): δ = −75.93 (s, CF$_3$) ppm. $C_{20}H_{19}F_3N_2O_3$ (392.37). Found, %: C, 61.20; H, 4.88; N, 7.16. Calculated, %: C, 61.22; H, 4.88; N, 7.14. LCMS: [MH]$^+$ 393.

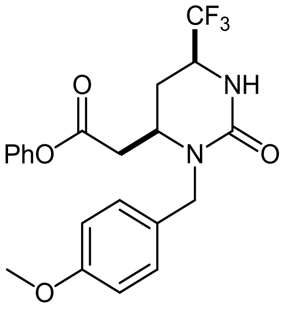

Phenyl 2-(3-(4-methoxybenzyl)-2-oxo-6-(trifluoromethyl)hexahydropyrimidin-4-yl)acetate (11c). White solid, yield: 0.81 g (96%), m.p. 138–140 °C. ^{1}H NMR (400 MHz, DMSO-d$_6$): δ = 1.92–2.12 (m, 1H, CH$_2$), 2.18-2.33 (m, 1H, CH$_2$), 2.76 (dd, *J* = 16.32, 7.93 Hz, 1H, CH$_2$), 3.08 (d, *J* = 15.86 Hz, 1H, CH$_2$), 3.73 (s, 3H, CH$_3$), 3.83 (br s, 1H, CH), 4.22 (d, *J* = 14.92 Hz, 2H, CH, CH$_2$), 4.85 (d, *J* = 15.86 Hz, 1H, CH$_2$), 6.90 (d, *J* = 6.53 Hz, 2H, CH), 7.05 (d, *J* = 6.53 Hz, 2H, CH), 7.20 (d, *J* = 6.53 Hz, 2H, CH), 7.27 (br s, 2H, CH, NH), 7.41 (br s, 2H, CH) ppm; ^{13}C NMR (101 MHz, DMSO-d$_6$): δ = 25.8, 37.6, 46.6, 49.1, 50.6 (q, *J* = 30.1 Hz, C-4), 55.5, 114.3, 122.1, 125.4 (q, *J* = 281.0 Hz, CF$_3$), 126.5, 128.9, 130.0, 130.8, 150.6, 155.9, 158,7, 169.9 ppm; ^{19}F NMR (376 MHz, DMSO-d$_6$): δ = −75.94 (s, CF$_3$) ppm. $C_{21}H_{21}F_3N_2O_4$ (422.40). Found, %: C, 59.66; H, 5.00; N, 6.71. Calculated, %: C, 59.71; H, 5.01; N, 6.63. LCMS: [MH]$^+$ 423.

2.8. General procedure for the synthesis of compounds 9–11d.

Compound **9–11c** (2 mmol) was dissolved in trifluoroacetic acid (10 mL). The solution was stirred at 72 °C for 20 minutes. Trifluoroacetic acid was evaporated and the residue was treated with methyl *tert*-butyl ether (10 mL). The mixture was filtered and the obtained filtrate was diluted with hexane (10 mL). The white precipitate formed was collected by filtration and recrystallized from toluene/hexane 1:1.

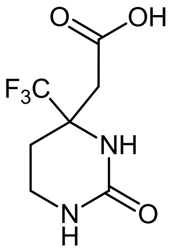

2-(2-Oxo-4-(trifluoromethyl)hexahydropyrimidin-4-yl)acetic acid (9d). White solid, yield: 0.35 g (79%), m.p. 213–215 °C. ^{1}H NMR (500 MHz, DMSO-d$_6$): δ = 2.03–2.12 (m, 1H, CH$_2$), 2.12–2.20 (m, 1H, CH$_2$), 2.63–2.71 (m, 2H, CH$_2$), 3.07–3.20 (m, 2H, CH$_2$), 6.63 (s, 1H, NH), 6.83 (s, 1H, NH), 12.67 (br s, 1H, COOH) ppm; ^{13}C NMR (126 MHz, DMSO-d$_6$): δ = 24.9, 36.2, 39.1, 57.4 (q, *J* = 26.9 Hz, C-4), 126.9 (q, *J* = 288.5 Hz, CF$_3$), 155.1, 170.5 ppm; ^{19}F NMR (376 MHz, DMSO-d$_6$): δ = −77.68 (s, CF$_3$) ppm. $C_7H_9F_3N_2O_3$ (226.15). Found, %: C, 37.31; H, 4.14; N, 12.35. Calculated, %: C, 37.18; H, 4.01; N, 12.39. LCMS: [MH]$^+$ 227.

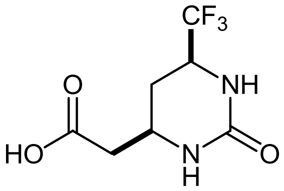

2-(2-Oxo-6-(trifluoromethyl)hexahydropyrimidin-4-yl)acetic acid (10d). White solid, yield: 0.32 g (70%), m.p. 231–233 °C. ^{1}H NMR (400 MHz, DMSO-d$_6$): δ = 1.48 (q, *J* = 12.12 Hz, 1H, CH_2), 2.15 (d, *J* = 12.12 Hz, 1H, CH_2), 2.38 (dd, *J* = 16.79, 7.46 Hz, 1H, CH_2), 2.56 (m, *J* = 5.60 Hz, 1H, CH_2), 3.57–3.73 (m, 1H, CH), 4.07–4.24 (m, 1H, CH), 6.45 (br s, 1H, NH), 7.02 (br s, 1H, NH), 12.24 (br s, 1H, COOH) ppm; ^{13}C NMR (101 MHz, DMSO-d$_6$): δ = 27.1, 39.5, 45.6, 51.5 (q, *J* = 30.1 Hz, C-4), 125.4 (q, *J* = 281.8 Hz, CF_3), 156.3, 172.5 ppm; ^{19}F NMR (376 MHz, DMSO-d$_6$): δ = −77.20 (s, CF_3) ppm. $C_7H_9F_3N_2O_3$ (226.15). Found, %: C, 37.00; H, 3.92; N, 12.48. Calculated, %: C, 37.18; H, 4.01; N, 12.39. LCMS: $[MH]^+$ 227.

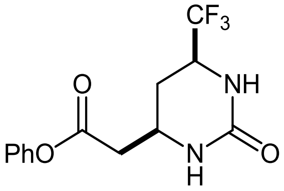

Phenyl 2-(2-oxo-6-(trifluoromethyl)hexahydropyrimidin-4-yl)acetate (11d). White solid, yield: 0.44 g (73%), m.p. 125–127 °C. ^{1}H NMR (400 MHz, DMSO-d$_6$): δ = 1.62 (q, *J* = 11.19 Hz, 1H, CH_2), 2.22 (d, *J* = 13.06 Hz, 1H, CH_2), 2.77–2.92 (m, 2H, CH_2), 3.84 (d, *J* = 7.46 Hz, 1H, CH), 4.16–4.27 (m, 1H, CH), 6.66 (s, 1H, NH), 6.97 (s, 1H, NH), 7.18 (d, *J* = 8.39 Hz, 2H, CH), 7.26 (t, *J* = 7.46 Hz, 1H, CH), 7.42 (t, *J* = 7.46 Hz, 2H, CH) ppm; ^{13}C NMR (126 MHz, DMSO-d$_6$): δ = 27.1, 39.4, 45.4, 51.6 (q, *J* = 30.8 Hz, C-4), 122.3, 125.7 (q, *J* = 281.7 Hz, CF_3), 126.4, 129.9, 150.8, 156.1, 169.6 ppm; ^{19}F NMR (376 MHz, DMSO-d$_6$): δ = −77.11 (s, CF_3) ppm. $C_{13}H_{13}F_3N_2O_3$ (302.25). Found, %: C, 51.69; H, 4.34; N, 9.26. Calculated, %: C, 51.66; H, 4.34; N, 9.27. LCMS: $[MH]^+$ 303.

3. Representative procedure for the synthesis of 2-[2-oxo-6-(trifluoromethyl)-1,2,3,4-tetrahydropyrimidin-4-yl]acetamides

The solution of compound **6a** (0.63 g, 2 mmol), **6j** (0.93 g, 2 mmol) or **8a** (0.72 g, 2 mmol) and benzylamine (0.42 g, 4 mmol) in dichloromethane (10 mL) was stirred at 40 °C for 4 hours. The mixture was cooled to room temperature and the product was separated by filtration. The solid was washed consecutively with dichloromethane (10 mL), 0.1 M hydrochloric acid (10 mL), water (10 mL) and then dried on air.

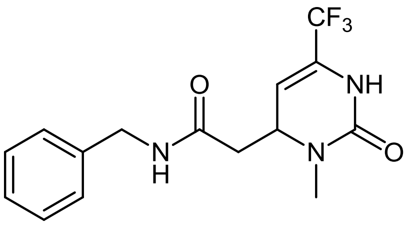

***N*-Benzyl-2-(3-methyl-2-oxo-6-(trifluoromethyl)-1,2,3,4-tetrahydropyrimidin-4-yl)acetamide.** White solid, yield: 0.5 g (77 %, from **6a**), 0.53 g (81 %, from **8a**), m.p. 207–209 °C. ^{1}H NMR (400 MHz, DMSO-d$_6$): δ = 2.30–2.43 (m, 1H, CH$_2$), 2.60–2.69 (m, 1H, CH$_2$), 2.80 (s, 3H, CH$_3$), 4.20–4.35 (m, 2H, CH$_2$), 4.39 (s, 1H, CH), 5.40 (s, 1H, CH), 7.17–7.36 (m, 5H, CH), 8.53 (br s, 1H, NH), 9.38 (br s, 1H, NH) ppm; ^{13}C NMR (126 MHz, DMSO-d$_6$): δ = 32.9, 40.6, 42.6, 55.1, 102.8, 120.3 (q, *J* = 273.4 Hz, CF$_3$), 127.3, 127.4 (q, *J* = 34.4 Hz, C-4), 127.7, 128.7, 139.7, 152.8, 169.2 ppm; ^{19}F NMR (376 MHz, DMSO-d$_6$): δ = −69.57 (s, CF$_3$) ppm. $C_{15}H_{16}F_3N_3O_2$ (327.30). Found, %: C, 54.88; H, 4.92; N, 12.84. Calculated, %: C, 55.04; H, 4.93; N, 12.84. LCMS: [MH]$^+$ 328.

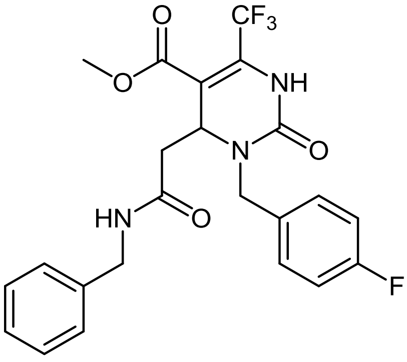

Methyl 4-(2-(benzylamino)-2-oxoethyl)-3-(4-fluorobenzyl)-2-oxo-6-(trifluoromethyl)-1,2,3,4-tetrahydropyrimidine-5-carboxylate. White solid, yield: 0.77 g (80 %, from **6**j), m.p. 170–172 °C. ^{1}H NMR (400 MHz, DMSO-d$_6$): δ = 2.55–2.63 (m, 2H, CH$_2$), 3.60 (s, 3H, CH$_3$), 4.06 (d, *J* = 15.86 Hz, 1H, CH$_2$), 4.26 (d, *J* = 3.70 Hz 2H, CH$_2$), 4.58 (t, *J* = 5.13 Hz, 1H, CH), 4.89 (d, *J* = 14.92 Hz, 1H, CH$_2$), 7.17 (t, *J* = 8.39 Hz, 2H, CH), 7.17–7.32 (m, 5H, CH), 7.33 (t, *J* = 7.46 Hz, 2H, CH), 8.57 (br s, 1H, NH), 10.08 (br s, 1H, NH) ppm; ^{13}C NMR (126 MHz, DMSO-d$_6$): δ = 39.0, 42.8, 47.6, 52.6, 53.5, 107.2, 115.8 (d, *J* = 21.9 Hz), 119.7 (q, *J* = 275.9 Hz, CF$_3$), 127.3, 127.9, 128.7, 129.8 (d, *J* = 8.0 Hz), 133.0(q, *J* = 33.9 Hz, C-4), 133.9, 139.5, 152.2, 161.9 (d, *J* = 242.9 Hz), 163.7 169.0 ppm. ^{19}F NMR (376 MHz, DMSO-d$_6$): δ = −65.45 (s, CF$_3$), −116.07 (s, CF) ppm. $C_{23}H_{21}F_4N_3O_4$ (479.42). Found, %: C, 57.58; H, 4.48; N, 8.77. Calculated, %: C, 57.62; H, 4.42; N, 8.76. LCMS: [MH]$^+$ 480.

4. Representative procedure for the hydrolysis of compounds 6 and 11

To the solution of compound **6g** or **11b** (0.78 g, 2 mmol) in tetrahydrofuran (10 mL) was added a solution of lithium hydroxide hydrate (0.17 g, 4 mmol) in water (10 mL) and the mixture was stirred at 40 °C for 2 hours. After cooling to room temperature the mixture was

evaporated and 0.1 M hydrochloric acid was added (20 mL). The precipitated solid was filtered, washed with water (10 mL) and recrystallized from methanol/ water 1:1. Compounds **5g** (0.42 g, 67%, from **6g**) or **10b** (0.56 g, 89%, from **11b**) were obtained.

5. X-ray structure determination for compound 11b

5.1. Experimental part

The colourless crystals of **11b (**$C_{20}H_{19}N_2O_3F_3$) are monoclinic. At 293 *K*a = 24.814(6), *b* = 5.601(1), *c* = 29.859(10) Å, β = 114.49(3)°, *V* = 3776(2) Å^3, M_r = 392.37, *Z* = 8, space group $P2_1/n$, d_{calc}=1.380 g/cm^3, μ(Mo K_α)=0.113 mm^{-1}, F(000) = 1632. Intensities of 23485 reflections (6603 independent, R_{int} = 0.277) were measured on the «Xcalibur-3» diffractometer (graphite monochromated Mo K_α radiation, CCD detector, ω-scaning, $2\Theta_{max}$ = 50°). The structure was solved by direct method using SHELXTL package [7]. Positions of the hydrogen atoms were located from electron density difference maps and refined by “riding” model with U_{iso} = $1.2U_{eq}$ of the carrier atom. Full-matrix least-squares refinement against F^2 in anisotropic approximation for non-hydrogen atoms using 6603 reflections was converged to wR_2 = 0.164 (R_1 = 0.070 for 1304 reflections with F>4σ(F), S = 0.663). The final atomic coordinates, and crystallographic data for molecule **11b** have been deposited to with the Cambridge Crystallographic Data Centre, 12 Union Road, CB2 1EZ, UK (fax: +44-1223-336033; e-mail: deposit@ccdc.cam.ac.uk) and are available on request quoting the deposition numbers CCDC 1555861).

5.2. The molecular and crystal structure of the compound 11b

There are two enantiomers (A and B) with the different configurations of the C1 and C3 asymmetric atoms found in the crystals of **11b**. The heterocycle adopts a half-chair conformation in molecules A and B (puckering parameters [8] are: S = 0.71, Θ = 41.5°, Ψ = 29.5° in molecule A and S = 0.70, Θ = 38.2°, Ψ = 27.2° in molecule B) (Figure S1). Deviations of the C2 and C3 atoms from the mean plane of the remaining atoms of the ring are 0.37 Å and −0.35 Å, respectively in molecule A and −0.40 Å and 0.28 Å in molecule B. The phenyl ring of the benzyl substituent is located in ±*ac*-conformation relatively the C4-N1 endocyclic bond and is turned to the N1-C5 bond (the C4-N1-C5-C6 torsion angle is 129.7(6)° in A and -126.6(6)° in B; the N1-C5-C6-C7 torsion angle is −38.9(9)° in A and 35.2(8)° in B). Trifluoromethyl group and the substituent at the C1 atom have equatorial orientations (the C4-N2-C3-C20 and C4-N1-C1-C12 torsion angles are −163.9(6)° A 163.1(6)° B and 134.7(6)° A −137.0(6)° B respectively). The carboxylic group is situated in ±*sc*-position relatively the N1-C1 endocyclic bond and is turned significantly relatively the C1-C12 bond (the N1-C1-C12-C13 torsion angle is −65.1(7)° in A and 68.4(7)° in B; the C1-C12-C13-O2 torsion angle is 66.1(8)° in A and −76(1)° in B). The phenyl ring of this substituent is located in *ap*-conformation to the C12-C13 bond and is turned to the C13-O3 bond (the C12-C13-O3-C14 torsion angle is 170.8(5)° in A and −172.5(6)° in B; the C13-O3-C14-C15 torsion angle is 63.6(9)° in A and −65.2(6)° in B).

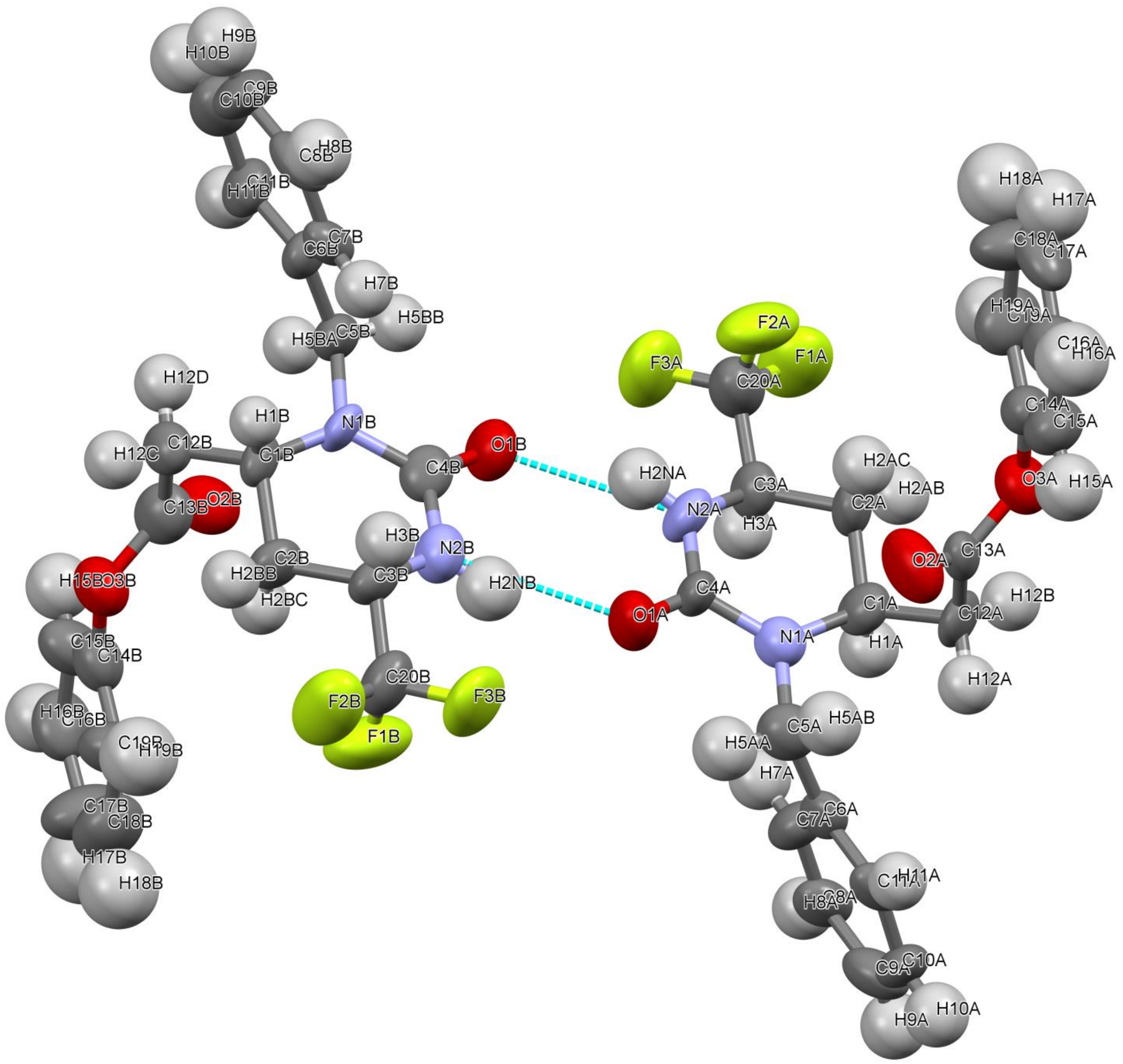

Figure S1: Structure of compound **11b** according to X-ray diffraction data. Thermal ellipsoids are defined at the 50 % probability level.

In the crystal phase molecules A and B form centrosymmetric dimers bonded by intermolecular hydrogen bonds: N2a-H…O1b' (H…O 2.12 Å N-H…O 152°) and N2b-H…O1a' (H…O 2.08 Å N-H…O 155°). In addition a lot of weak intermolecular hydrogen bonds are found in the crystal **11b**:

C10b-H…F2a' (0.5-x, -0.5+y, 0.5-z) H…F 2.51 Å C-H…F 163°;
C8a-H…C8a' (π (1.5-x, 0.5+y, 0.5-z) H…C 2.85 Å C-H…C 153°;
C8a-H…C9a' (π) (1.5-x, 0.5+y, 0.5-z) H…C 2.86 Å C-H…C 162°;
C8b-H…C8b' (π) (0.5-x, -0.5+y, 0.5-z) H…C 2.76 Å C-H…C 160°;
C3a-H…O1a' (x, 1+y, z) H…O 2.42 Å C-H…O 168°;
C3b-H…O1b' (x, y-1, z) H…O 2.44 Å C-H…O 166°.

6. References

1. Blaquiere, N.; Shore, D. G.; Rousseaux, S.; Fagnou K. *J. Org. Chem.* **2009,** *74(16),* 6190–6198. doi:10.1021/jo901022j

2. Bae, H. Y.; Sim, J. H.; Lee, J.-W.; List, B.; Song, C. E. *Angew. Chem. Int. Ed*. **2013,** *52(46),* 12143–12147. doi:10.1002/anie.201306297

3. Sukach, V. A.; Resetnic, A. A.; Tkachuk, V. M.; Lin, Z., Kortz, U.; Vovk, M. V.; Röschenthaler, G.-V. *Eur. J. Org. Chem.* **2015,** 1290–1301. doi:10.1002/ejoc.201403495

4. Zanatta, N.; Faoro, D.; Fernandes, L. da. S.; Brondani, P. B.; Flores, D. C.; Flores, A. F. C.; Bonacorso, H. G.; Martins, M. A. P. *Eur. J. Org. Chem.* **2008,** 5832–5838. doi:10.1002/ejoc.200800822

5. Sukach, V. A.; Tkachuk, V. M.; Shoba, V. M.; Pirozhenko, V. V.; Rusanov, E. B.; Chekotilo, A. A.; Röschenthaler, G-V.; Vovk, M. V. *Eur. J. Org. Chem.* **2014,** 1452–1460. doi:10.1002/ejoc.201301542

6. Sukach, V. A.; Tkachuk, V. M.; Rusanov, E. B.; Roeschenthaler, G.-V.; Vovk, M. V. *Tetrahedron* **2012,** *68,* 8408–8415. doi:10.1016/j.tet.2012.07.099

7. Sheldrick G.M. *Acta Crystallogr., Sect. A* **2008**, *A64*, 112–122. doi:10.1107/S0108767307043930

8. Zefirov,N. S.; Palyulin,V. A.; Dashevskaya, E. E. *J. Phys. Org. Chem.* **1990,** 3, 147–154. doi:10.1002/poc.610030304
