# Supplementary material for: Regioselective decarboxylative addition of malonic acid and its mono(thio)esters to 4-trifluoromethylpyrimidin-2(1H)-ones
Source: Beilstein J Org Chem. 2017 Dec 7;13:2617–25. doi: 10.3762/bjoc.13.259 (PMC5727768; doi:10.3762/bjoc.13.259)

Supporting Information File 2

for

Regioselective decarboxylative addition of malonic acid and its mono(thio)esters to 4-trifluoromethylpyrimidin-2(1*H*)-ones

Sergii V. Melnykov[1], Andrii S. Pataman[2], Yurii V. Dmytriv[2,3], Svitlana V. Shishkina[4,5], Mykhailo V. Vovk[1] and Volodymyr A. Sukach*[1]

Address: [1]Institute of Organic Chemistry, National Academy of Sciences of Ukraine, 5 Murmanska str., Kyiv 02660, Ukraine, [2]Enamine LTD, 78 Chervonotkats‘ka str., Kyiv 02094, Ukraine, [3]National Technical University of Ukraine “Igor Sikorsky Kyiv Polytechnic Institute”, 37 Peremohy ave., Kyiv 03056, Ukraine, [4]STC ‘‘Institute for Single Crystals’’, National Academy of Sciences of Ukraine, 60 Nauky ave., Kharkiv 61001, Ukraine and [5]Department of Inorganic Chemisrtry, V.M. Karasin Kharkiv National University, 4 Svobody sq, Kharkiv 61122, Ukraine

Email: Volodymyr A. Sukach - vsukach@gmail.com

* Corresponding author

Copies of the ^{1}H, ^{13}C, and ^{19}F NMR spectra

Compound **4a**

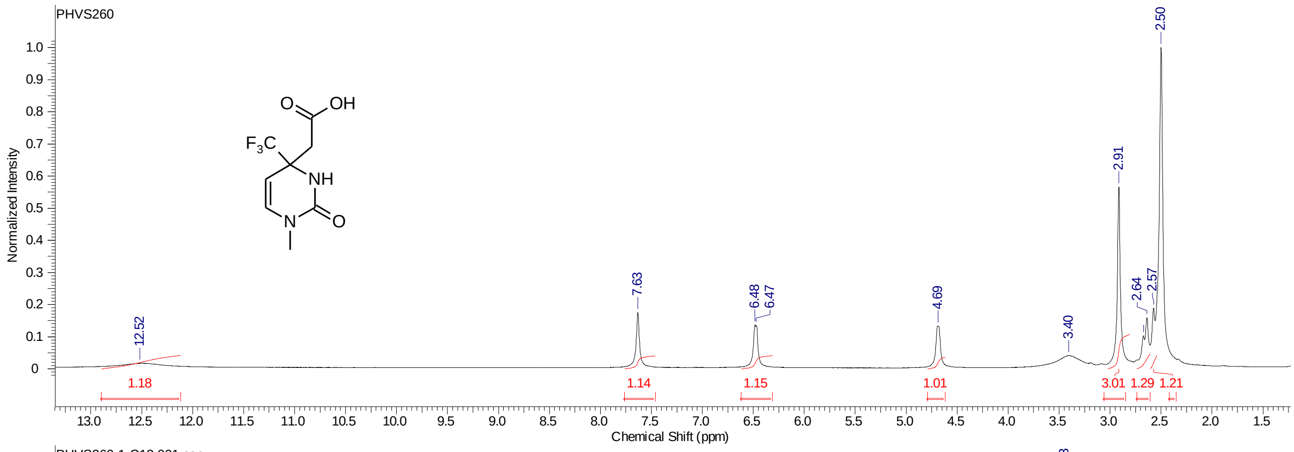
PHVS260
Normalized Intensity
Chemical Shift (ppm)
12.52
7.63
6.48
6.47
4.69
3.40
2.91
2.64
2.57
2.50
1.18
1.14
1.15
1.01
3.01
1.29
1.21
O
OH
F3C
NH
N
O

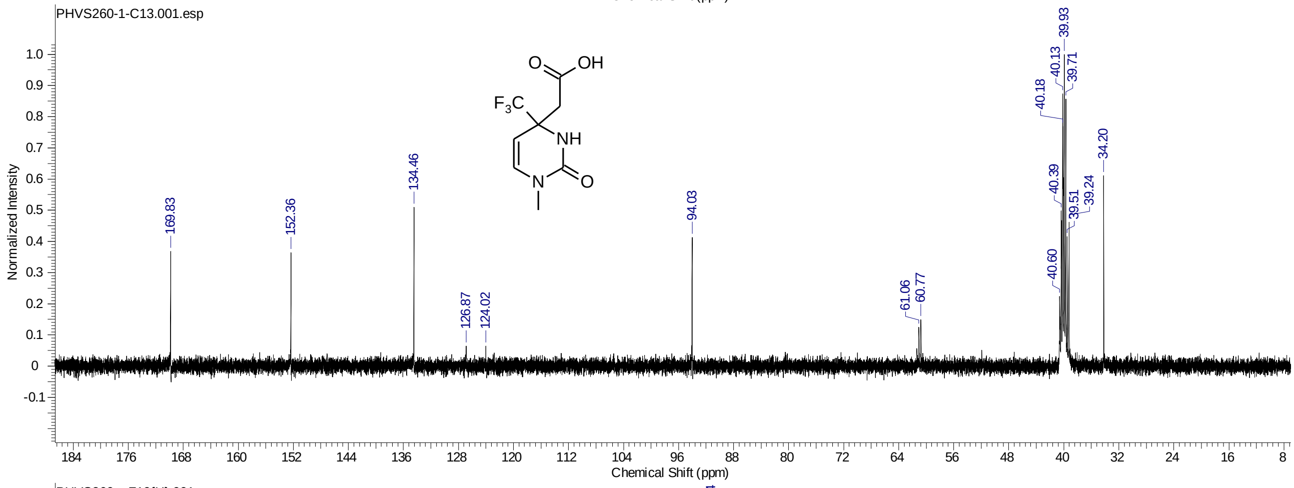
PHVS260-1-C13.001.esp
Normalized Intensity
Chemical Shift (ppm)
169.83
152.36
134.46
126.87
124.02
94.03
61.06
60.77
40.60
40.39
40.18
40.13
39.93
39.71
39.51
39.24
34.20
O
OH
F3C
NH
N
O

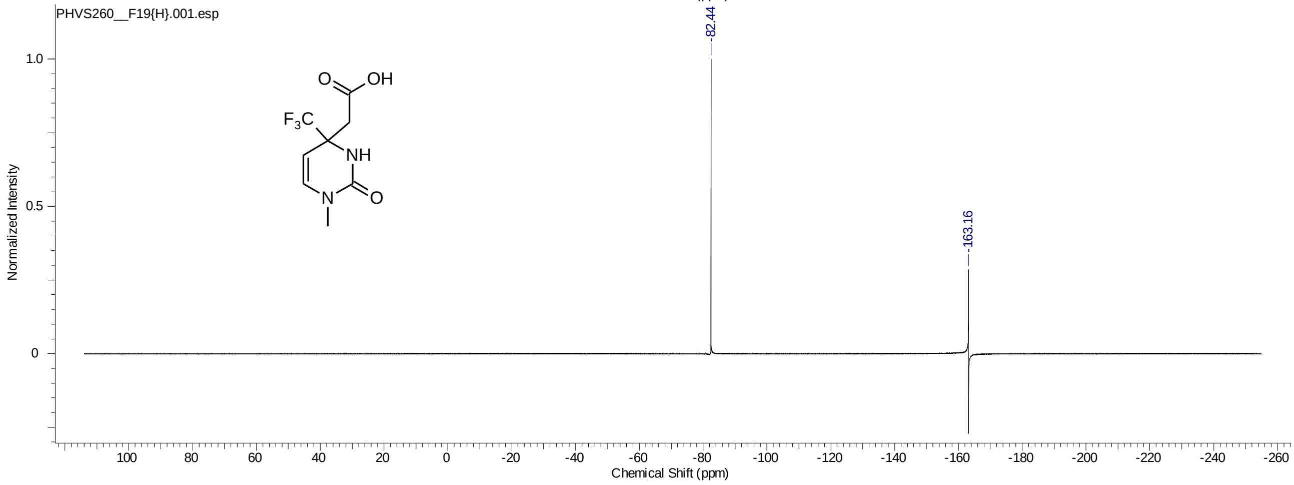
PHVS260__F19{H}.001.esp
Normalized Intensity
Chemical Shift (ppm)
-82.44
-163.16
O
OH
F3C
NH
N
O

Compound **4b**

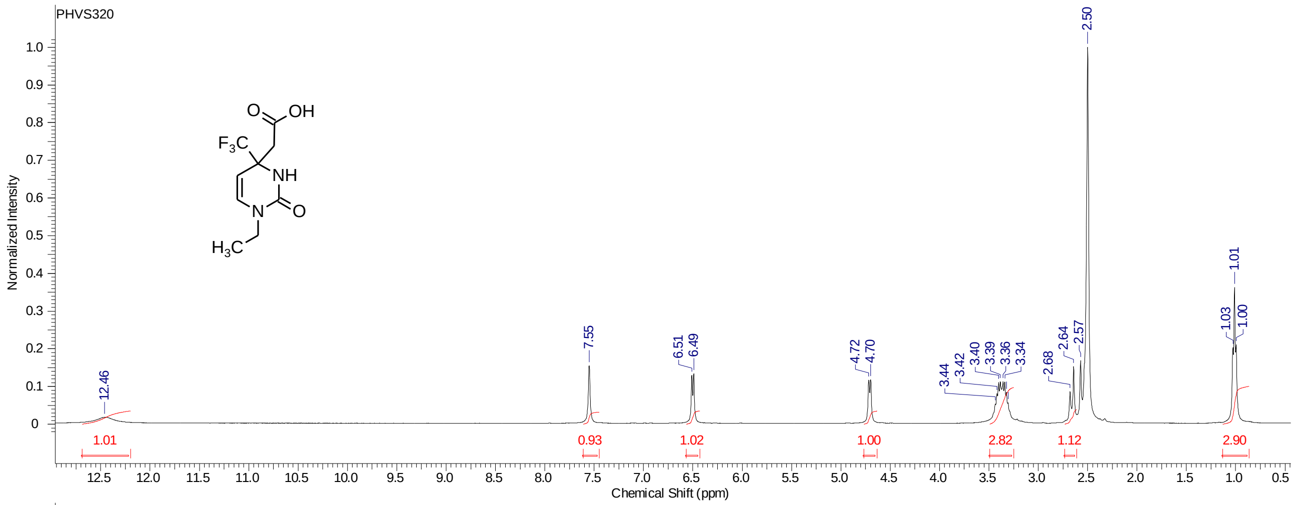
PHVS320
12.46
7.55
6.51
6.49
4.72
4.70
3.44
3.42
3.40
3.39
3.36
3.34
2.68
2.64
2.57
2.50
1.03
1.01
1.00
1.01
0.93
1.02
1.00
2.82
1.12
2.90
Normalized Intensity
Chemical Shift (ppm)
O
OH
F_3C
NH
N
O
H_3C

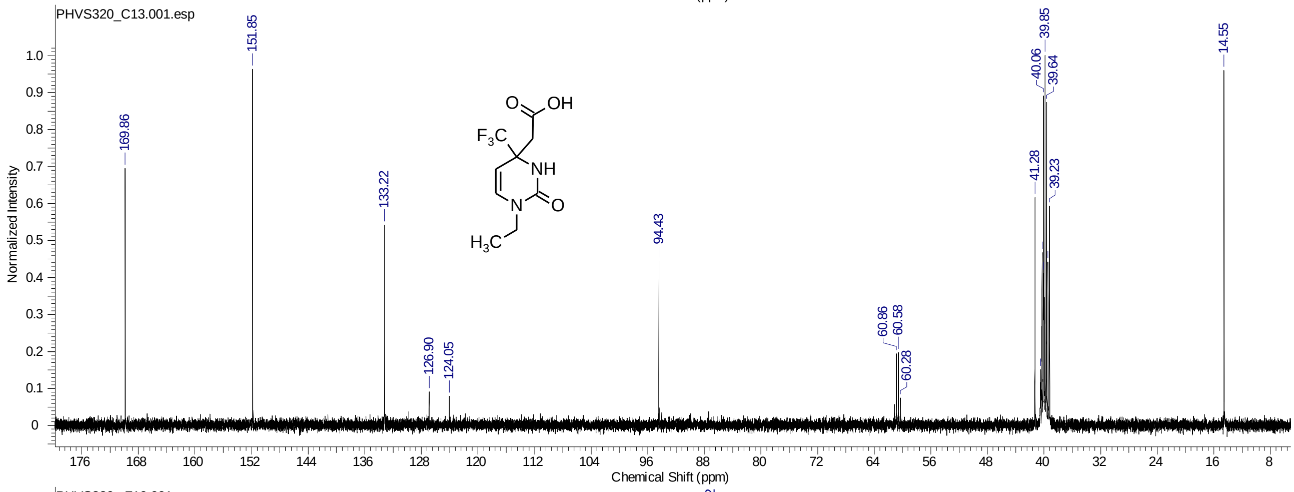
PHVS320_C13.001.esp
169.86
151.85
133.22
126.90
124.05
94.43
60.86
60.58
60.28
41.28
40.06
39.85
39.64
39.23
14.55
Normalized Intensity
Chemical Shift (ppm)
O
OH
F_3C
NH
N
O
H_3C

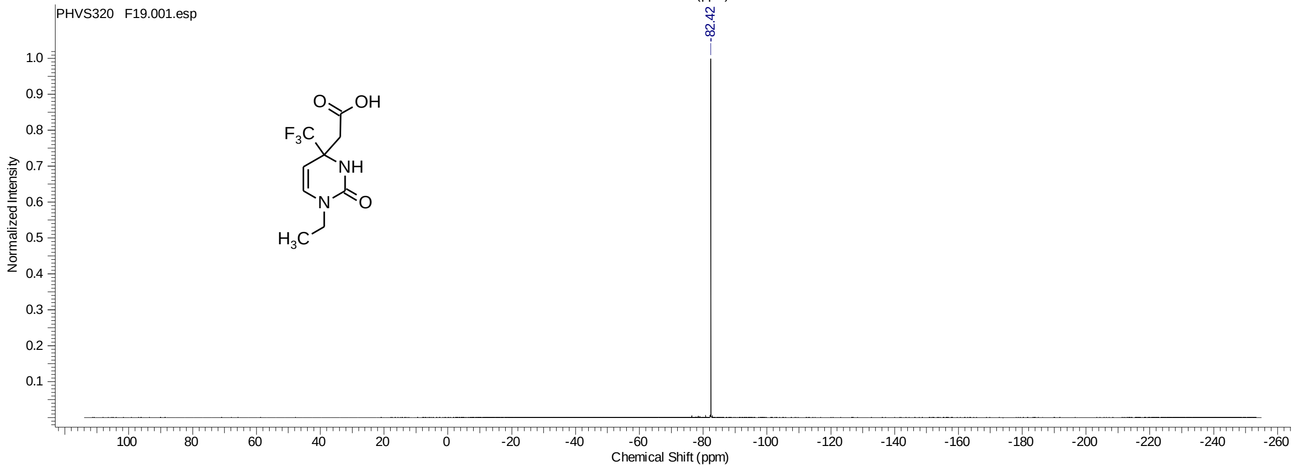
PHVS320_F19.001.esp
-82.42
Normalized Intensity
Chemical Shift (ppm)
O
OH
F_3C
NH
N
O
H_3C

Compound **4c**

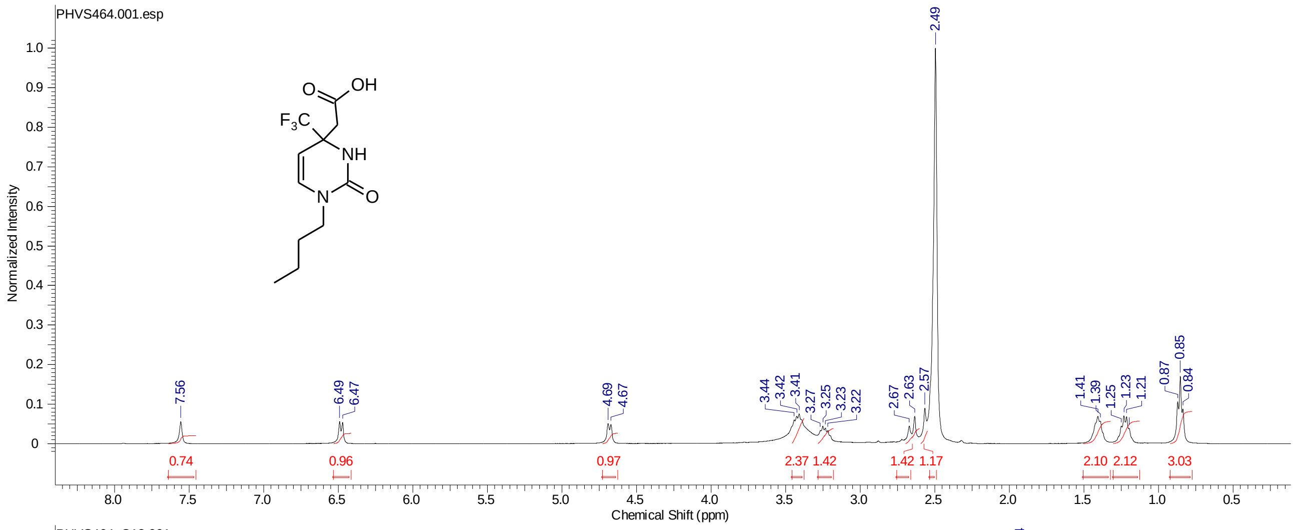
PHVS464.001.esp
Normalized Intensity
Chemical Shift (ppm)
O
OH
F3C
NH
N
O
7.56
6.49
6.47
4.69
4.67
3.44
3.42
3.41
3.27
3.25
3.23
3.22
2.67
2.63
2.57
2.49
1.41
1.39
1.25
1.23
1.21
0.87
0.85
0.84
0.74
0.96
0.97
2.37
1.42
1.42
1.17
2.10
2.12
3.03

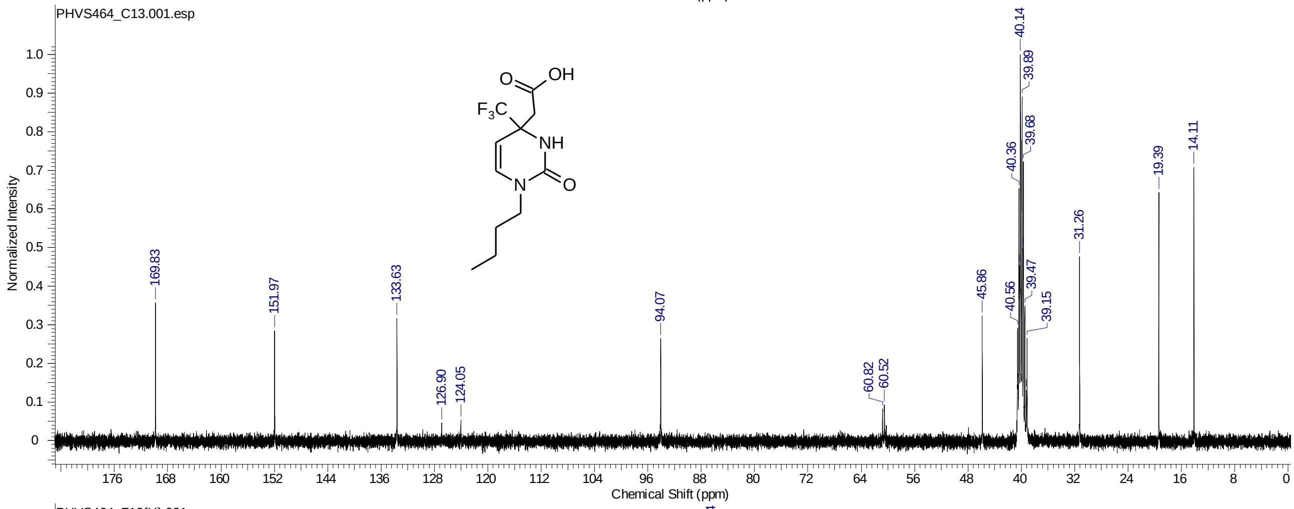
PHVS464_C13.001.esp
Normalized Intensity
Chemical Shift (ppm)
169.83
151.97
133.63
126.90
124.05
94.07
60.82
60.52
45.86
40.56
40.36
40.14
39.89
39.68
39.47
39.15
31.26
19.39
14.11

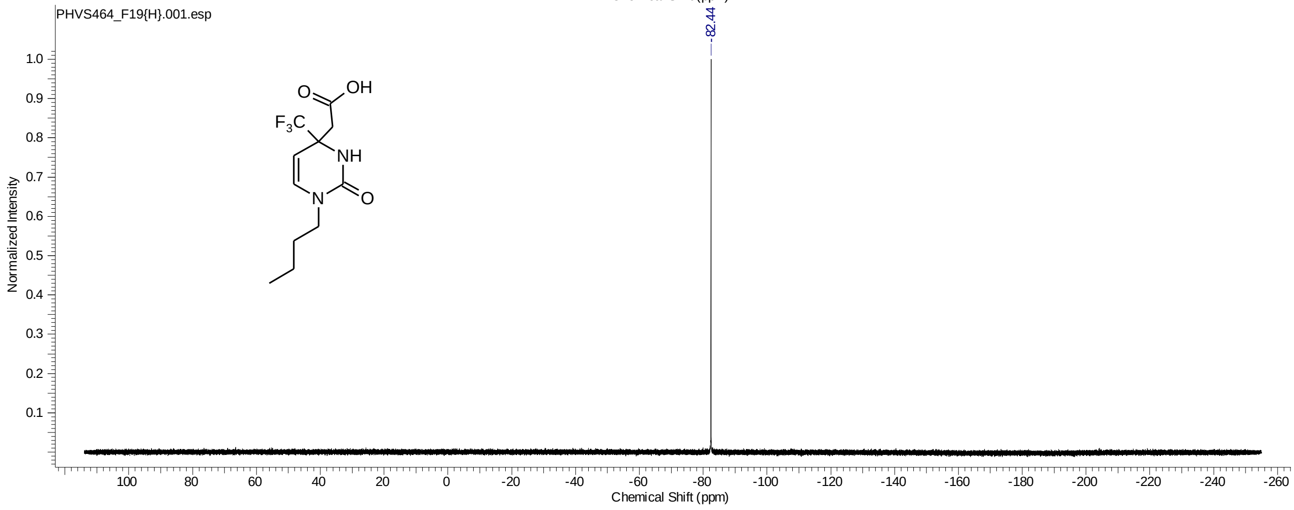
PHVS464_F19{H}.001.esp
Normalized Intensity
Chemical Shift (ppm)
-82.44

Compound **4d**

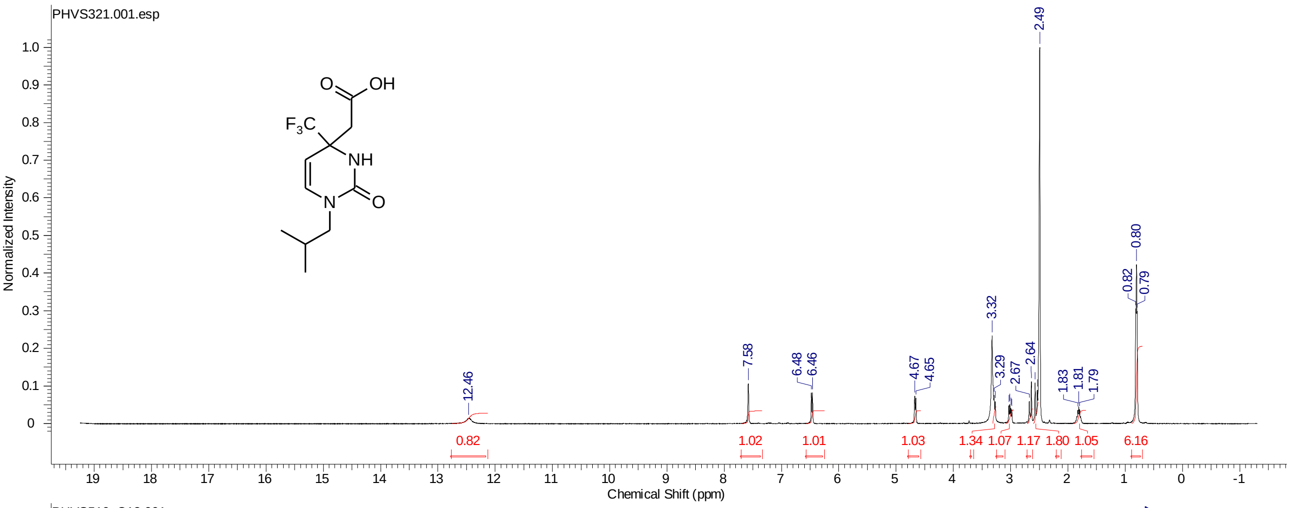
PHVS321.001.esp
Normalized Intensity
Chemical Shift (ppm)
O
OH
F3C
NH
N
O
12.46
7.58
6.48
6.46
4.67
4.65
3.32
3.29
2.67
2.64
2.49
1.83
1.81
1.79
0.82
0.80
0.79
0.82
1.02
1.01
1.03
1.34
1.07
1.17
1.80
1.05
6.16

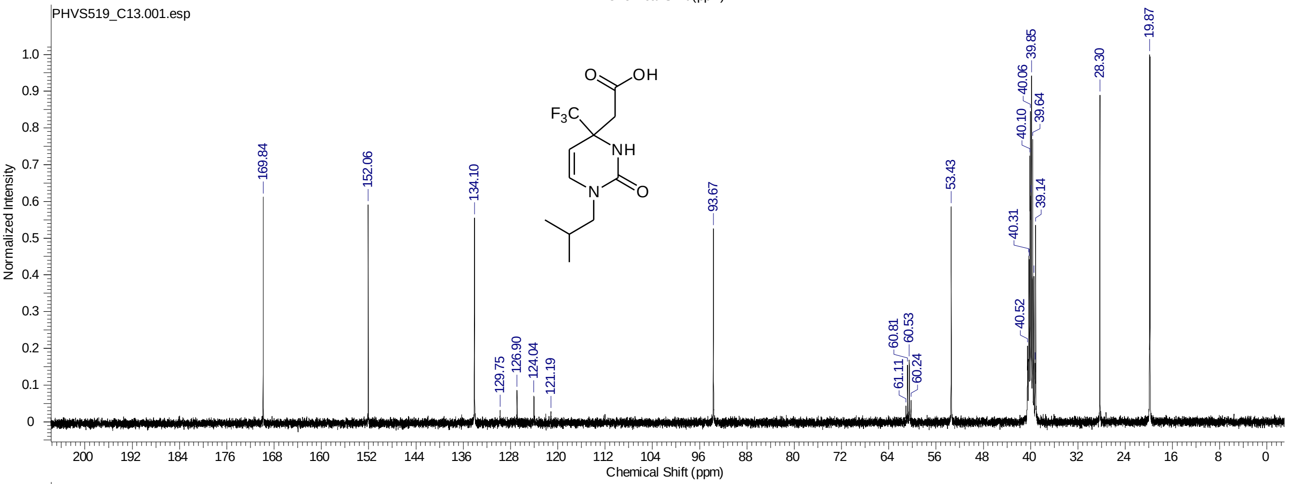
PHVS519_C13.001.esp
Normalized Intensity
Chemical Shift (ppm)
O
OH
F3C
NH
N
O
169.84
152.06
134.10
129.75
126.90
124.04
121.19
93.67
61.11
60.81
60.53
60.24
53.43
40.52
40.31
40.10
40.06
39.85
39.64
39.14
28.30
19.87

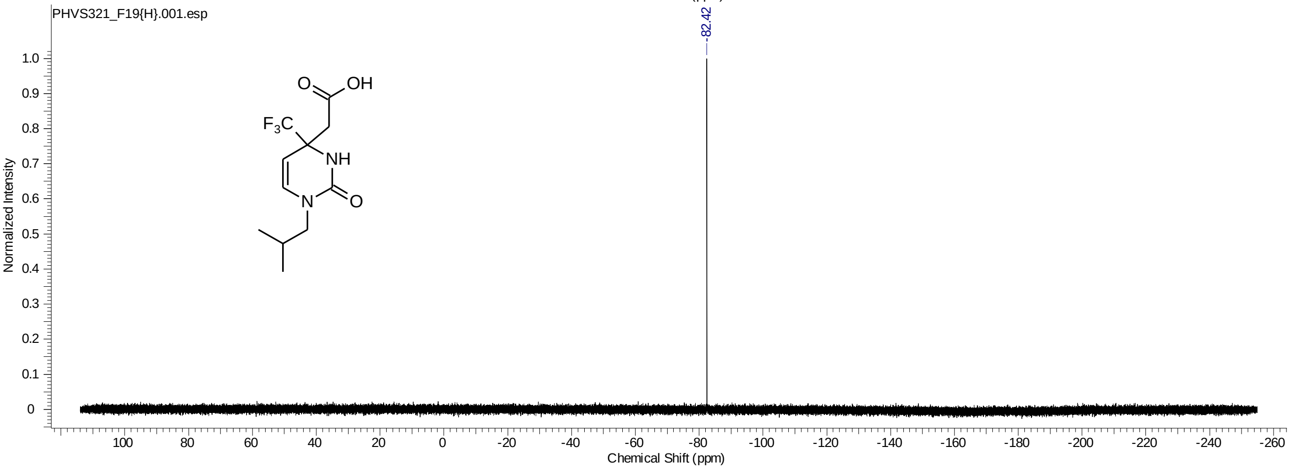
PHVS321_F19{H}.001.esp
Normalized Intensity
Chemical Shift (ppm)
O
OH
F3C
NH
N
O
-82.42

Compound **4e**

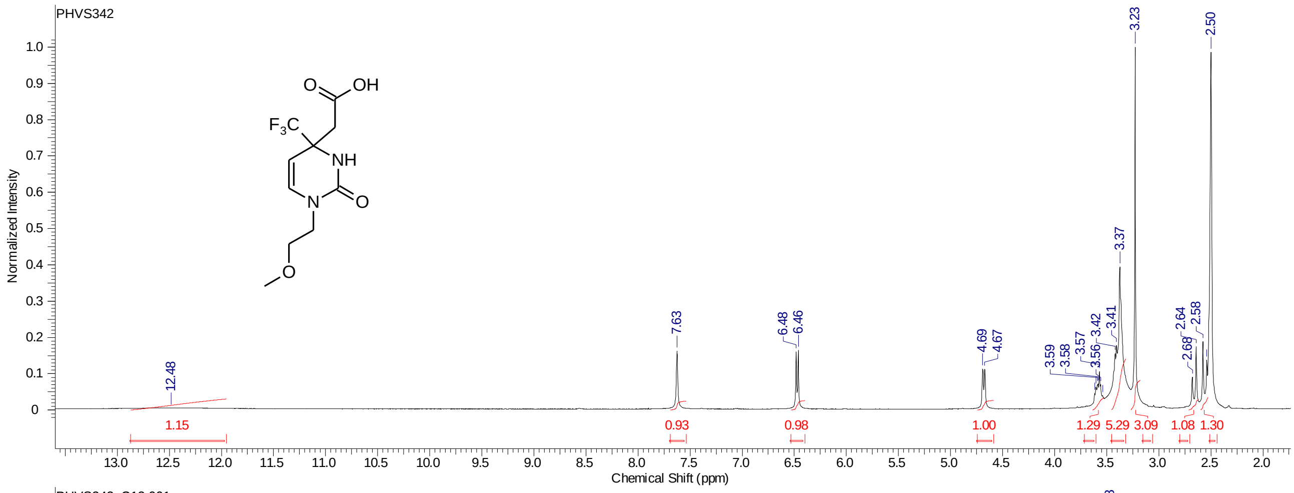
PHVS342
Normalized Intensity
Chemical Shift (ppm)
OH
O
F3C
NH
N
O
O
12.48
7.63
6.48
6.46
4.69
4.67
3.59
3.58
3.57
3.56
3.42
3.41
3.37
3.23
2.68
2.64
2.58
2.50
1.15
0.93
0.98
1.00
1.29
5.29
3.09
1.08
1.30

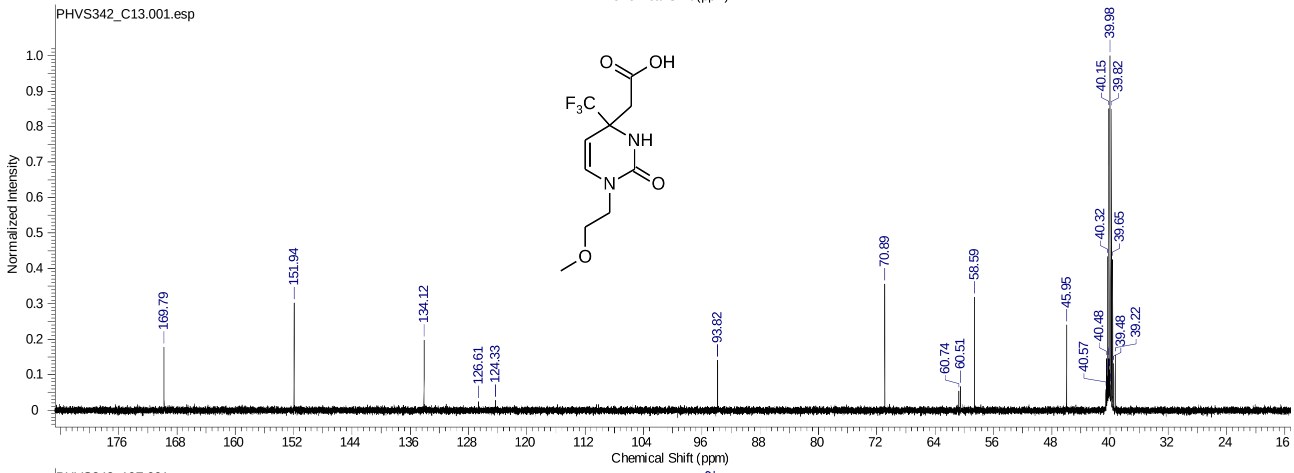
PHVS342_C13.001.esp
Normalized Intensity
Chemical Shift (ppm)
OH
O
F3C
NH
N
O
O
169.79
151.94
134.12
126.61
124.33
93.82
70.89
60.74
60.51
58.59
45.95
40.57
40.48
40.32
40.15
39.98
39.82
39.65
39.48
39.22

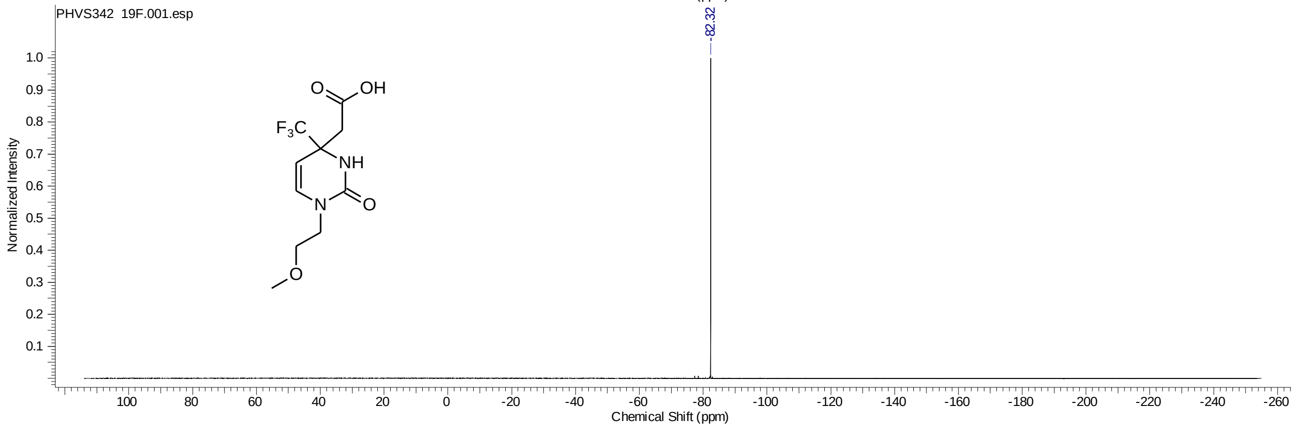
PHVS342_19F.001.esp
Normalized Intensity
Chemical Shift (ppm)
OH
O
F3C
NH
N
O
O
-82.32

Compound **4f**

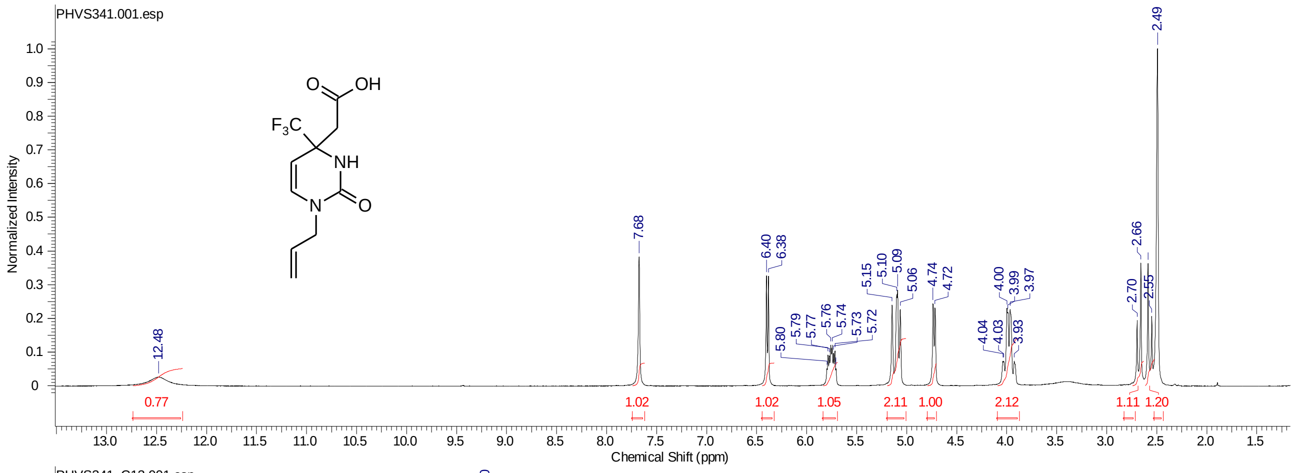
PHVS341.001.esp
Normalized Intensity
Chemical Shift (ppm)
O
OH
F3C
NH
N
O
12.48
7.68
6.40
6.38
5.80
5.79
5.77
5.76
5.74
5.73
5.72
5.15
5.10
5.09
5.06
4.74
4.72
4.04
4.03
4.00
3.99
3.97
3.93
2.70
2.66
2.55
2.49
0.77
1.02
1.02
1.05
2.11
1.00
2.12
1.11
1.20

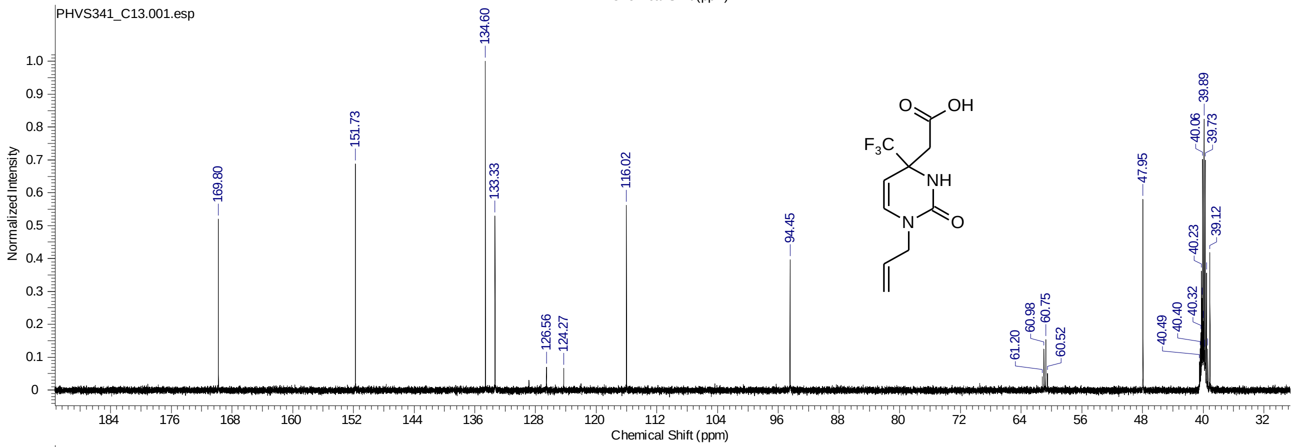
PHVS341_C13.001.esp
Normalized Intensity
Chemical Shift (ppm)
O
OH
F3C
NH
N
O
169.80
151.73
134.60
133.33
126.56
124.27
116.02
94.45
61.20
60.98
60.75
60.52
47.95
40.49
40.40
40.32
40.23
40.06
39.89
39.73
39.12

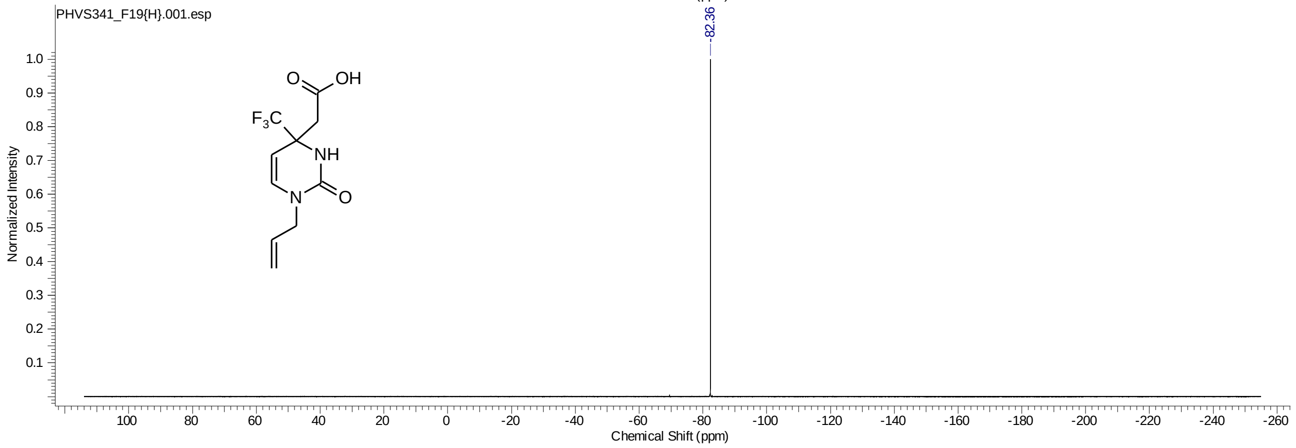
PHVS341_F19{H}.001.esp
Normalized Intensity
Chemical Shift (ppm)
O
OH
F3C
NH
N
O
-82.36

Compound **4g**

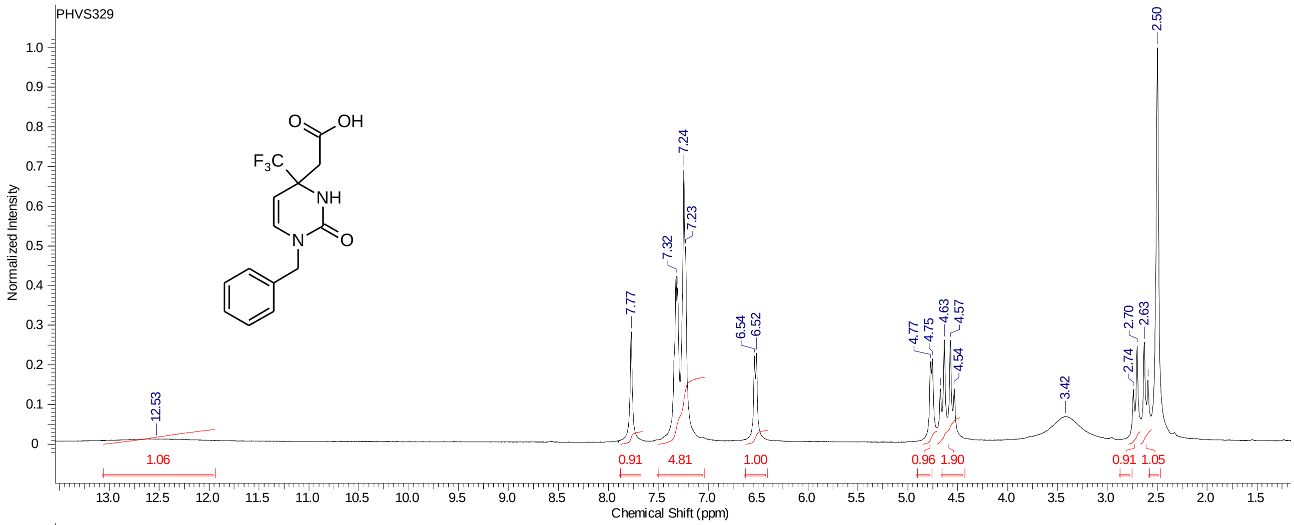
PHVS329
Normalized Intensity
Chemical Shift (ppm)
OH
O
F3C
NH
N
O
12.53
7.77
7.32
7.24
7.23
6.54
6.52
4.77
4.75
4.63
4.57
4.54
3.42
2.74
2.70
2.63
2.50
1.06
0.91
4.81
1.00
0.96
1.90
0.91
1.05

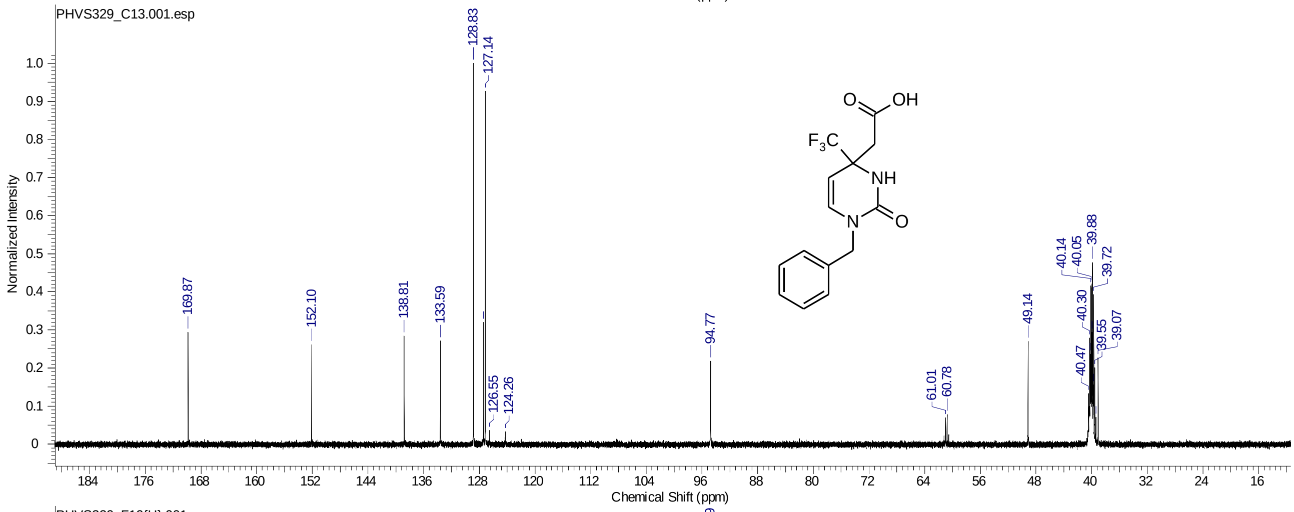
PHVS329_C13.001.esp
Normalized Intensity
Chemical Shift (ppm)
169.87
152.10
138.81
133.59
128.83
127.14
126.55
124.26
94.77
61.01
60.78
49.14
40.47
40.30
40.14
40.05
39.88
39.72
39.55
39.07

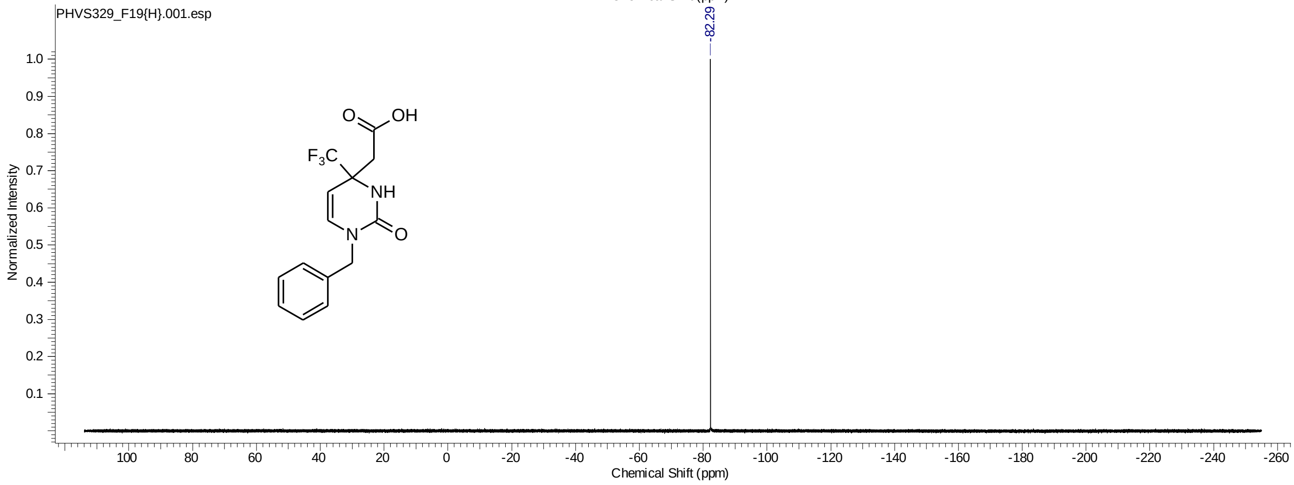
PHVS329_F19{H}.001.esp
Normalized Intensity
Chemical Shift (ppm)
-82.29

Compound **4h**

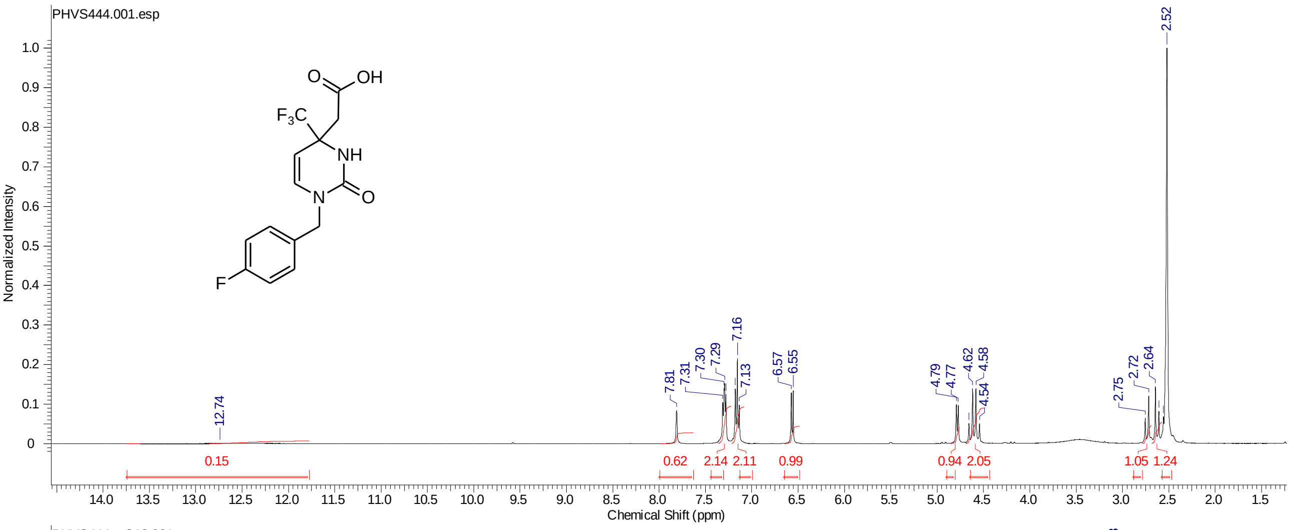
PHVS444.001.esp
Normalized Intensity
Chemical Shift (ppm)
12.74
7.81
7.31
7.30
7.29
7.16
7.13
6.57
6.55
4.79
4.77
4.62
4.58
4.54
2.75
2.72
2.64
2.52
0.15
0.62
2.14
2.11
0.99
0.94
2.05
1.05
1.24

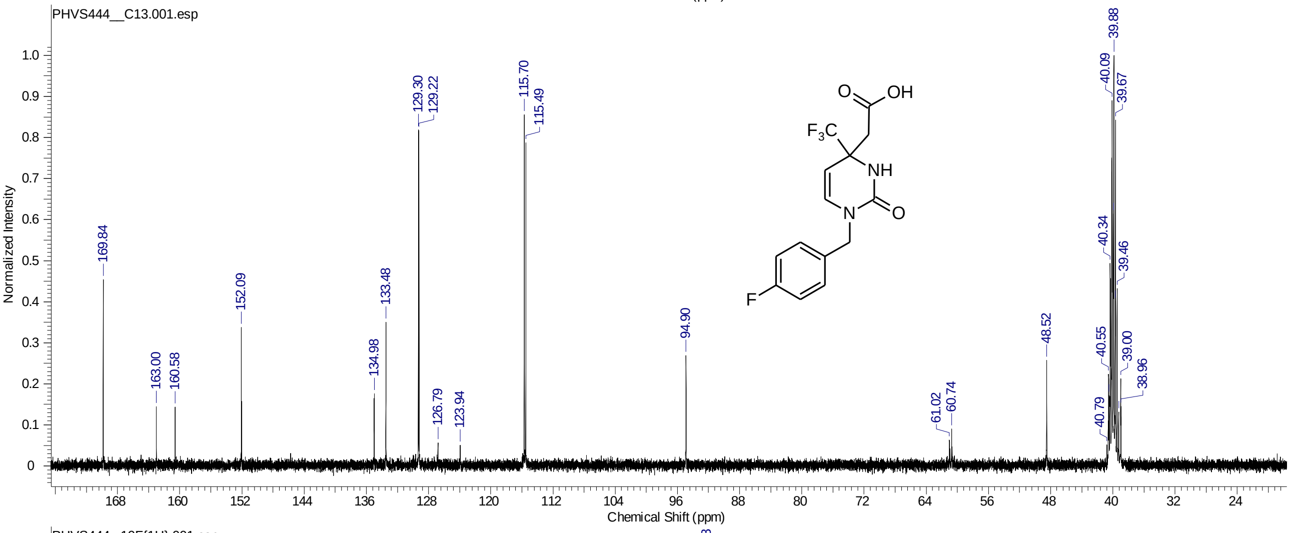
PHVS444__C13.001.esp
Normalized Intensity
Chemical Shift (ppm)
169.84
163.00
160.58
152.09
134.98
133.48
129.30
129.22
126.79
123.94
115.70
115.49
94.90
61.02
60.74
48.52
40.79
40.55
40.34
40.09
39.88
39.67
39.46
39.00
38.96

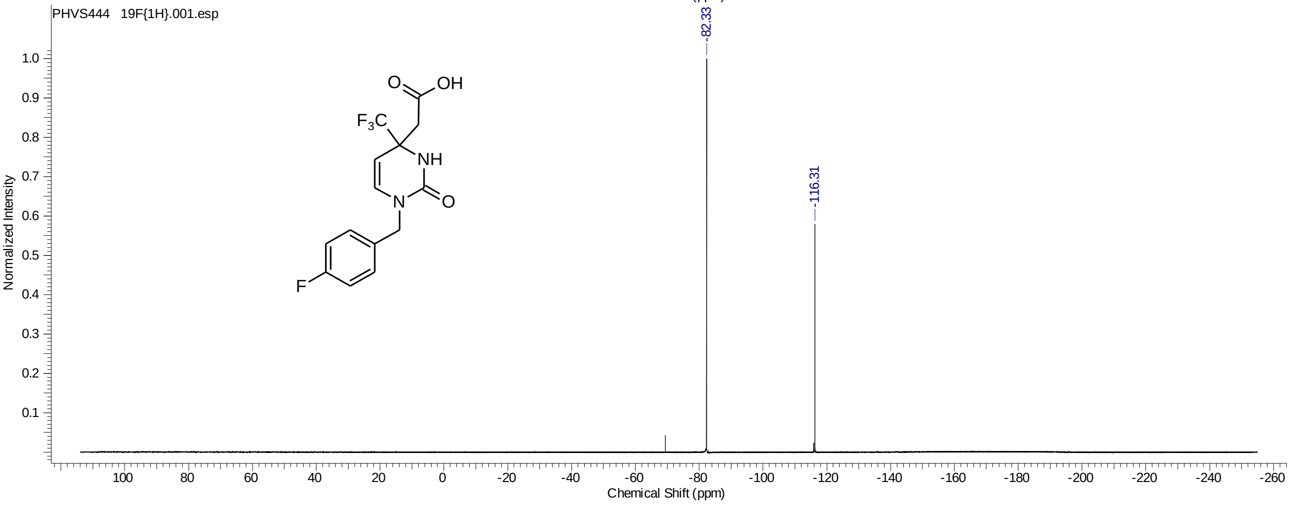
PHVS444 19F{1H}.001.esp
Normalized Intensity
Chemical Shift (ppm)
-82.33
-116.31

Compound **4i**

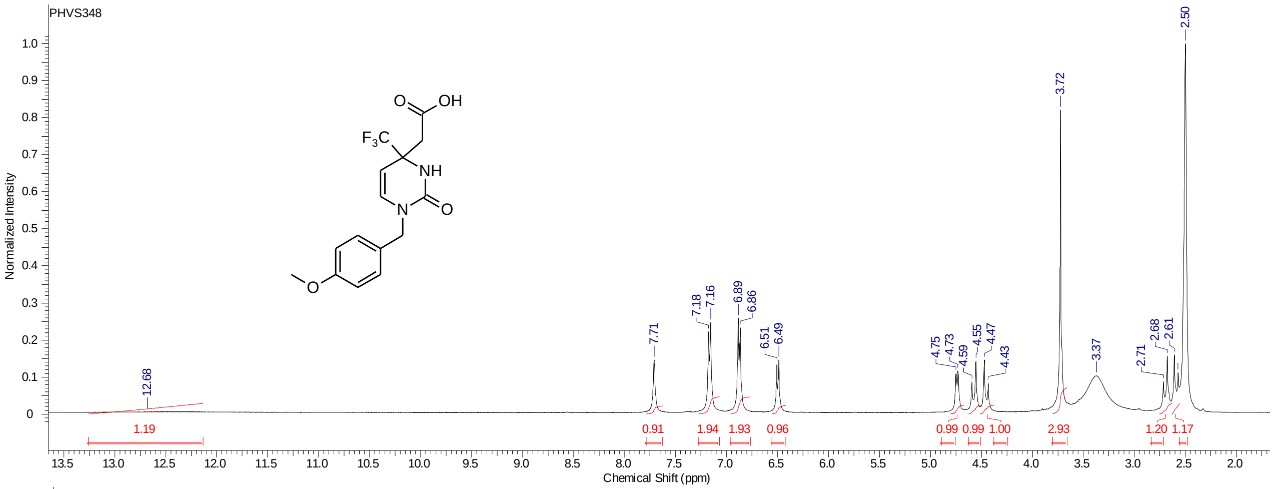

PHVS348
Normalized Intensity
Chemical Shift (ppm)
F3C
OH
O
NH
N
O
O
12.68
7.71
7.18
7.16
6.89
6.86
6.51
6.49
4.75
4.73
4.59
4.55
4.47
4.43
3.72
3.37
2.71
2.68
2.61
2.50
1.19
0.91
1.94
1.93
0.96
0.99
0.99
1.00
2.93
1.20
1.17

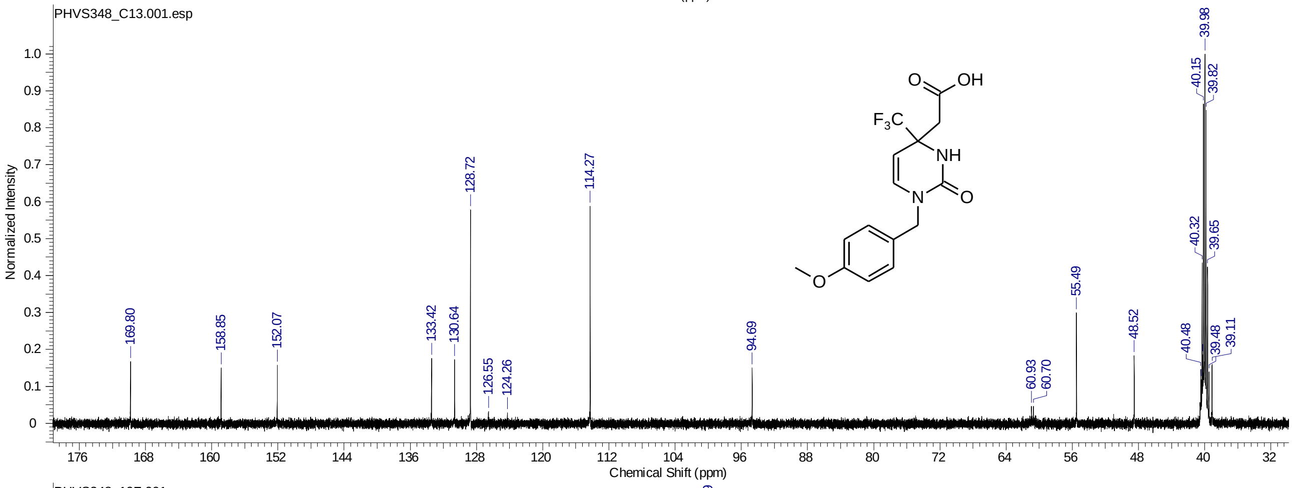

PHVS348_C13.001.esp
Normalized Intensity
Chemical Shift (ppm)
169.80
158.85
152.07
133.42
130.64
128.72
126.55
124.26
114.27
94.69
60.93
60.70
55.49
48.52
40.48
40.32
40.15
39.98
39.82
39.65
39.48
39.11

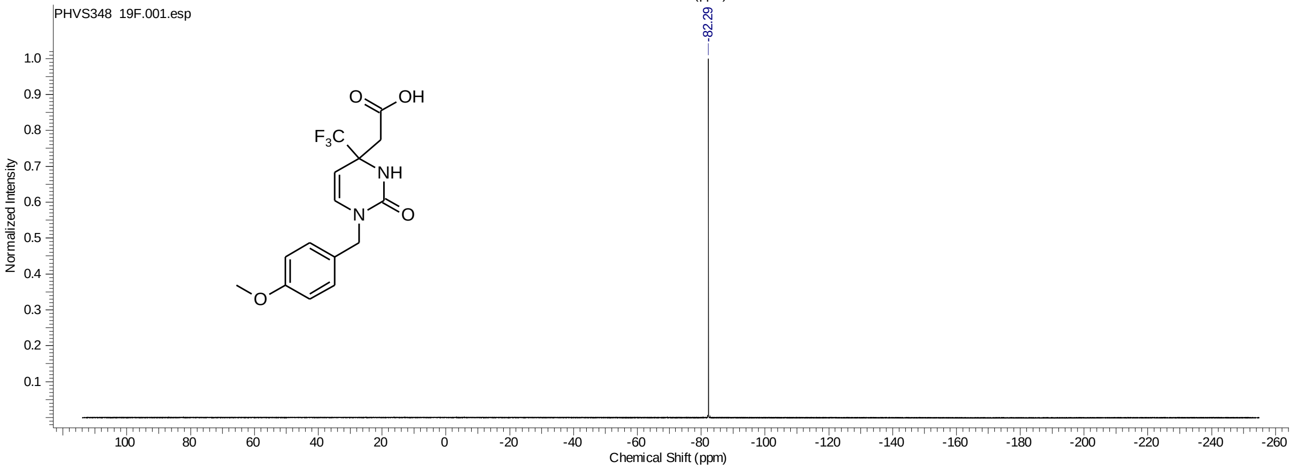

PHVS348 19F.001.esp
Normalized Intensity
Chemical Shift (ppm)
-82.29

Compound **4j**

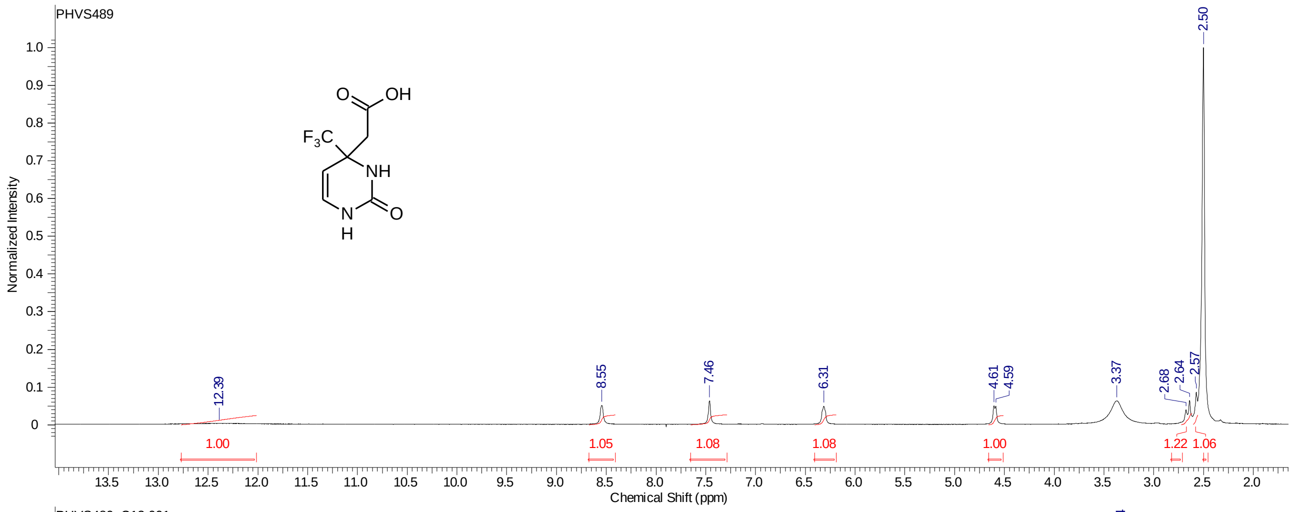
PHVS489
Normalized Intensity
Chemical Shift (ppm)
12.39
8.55
7.46
6.31
4.61
4.59
3.37
2.68
2.64
2.57
2.50
1.00
1.05
1.08
1.08
1.00
1.22
1.06
F3C
OH
O
NH
N
H
O

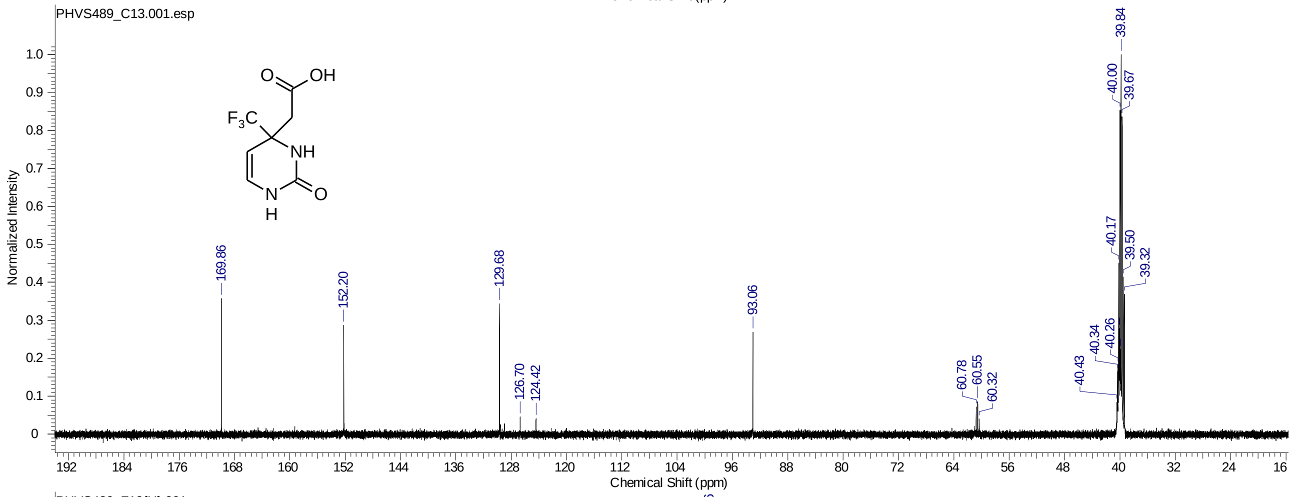
PHVS489_C13.001.esp
Normalized Intensity
Chemical Shift (ppm)
169.86
152.20
129.68
126.70
124.42
93.06
60.78
60.55
60.32
40.43
40.34
40.26
40.17
40.00
39.84
39.67
39.50
39.32

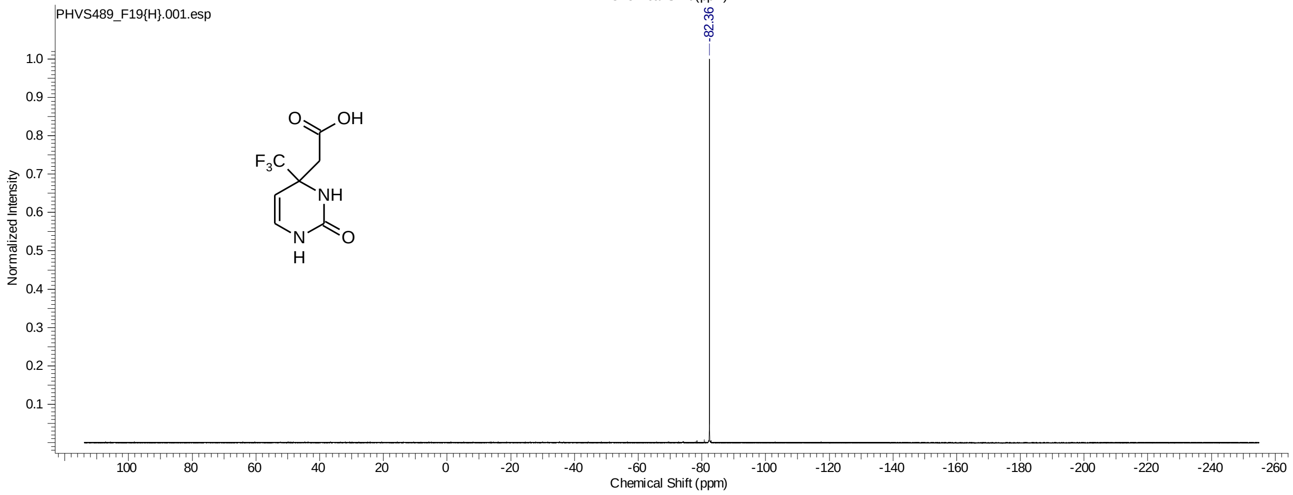
PHVS489_F19{H}.001.esp
Normalized Intensity
Chemical Shift (ppm)
-82.36

Compound **5a**

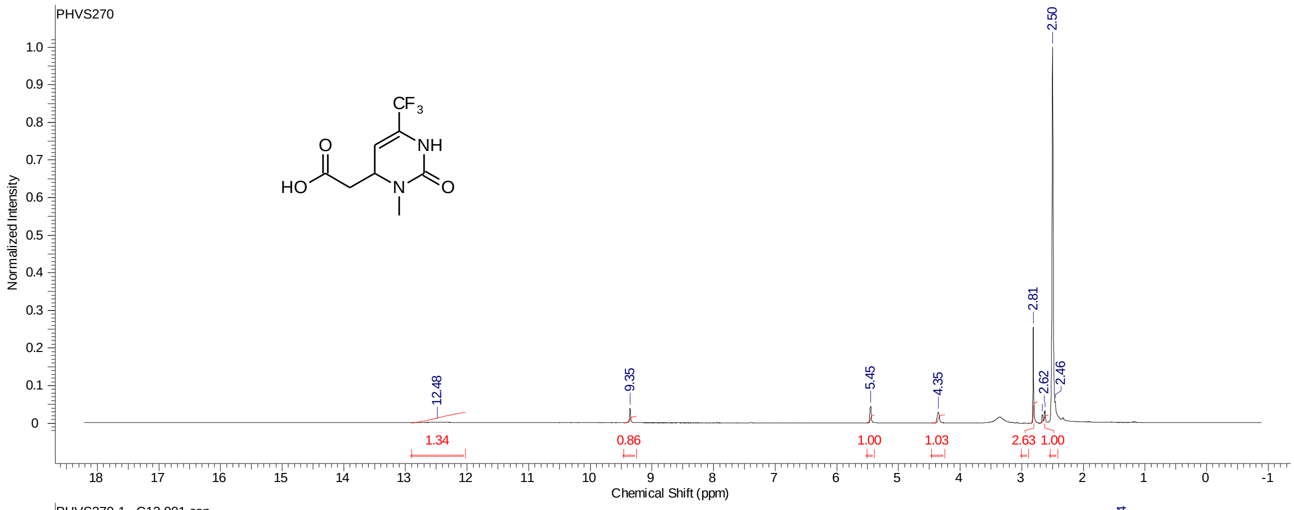
PHVS270
Normalized Intensity
Chemical Shift (ppm)
CF3
NH
O
HO
N
12.48
9.35
5.45
4.35
2.81
2.62
2.50
2.46
1.34
0.86
1.00
1.03
2.63
1.00

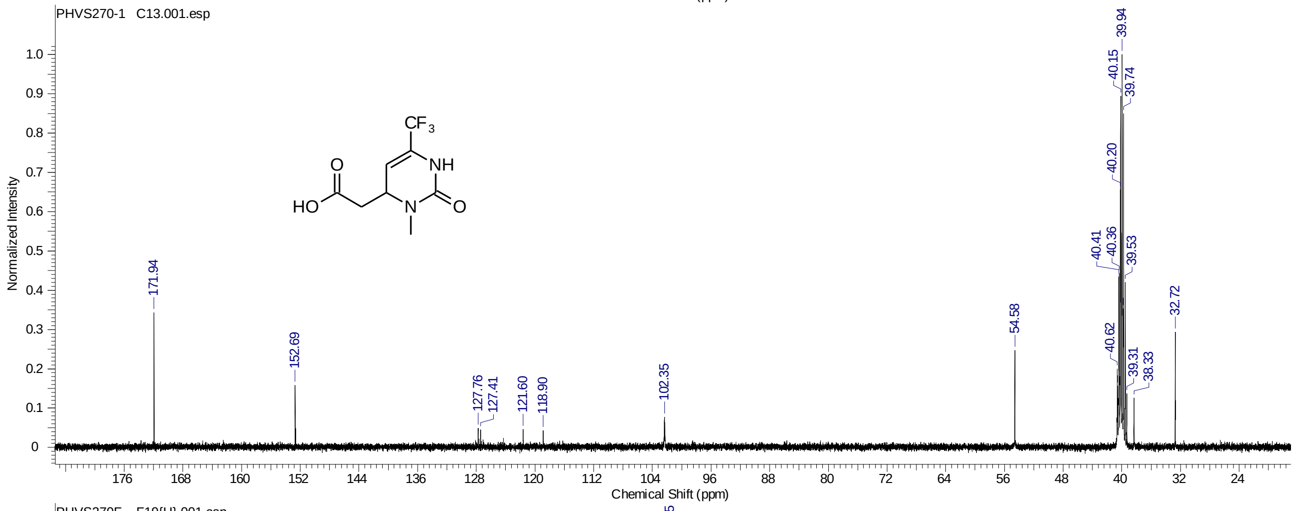
PHVS270-1 C13.001.esp
Normalized Intensity
Chemical Shift (ppm)
171.94
152.69
127.76
127.41
121.60
118.90
102.35
54.58
40.62
40.41
40.36
40.20
40.15
39.94
39.74
39.53
39.31
38.33
32.72

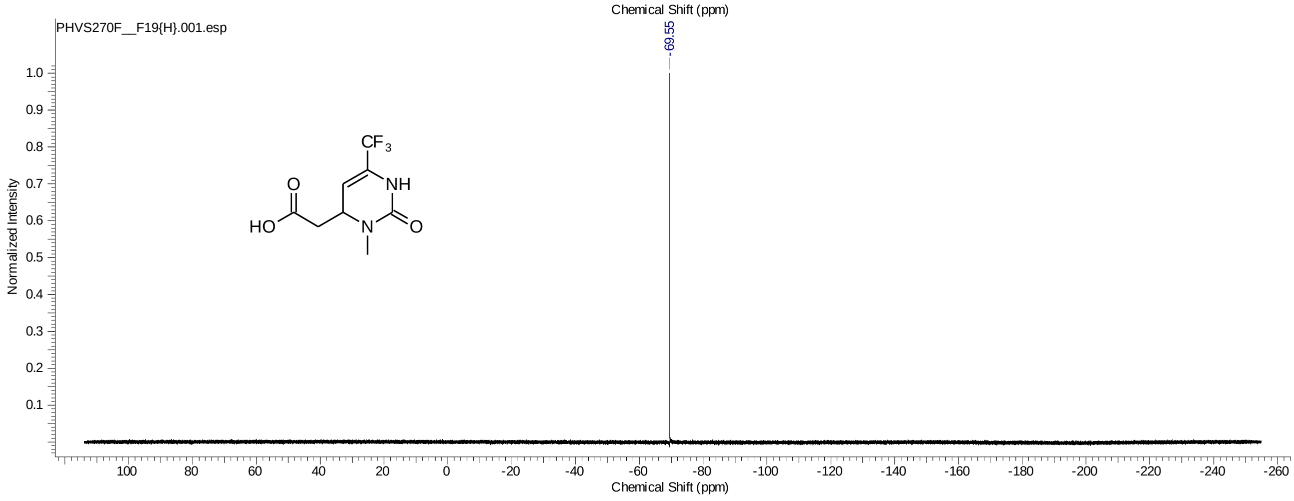
PHVS270F__F19{H}.001.esp
Normalized Intensity
Chemical Shift (ppm)
-69.55

Compound **5b**

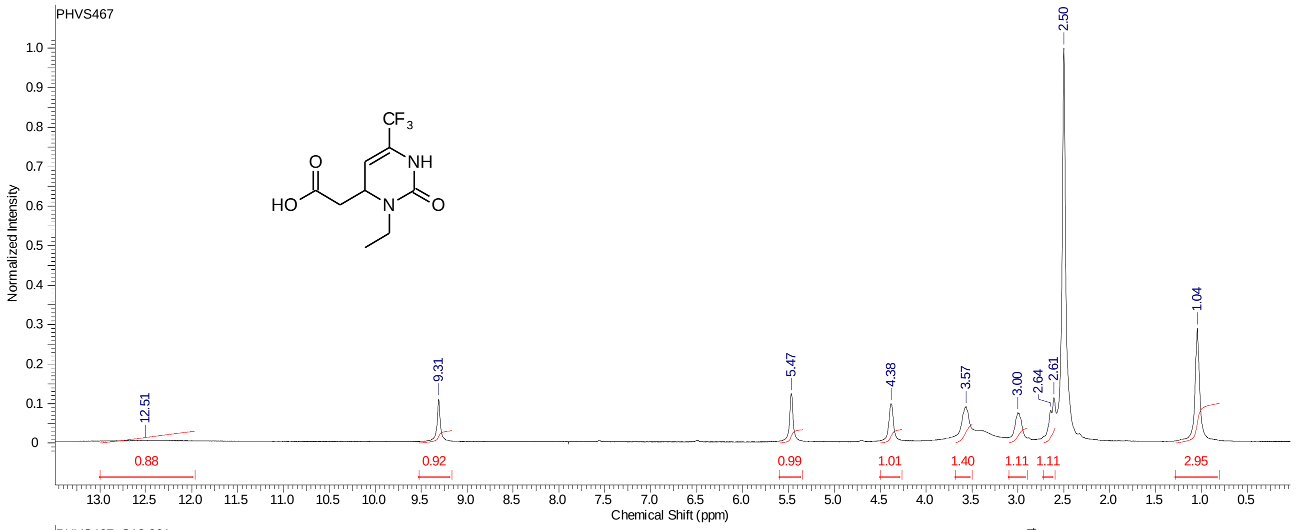
PHVS467
Normalized Intensity
Chemical Shift (ppm)
12.51
9.31
5.47
4.38
3.57
3.00
2.64
2.61
2.50
1.04
0.88
0.92
0.99
1.01
1.40
1.11
1.11
2.95

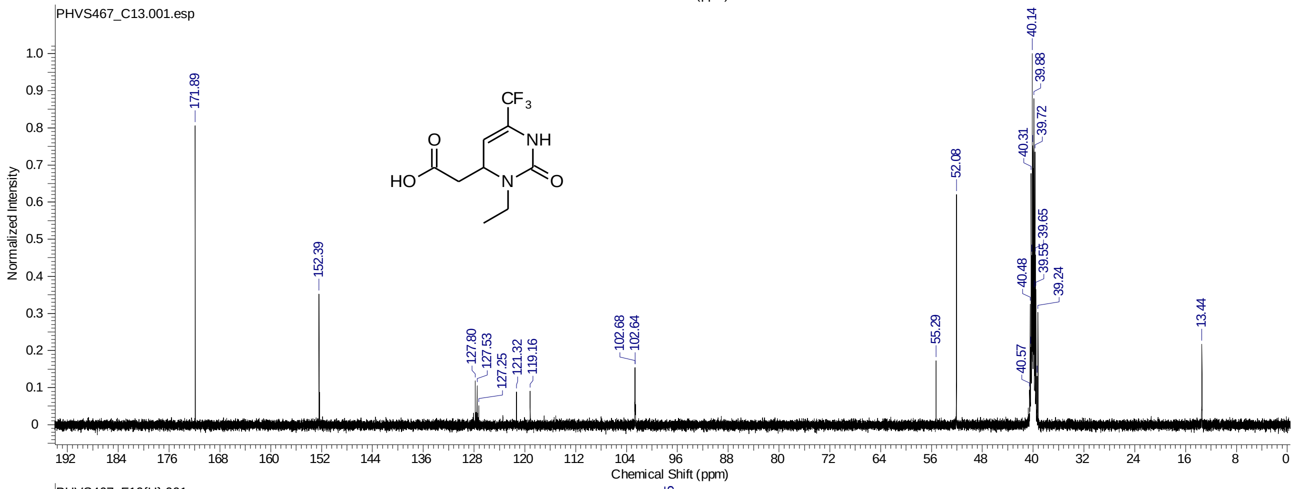
PHVS467_C13.001.esp
Normalized Intensity
Chemical Shift (ppm)
171.89
152.39
127.80
127.53
127.25
121.32
119.16
102.68
102.64
55.29
52.08
40.57
40.48
40.31
40.14
39.88
39.72
39.55 39.65
39.24
13.44

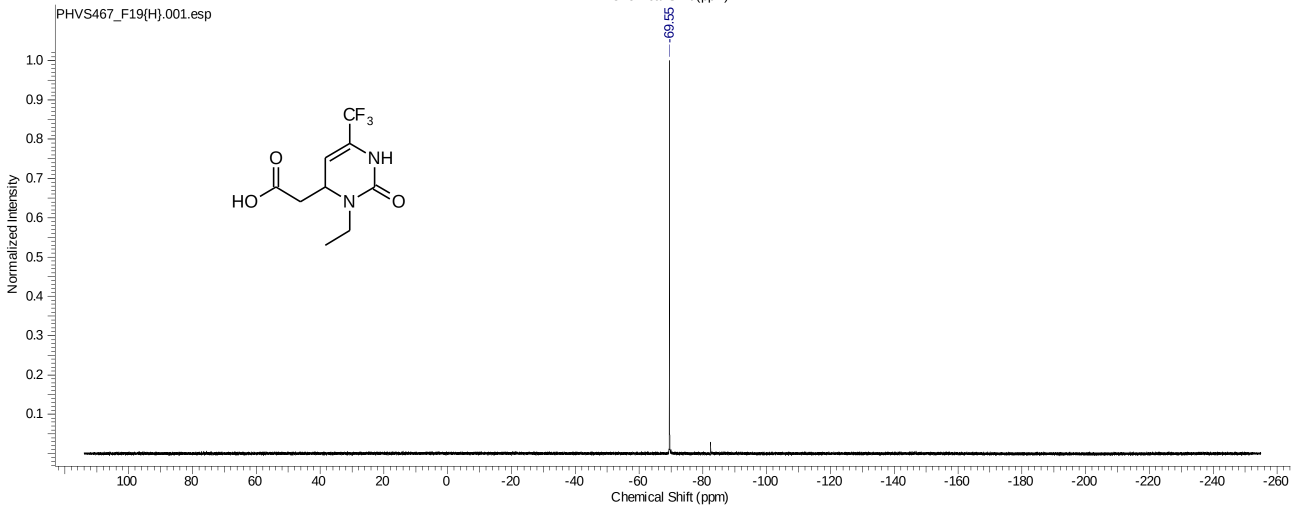
PHVS467_F19{H}.001.esp
Normalized Intensity
Chemical Shift (ppm)
-69.55

Compound **5c**

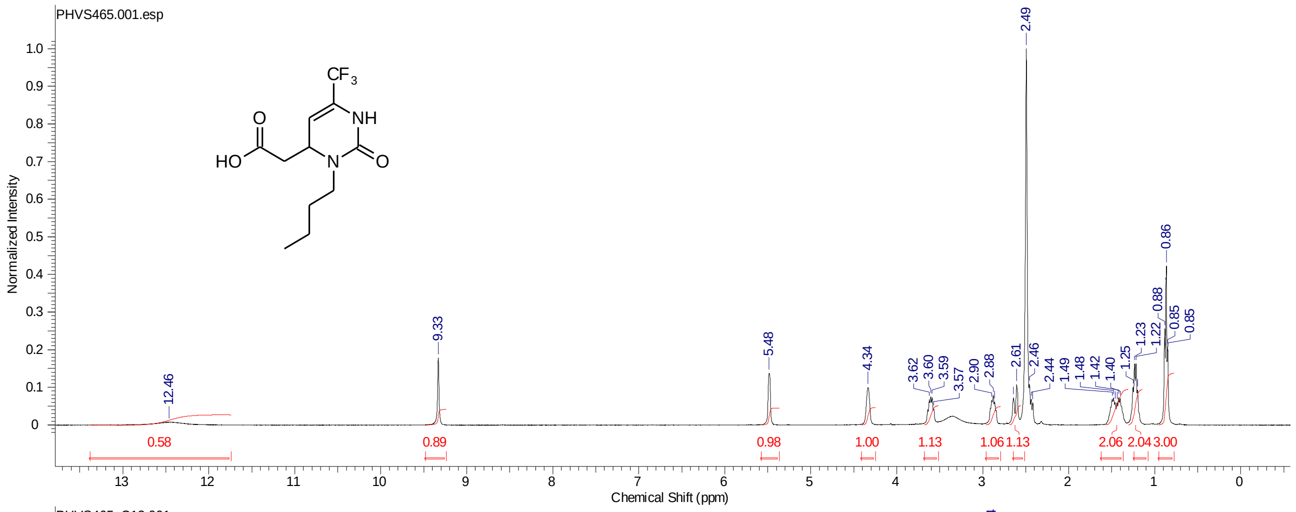
PHVS465.001.esp
Normalized Intensity
Chemical Shift (ppm)
12.46
9.33
5.48
4.34
3.62
3.60
3.59
3.57
2.90
2.88
2.61
2.49
2.46
2.44
1.49
1.48
1.42
1.40
1.25
1.23
1.22
0.88
0.86
0.85
0.85
0.58
0.89
0.98
1.00
1.13
1.06
1.13
2.06
2.04
3.00
CF3
NH
O
HO
N
O

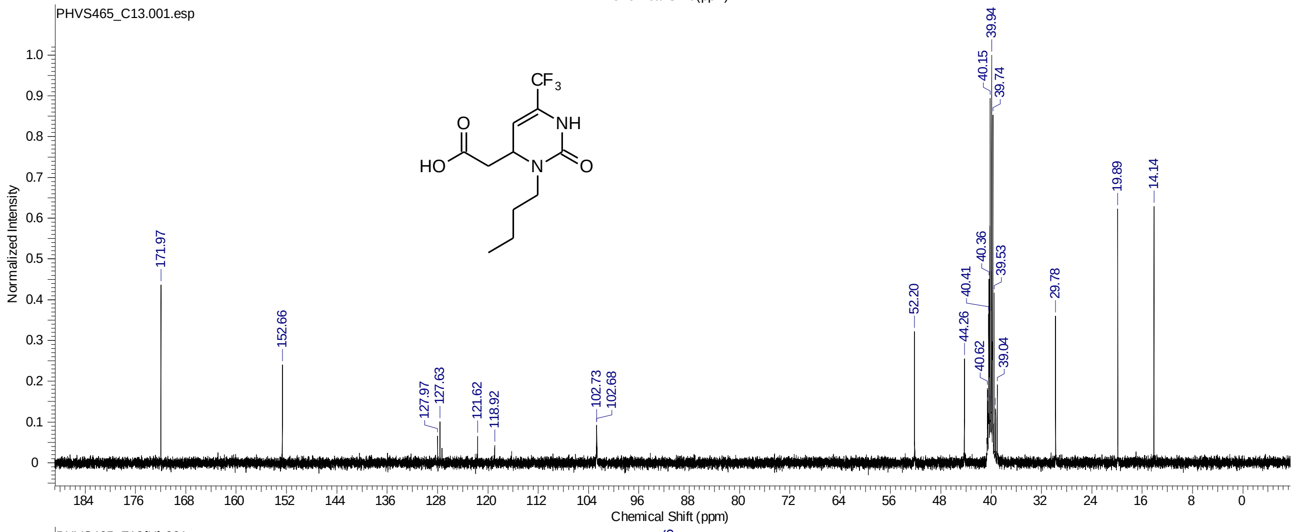
PHVS465_C13.001.esp
Normalized Intensity
Chemical Shift (ppm)
171.97
152.66
127.97
127.63
121.62
118.92
102.73
102.68
52.20
44.26
40.62
40.41
40.36
40.15
39.94
39.74
39.53
39.04
29.78
19.89
14.14

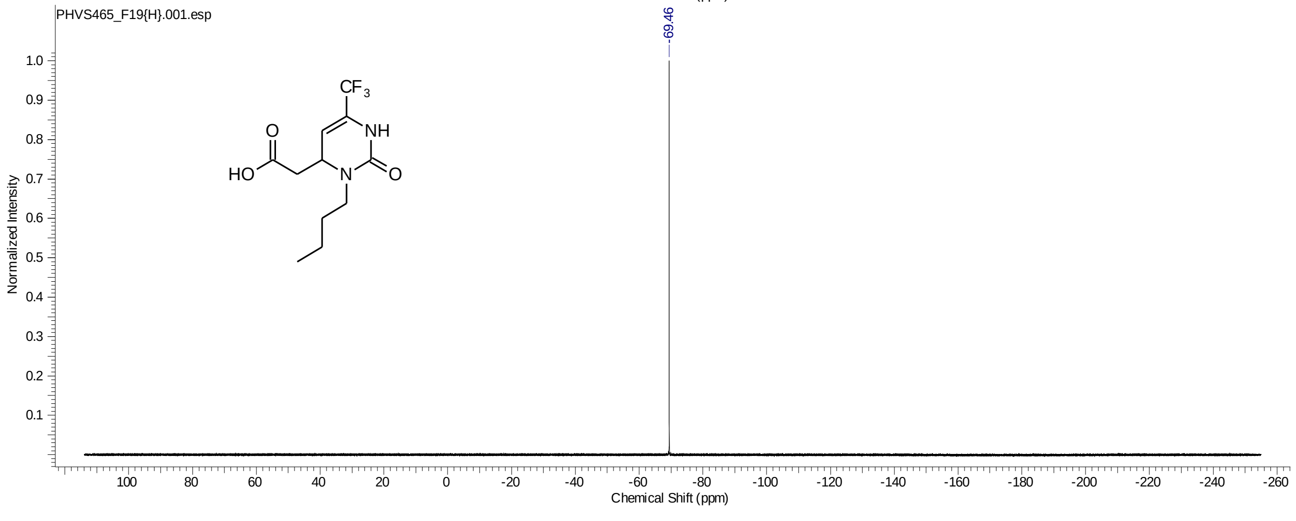
PHVS465_F19{H}.001.esp
Normalized Intensity
Chemical Shift (ppm)
-69.46

Compound **5d**

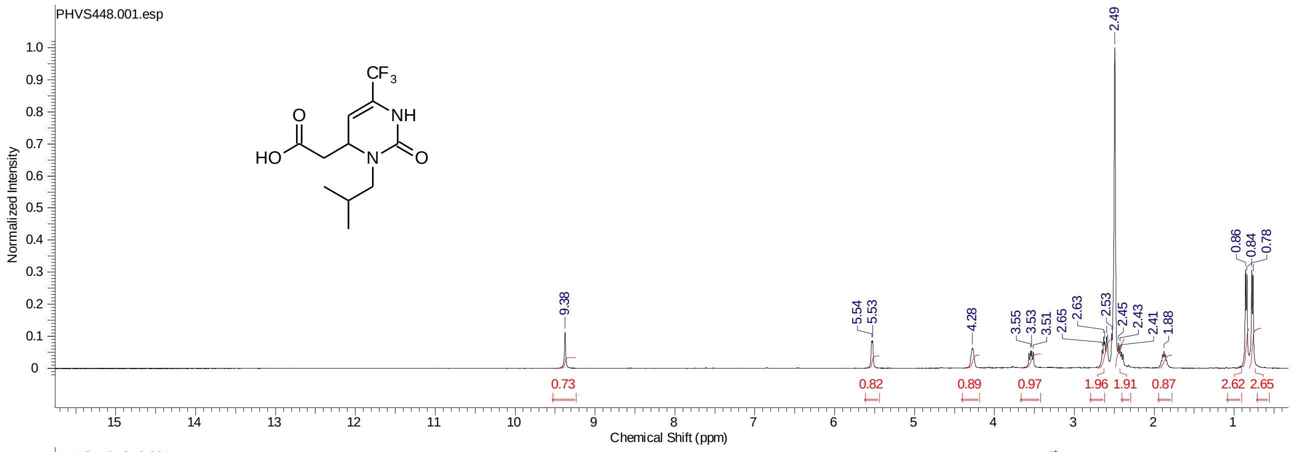
PHVS448.001.esp
Normalized Intensity
Chemical Shift (ppm)
CF3
NH
O
HO
N
O
9.38
5.54
5.53
4.28
3.55
3.53
3.51
2.65
2.63
2.53
2.49
2.45
2.43
2.41
1.88
0.86
0.84
0.78
0.73
0.82
0.89
0.97
1.96
1.91
0.87
2.62
2.65

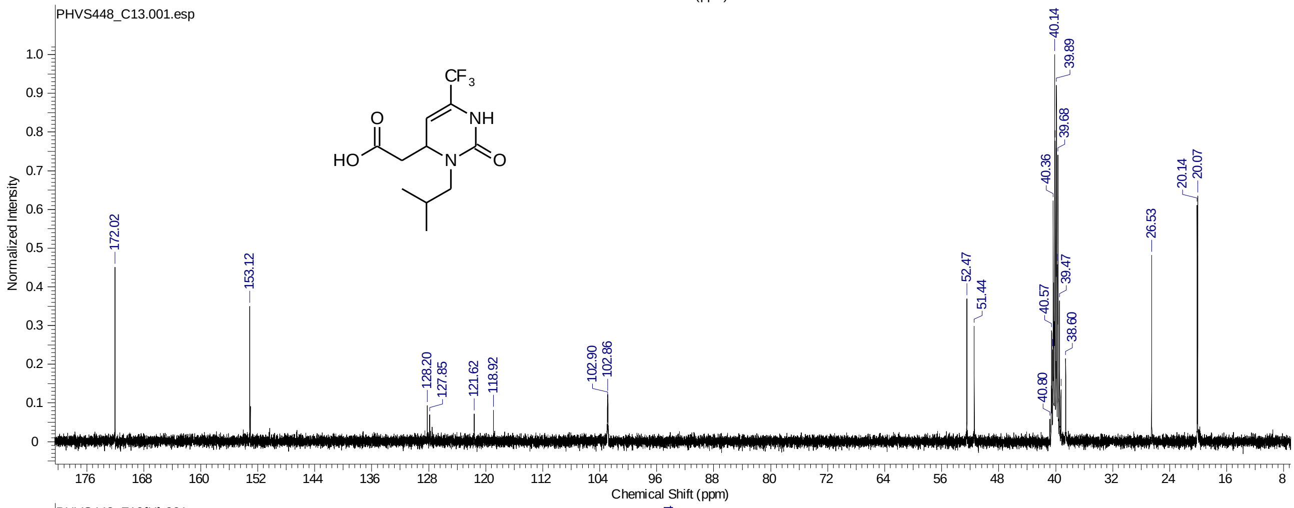
PHVS448_C13.001.esp
Normalized Intensity
Chemical Shift (ppm)
172.02
153.12
128.20
127.85
121.62
118.92
102.90
102.86
52.47
51.44
40.80
40.57
40.36
40.14
39.89
39.68
39.47
38.60
26.53
20.14
20.07

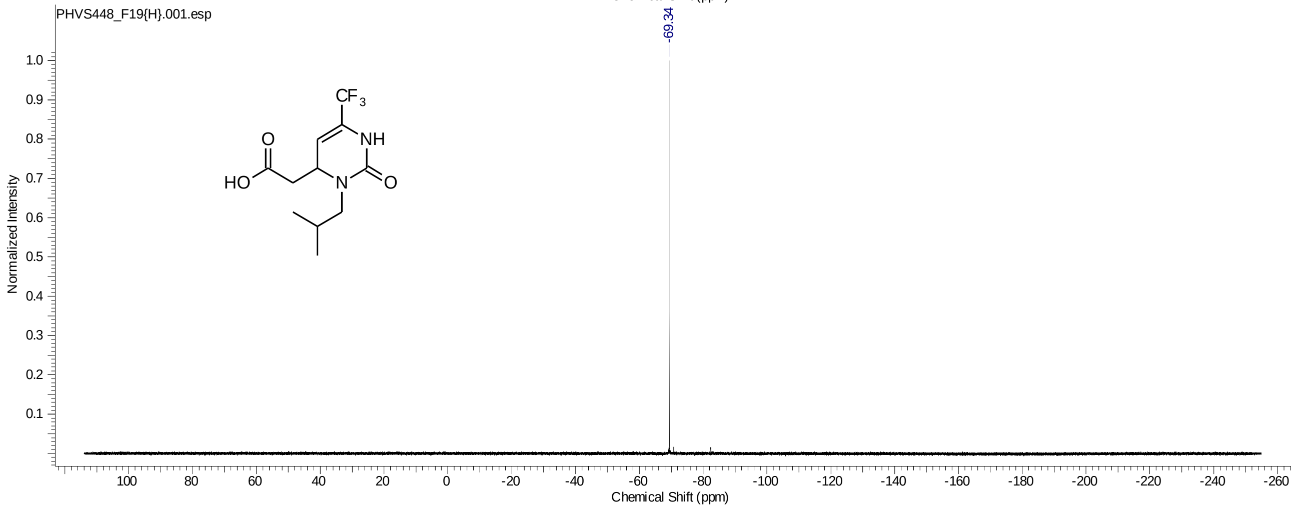
PHVS448_F19{H}.001.esp
Normalized Intensity
Chemical Shift (ppm)
-69.34

Compound **5e**

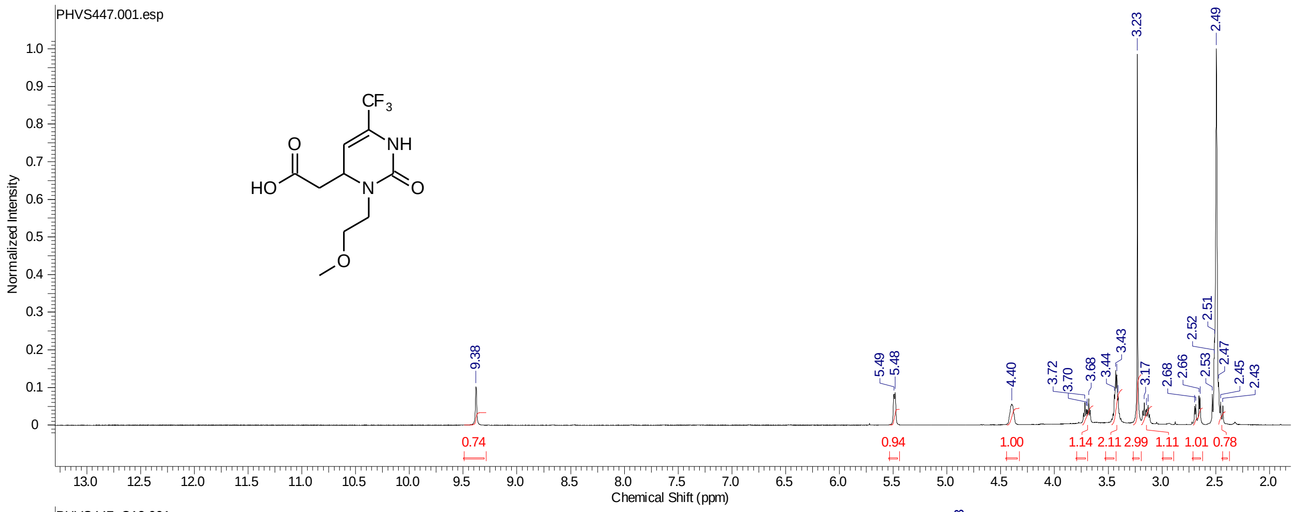
PHVS447.001.esp
Normalized Intensity
Chemical Shift (ppm)
9.38
5.49
5.48
4.40
3.72
3.70
3.68
3.44
3.43
3.23
3.17
2.68
2.66
2.53
2.52
2.51
2.49
2.47
2.45
2.43
0.74
0.94
1.00
1.14
2.11
2.99
1.11
1.01
0.78
CF3
NH
O
HO
N
O
O

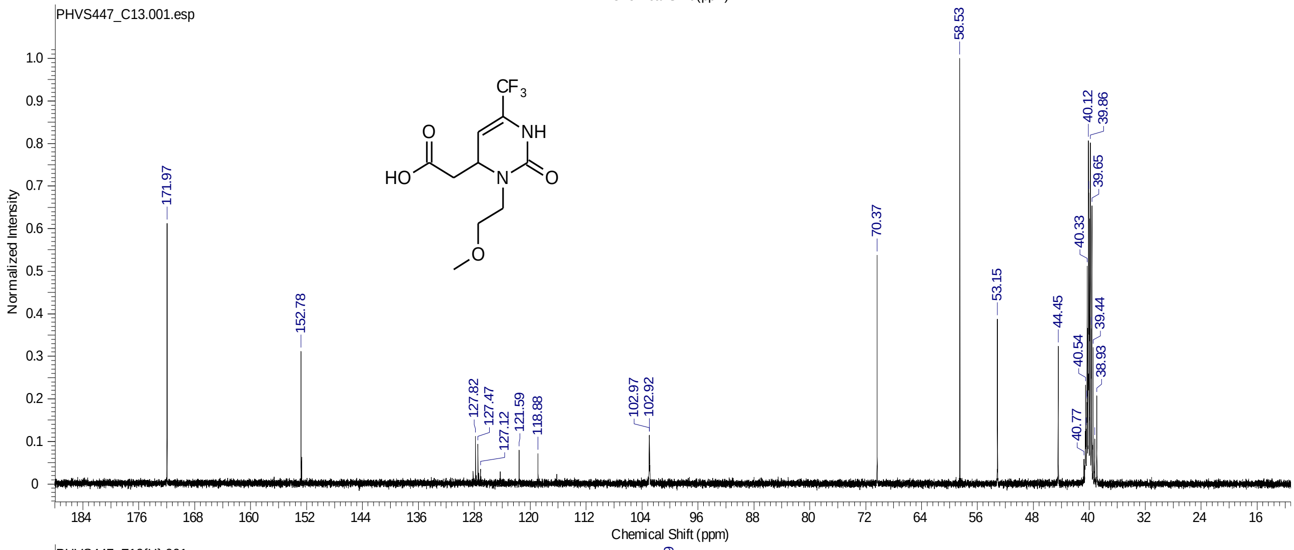
PHVS447_C13.001.esp
Normalized Intensity
Chemical Shift (ppm)
171.97
152.78
127.82
127.47
127.12
121.59
118.88
102.97
102.92
70.37
58.53
53.15
44.45
40.77
40.54
40.33
40.12
39.86
39.65
39.44
38.93
CF3
NH
O
HO
N
O
O

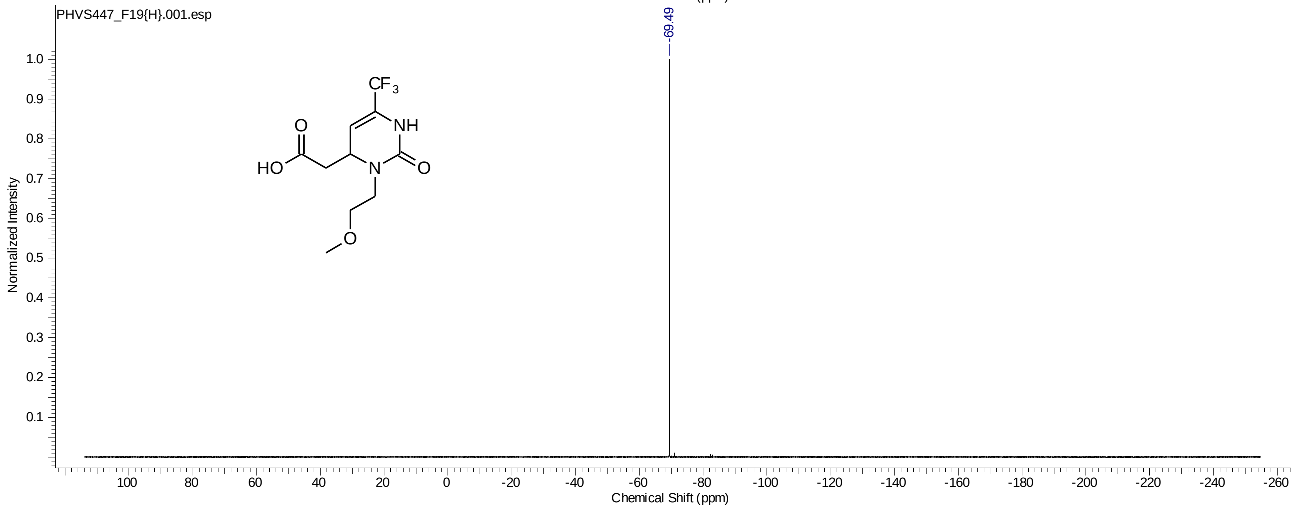
PHVS447_F19{H}.001.esp
Normalized Intensity
Chemical Shift (ppm)
-69.49
CF3
NH
O
HO
N
O
O

Compound **5f**

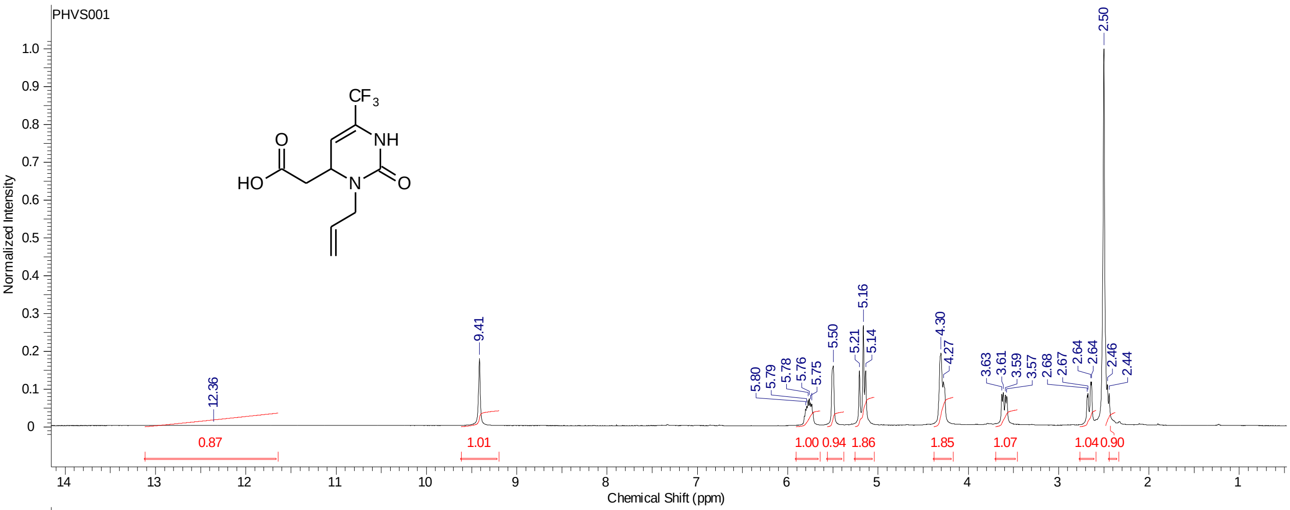

PHVS001
Normalized Intensity
Chemical Shift (ppm)
HO
O
CF3
NH
N
O
12.36
9.41
5.80
5.79
5.78
5.76
5.75
5.50
5.21
5.16
5.14
4.30
4.27
3.63
3.61
3.59
3.57
2.68
2.67
2.64
2.64
2.50
2.46
2.44
0.87
1.01
1.00
0.94
1.86
1.85
1.07
1.04
0.90

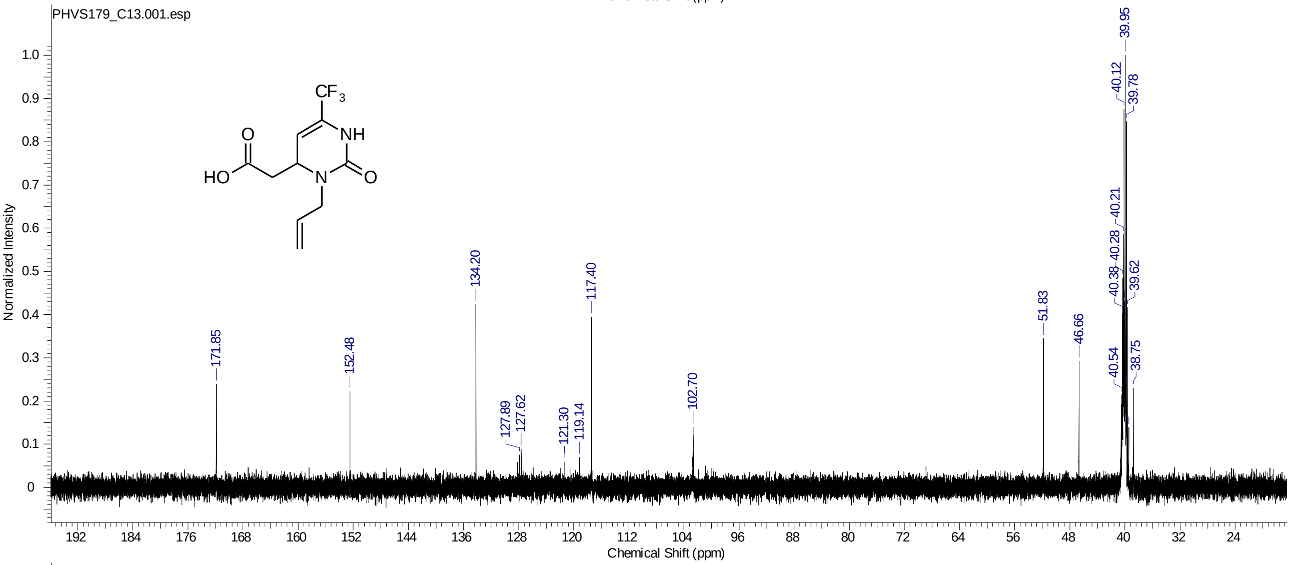

PHVS179_C13.001.esp
Normalized Intensity
Chemical Shift (ppm)
HO
O
CF3
NH
N
O
171.85
152.48
134.20
127.89
127.62
121.30
119.14
117.40
102.70
51.83
46.66
40.54
40.38
40.28
40.21
40.12
39.95
39.78
39.62
38.75

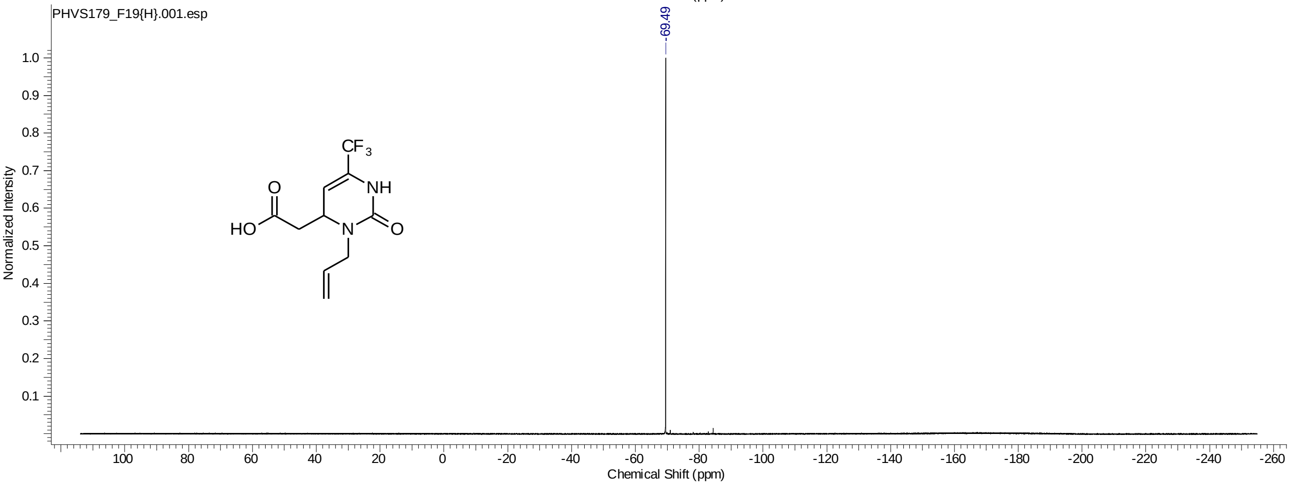

PHVS179_F19{H}.001.esp
Normalized Intensity
Chemical Shift (ppm)
HO
O
CF3
NH
N
O
-69.49

Compound **5g**

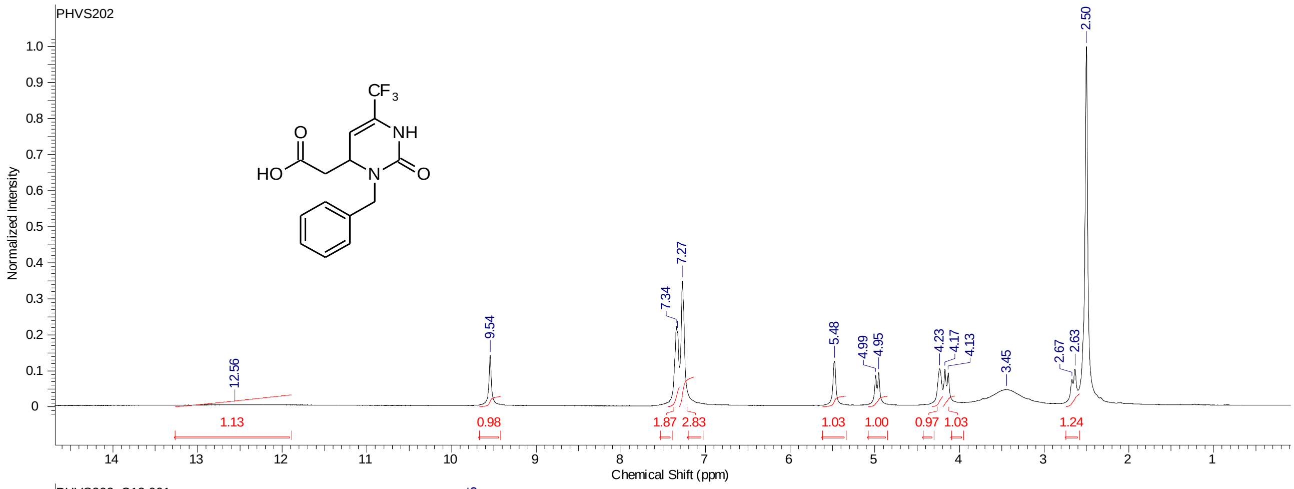
PHVS202
Normalized Intensity
Chemical Shift (ppm)
12.56
9.54
7.34
7.27
5.48
4.99
4.95
4.23
4.17
4.13
3.45
2.67
2.63
2.50
1.13
0.98
1.87
2.83
1.03
1.00
0.97
1.03
1.24
CF3
NH
O
HO
N
O

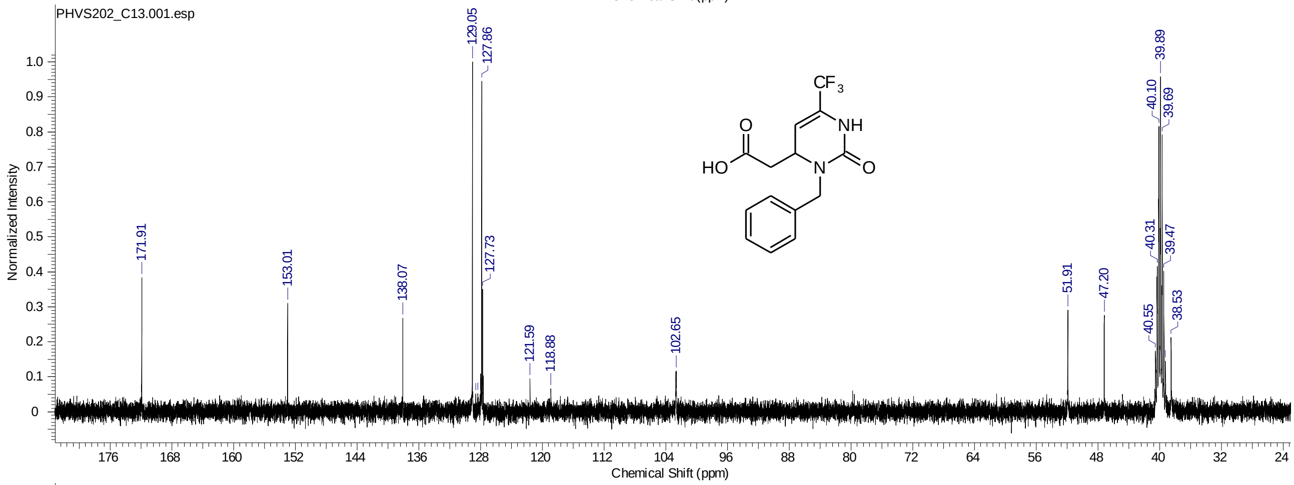
PHVS202_C13.001.esp
Normalized Intensity
Chemical Shift (ppm)
171.91
153.01
138.07
129.05
127.86
127.73
121.59
118.88
102.65
51.91
47.20
40.55
40.31
40.10
39.89
39.69
39.47
38.53
CF3
NH
O
HO
N
O

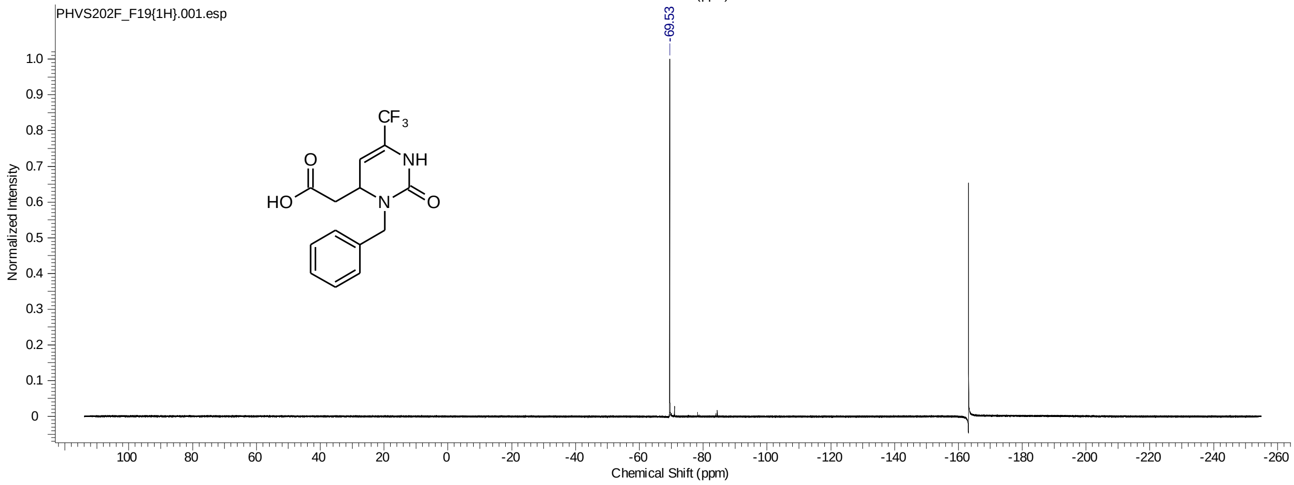
PHVS202F_F19{1H}.001.esp
Normalized Intensity
Chemical Shift (ppm)
-69.53
CF3
NH
O
HO
N
O

Compound **5h**

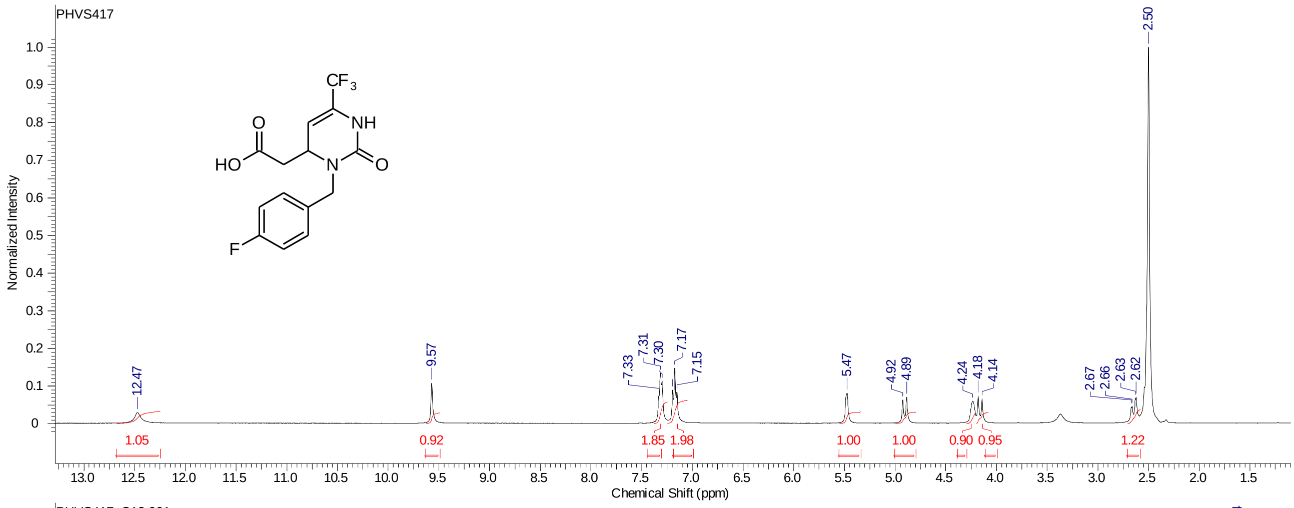
PHVS417
CF3
NH
O
HO
N
O
F
12.47
9.57
7.33
7.31
7.30
7.17
7.15
5.47
4.92
4.89
4.24
4.18
4.14
2.67
2.66
2.63
2.62
2.50
1.05
0.92
1.85
1.98
1.00
1.00
0.90
0.95
1.22
Normalized Intensity
Chemical Shift (ppm)

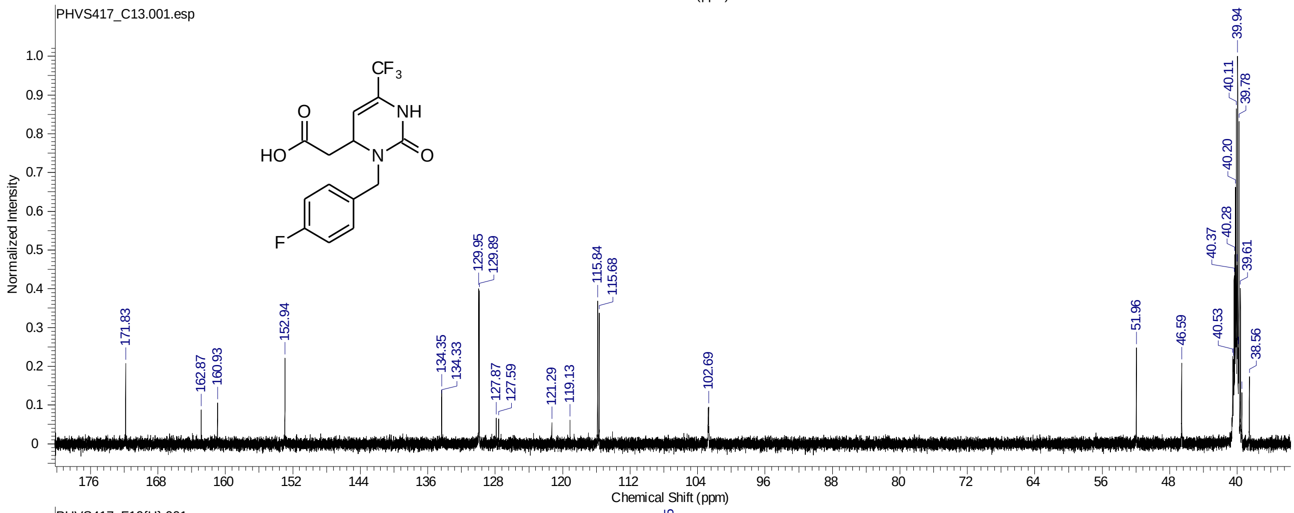
PHVS417_C13.001.esp
CF3
NH
O
HO
N
O
F
171.83
162.87
160.93
152.94
134.35
134.33
129.95
129.89
127.87
127.59
121.29
119.13
115.84
115.68
102.69
51.96
46.59
40.53
40.37
40.28
40.20
40.11
39.94
39.78
39.61
38.56
Normalized Intensity
Chemical Shift (ppm)

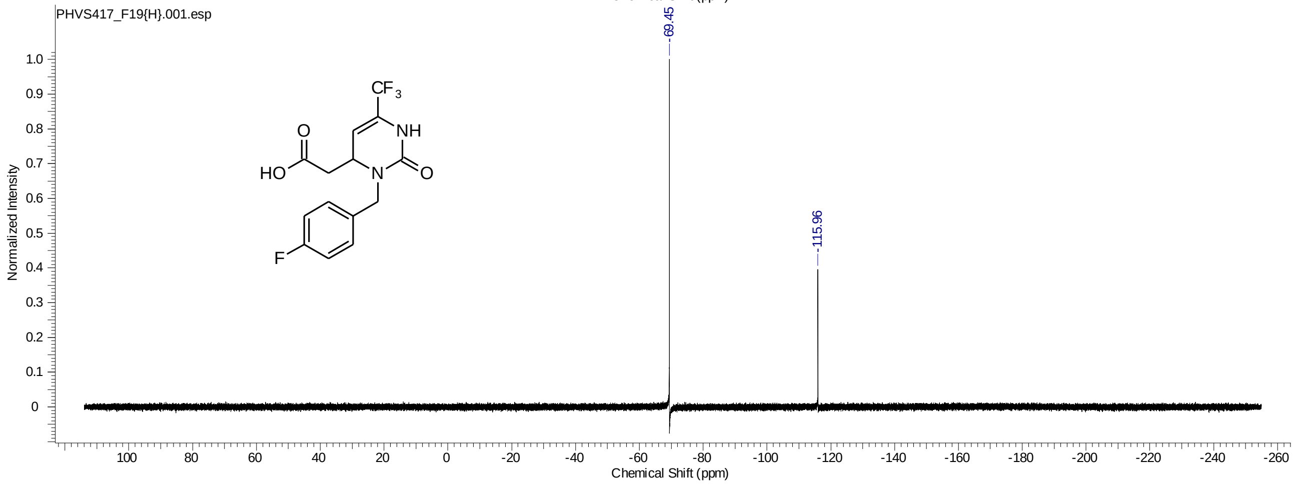
PHVS417_F19{H}.001.esp
CF3
NH
O
HO
N
O
F
-69.45
-115.96
Normalized Intensity
Chemical Shift (ppm)

Compound **5i**

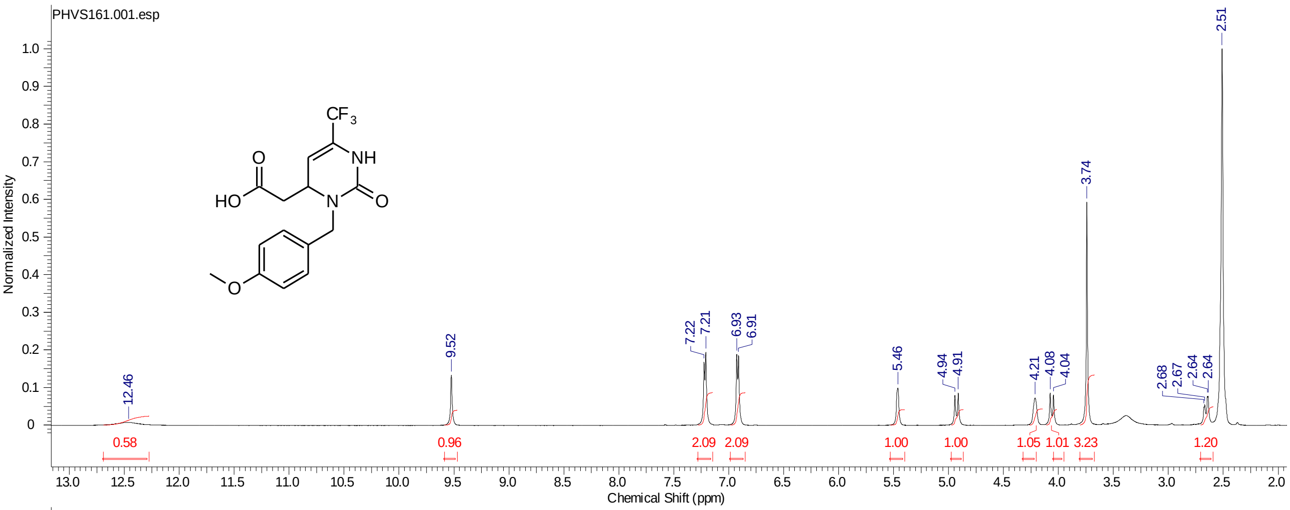
PHVS161.001.esp
CF3
NH
O
HO
N
O
O
Normalized Intensity
Chemical Shift (ppm)
12.46
9.52
7.22
7.21
6.93
6.91
5.46
4.94
4.91
4.21
4.08
4.04
3.74
2.68
2.67
2.64
2.64
2.51
0.58
0.96
2.09
2.09
1.00
1.00
1.05
1.01
3.23
1.20

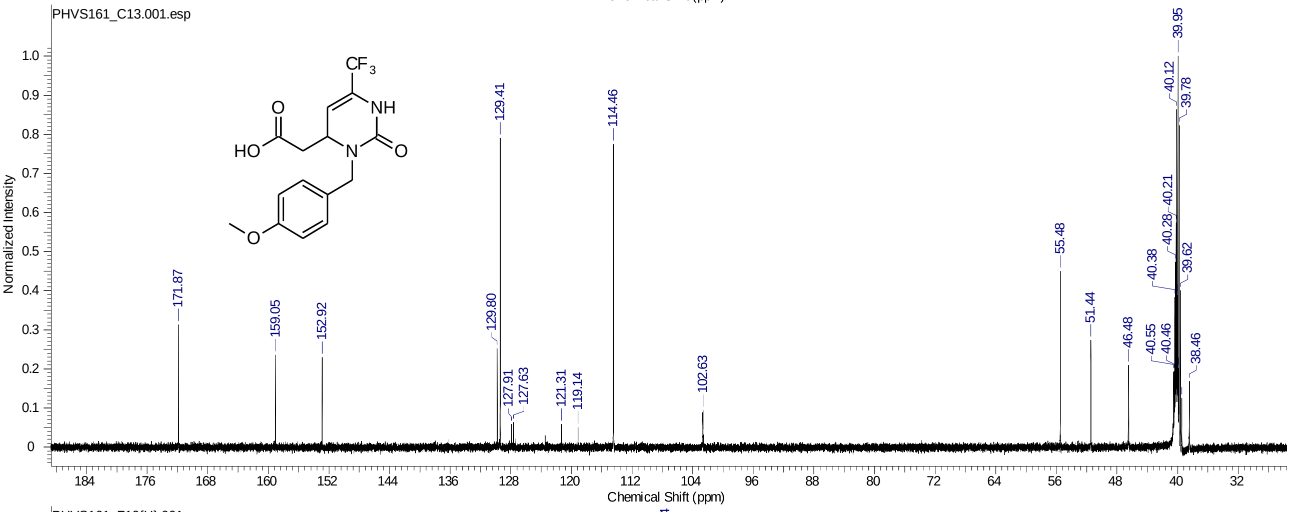
PHVS161_C13.001.esp
Normalized Intensity
Chemical Shift (ppm)
171.87
159.05
152.92
129.80
129.41
127.91
127.63
121.31
119.14
114.46
102.63
55.48
51.44
46.48
40.55
40.46
40.38
40.28
40.21
40.12
39.95
39.78
39.62
38.46

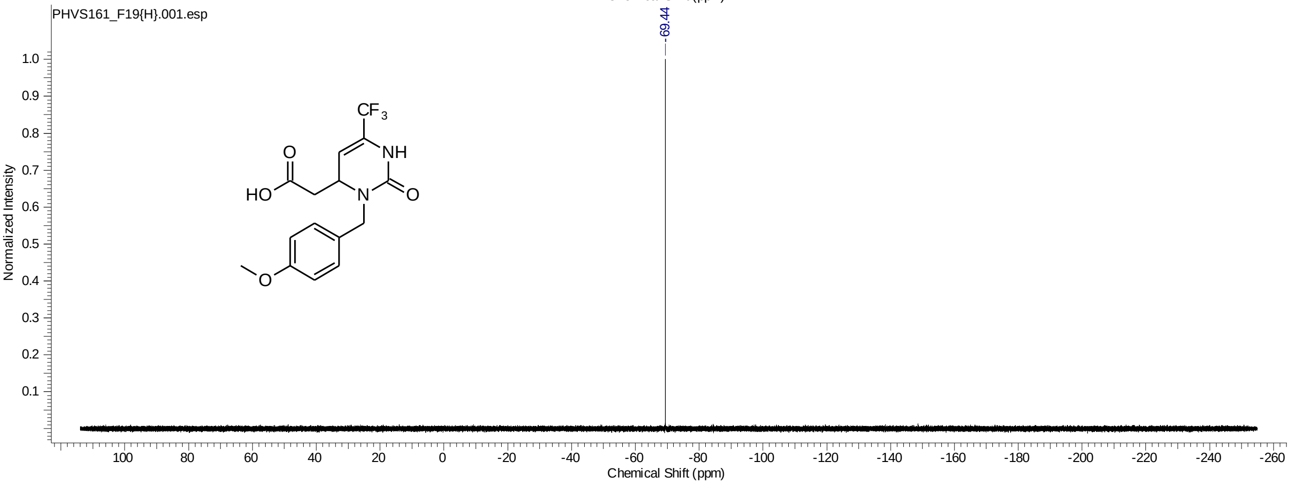
PHVS161_F19{H}.001.esp
Normalized Intensity
Chemical Shift (ppm)
-69.44

Compound **5j**

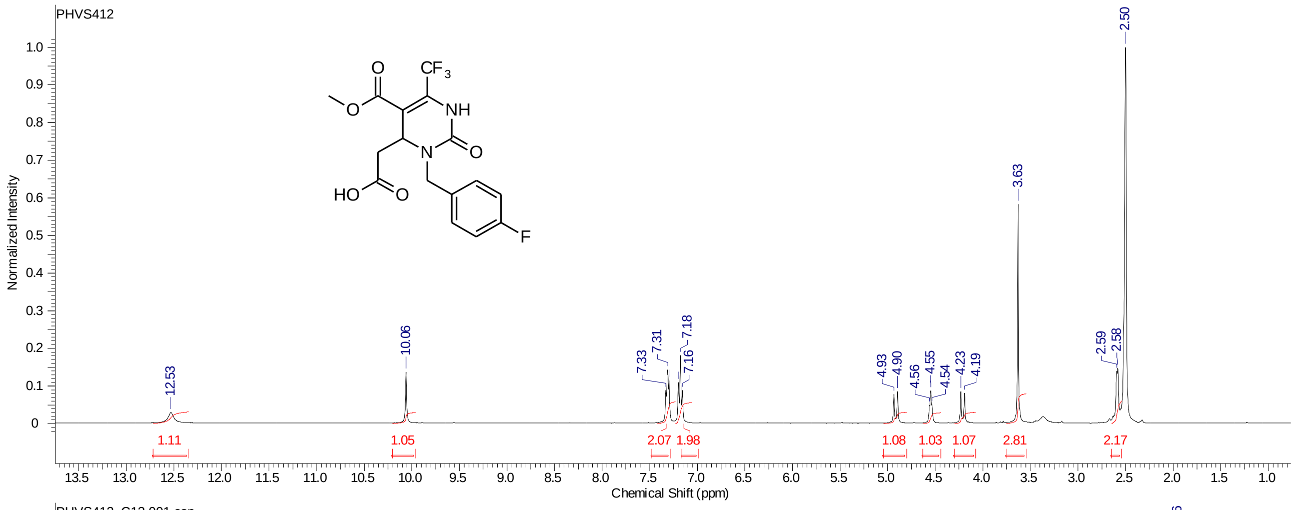
PHVS412
Normalized Intensity
Chemical Shift (ppm)
12.53
10.06
7.33
7.31
7.18
7.16
4.93
4.90
4.56
4.55
4.54
4.23
4.19
3.63
2.59
2.58
2.50
1.11
1.05
2.07
1.98
1.08
1.03
1.07
2.81
2.17

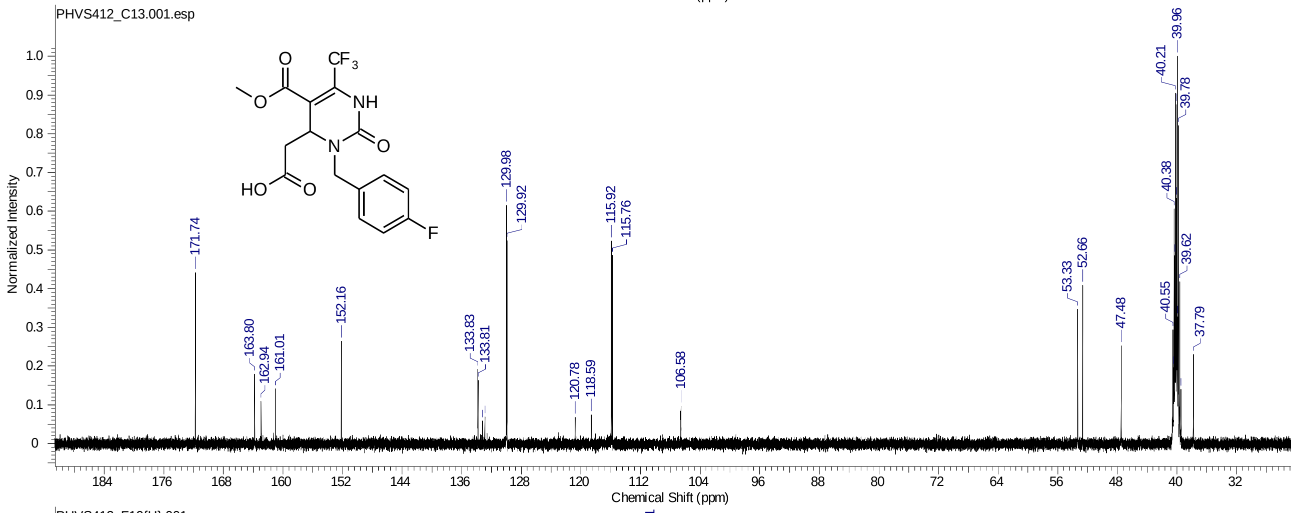
PHVS412_C13.001.esp
Normalized Intensity
Chemical Shift (ppm)
171.74
163.80
162.94
161.01
152.16
133.83
133.81
129.98
129.92
120.78
118.59
115.92
115.76
106.58
53.33
52.66
47.48
40.55
40.38
40.21
39.96
39.78
39.62
37.79

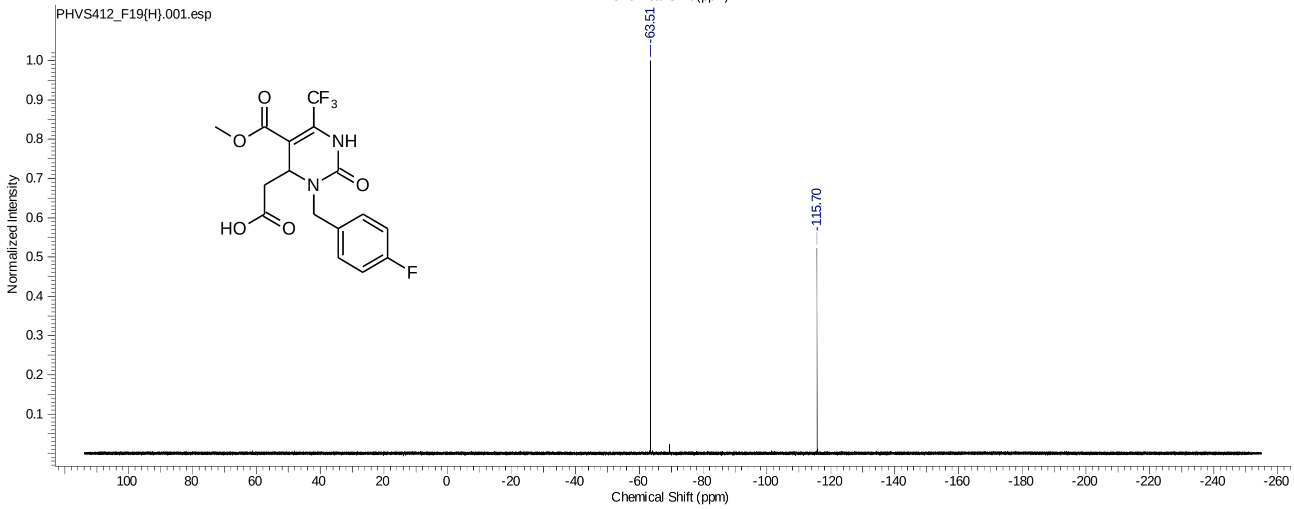
PHVS412_F19{H}.001.esp
Normalized Intensity
Chemical Shift (ppm)
-63.51
-115.70

Compound **5k**

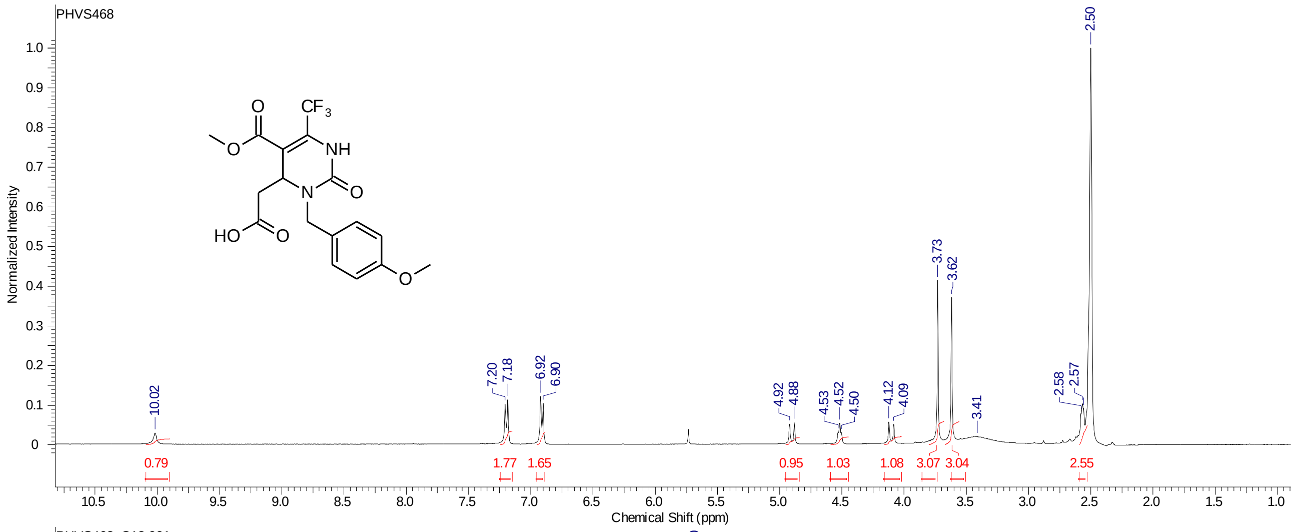
PHVS468
Normalized Intensity
Chemical Shift (ppm)

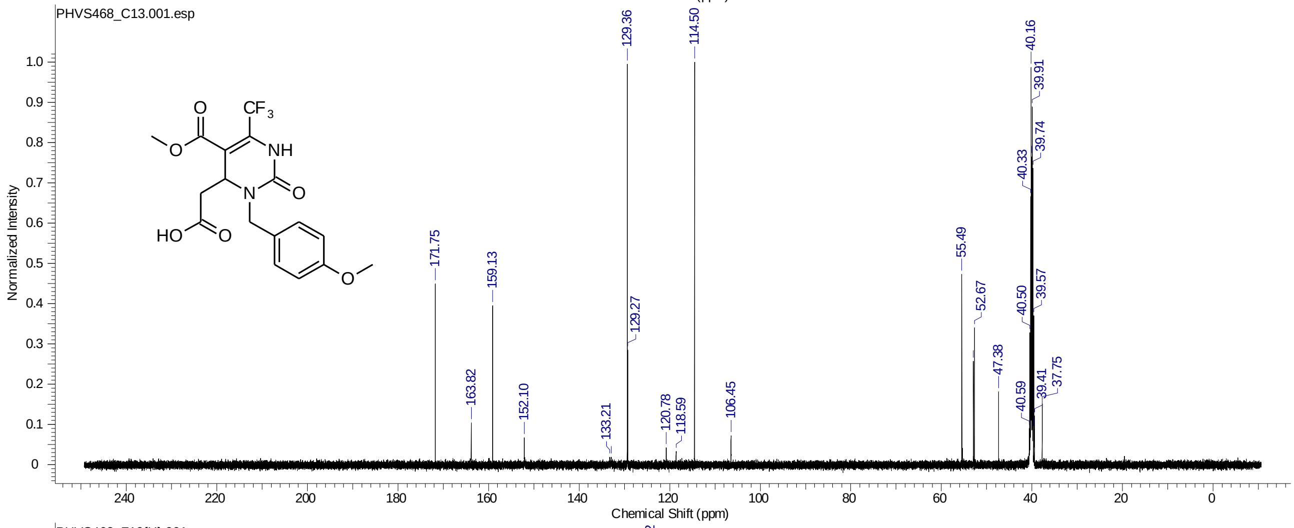
PHVS468_C13.001.esp
Normalized Intensity
Chemical Shift (ppm)

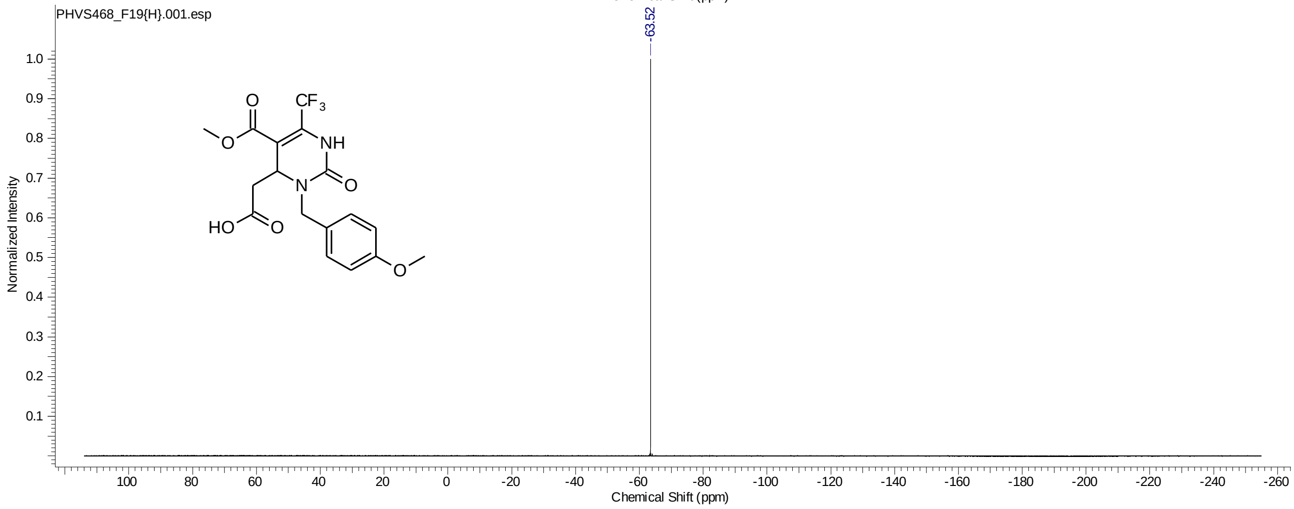
PHVS468_F19{H}.001.esp
Normalized Intensity
Chemical Shift (ppm)

Compound **5l**

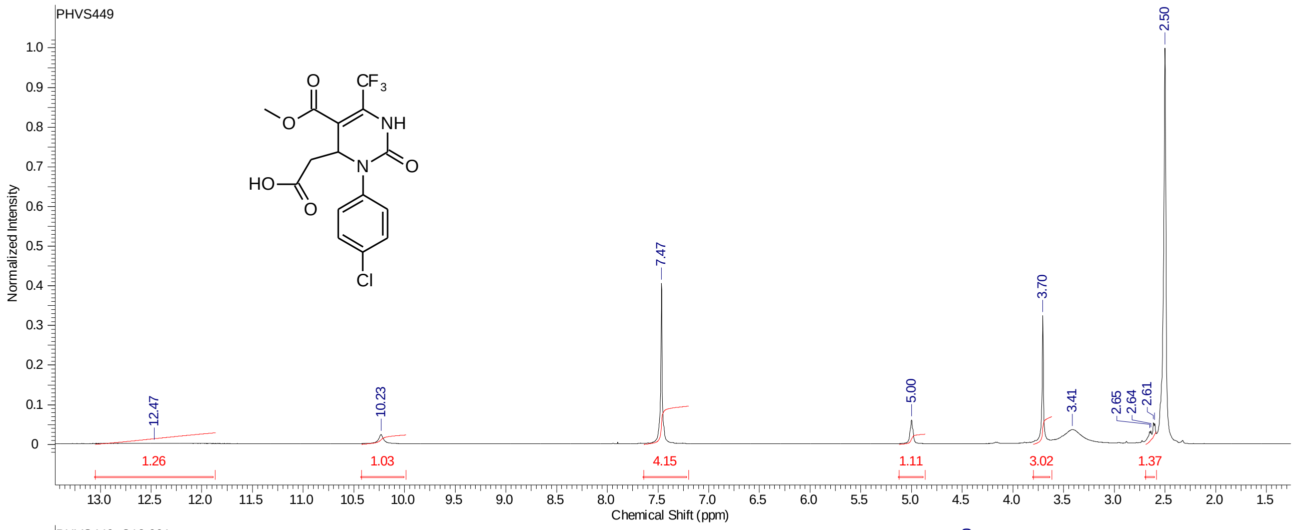
PHVS449
2.50
7.47
3.70
12.47
10.23
5.00
3.41
2.65
2.64
2.61
1.26
1.03
4.15
1.11
3.02
1.37
Normalized Intensity
Chemical Shift (ppm)
CF3
NH
HO
O
N
Cl

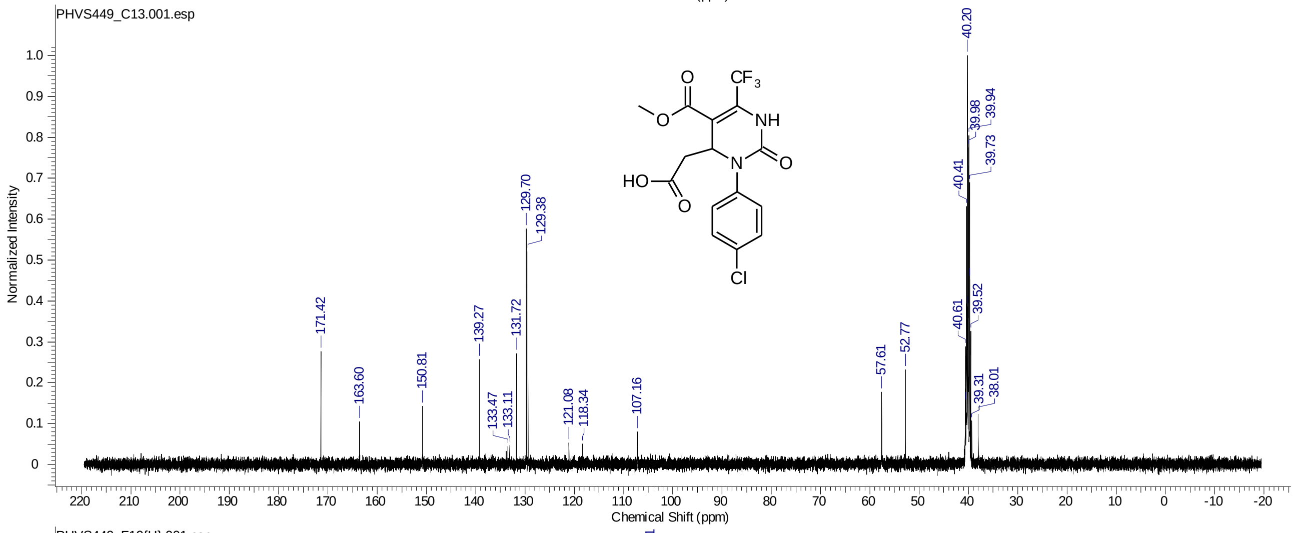
PHVS449_C13.001.esp
40.20
39.98
39.94
39.73
40.41
129.70
129.38
171.42
139.27
131.72
40.61
39.52
57.61
52.77
163.60
150.81
133.47
133.11
121.08
118.34
107.16
39.31
38.01
Normalized Intensity
Chemical Shift (ppm)
CF3
NH
HO
O
N
Cl

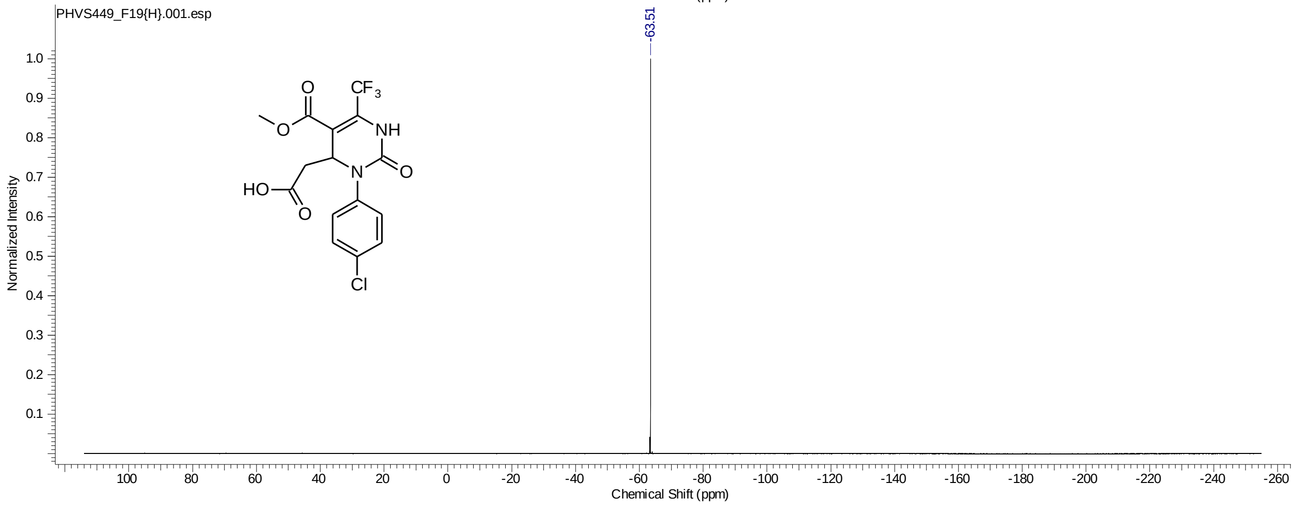
PHVS449_F19{H}.001.esp
-63.51
Normalized Intensity
Chemical Shift (ppm)
CF3
NH
HO
O
N
Cl

Compound **5m**

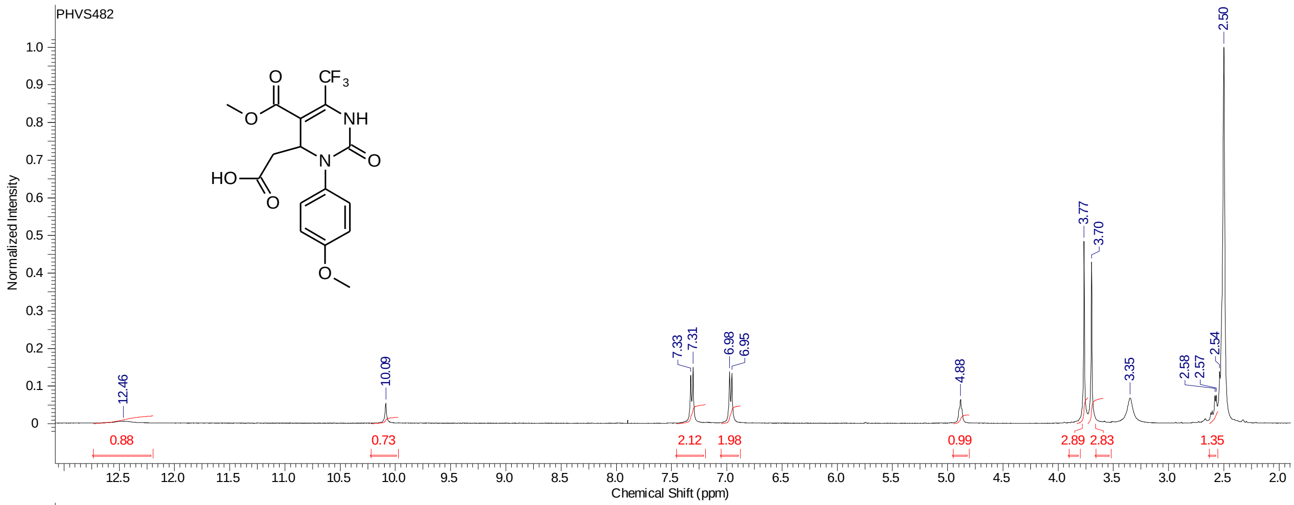
PHVS482
Normalized Intensity
Chemical Shift (ppm)
HO
O
NH
N
CF3
12.46
10.09
7.33
7.31
6.98
6.95
4.88
3.77
3.70
3.35
2.58
2.57
2.54
2.50
0.88
0.73
2.12
1.98
0.99
2.89
2.83
1.35

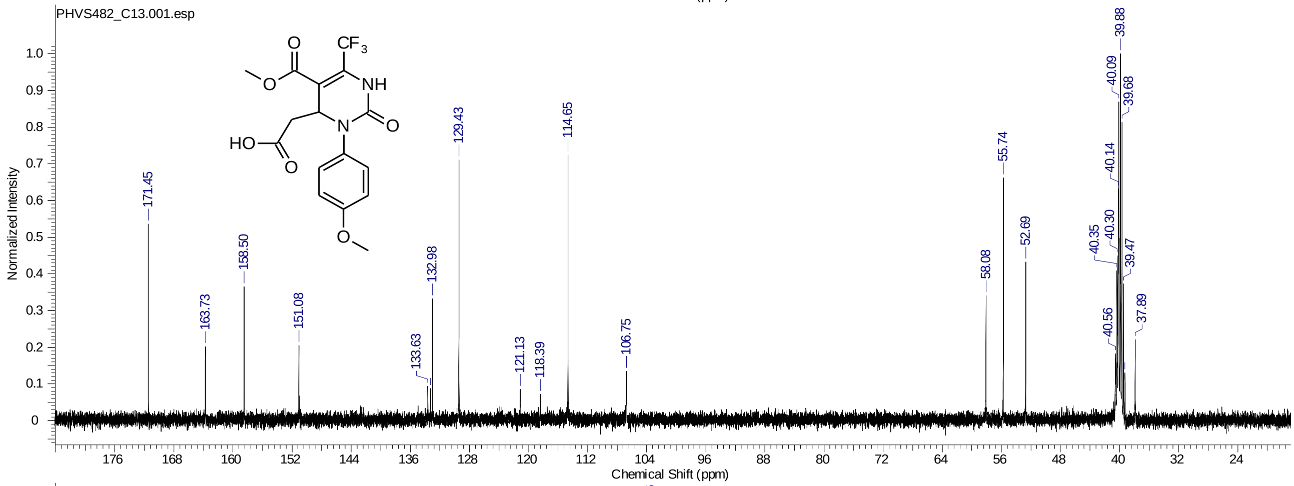
PHVS482_C13.001.esp
Normalized Intensity
Chemical Shift (ppm)
171.45
163.73
158.50
151.08
133.63
132.98
129.43
121.13
118.39
114.65
106.75
58.08
55.74
52.69
40.56
40.35
40.30
40.14
40.09
39.88
39.68
39.47
37.89

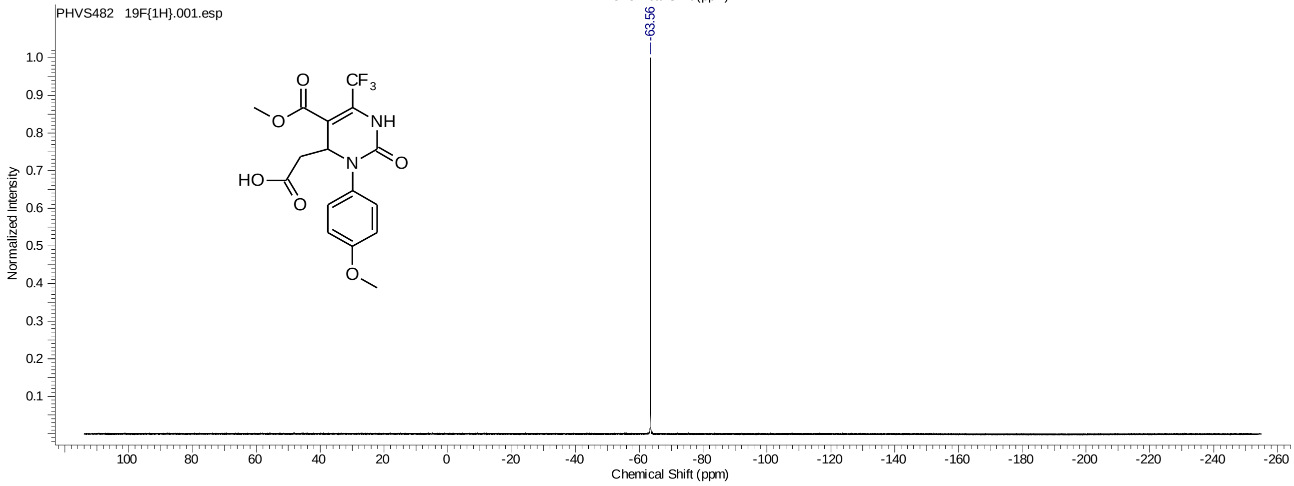
PHVS482 19F{1H}.001.esp
Normalized Intensity
Chemical Shift (ppm)
-63.56

Compound **5n**

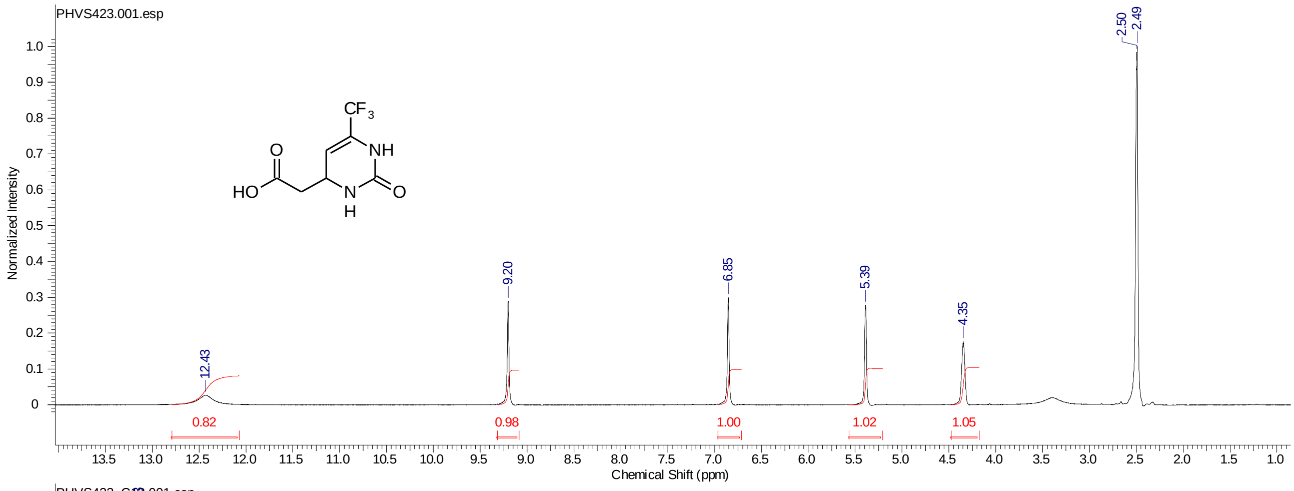
PHVS423.001.esp
Normalized Intensity
Chemical Shift (ppm)
12.43
9.20
6.85
5.39
4.35
2.50
2.49
0.82
0.98
1.00
1.02
1.05
CF3
NH
O
HO
N
H
O

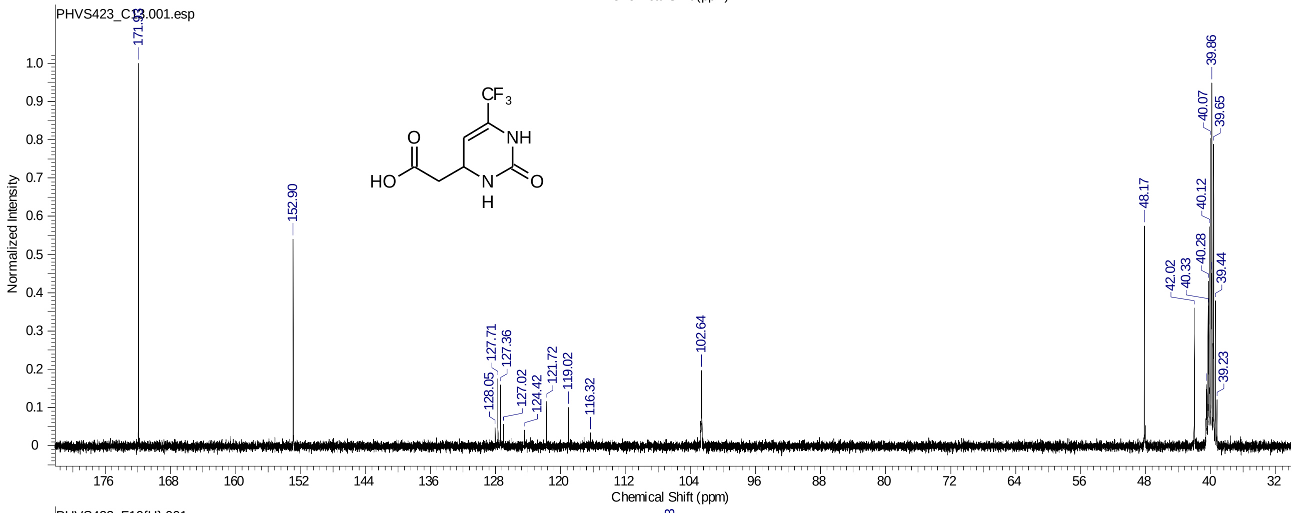
PHVS423_C13.001.esp
Normalized Intensity
Chemical Shift (ppm)
171.93
152.90
128.05
127.71
127.36
127.02
124.42
121.72
119.02
116.32
102.64
48.17
42.02
40.33
40.28
40.12
40.07
39.86
39.65
39.44
39.23
CF3
NH
O
HO
N
H
O

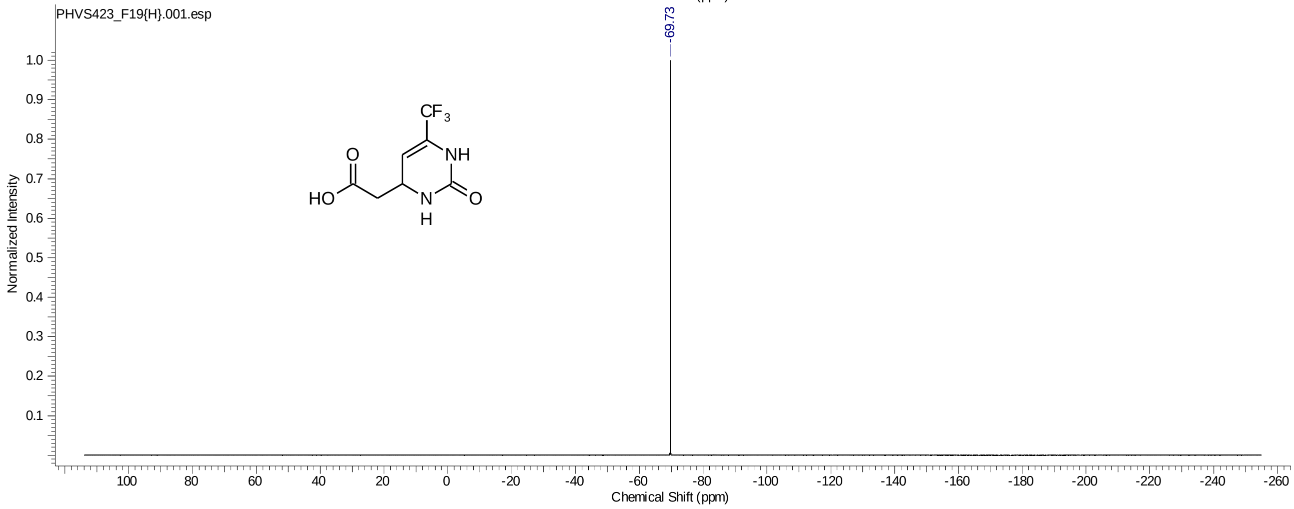
PHVS423_F19{H}.001.esp
Normalized Intensity
Chemical Shift (ppm)
-69.73
CF3
NH
O
HO
N
H
O

Compound **5o**

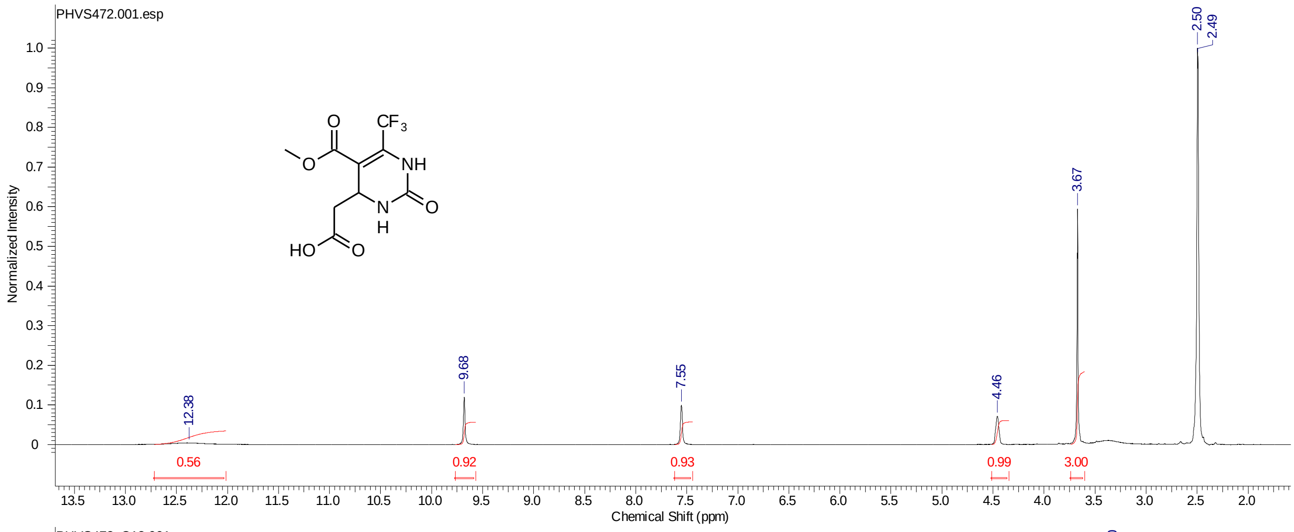
PHVS472.001.esp
Normalized Intensity
Chemical Shift (ppm)
2.50
2.49
3.67
4.46
7.55
9.68
12.38
0.56
0.92
0.93
0.99
3.00
CF3
NH
O
N
H
HO

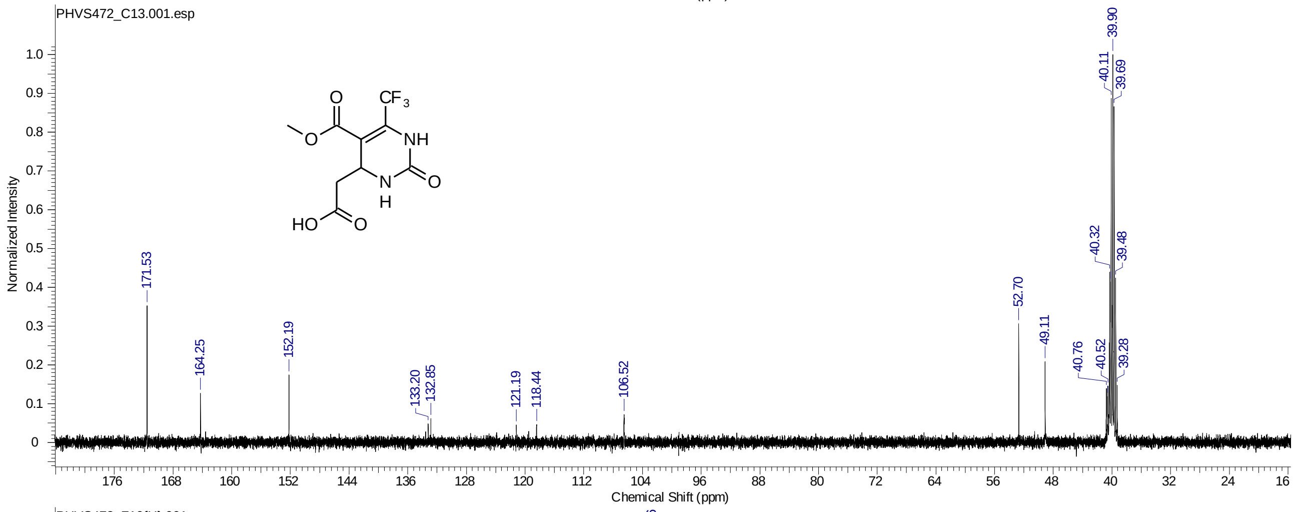
PHVS472_C13.001.esp
Normalized Intensity
Chemical Shift (ppm)
171.53
164.25
152.19
133.20
132.85
121.19
118.44
106.52
52.70
49.11
40.76
40.52
40.32
40.11
39.90
39.69
39.48
39.28

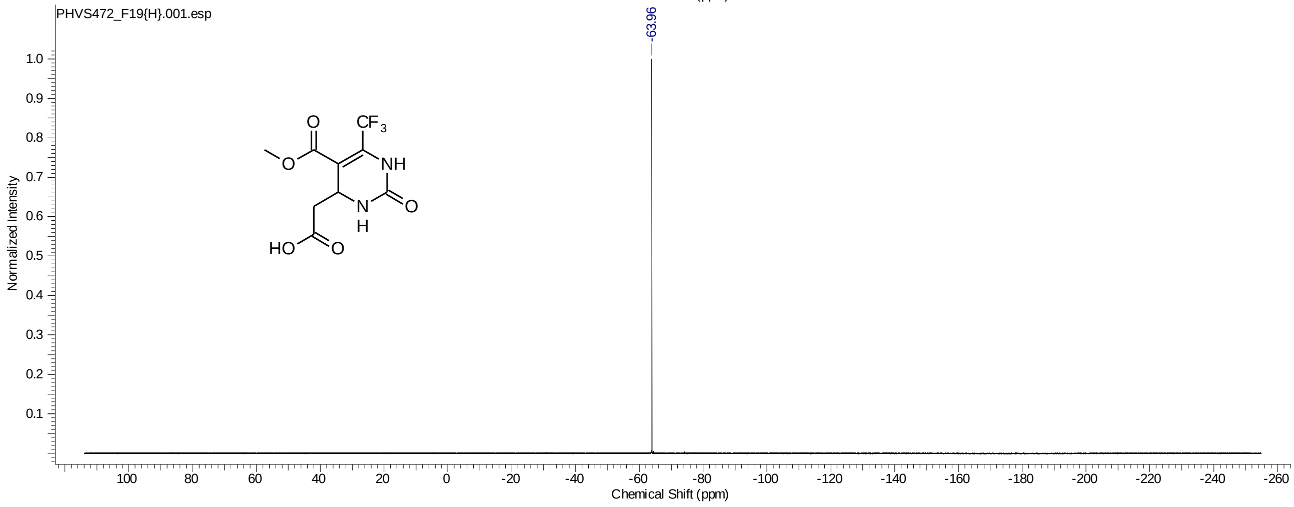
PHVS472_F19{H}.001.esp
Normalized Intensity
Chemical Shift (ppm)
-63.96

Compound **6a**

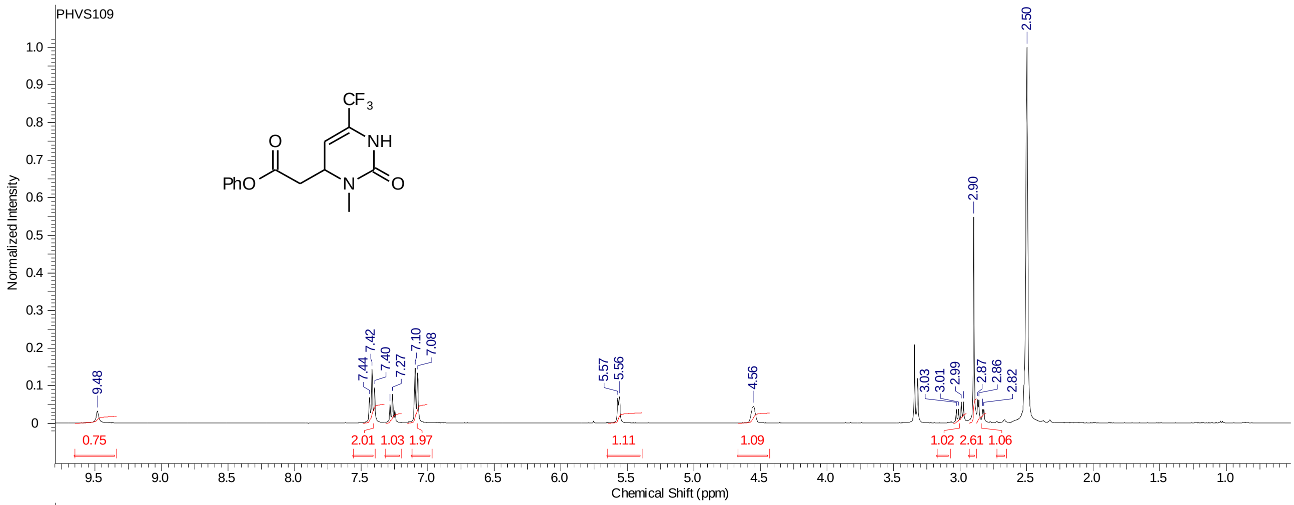
PHVS109
CF3
O
NH
PhO
N
O
Normalized Intensity
9.48
7.44
7.42
7.40
7.27
7.10
7.08
5.57
5.56
4.56
3.03
3.01
2.99
2.90
2.87
2.86
2.82
2.50
0.75
2.01
1.03
1.97
1.11
1.09
1.02
2.61
1.06
Chemical Shift (ppm)

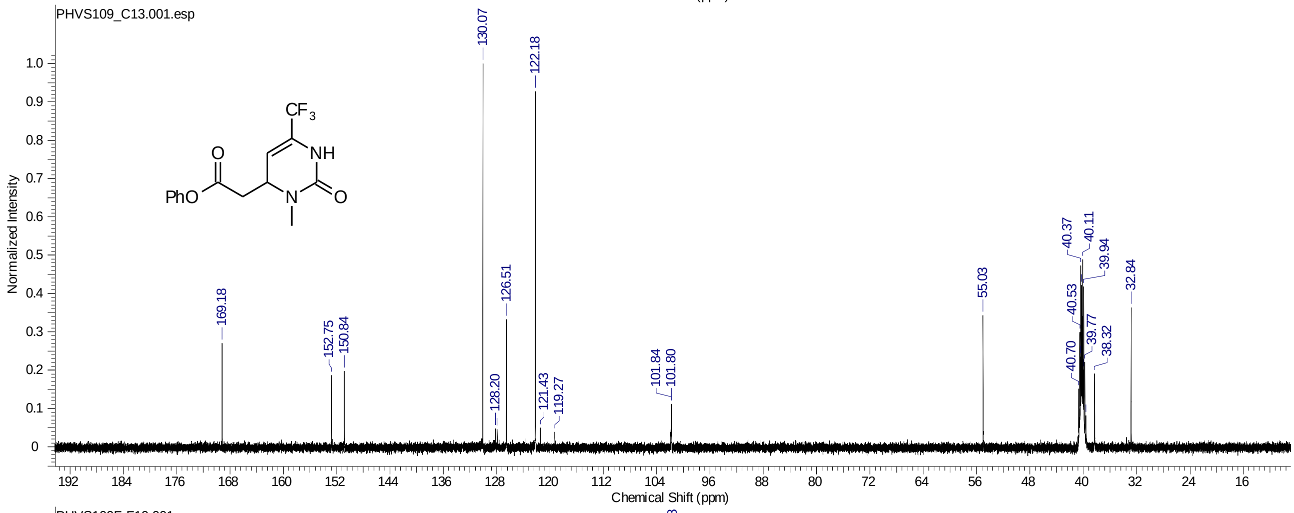
PHVS109_C13.001.esp
CF3
O
NH
PhO
N
O
Normalized Intensity
169.18
152.75
150.84
130.07
128.20
126.51
122.18
121.43
119.27
101.84
101.80
55.03
40.70
40.53
40.37
40.11
39.94
39.77
38.32
32.84
Chemical Shift (ppm)

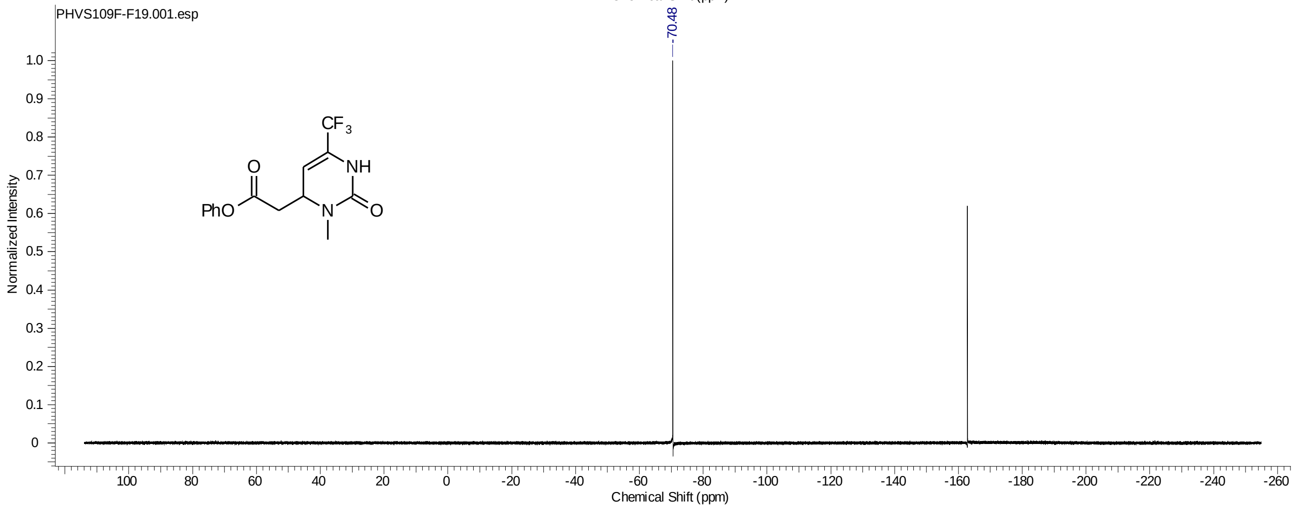
PHVS109F-F19.001.esp
CF3
O
NH
PhO
N
O
Normalized Intensity
-70.48
Chemical Shift (ppm)

Compound **6f**

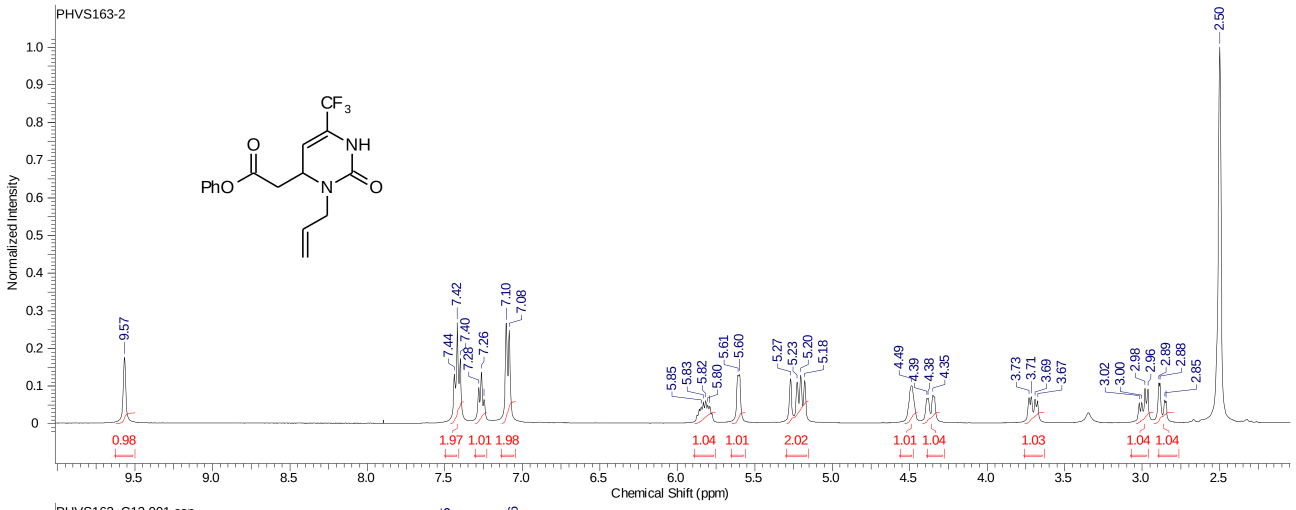
PHVS163-2
Normalized Intensity
Chemical Shift (ppm)
CF3
NH
O
PhO
N
O
9.57
7.44
7.42
7.40
7.28
7.26
7.10
7.08
5.85
5.83
5.82
5.80
5.61
5.60
5.27
5.23
5.20
5.18
4.49
4.39
4.38
4.35
3.73
3.71
3.69
3.67
3.02
3.00
2.98
2.96
2.89
2.88
2.85
2.50
0.98
1.97
1.01
1.98
1.04
1.01
2.02
1.01
1.04
1.03
1.04
1.04

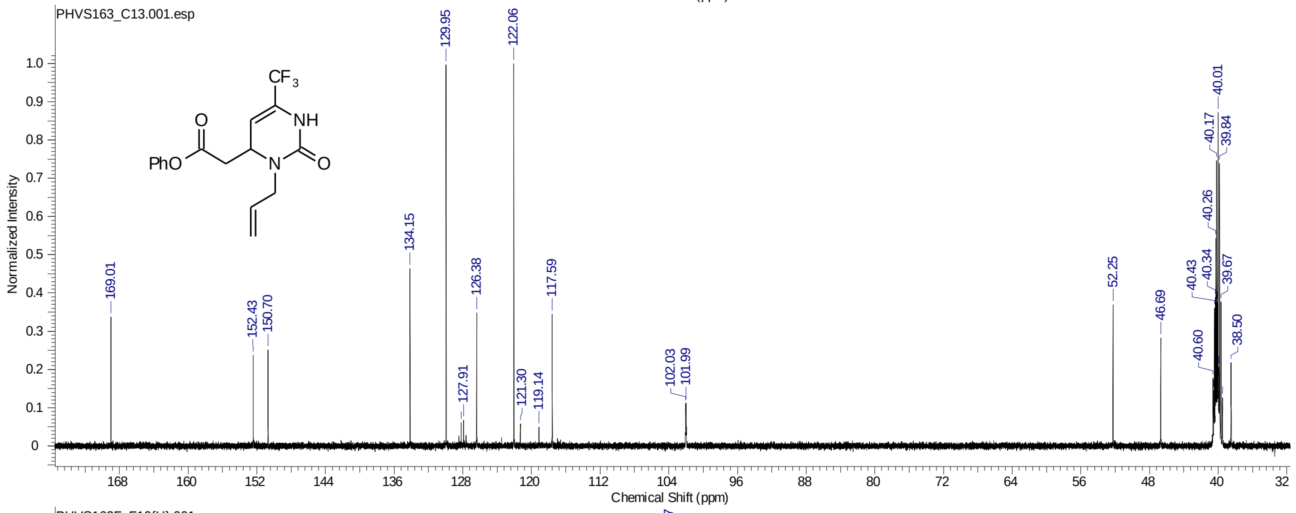
PHVS163_C13.001.esp
Normalized Intensity
Chemical Shift (ppm)
CF3
NH
O
PhO
N
O
169.01
152.43
150.70
134.15
129.95
127.91
126.38
122.06
121.30
119.14
117.59
102.03
101.99
52.25
46.69
40.60
40.43
40.34
40.26
40.17
40.01
39.84
39.67
38.50

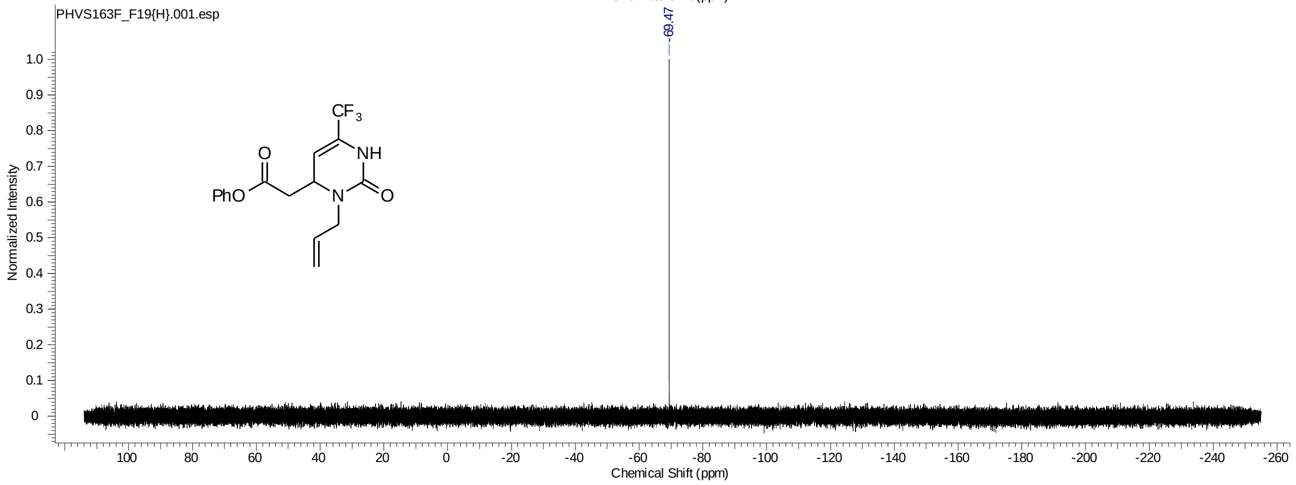
PHVS163F_F19{H}.001.esp
Normalized Intensity
Chemical Shift (ppm)
CF3
NH
O
PhO
N
O
-69.47

Compound **6g**

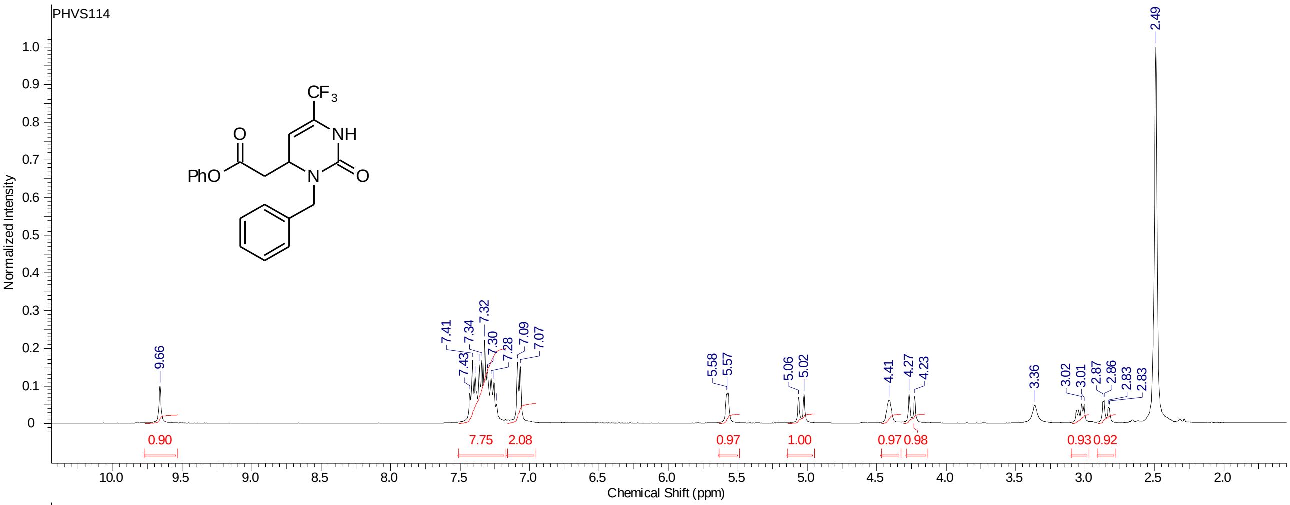
PHVS114
Normalized Intensity
Chemical Shift (ppm)
CF3
NH
O
PhO
N
O
9.66
7.43
7.41
7.34
7.32
7.30
7.28
7.09
7.07
5.58
5.57
5.06
5.02
4.41
4.27
4.23
3.36
3.02
3.01
2.87
2.86
2.83
2.83
2.49
0.90
7.75
2.08
0.97
1.00
0.97
0.98
0.93
0.92

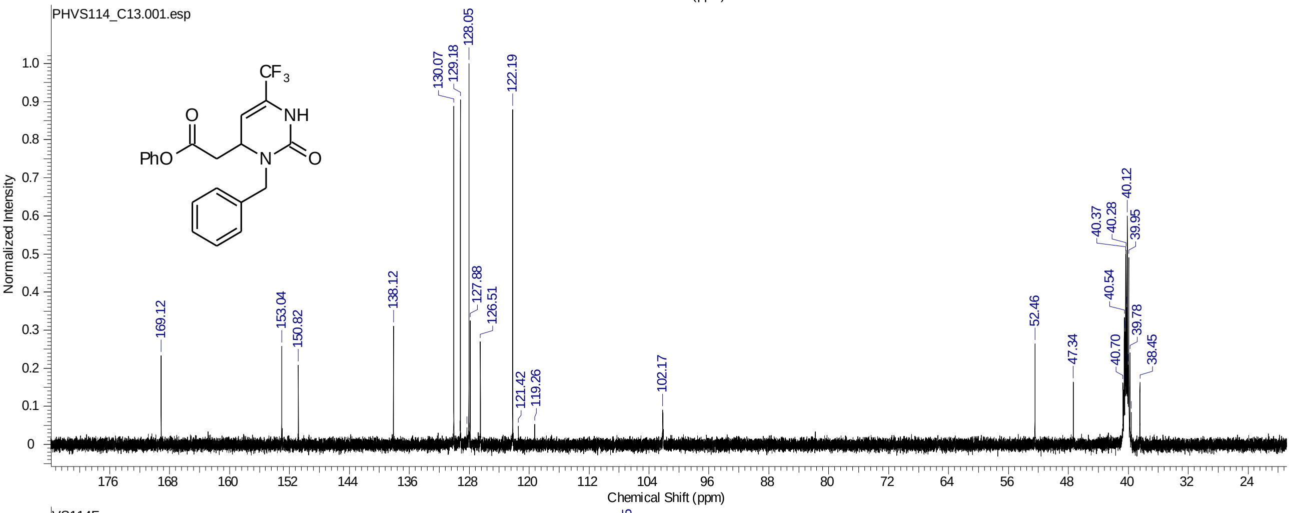
PHVS114_C13.001.esp
Normalized Intensity
Chemical Shift (ppm)
CF3
NH
O
PhO
N
O
169.12
153.04
150.82
138.12
130.07
129.18
128.05
127.88
126.51
122.19
121.42
119.26
102.17
52.46
47.34
40.70
40.54
40.37
40.28
40.12
39.95
39.78
38.45

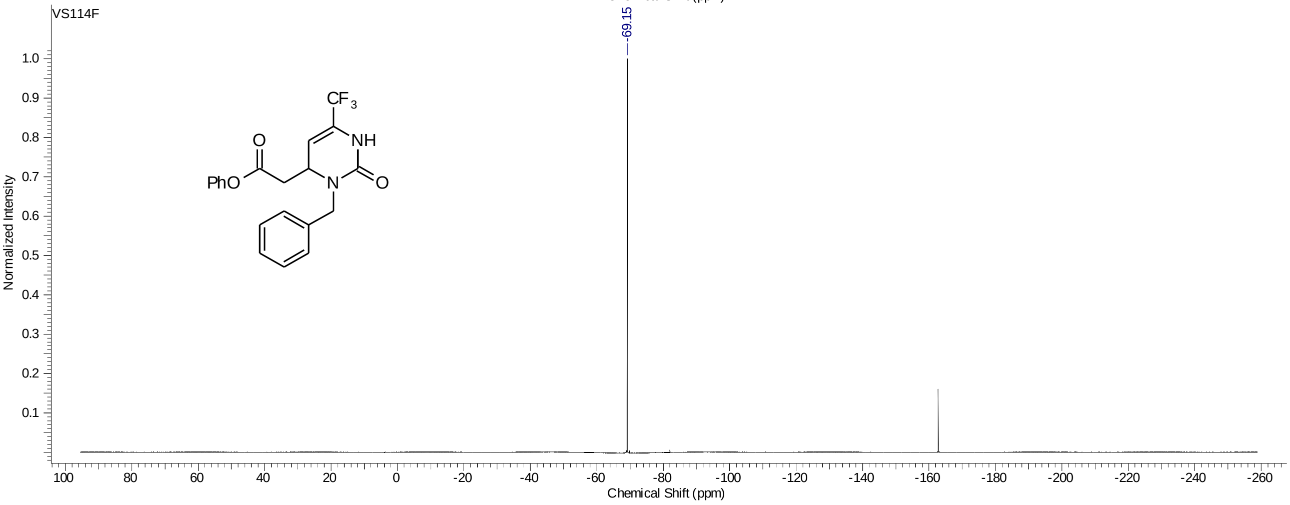
VS114F
Normalized Intensity
Chemical Shift (ppm)
CF3
NH
O
PhO
N
O
-69.15

Compound **6h**

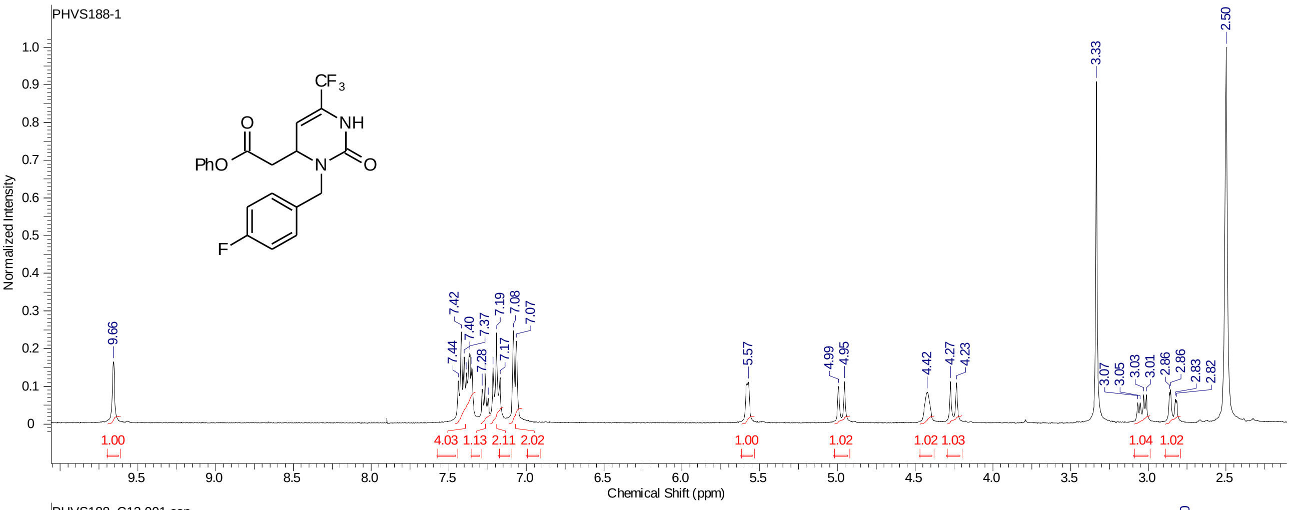
PHVS188-1
CF3
NH
O
PhO
N
O
F
Normalized Intensity
Chemical Shift (ppm)
9.66
7.44
7.42
7.40
7.37
7.28
7.19
7.17
7.08
7.07
5.57
4.99
4.95
4.42
4.27
4.23
3.33
3.07
3.05
3.03
3.01
2.86
2.86
2.83
2.82
2.50
1.00
4.03
1.13
2.11
2.02
1.00
1.02
1.02
1.03
1.04
1.02

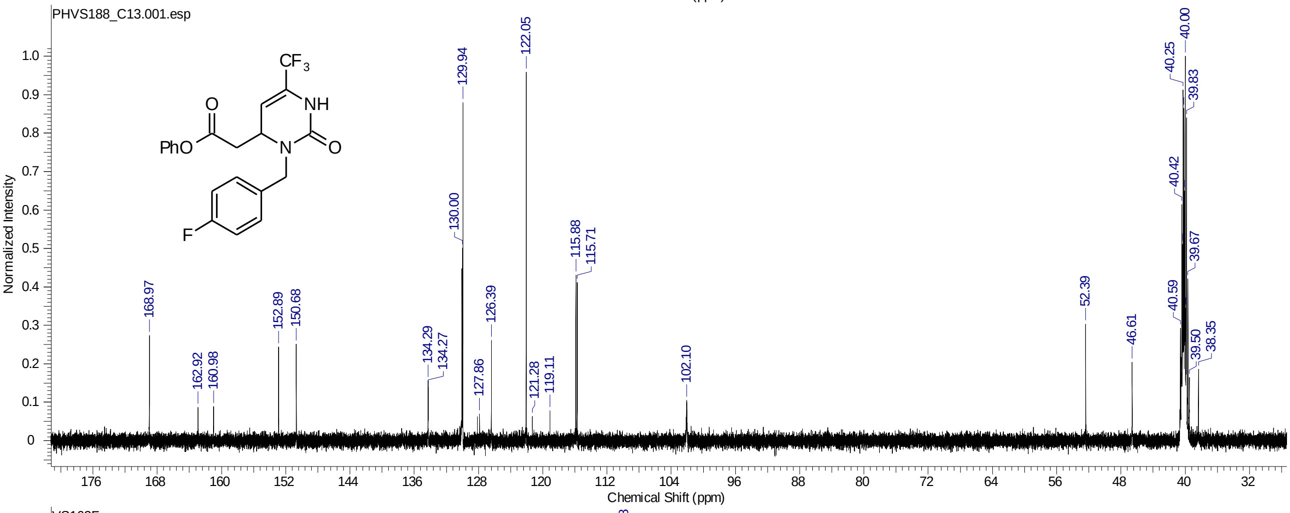
PHVS188_C13.001.esp
CF3
NH
O
PhO
N
O
F
Normalized Intensity
Chemical Shift (ppm)
168.97
162.92
160.98
152.89
150.68
134.29
134.27
130.00
129.94
127.86
126.39
122.05
121.28
119.11
115.88
115.71
102.10
52.39
46.61
40.59
40.42
40.25
40.00
39.83
39.67
39.50
38.35

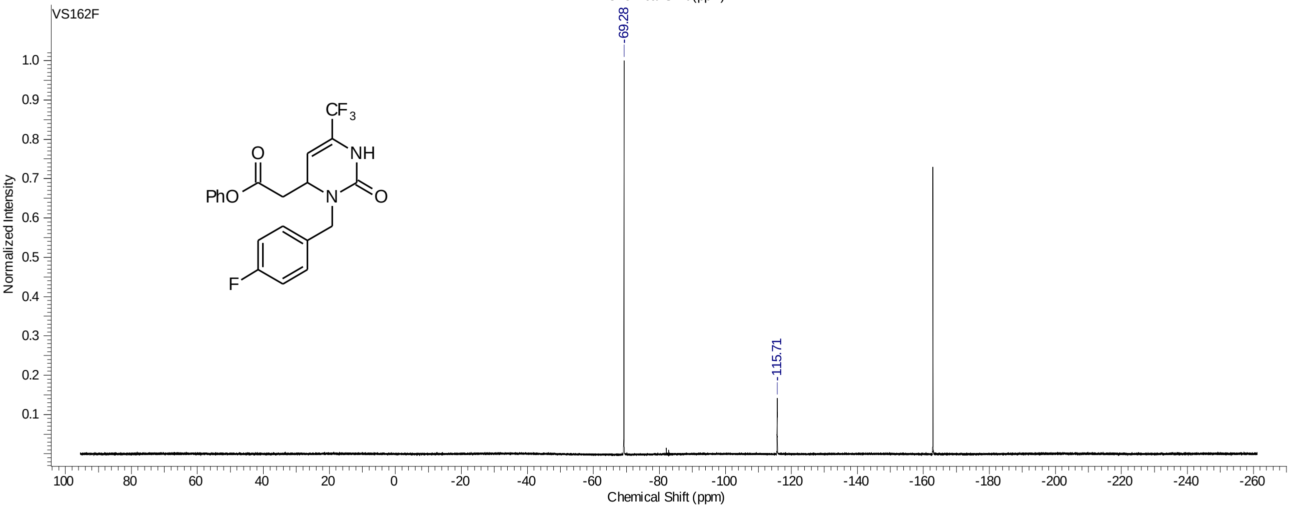
VS162F
CF3
NH
O
PhO
N
O
F
Normalized Intensity
Chemical Shift (ppm)
-69.28
-115.71

Compound **6i**

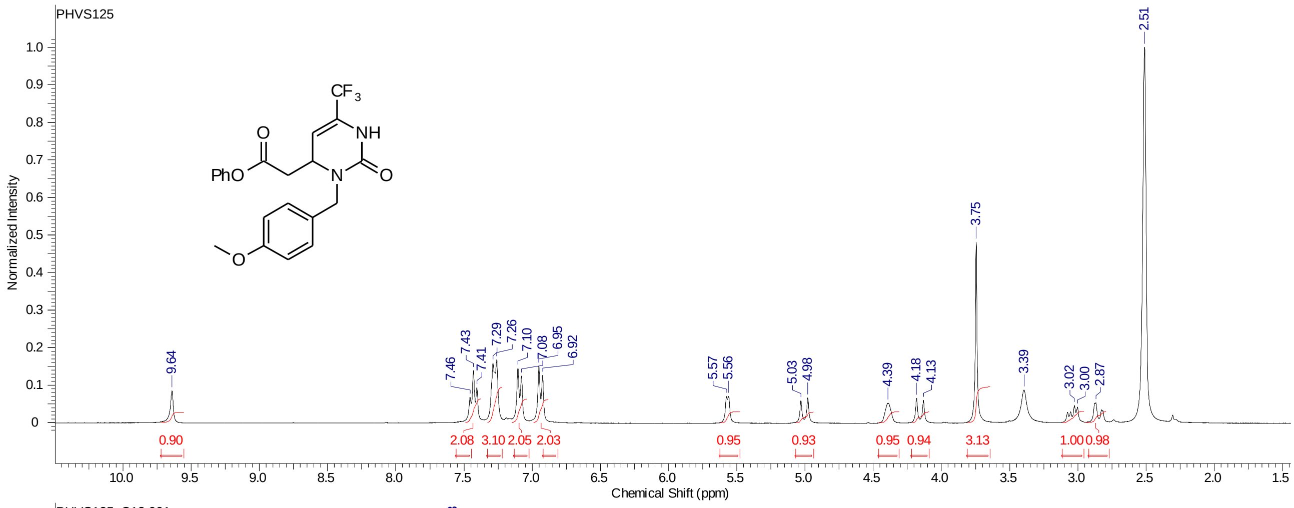
PHVS125
CF3
NH
O
PhO
N
O
O
Normalized Intensity
Chemical Shift (ppm)
2.51
3.75
9.64
7.46
7.43
7.41
7.29
7.26
7.10
7.08
6.95
6.92
5.57
5.56
5.03
4.98
4.39
4.18
4.13
3.39
3.02
3.00
2.87
0.90
2.08
3.10
2.05
2.03
0.95
0.93
0.95
0.94
3.13
1.00
0.98

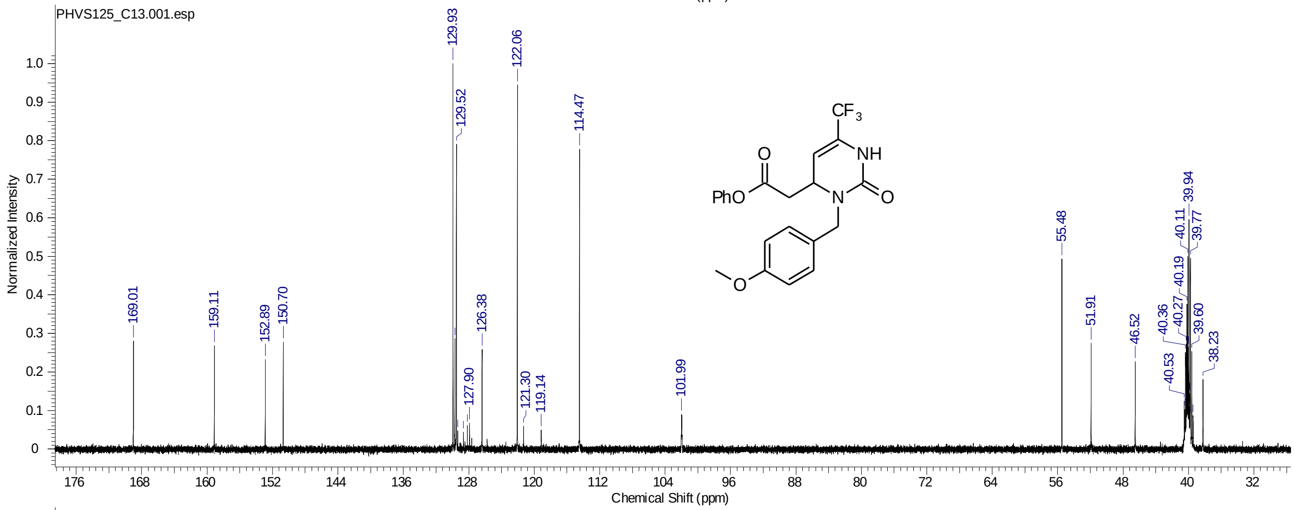
PHVS125_C13.001.esp
Normalized Intensity
Chemical Shift (ppm)
129.93
129.52
122.06
114.47
169.01
159.11
152.89
150.70
126.38
127.90
121.30
119.14
101.99
55.48
51.91
46.52
40.53
40.36
40.27
40.19
40.11
39.94
39.77
39.60
38.23

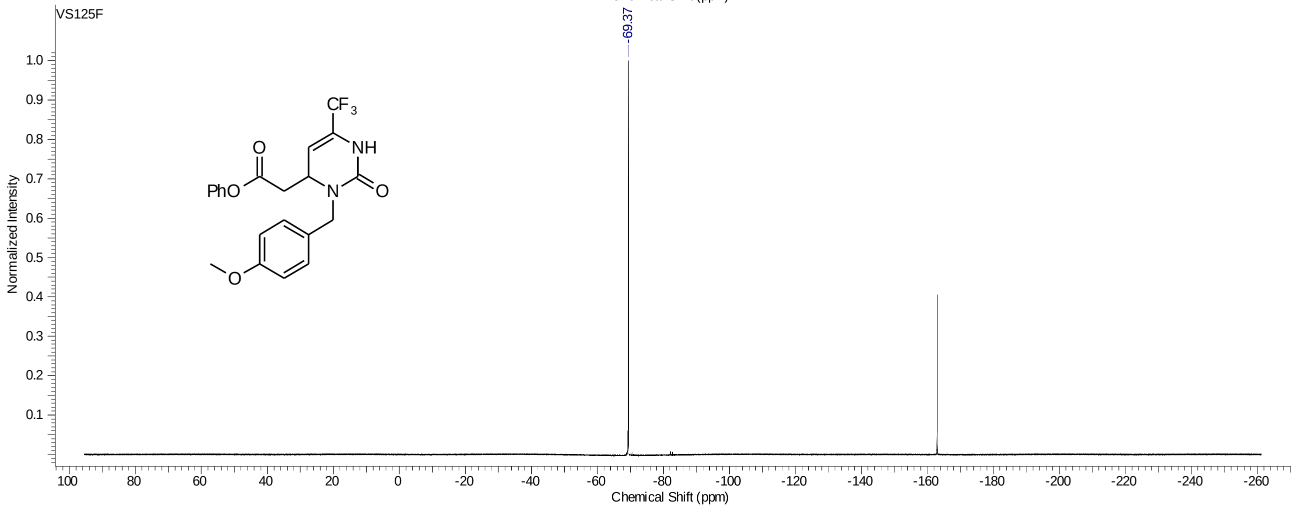
VS125F
Normalized Intensity
Chemical Shift (ppm)
-69.37

Compound 6j

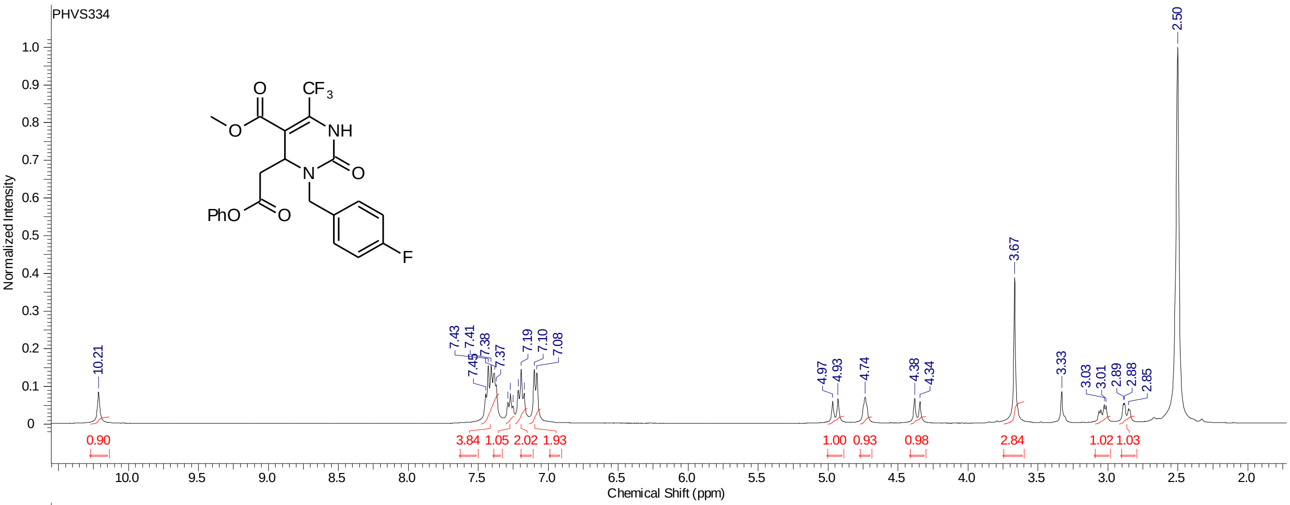
PHVS334
Normalized Intensity
Chemical Shift (ppm)
CF3
NH
N
O
PhO
F
10.21
7.45 7.43 7.41 7.38 7.37
7.19 7.10 7.08
4.97 4.93
4.74
4.38 4.34
3.67
3.33
3.03 3.01 2.89 2.88 2.85
2.50
0.90
3.84 1.05 2.02 1.93
1.00 0.93
0.98
2.84
1.02 1.03

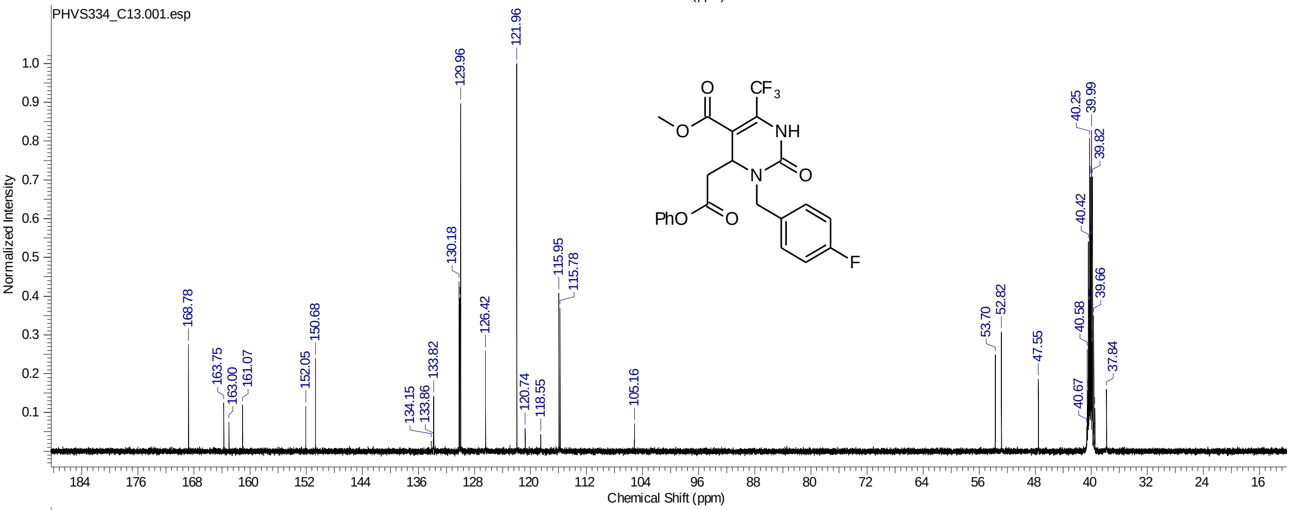
PHVS334_C13.001.esp
Normalized Intensity
Chemical Shift (ppm)
CF3
NH
N
O
PhO
F
168.78
163.75 163.00 161.07
152.05 150.68
134.15 133.86 133.82
130.18 129.96
126.42
121.96
120.74 118.55
115.95 115.78
105.16
53.70 52.82
47.55
40.67 40.58 40.42 40.25 39.99 39.82 39.66
37.84

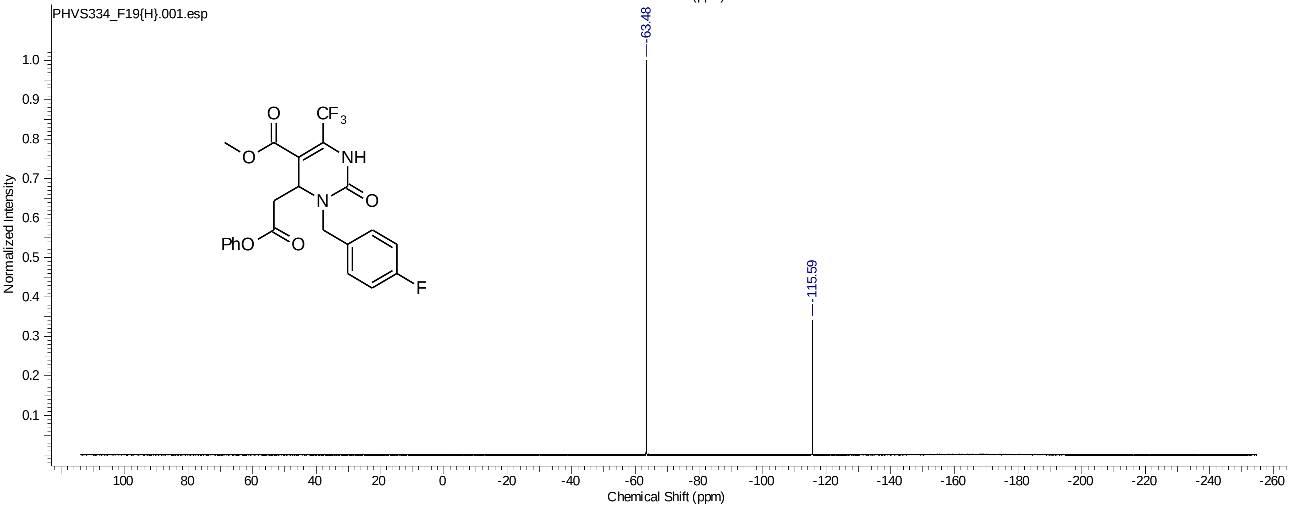
PHVS334_F19{H}.001.esp
Normalized Intensity
Chemical Shift (ppm)
CF3
NH
N
O
PhO
F
-63.48
-115.59

Compound **6k**

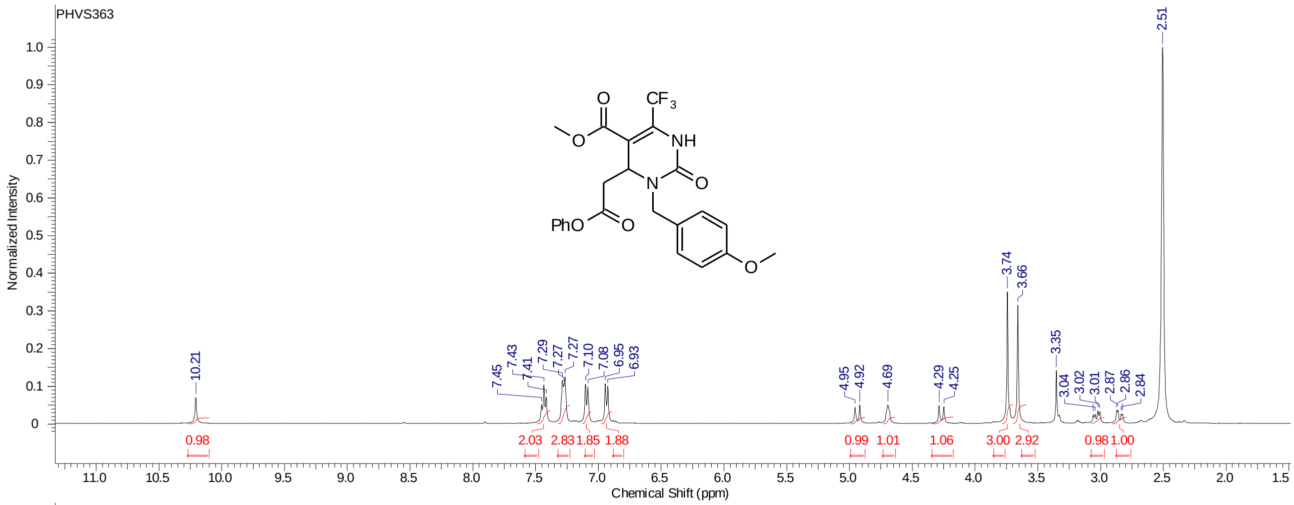
PHVS363
Normalized Intensity
Chemical Shift (ppm)
10.21
7.45 7.43 7.41 7.29 7.27 7.27 7.10 7.08 6.95 6.93
4.95 4.92 4.69 4.29 4.25
3.74 3.66 3.35 3.04 3.02 3.01 2.87 2.86 2.84
2.51
0.98 2.03 2.83 1.85 1.88 0.99 1.01 1.06 3.00 2.92 0.98 1.00
CF3
NH
PhO

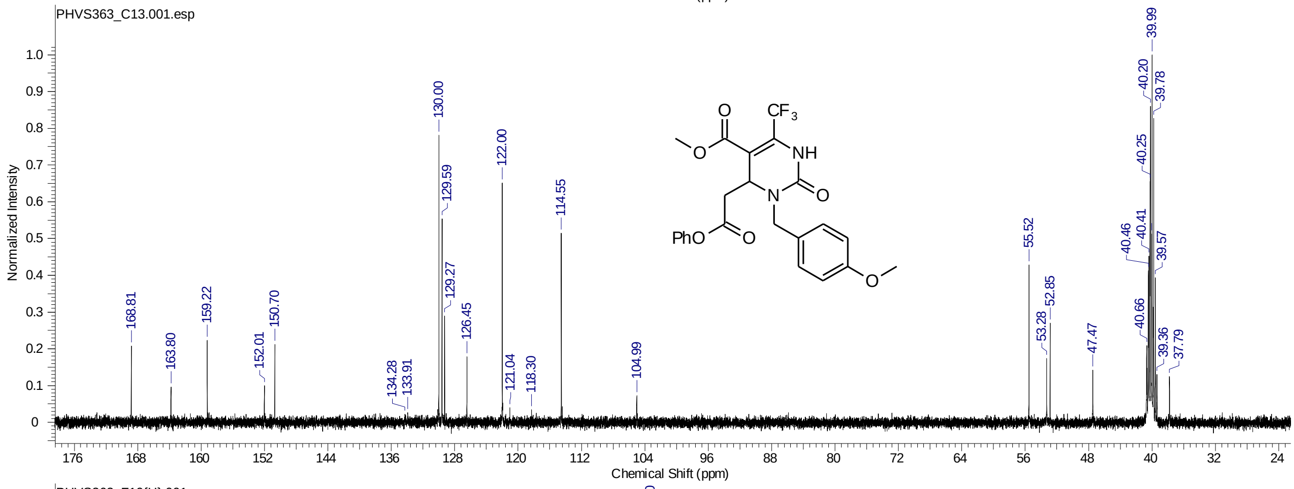
PHVS363_C13.001.esp
Normalized Intensity
Chemical Shift (ppm)
168.81 163.80 159.22 152.01 150.70
134.28 133.91 130.00 129.59 129.27 126.45 122.00 121.04 118.30 114.55 104.99
55.52 53.28 52.85 47.47
40.66 40.46 40.41 40.25 40.20 39.99 39.78 39.57 39.36 37.79
CF3
NH
PhO

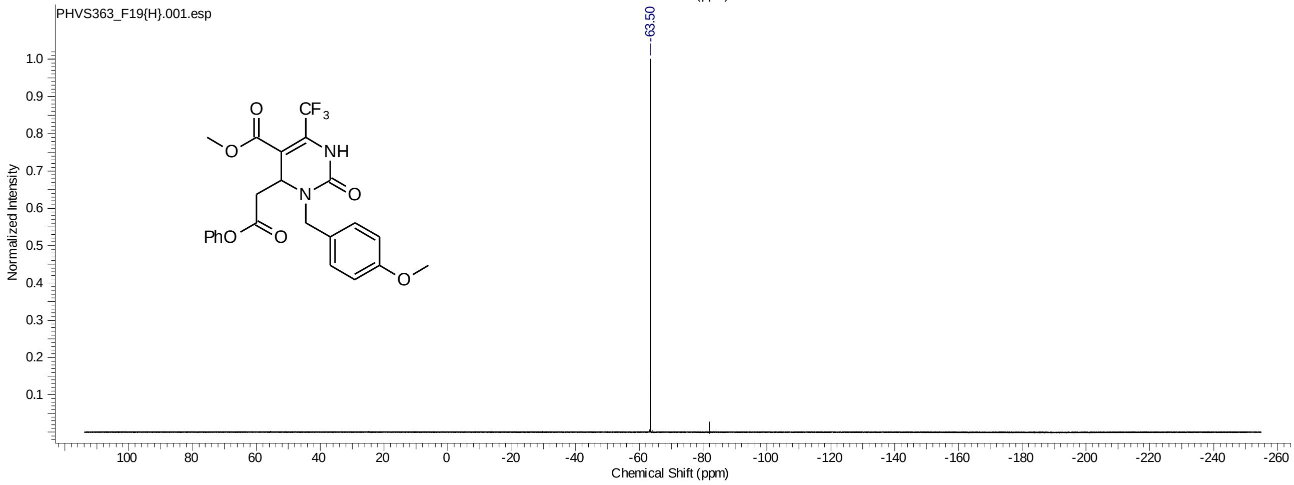
PHVS363_F19{H}.001.esp
Normalized Intensity
Chemical Shift (ppm)
-63.50
CF3
NH
PhO

Compound **6l**

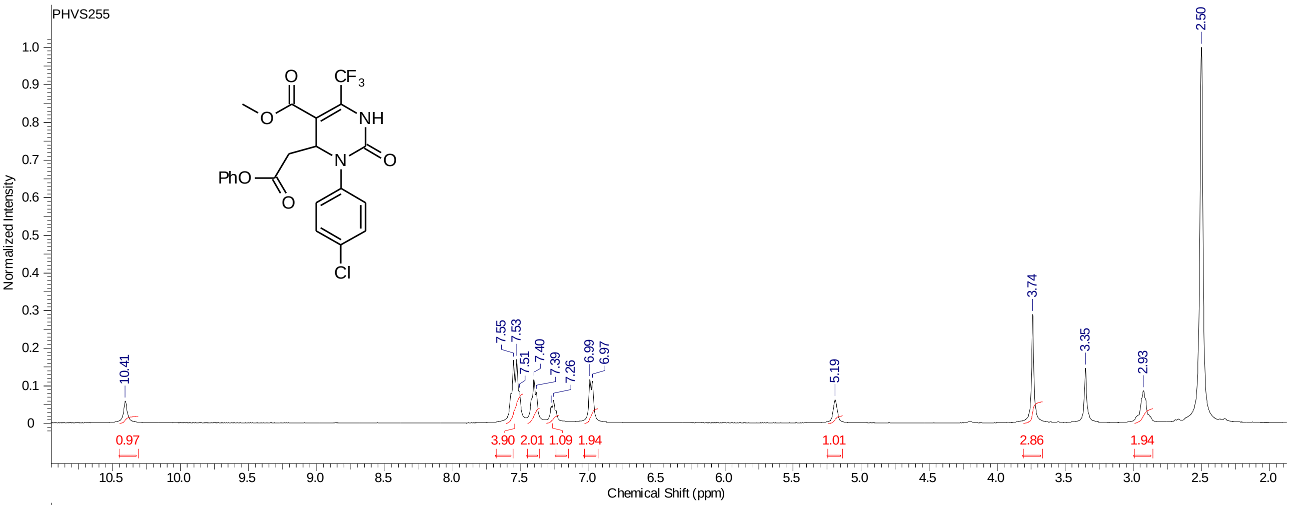
PHVS255
Normalized Intensity
Chemical Shift (ppm)
10.41
7.55
7.53
7.51
7.40
7.39
7.26
6.99
6.97
5.19
3.74
3.35
2.93
2.50
0.97
3.90
2.01
1.09
1.94
1.01
2.86
1.94
CF3
NH
O
N
PhO
Cl

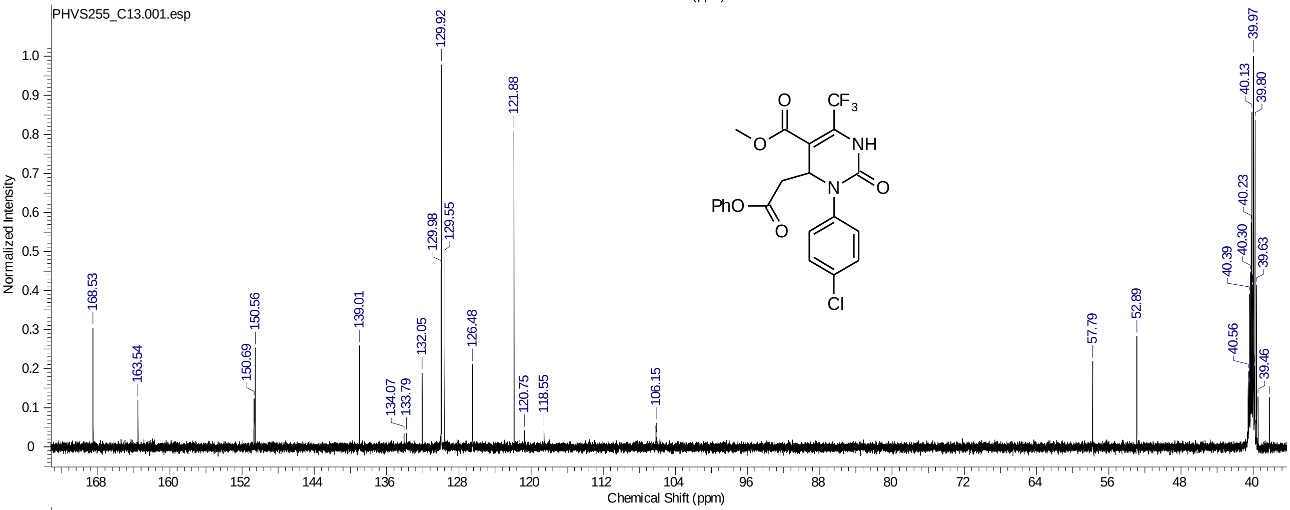
PHVS255_C13.001.esp
Normalized Intensity
Chemical Shift (ppm)
168.53
163.54
150.69
150.56
139.01
134.07
133.79
132.05
129.98
129.92
129.55
126.48
121.88
120.75
118.55
106.15
57.79
52.89
40.56
40.39
40.30
40.23
40.13
39.97
39.80
39.63
39.46
CF3
NH
O
N
PhO
Cl

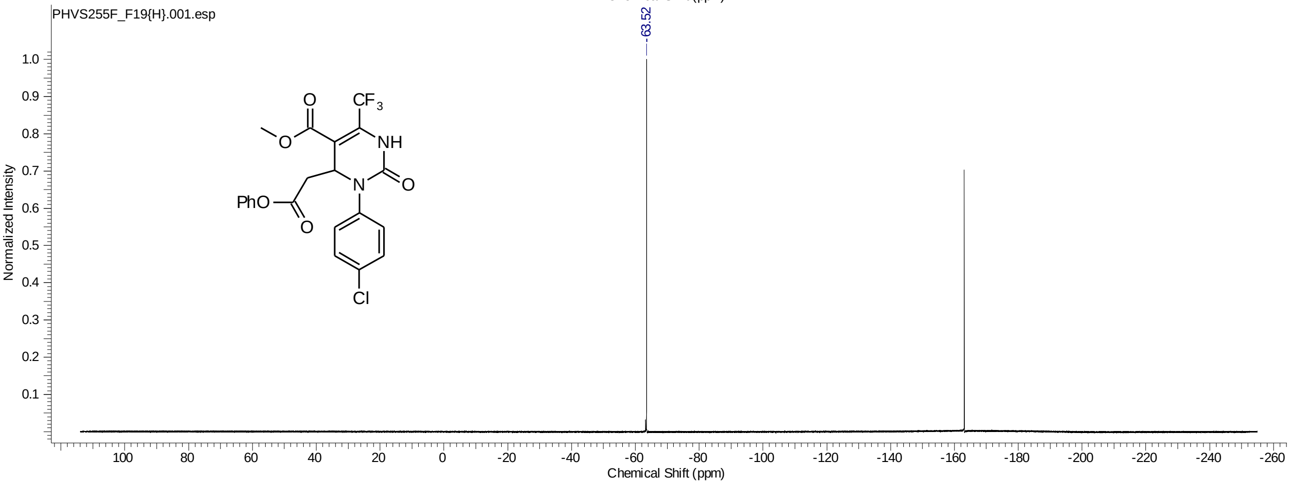
PHVS255F_F19{H}.001.esp
Normalized Intensity
Chemical Shift (ppm)
-63.52
CF3
NH
O
N
PhO
Cl

Compound **6m**

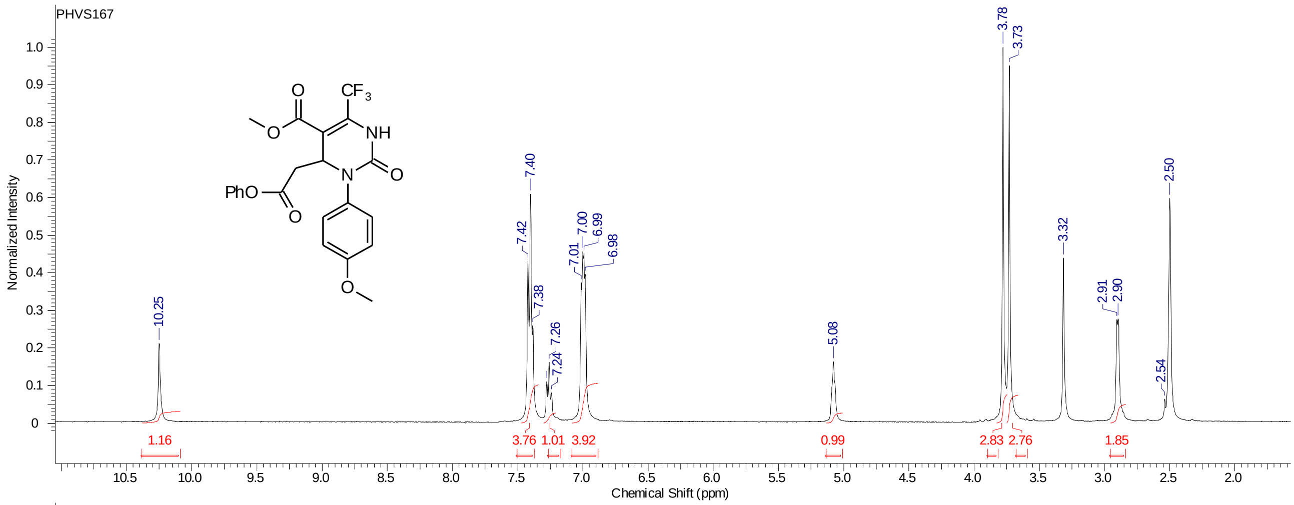
PHVS167
Normalized Intensity
Chemical Shift (ppm)
10.25
7.42
7.40
7.38
7.26
7.24
7.01
7.00
6.99
6.98
5.08
3.78
3.73
3.32
2.91
2.90
2.54
2.50
1.16
3.76
1.01
3.92
0.99
2.83
2.76
1.85
CF3
NH
O
N
PhO

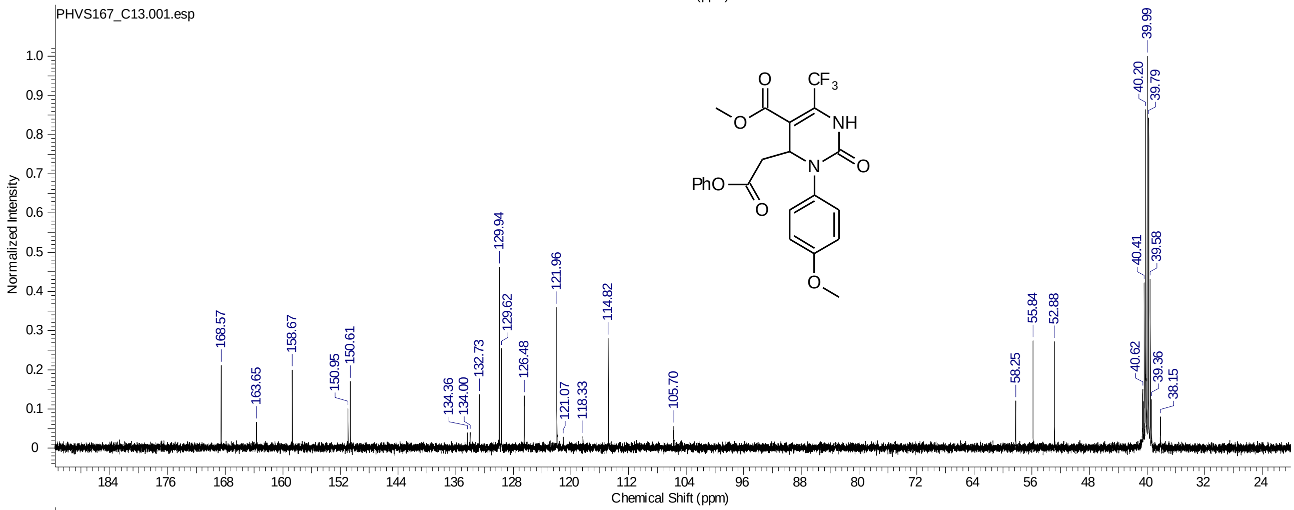
PHVS167_C13.001.esp
Normalized Intensity
Chemical Shift (ppm)
168.57
163.65
158.67
150.95
150.61
134.36
134.00
132.73
129.94
129.62
126.48
121.96
121.07
118.33
114.82
105.70
58.25
55.84
52.88
40.62
40.41
40.20
39.99
39.79
39.58
39.36
38.15

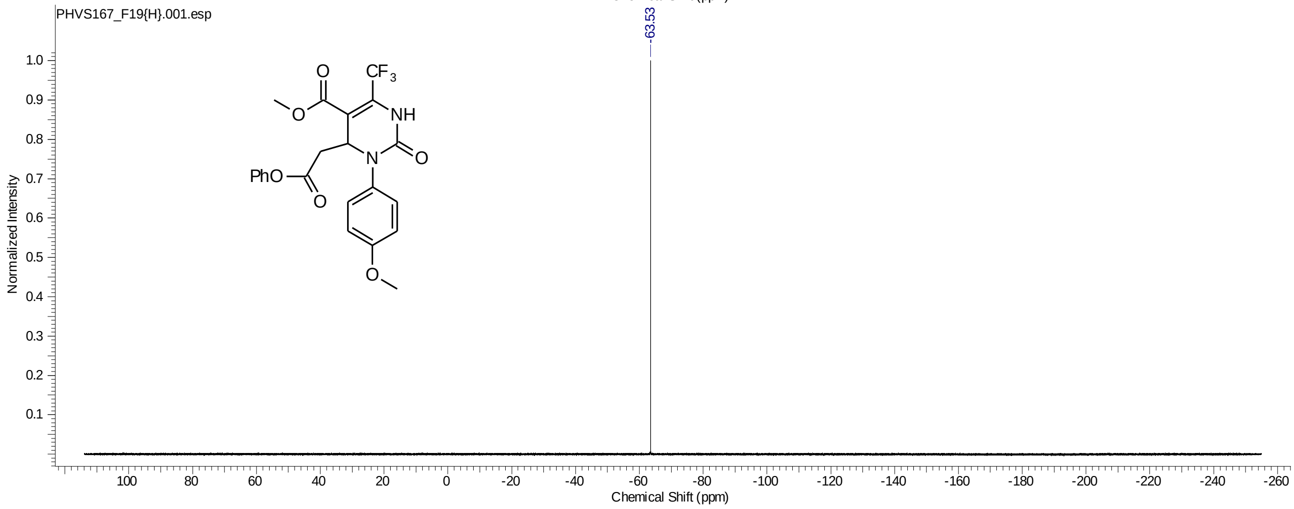
PHVS167_F19{H}.001.esp
Normalized Intensity
Chemical Shift (ppm)
-63.53

Compound **6n**

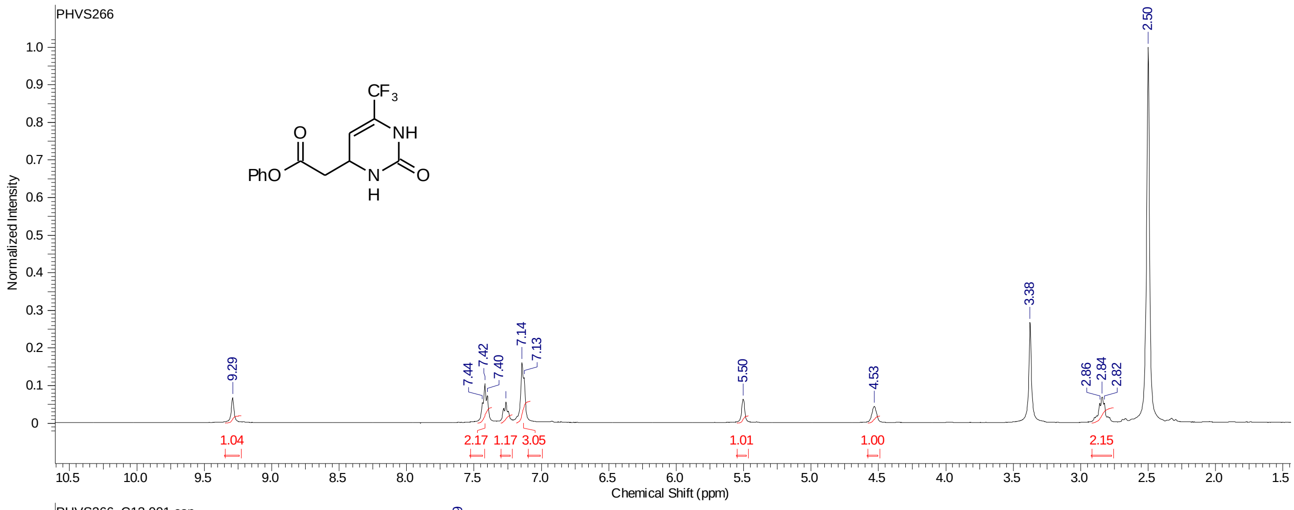
PHVS266
CF3
NH
O
PhO
N
H
O
Normalized Intensity
Chemical Shift (ppm)
9.29
7.44
7.42
7.40
7.14
7.13
5.50
4.53
3.38
2.86
2.84
2.82
2.50
1.04
2.17
1.17
3.05
1.01
1.00
2.15

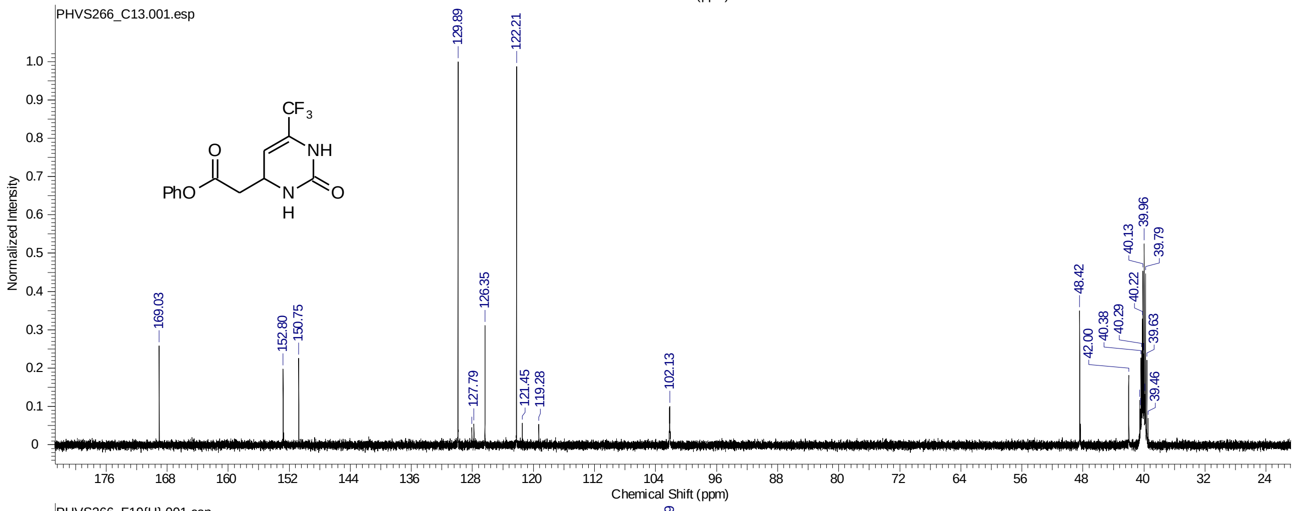
PHVS266_C13.001.esp
Normalized Intensity
Chemical Shift (ppm)
169.03
152.80
150.75
129.89
127.79
126.35
122.21
121.45
119.28
102.13
48.42
42.00
40.38
40.29
40.22
40.13
39.96
39.79
39.63
39.46

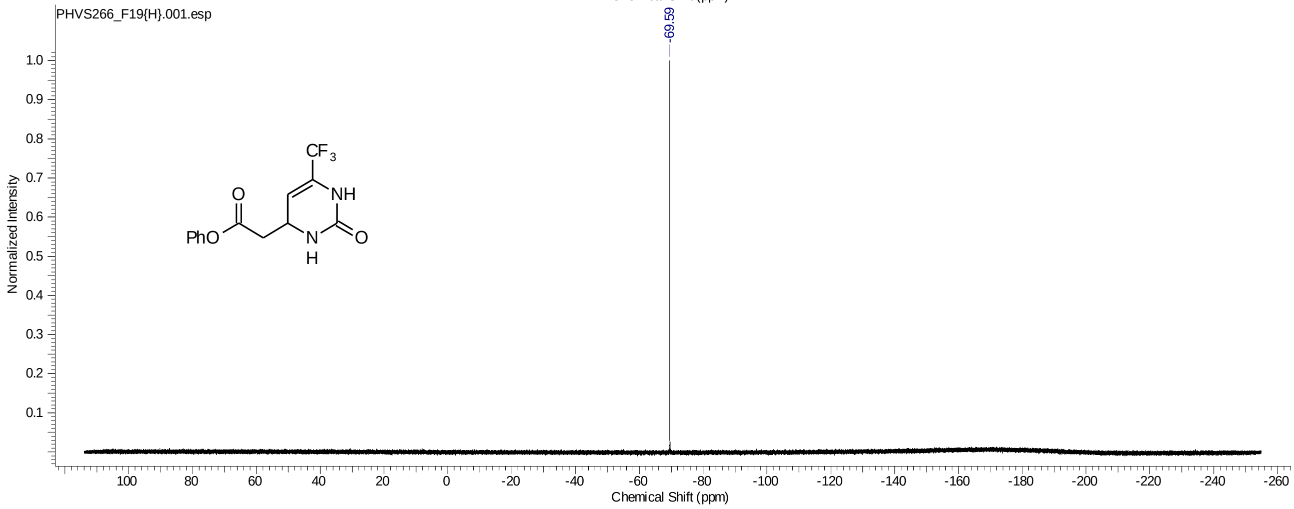
PHVS266_F19{H}.001.esp
Normalized Intensity
Chemical Shift (ppm)
-69.59

Compound **6o**

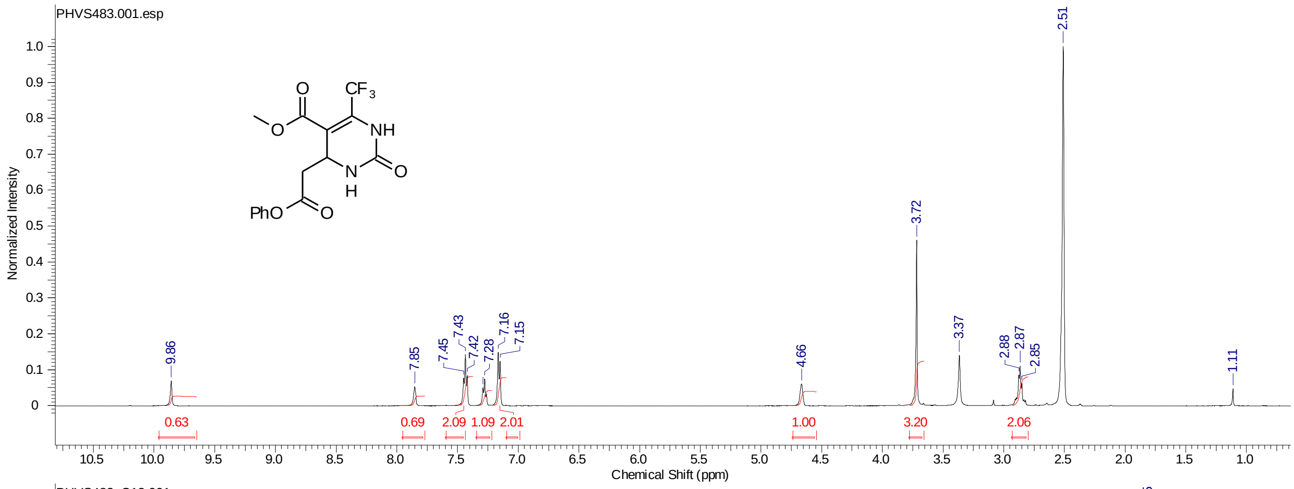
PHVS483.001.esp
Normalized Intensity
Chemical Shift (ppm)
9.86
7.85
7.45
7.43
7.42
7.28
7.16
7.15
4.66
3.72
3.37
2.88
2.87
2.85
2.51
1.11
0.63
0.69
2.09
1.09
2.01
1.00
3.20
2.06
CF3
NH
O
N
H
PhO

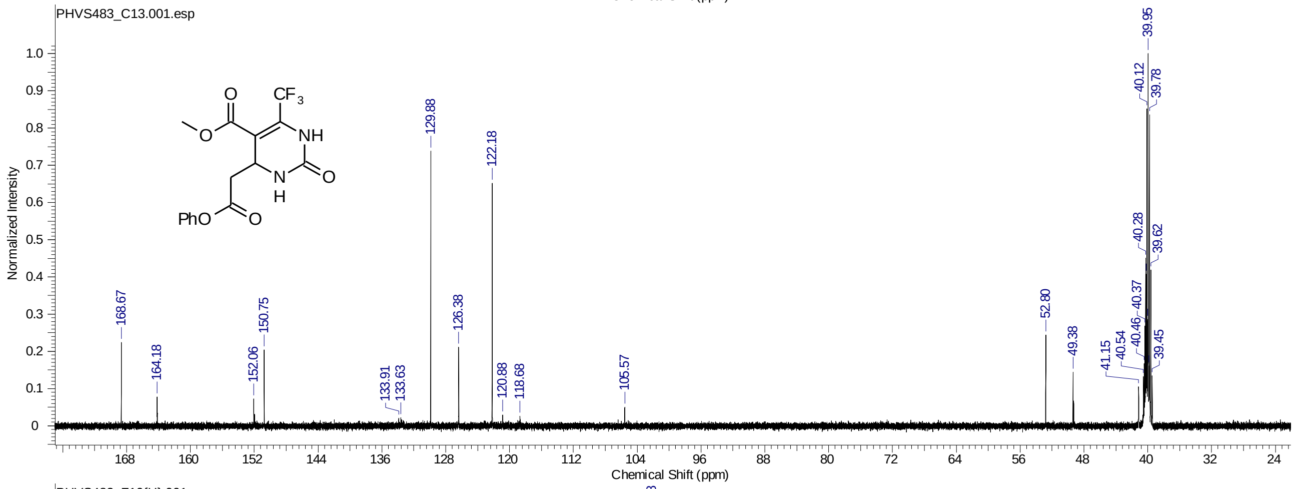
PHVS483_C13.001.esp
Normalized Intensity
Chemical Shift (ppm)
168.67
164.18
152.06
150.75
133.91
133.63
129.88
126.38
122.18
120.88
118.68
105.57
52.80
49.38
41.15
40.54
40.46
40.37
40.28
40.12
39.95
39.78
39.62
39.45
CF3
NH
O
N
H
PhO

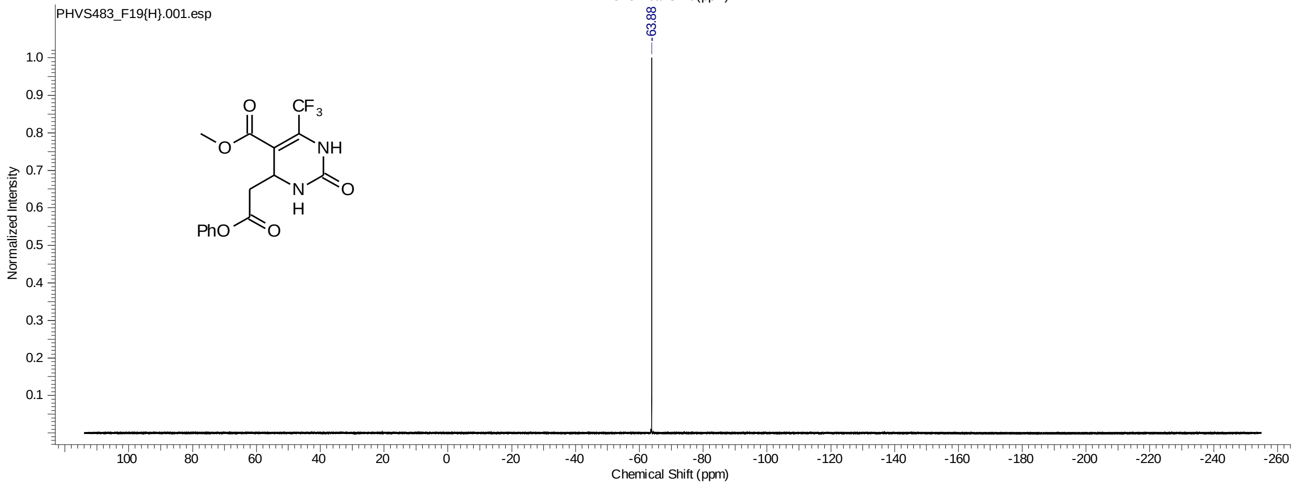
PHVS483_F19{H}.001.esp
Normalized Intensity
Chemical Shift (ppm)
-63.88
CF3
NH
O
N
H
PhO

Compound **8a**

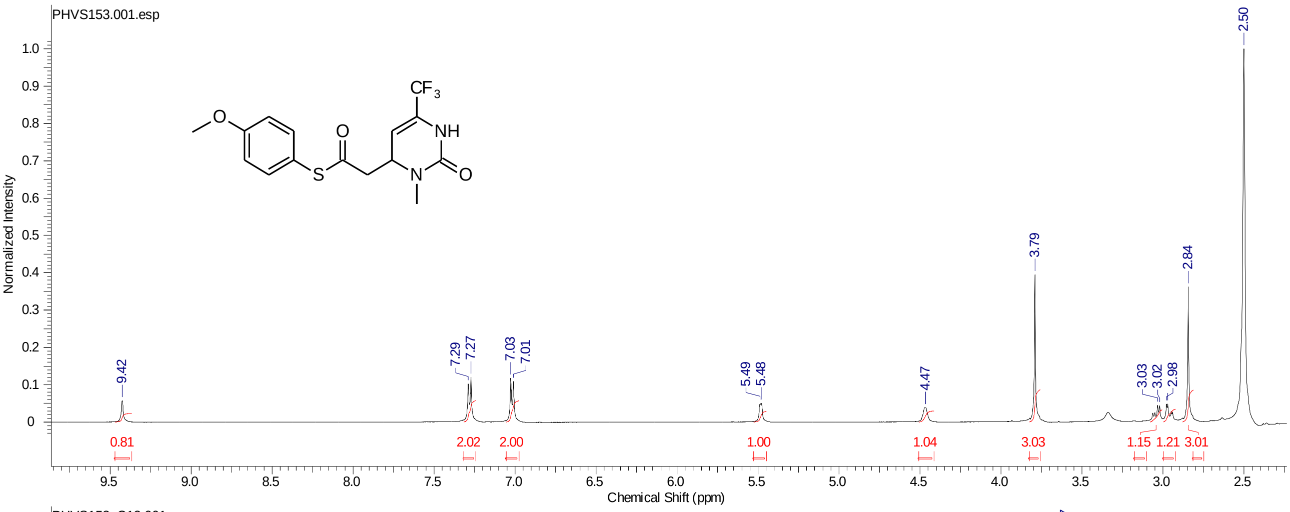
PHVS153.001.esp
Normalized Intensity
Chemical Shift (ppm)
9.42
7.29
7.27
7.03
7.01
5.49
5.48
4.47
3.79
3.03
3.02
2.98
2.84
2.50
0.81
2.02
2.00
1.00
1.04
3.03
1.15
1.21
3.01
CF3
NH
O
S
N
O
O

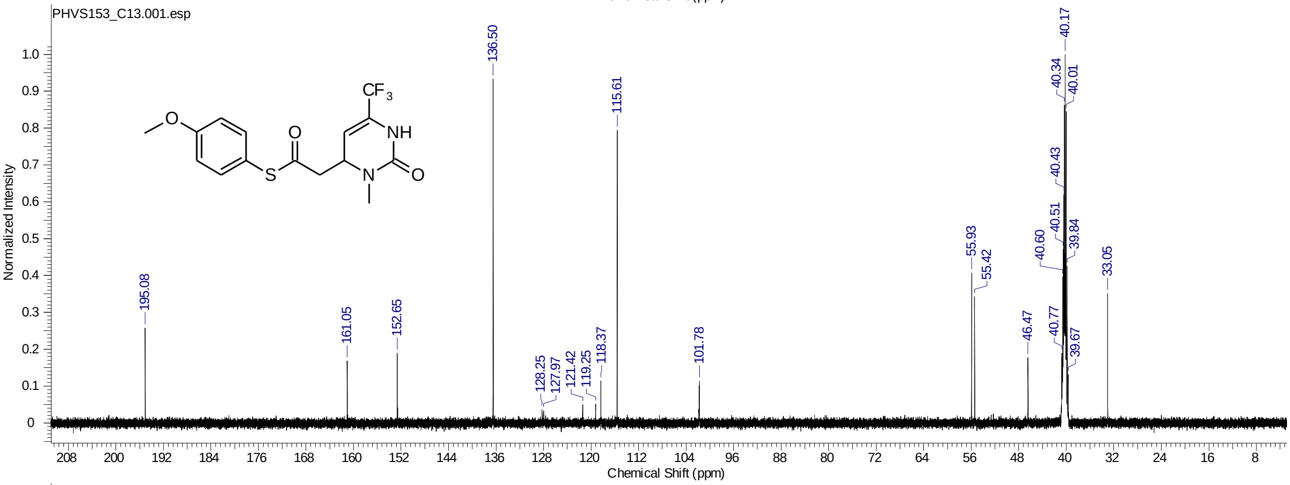
PHVS153_C13.001.esp
Normalized Intensity
Chemical Shift (ppm)
195.08
161.05
152.65
136.50
128.25
127.97
121.42
119.25
118.37
115.61
101.78
55.93
55.42
46.47
40.77
40.60
40.51
40.43
40.34
40.17
40.01
39.84
39.67
33.05

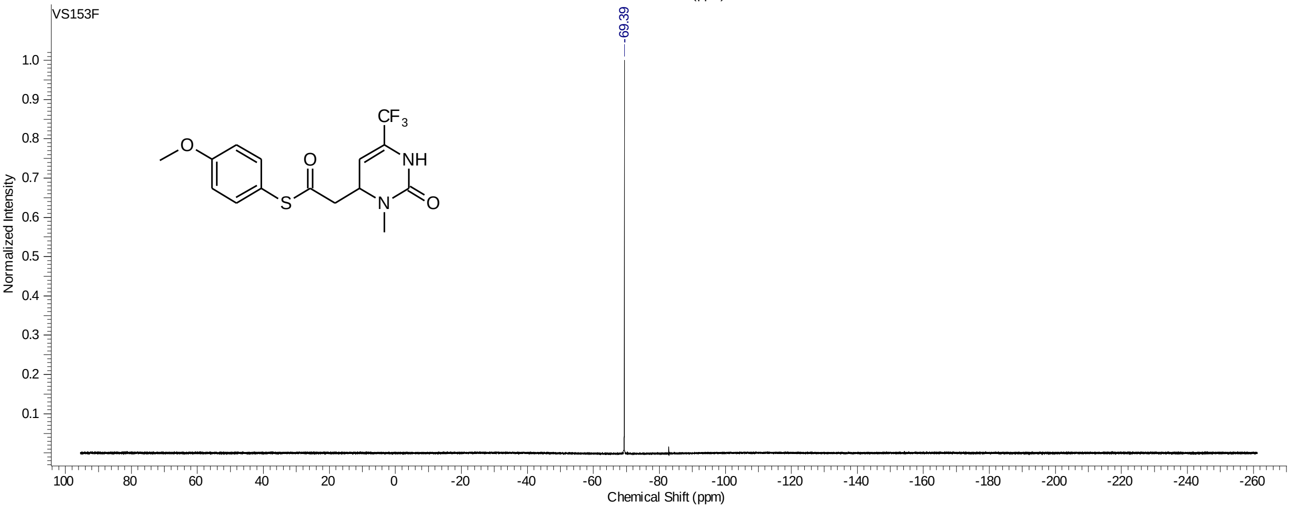
VS153F
Normalized Intensity
Chemical Shift (ppm)
-69.39

Compound **8b**

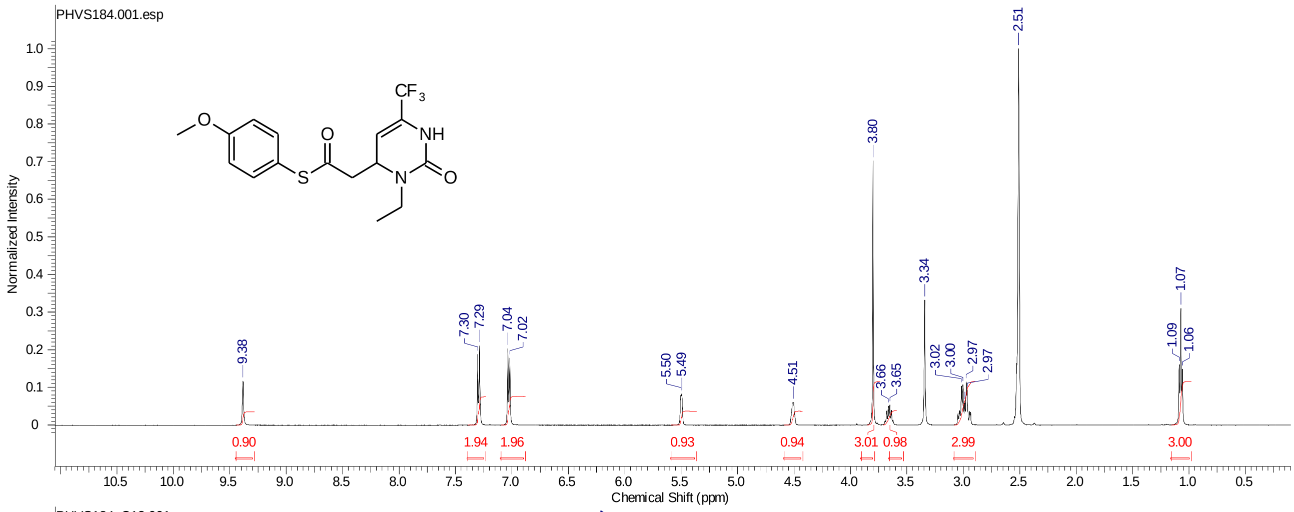
PHVS184.001.esp
Normalized Intensity
Chemical Shift (ppm)
CF3
NH
O
O
S
N
O
9.38
7.30
7.29
7.04
7.02
5.50
5.49
4.51
3.80
3.66
3.65
3.34
3.02
3.00
2.97
2.97
2.51
1.09
1.07
1.06
0.90
1.94
1.96
0.93
0.94
3.01
0.98
2.99
3.00

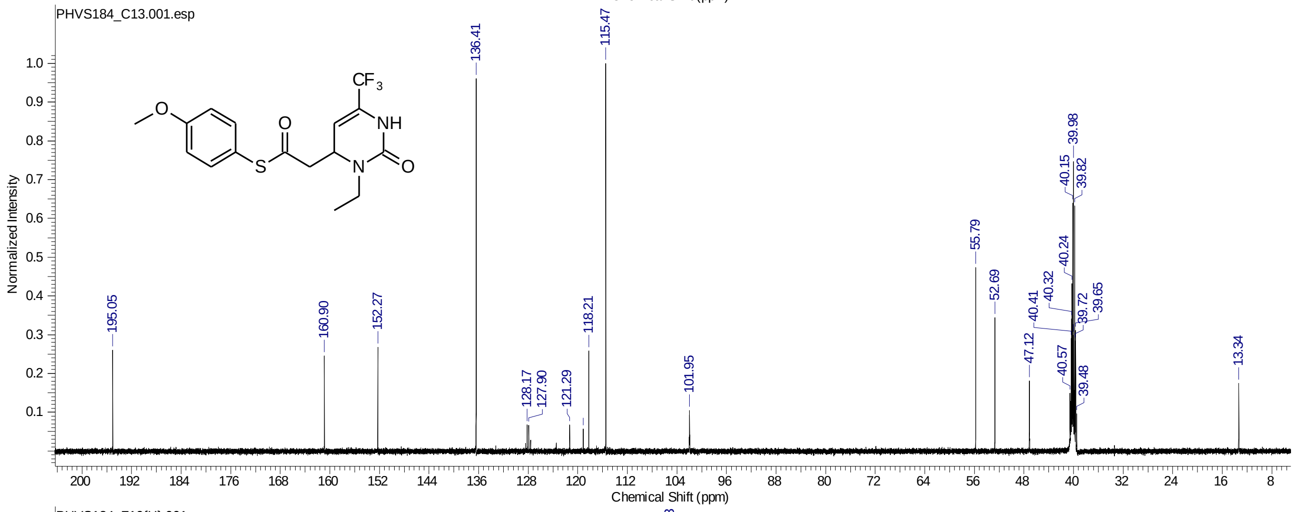
PHVS184_C13.001.esp
Normalized Intensity
Chemical Shift (ppm)
CF3
NH
O
O
S
N
O
195.05
160.90
152.27
136.41
128.17
127.90
121.29
118.21
115.47
101.95
55.79
52.69
47.12
40.57
40.41
40.32
40.24
40.15
39.98
39.82
39.72
39.65
39.48
13.34

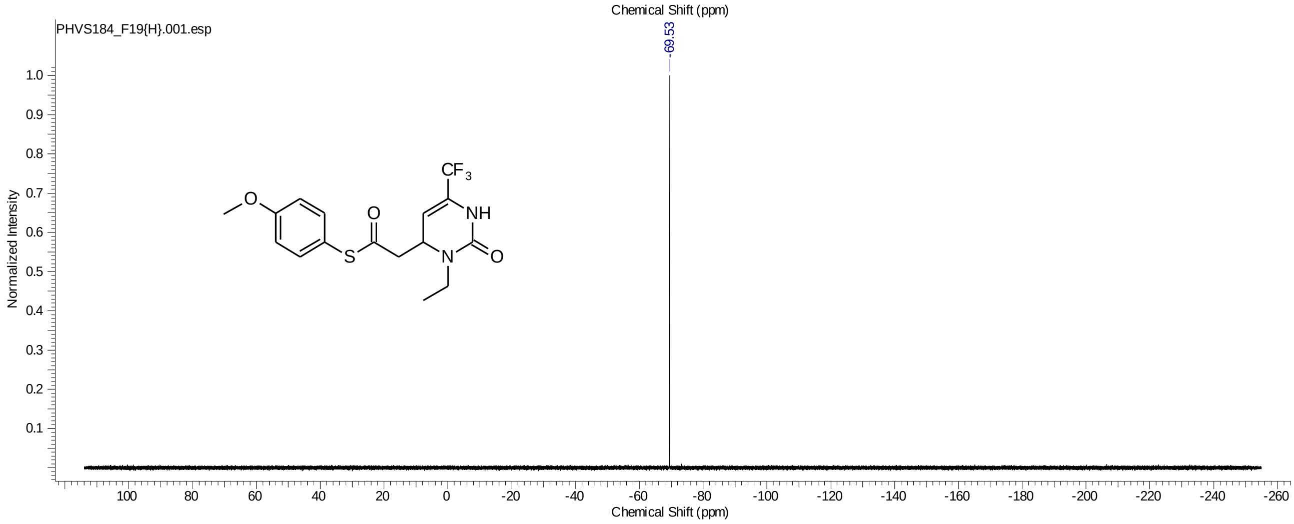
PHVS184_F19{H}.001.esp
Normalized Intensity
Chemical Shift (ppm)
CF3
NH
O
O
S
N
O
-69.53

Compound **8c**

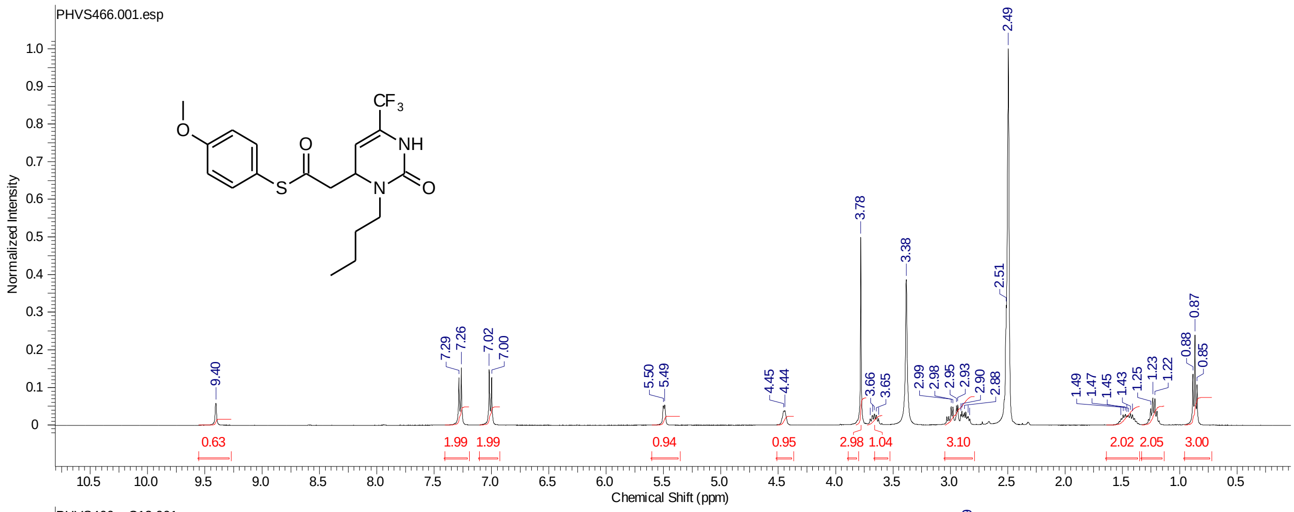
PHVS466.001.esp
Normalized Intensity
Chemical Shift (ppm)
CF3
NH
O
S
N
O
9.40
7.29
7.26
7.02
7.00
5.50
5.49
4.45
4.44
3.78
3.66
3.65
3.38
2.99
2.98
2.95
2.93
2.90
2.88
2.51
2.49
1.49
1.47
1.45
1.43
1.25
1.23
1.22
0.88
0.87
0.85
0.63
1.99
1.99
0.94
0.95
2.98
1.04
3.10
2.02
2.05
3.00

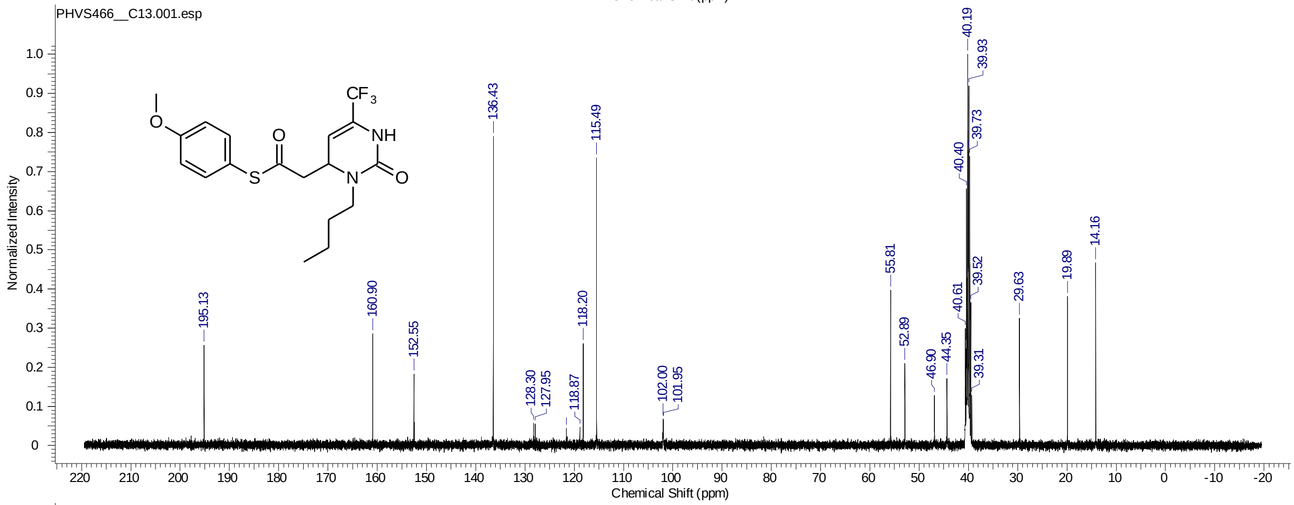
PHVS466__C13.001.esp
Normalized Intensity
Chemical Shift (ppm)
195.13
160.90
152.55
136.43
128.30
127.95
118.87
118.20
115.49
102.00
101.95
55.81
52.89
46.90
44.35
40.61
40.40
40.19
39.93
39.73
39.52
39.31
29.63
19.89
14.16

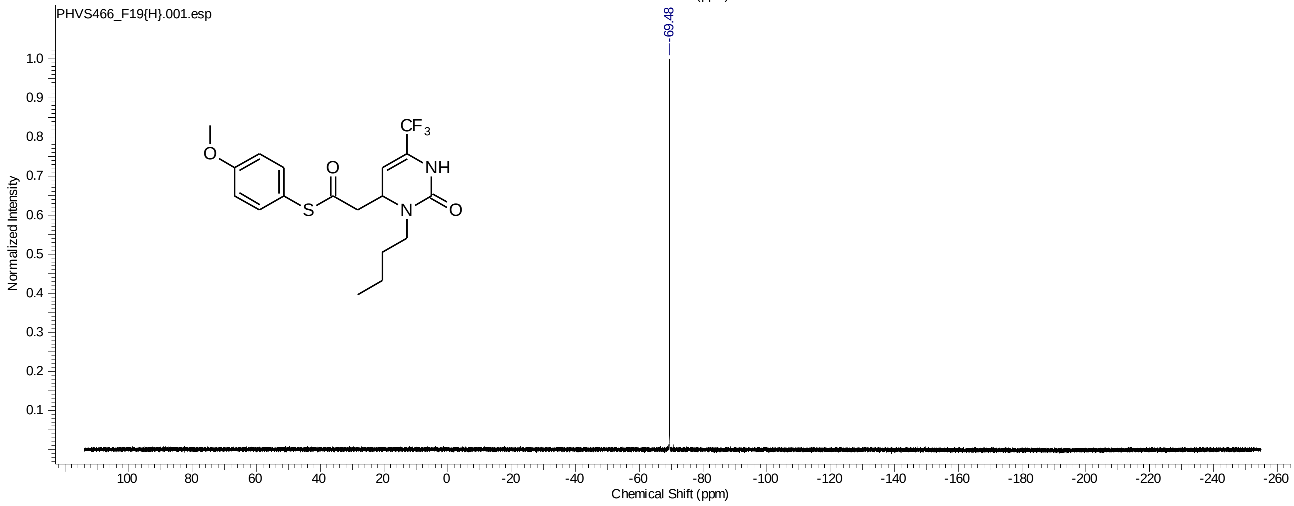
PHVS466_F19{H}.001.esp
Normalized Intensity
Chemical Shift (ppm)
-69.48

Compound **8d**

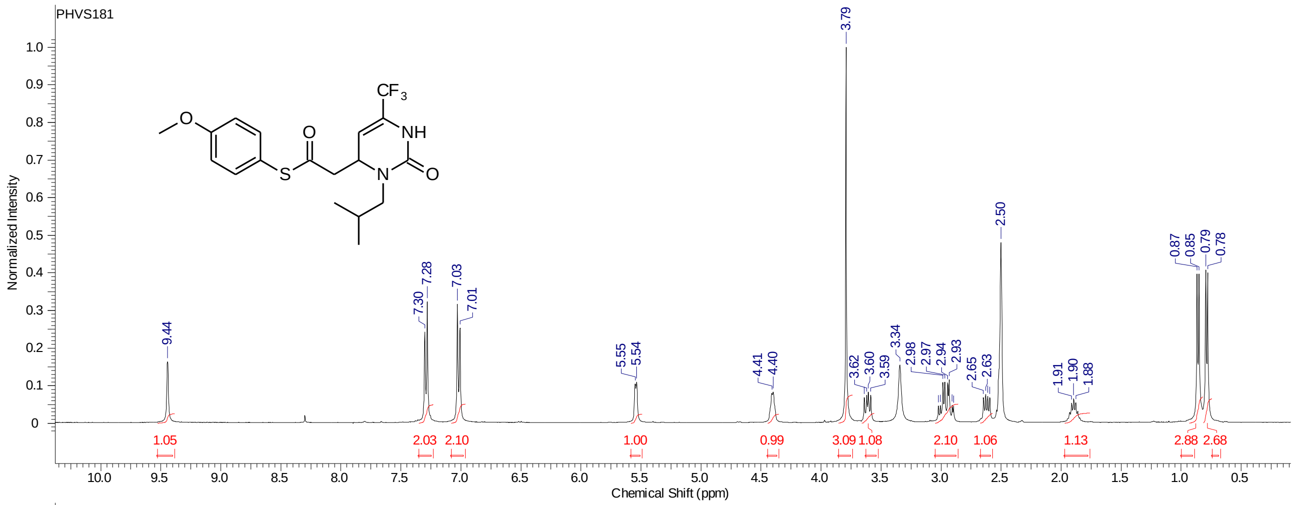
PHVS181
Normalized Intensity
Chemical Shift (ppm)
9.44
7.30
7.28
7.03
7.01
5.55
5.54
4.41
4.40
3.79
3.62
3.60
3.59
3.34
2.98
2.97
2.94
2.93
2.65
2.63
2.50
1.91
1.90
1.88
0.87
0.85
0.79
0.78
1.05
2.03
2.10
1.00
0.99
3.09
1.08
2.10
1.06
1.13
2.88
2.68

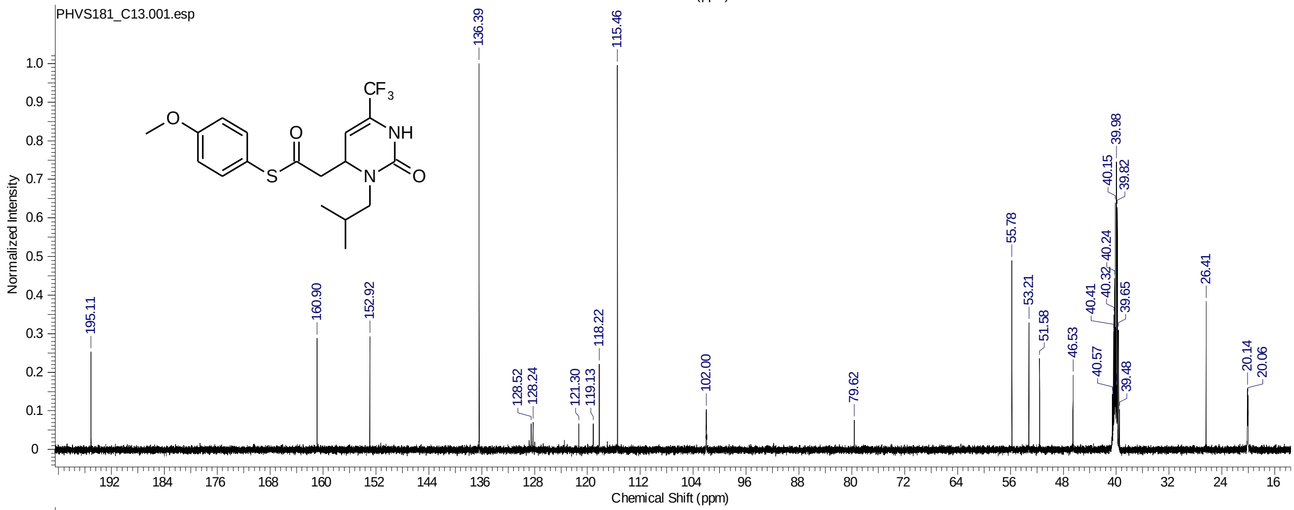
PHVS181_C13.001.esp
Normalized Intensity
Chemical Shift (ppm)
195.11
160.90
152.92
136.39
128.52
128.24
121.30
119.13
118.22
115.46
102.00
79.62
55.78
53.21
51.58
46.53
40.57
40.41
40.32
40.24
40.15
39.98
39.82
39.65
39.48
26.41
20.14
20.06

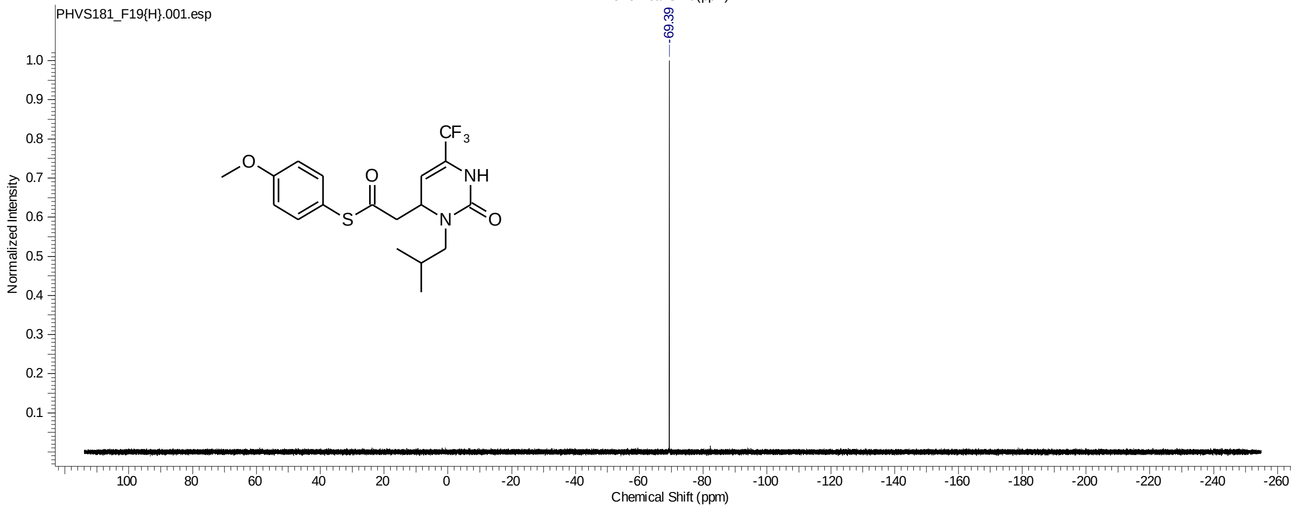
PHVS181_F19{H}.001.esp
Normalized Intensity
Chemical Shift (ppm)
-69.39

Compound **8e**

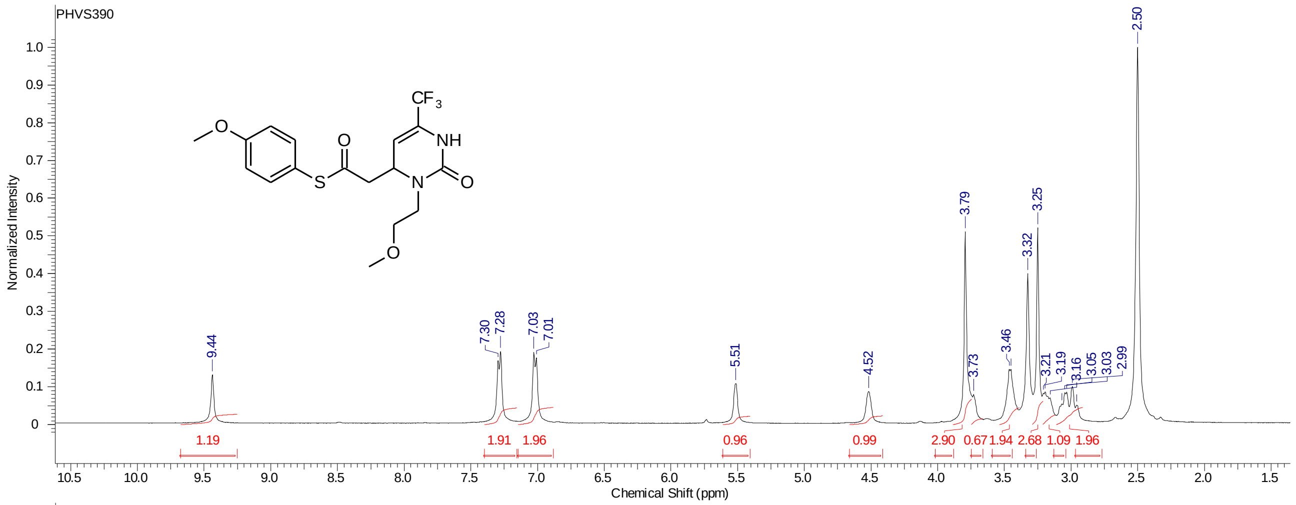
PHVS390
Normalized Intensity
Chemical Shift (ppm)
9.44
7.30
7.28
7.03
7.01
5.51
4.52
3.79
3.73
3.46
3.32
3.25
3.21
3.19
3.16
3.05
3.03
2.99
2.50
1.19
1.91
1.96
0.96
0.99
2.90
0.67
1.94
2.68
1.09
1.96
CF3
NH
O
S
N

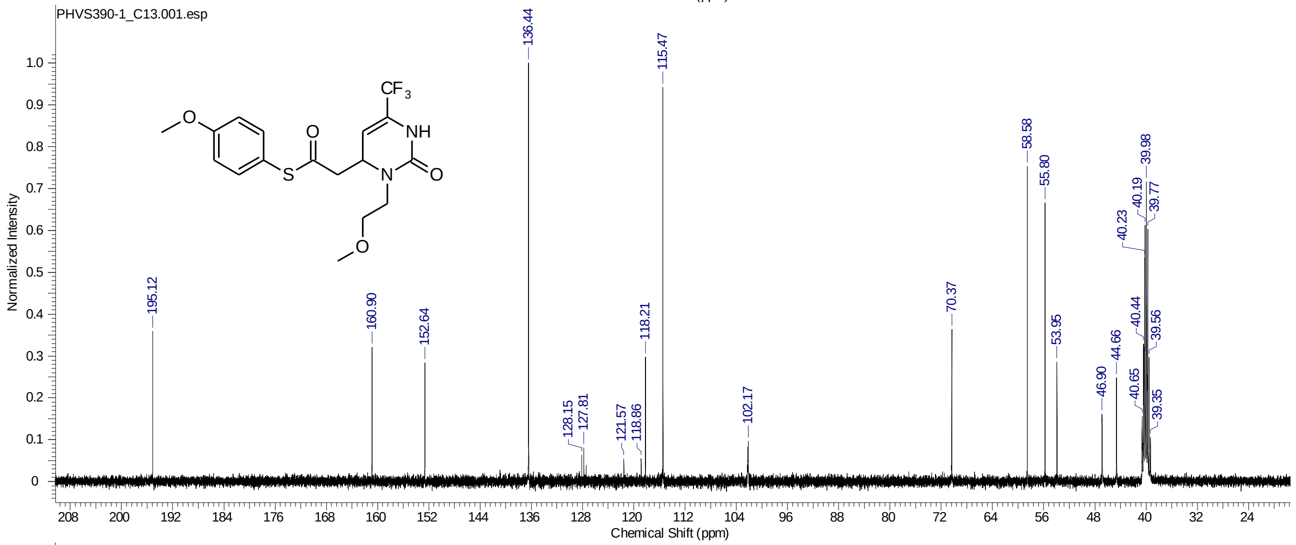
PHVS390-1_C13.001.esp
Normalized Intensity
Chemical Shift (ppm)
195.12
160.90
152.64
136.44
128.15
127.81
121.57
118.86
118.21
115.47
102.17
70.37
58.58
55.80
53.95
46.90
44.66
40.65
40.44
40.23
40.19
39.98
39.77
39.56
39.35

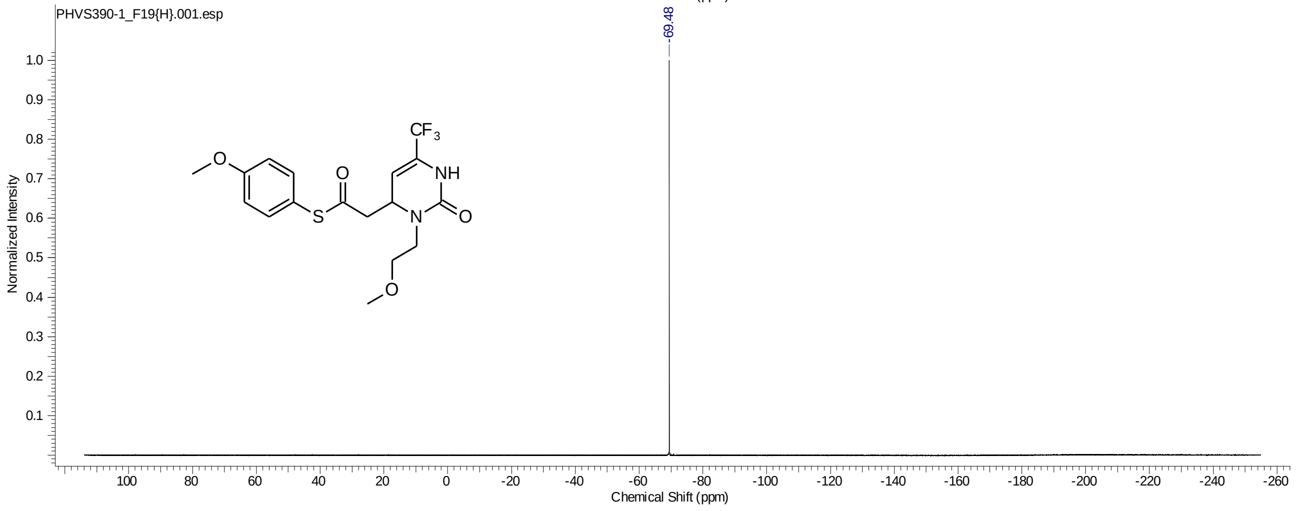
PHVS390-1_F19{H}.001.esp
Normalized Intensity
Chemical Shift (ppm)
-69.48

Compound **8f**

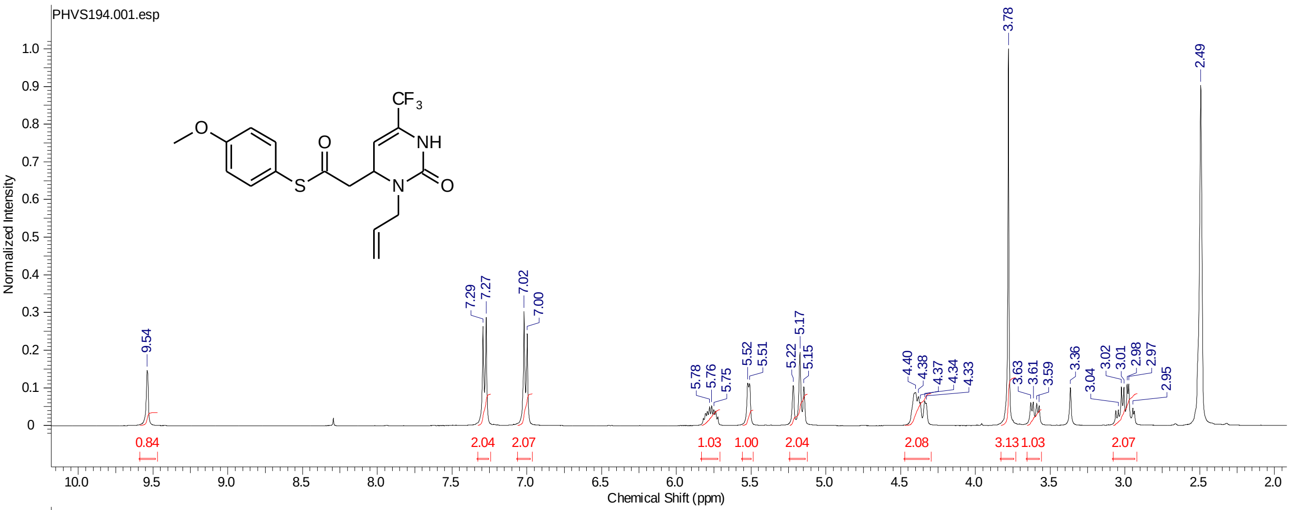
PHVS194.001.esp
Normalized Intensity
Chemical Shift (ppm)
CF3
NH
O
S
N
O
9.54
7.29
7.27
7.02
7.00
5.78
5.76
5.75
5.52
5.51
5.22
5.17
5.15
4.40
4.38
4.37
4.34
4.33
3.78
3.63
3.61
3.59
3.36
3.04
3.02
3.01
2.98
2.97
2.95
2.49
0.84
2.04
2.07
1.03
1.00
2.04
2.08
3.13
1.03
2.07

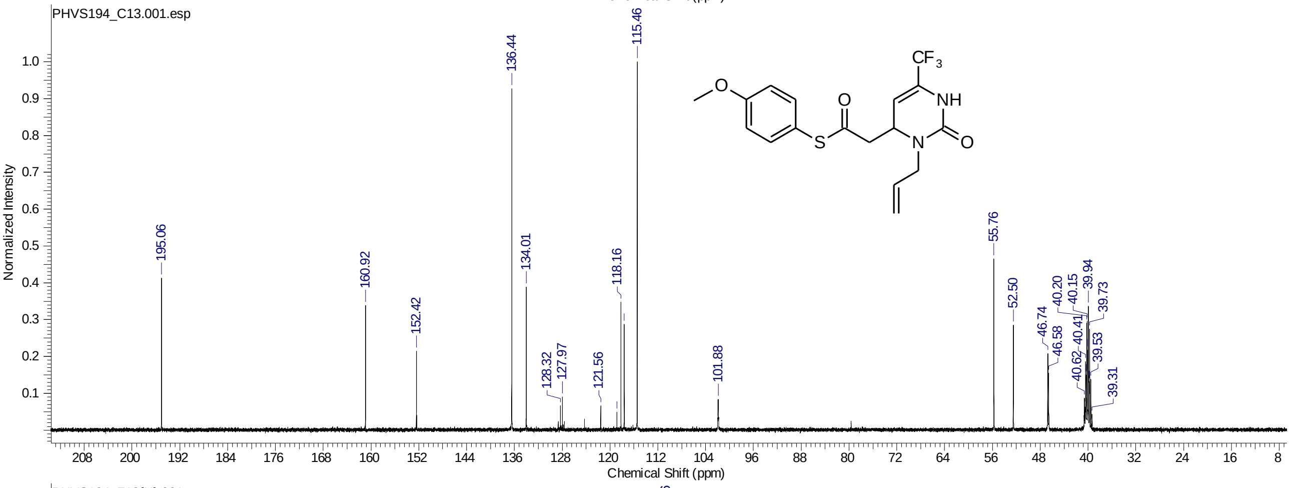
PHVS194_C13.001.esp
Normalized Intensity
Chemical Shift (ppm)
195.06
160.92
152.42
136.44
134.01
128.32
127.97
121.56
118.16
115.46
101.88
55.76
52.50
46.74
46.58
40.62
40.41
40.20
40.15
39.94
39.73
39.53
39.31

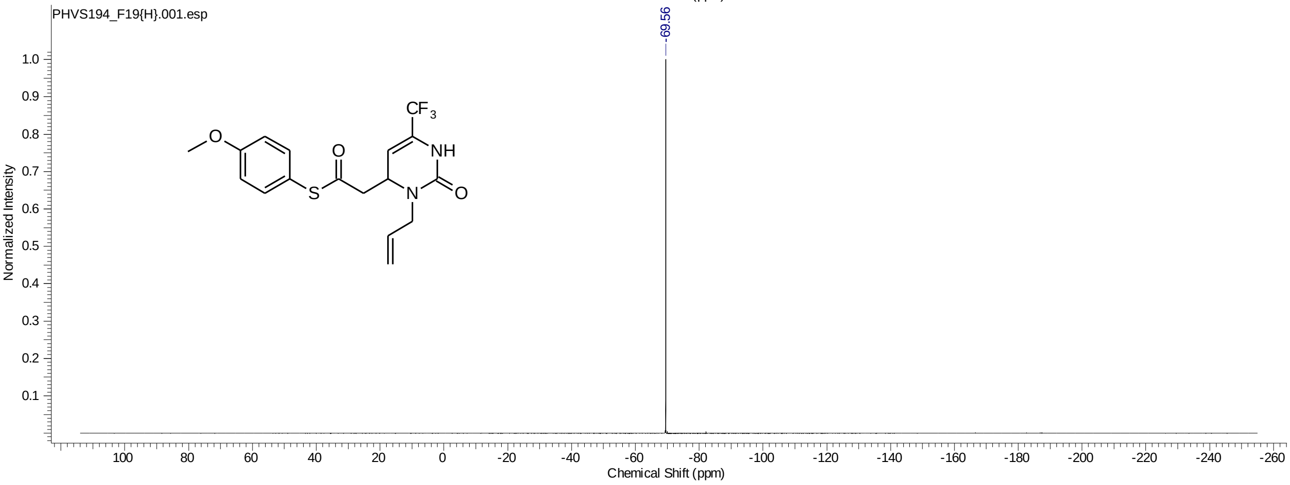
PHVS194_F19{H}.001.esp
Normalized Intensity
Chemical Shift (ppm)
-69.56

Compound **8g**

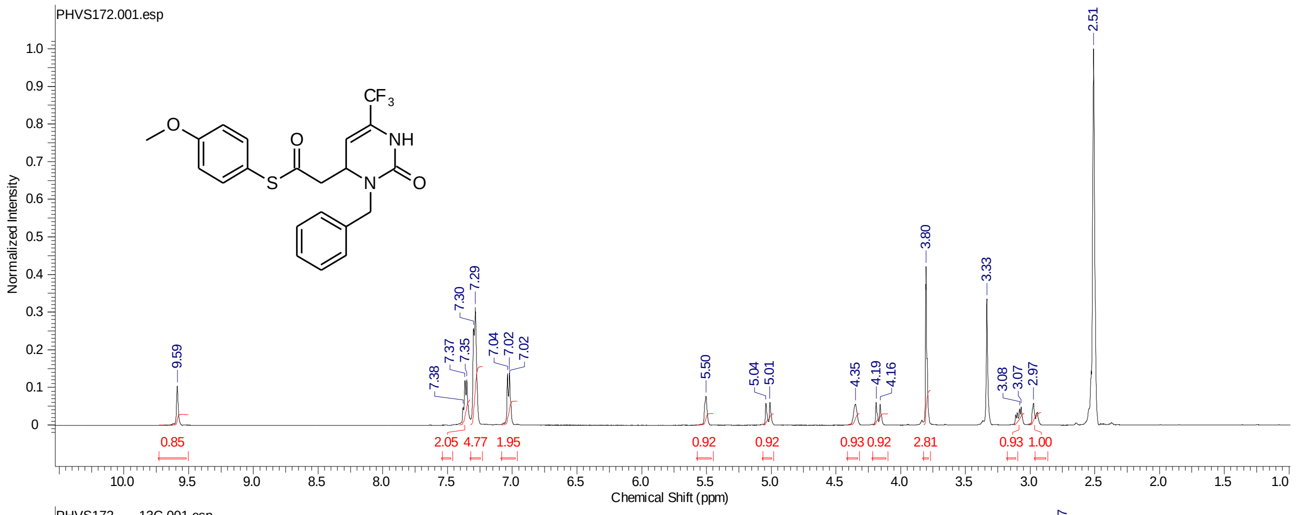

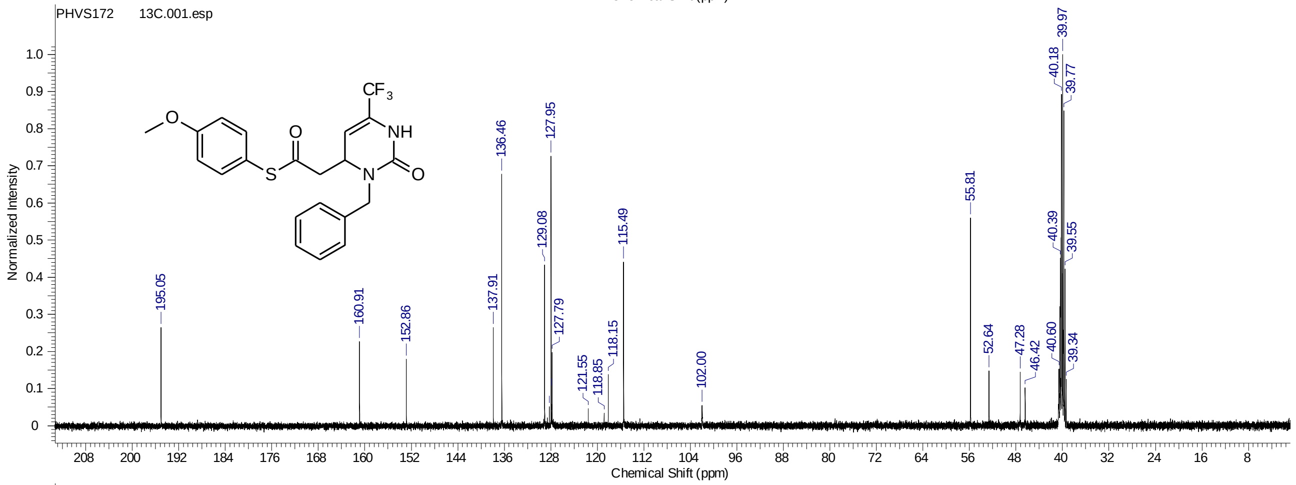

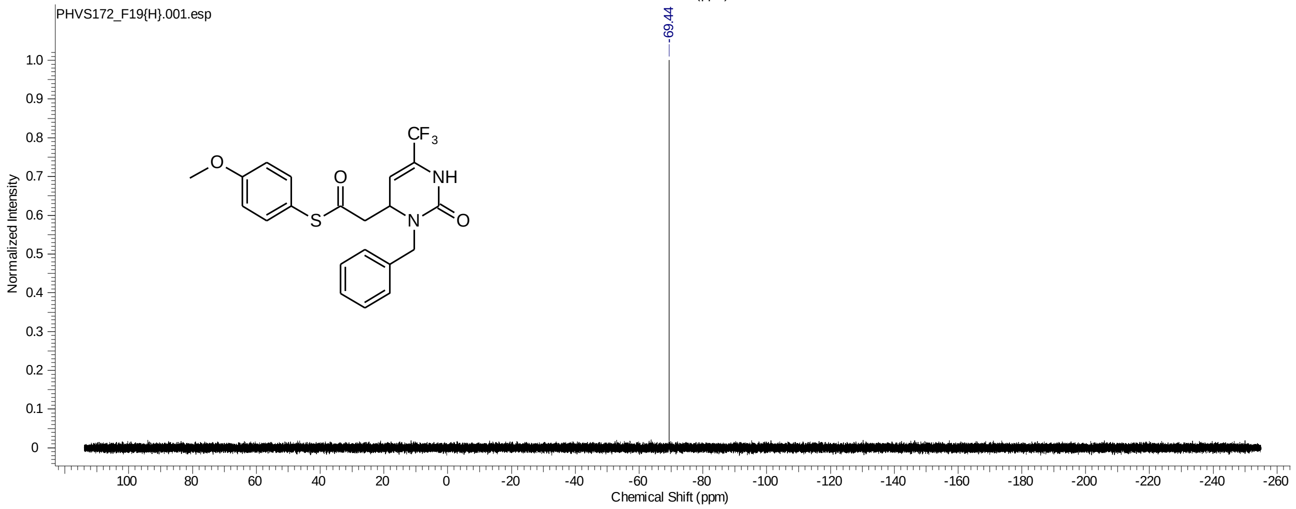

Compound **8h**

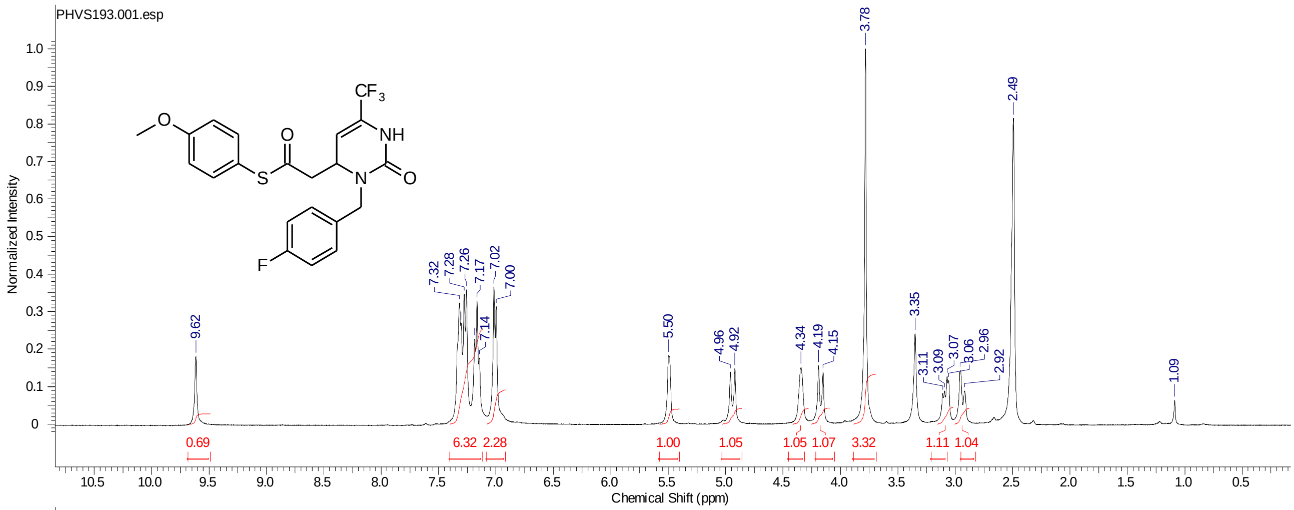
PHVS193.001.esp
Normalized Intensity
Chemical Shift (ppm)
9.62
7.32
7.28
7.26
7.17
7.14
7.02
7.00
5.50
4.96
4.92
4.34
4.19
4.15
3.78
3.35
3.11
3.09
3.07
3.06
2.96
2.92
2.49
1.09
0.69
6.32
2.28
1.00
1.05
1.05
1.07
3.32
1.11
1.04
CF3
NH
O
N
S
F

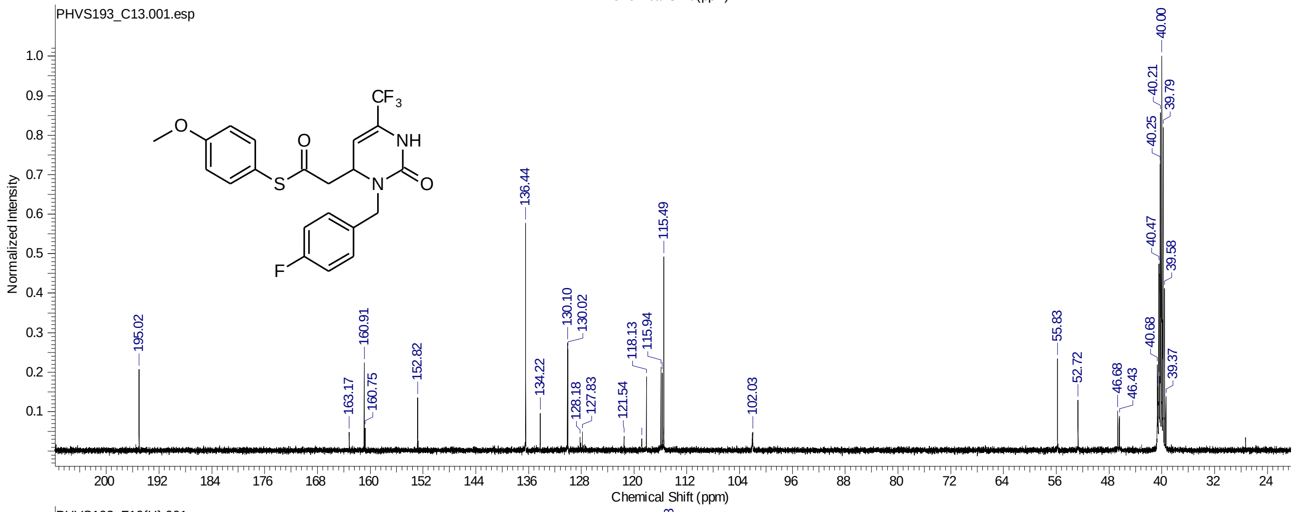
PHVS193_C13.001.esp
Normalized Intensity
Chemical Shift (ppm)
195.02
163.17
160.91
160.75
152.82
136.44
134.22
130.10
130.02
128.18
127.83
121.54
118.13
115.94
115.49
102.03
55.83
52.72
46.68
46.43
40.68
40.47
40.25
40.21
40.00
39.79
39.58
39.37

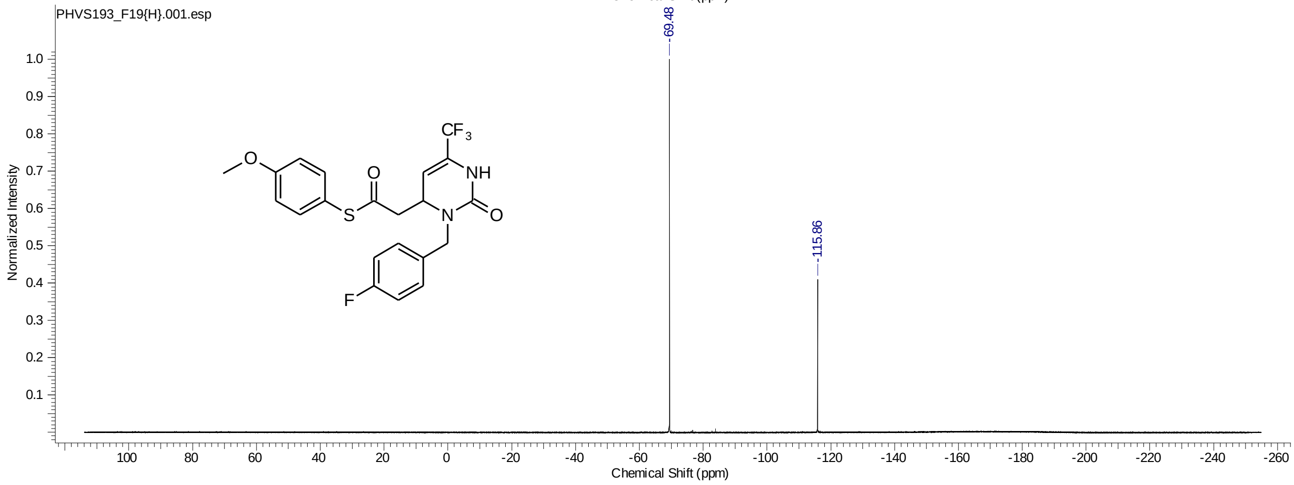
PHVS193_F19{H}.001.esp
Normalized Intensity
Chemical Shift (ppm)
-69.48
-115.86

Compound **8i**

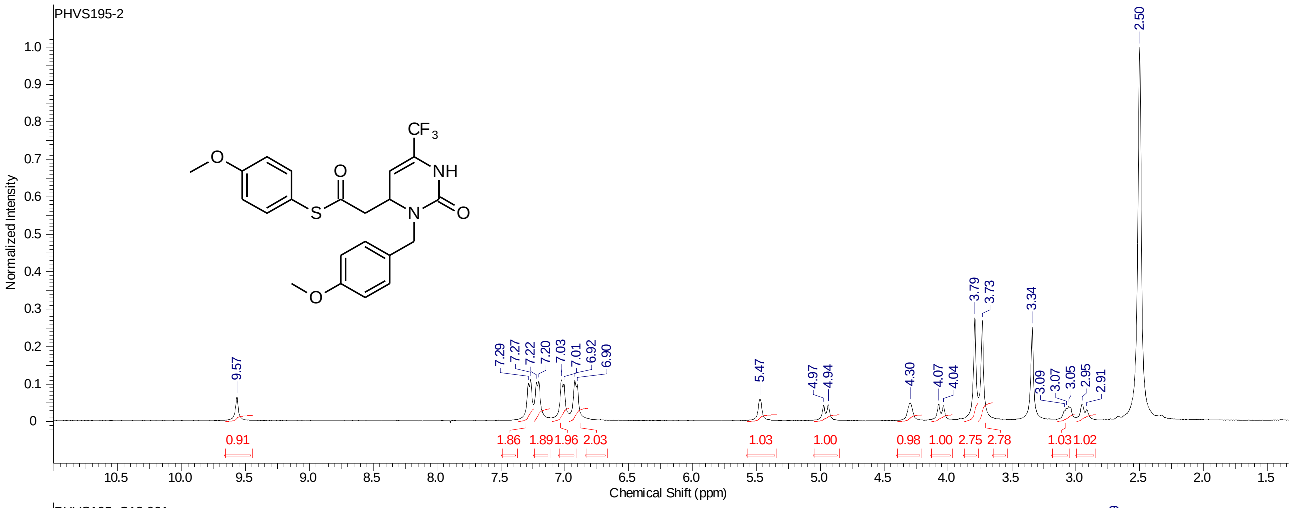
PHVS195-2
Normalized Intensity
Chemical Shift (ppm)
CF3
O
NH
O
S
N
O
O
9.57
7.29 7.27 7.22 7.20 7.03 7.01 6.92 6.90
5.47
4.97 4.94
4.30
4.07 4.04
3.79 3.73
3.34
3.09 3.07 3.05 2.95 2.91
2.50
0.91 1.86 1.89 1.96 2.03 1.03 1.00 0.98 1.00 2.75 2.78 1.03 1.02

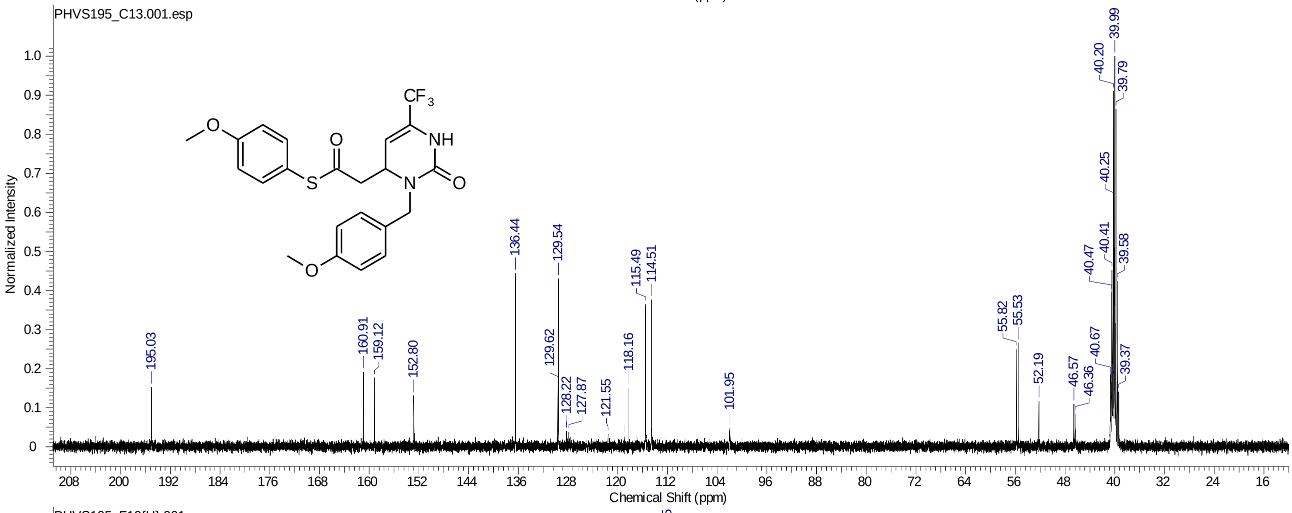
PHVS195_C13.001.esp
Normalized Intensity
Chemical Shift (ppm)
CF3
O
NH
O
S
N
O
O
195.03
160.91 159.12
152.80
136.44
129.62 129.54
128.22 127.87
121.55
118.16
115.49 114.51
101.95
55.82 55.53
52.19
46.57 46.36
40.67 40.47 40.41 40.25 40.20 39.99 39.79 39.58 39.37

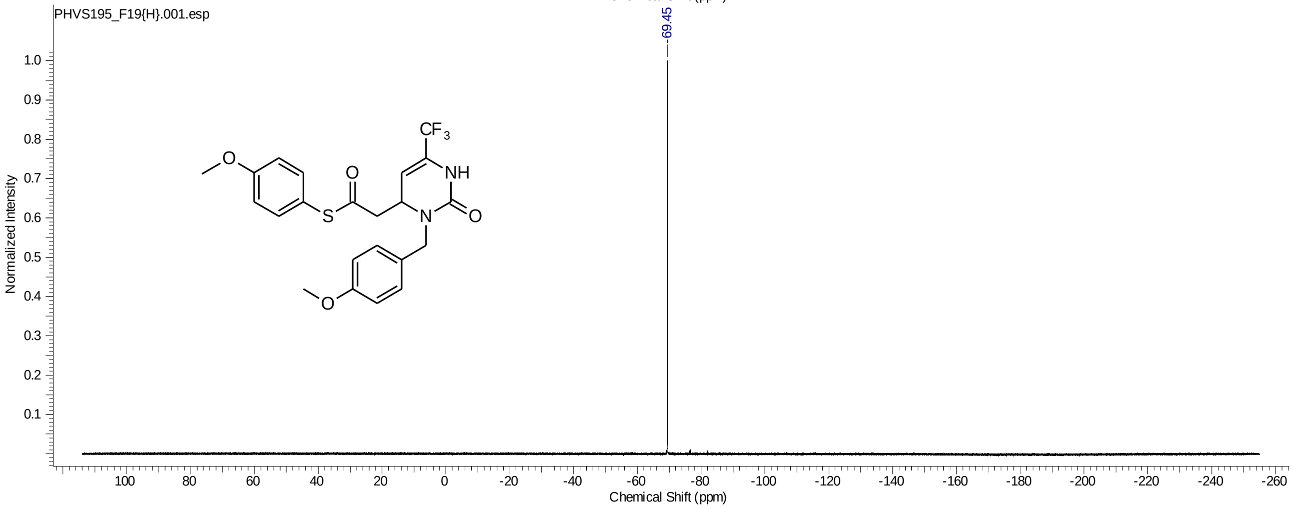
PHVS195_F19{H}.001.esp
Normalized Intensity
Chemical Shift (ppm)
CF3
O
NH
O
S
N
O
O
-69.45

Compound **8j**

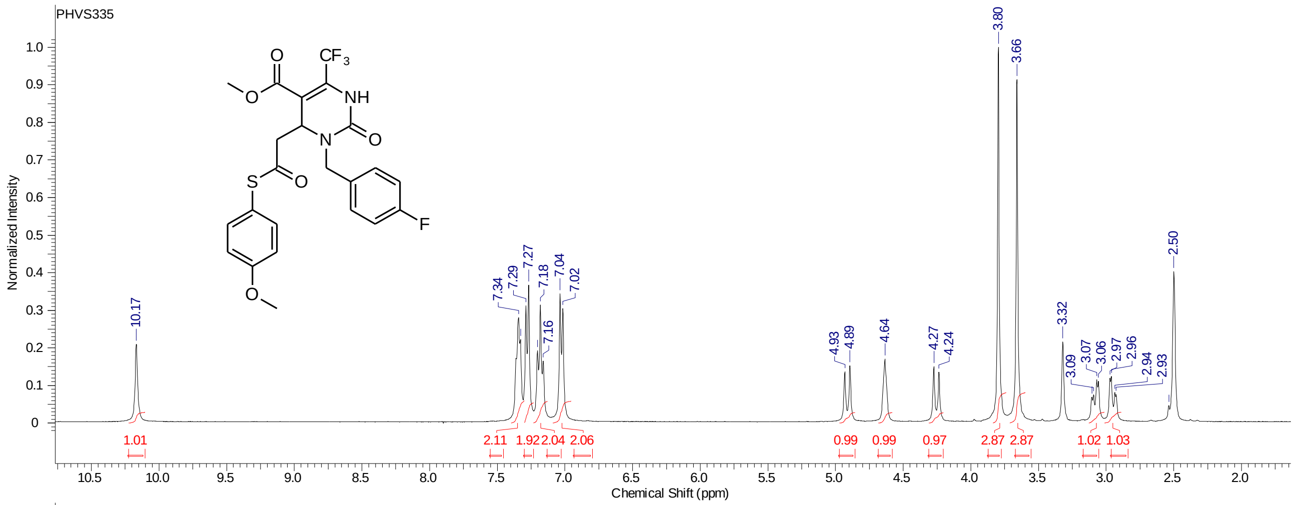

PHVS335
Normalized Intensity
Chemical Shift (ppm)
10.17
7.34 7.29 7.27 7.18 7.16 7.04 7.02
4.93 4.89 4.64 4.27 4.24
3.80 3.66 3.32
3.09 3.07 3.06 2.97 2.96 2.94 2.93
2.50
1.01 2.11 1.92 2.04 2.06 0.99 0.99 0.97 2.87 2.87 1.02 1.03

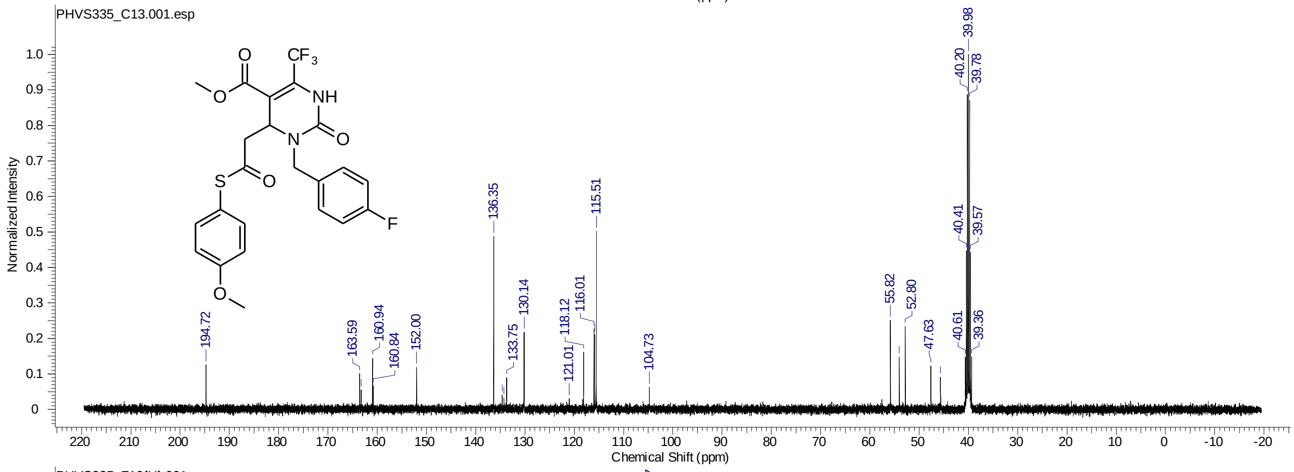

PHVS335_C13.001.esp
Normalized Intensity
Chemical Shift (ppm)
194.72
163.59 160.94 160.84 152.00
136.35 133.75 130.14
121.01 118.12 116.01 115.51
104.73
55.82 52.80 47.63
40.61 40.41 40.20 39.98 39.78 39.57 39.36

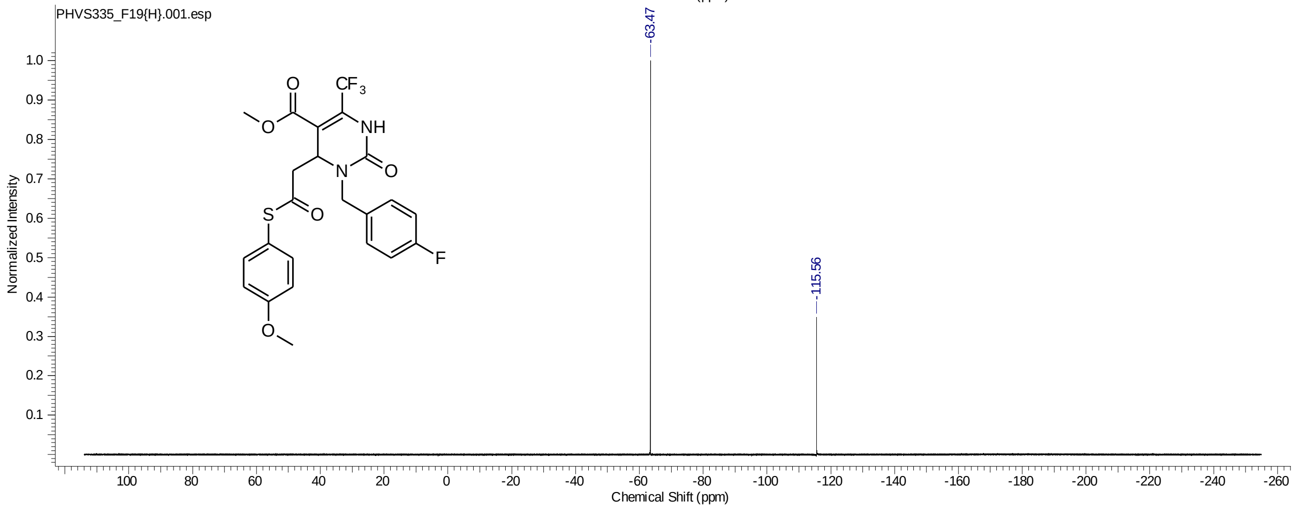

PHVS335_F19{H}.001.esp
Normalized Intensity
Chemical Shift (ppm)
-63.47
-115.56

Compound **8k**

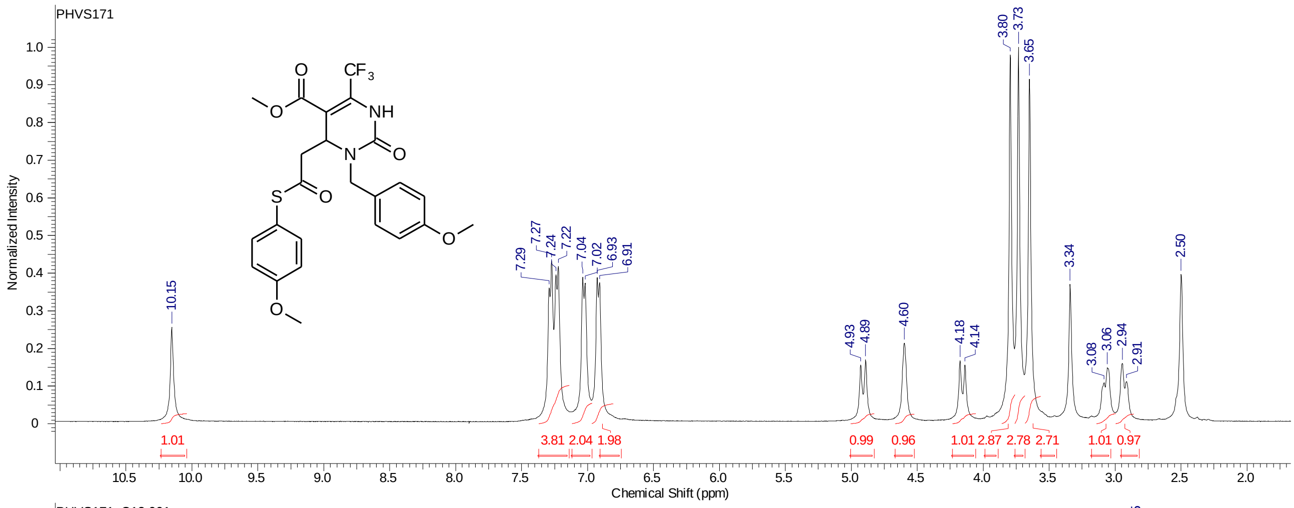
PHVS171
Normalized Intensity
Chemical Shift (ppm)
10.15
7.29 7.27 7.24 7.22 7.04 7.02 6.93 6.91
4.93 4.89 4.60 4.18 4.14 3.80 3.73 3.65 3.34 3.08 3.06 2.94 2.91 2.50
1.01 3.81 2.04 1.98 0.99 0.96 1.01 2.87 2.78 2.71 1.01 0.97

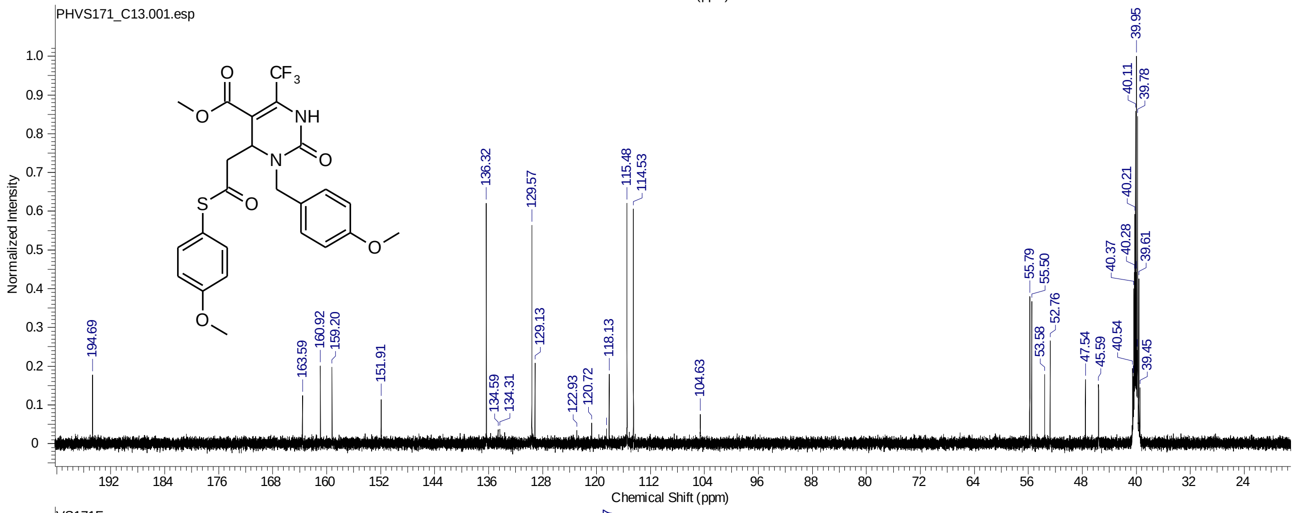
PHVS171_C13.001.esp
Normalized Intensity
Chemical Shift (ppm)
194.69 163.59 160.92 159.20 151.91 136.32 134.59 134.31 129.57 129.13 122.93 120.72 118.13 115.48 114.53 104.63
55.79 55.50 53.58 52.76 47.54 45.59 40.54 40.37 40.28 40.21 40.11 39.95 39.78 39.61 39.45

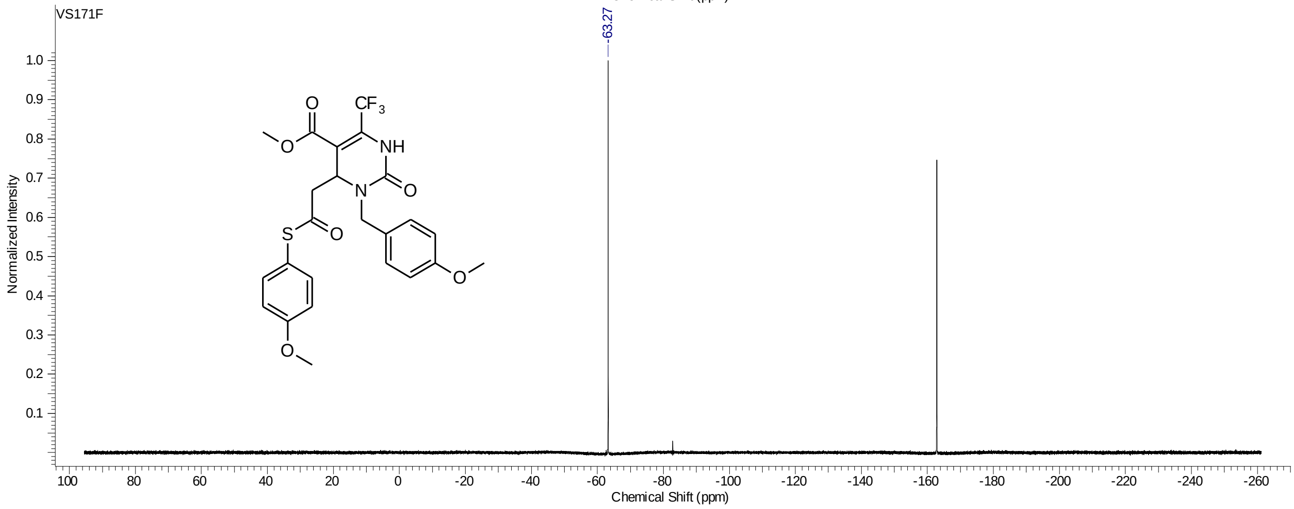
VS171F
Normalized Intensity
Chemical Shift (ppm)
-63.27

Compound **8l**

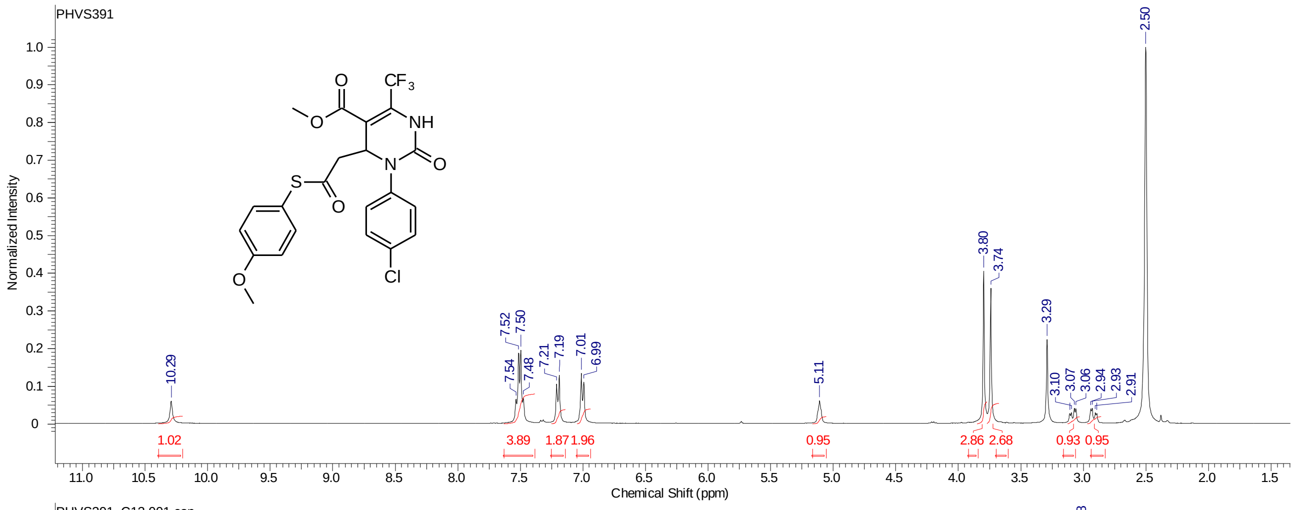

PHVS391
Normalized Intensity
Chemical Shift (ppm)
10.29
7.54
7.52
7.50
7.48
7.21
7.19
7.01
6.99
5.11
3.80
3.74
3.29
3.10
3.07
3.06
2.94
2.93
2.91
2.50
1.02
3.89
1.87
1.96
0.95
2.86
2.68
0.93
0.95

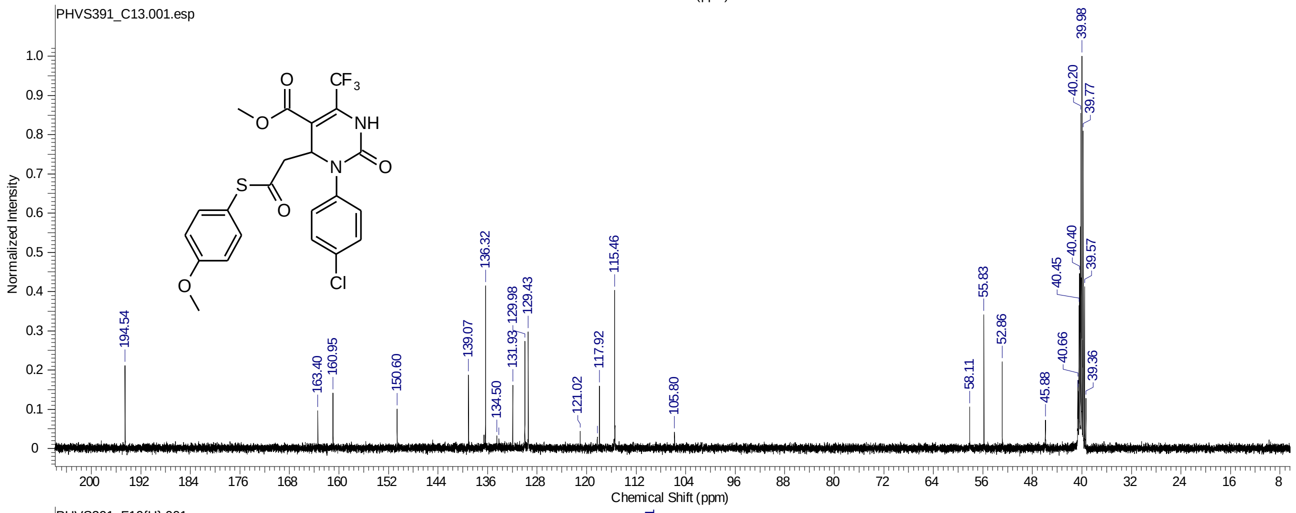

PHVS391_C13.001.esp
Normalized Intensity
Chemical Shift (ppm)
194.54
163.40
160.95
150.60
139.07
136.32
134.50
131.93
129.98
129.43
121.02
117.92
115.46
105.80
58.11
55.83
52.86
45.88
40.66
40.45
40.40
40.20
39.98
39.77
39.57
39.36

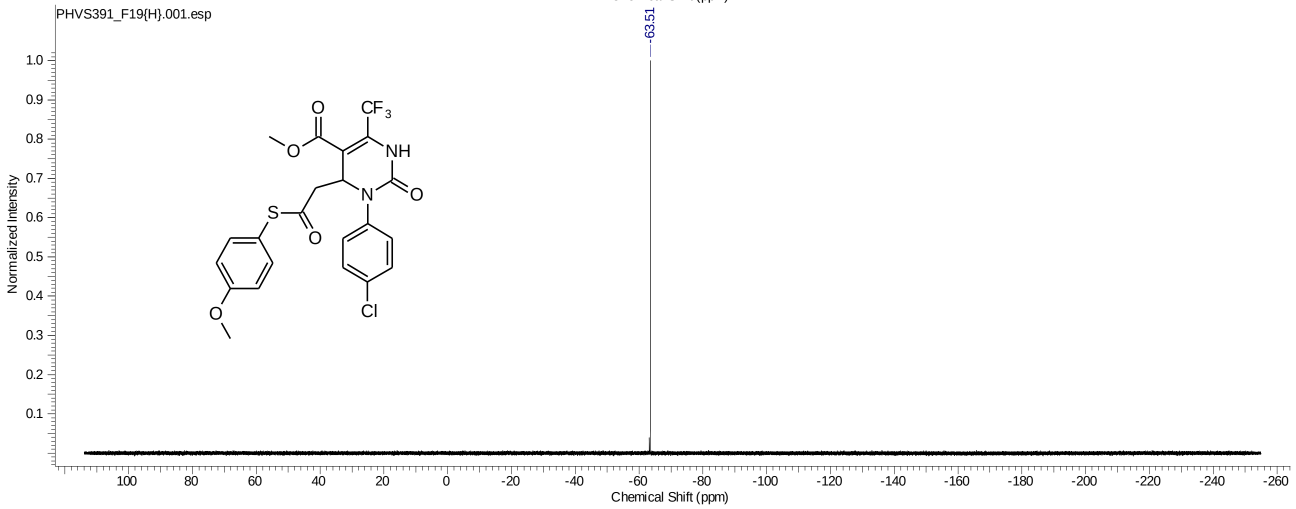

PHVS391_F19{H}.001.esp
Normalized Intensity
Chemical Shift (ppm)
-63.51

Compound **8m**

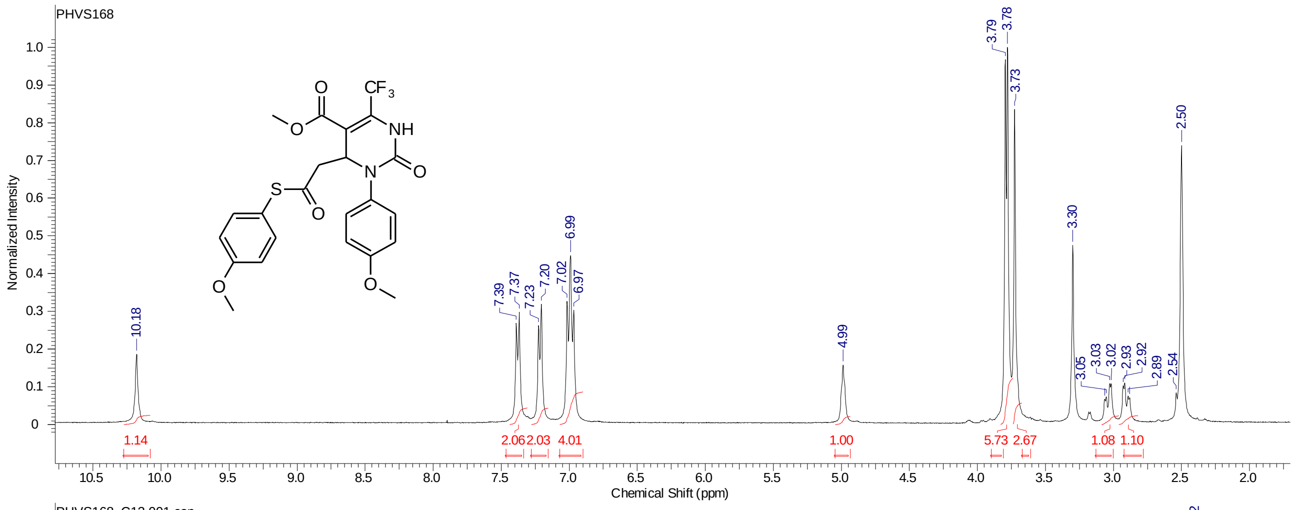
PHVS168
Normalized Intensity
Chemical Shift (ppm)
10.18
7.39 7.37 7.23 7.20 7.02 6.99 6.97
4.99
3.79 3.78 3.73
3.30
3.05 3.03 3.02 2.93 2.92 2.89 2.54
2.50
1.14
2.06 2.03 4.01
1.00
5.73 2.67
1.08 1.10

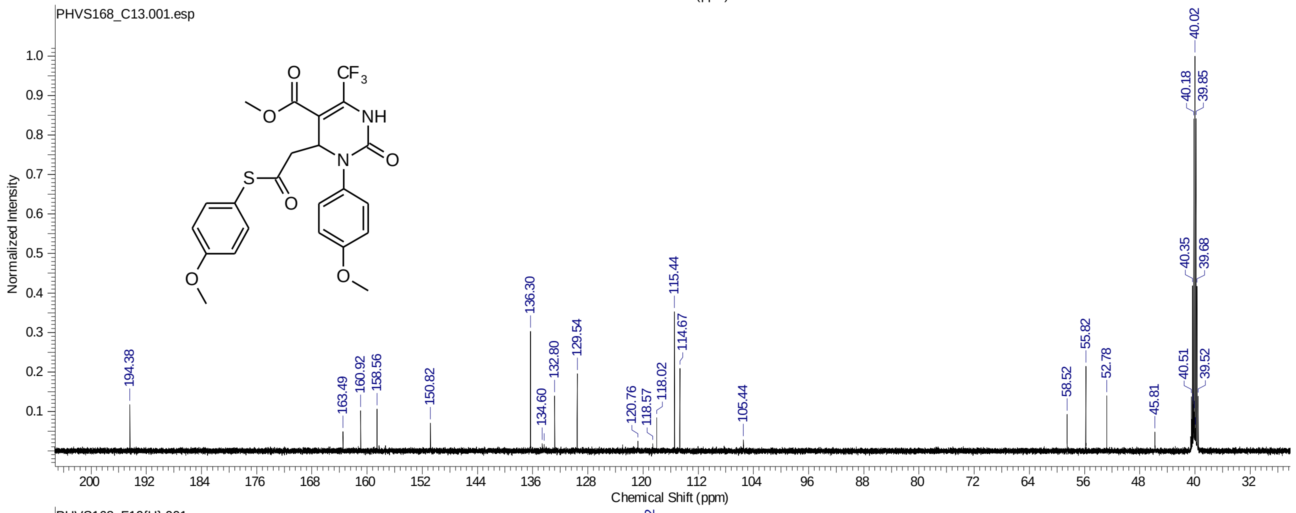
PHVS168_C13.001.esp
Normalized Intensity
Chemical Shift (ppm)
194.38
163.49 160.92 158.56
150.82
136.30 134.60 132.80 129.54
120.76 118.57 118.02 115.44 114.67
105.44
58.52 55.82 52.78
45.81
40.51 40.35 40.18 40.02 39.85 39.68 39.52

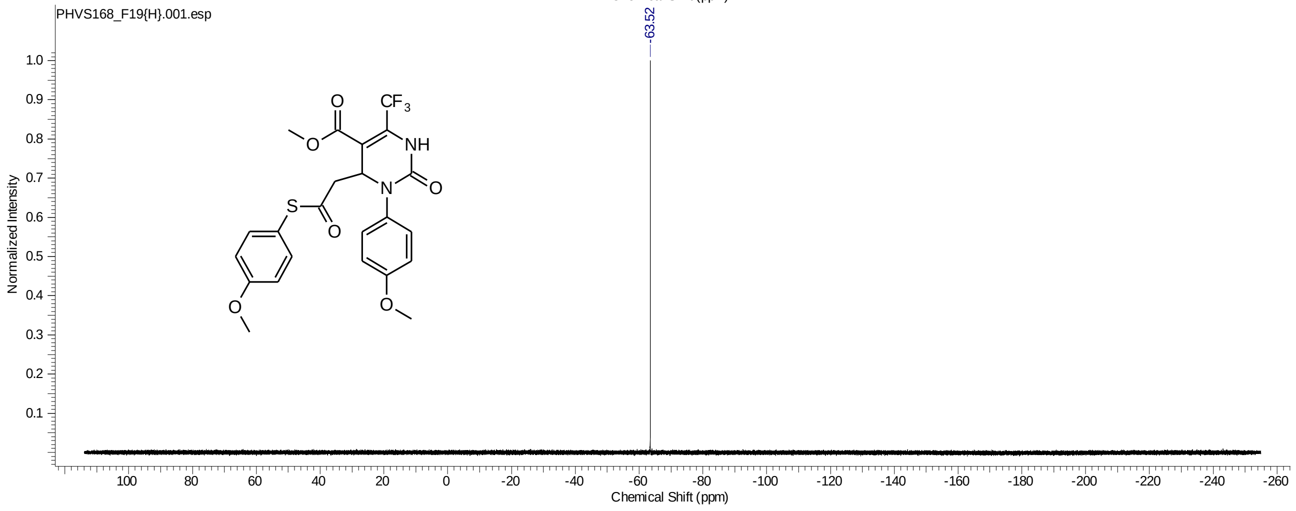
PHVS168_F19{H}.001.esp
Normalized Intensity
Chemical Shift (ppm)
-63.52

Compound **9a**

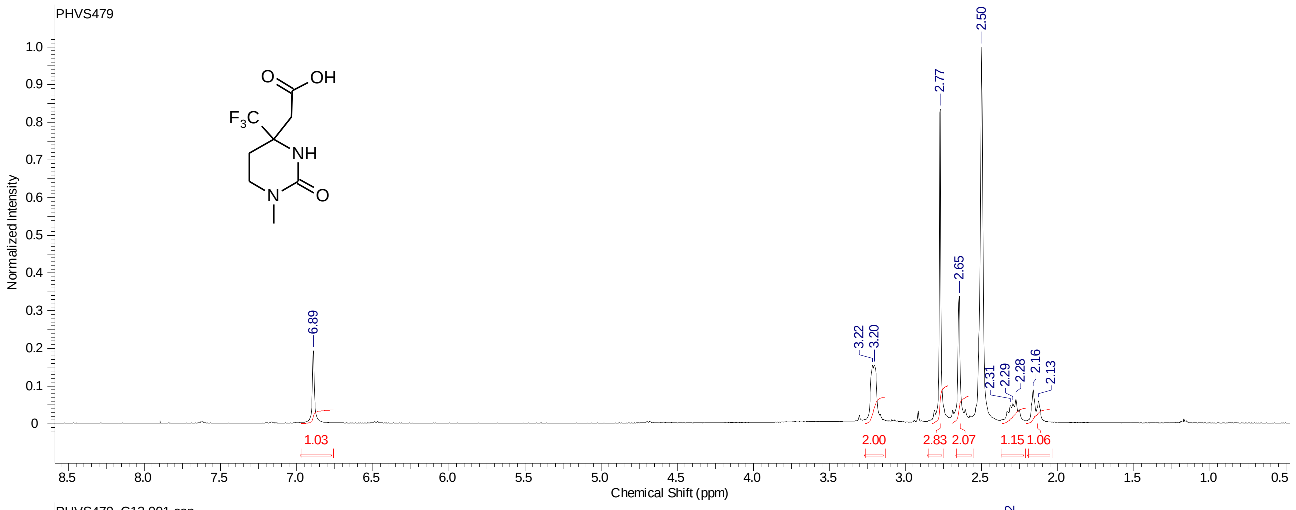
PHVS479
Normalized Intensity
Chemical Shift (ppm)
6.89
3.22
3.20
2.77
2.65
2.50
2.31
2.29
2.28
2.16
2.13
1.03
2.00
2.83
2.07
1.15
1.06

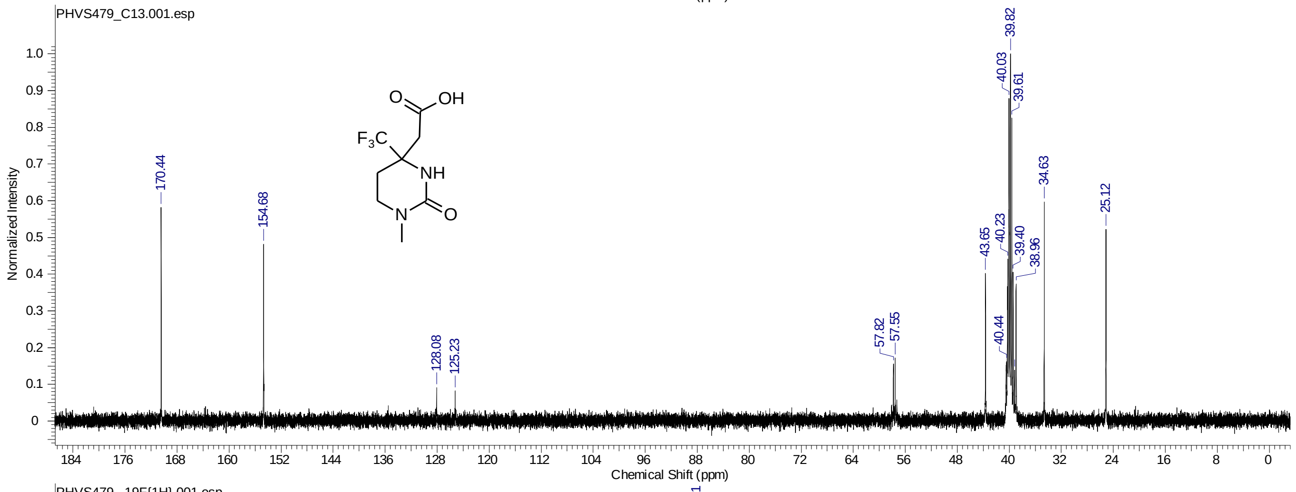
PHVS479_C13.001.esp
Normalized Intensity
Chemical Shift (ppm)
170.44
154.68
128.08
125.23
57.82
57.55
43.65
40.44
40.23
40.03
39.82
39.61
39.40
38.96
34.63
25.12

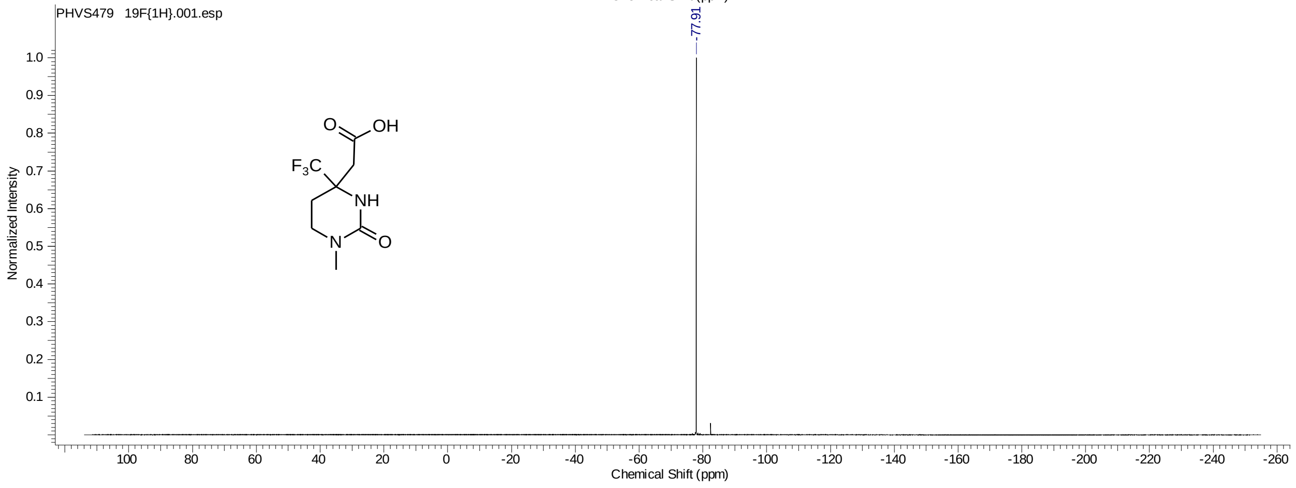
PHVS479 19F{1H}.001.esp
Normalized Intensity
Chemical Shift (ppm)
-77.91

Compound **9b**

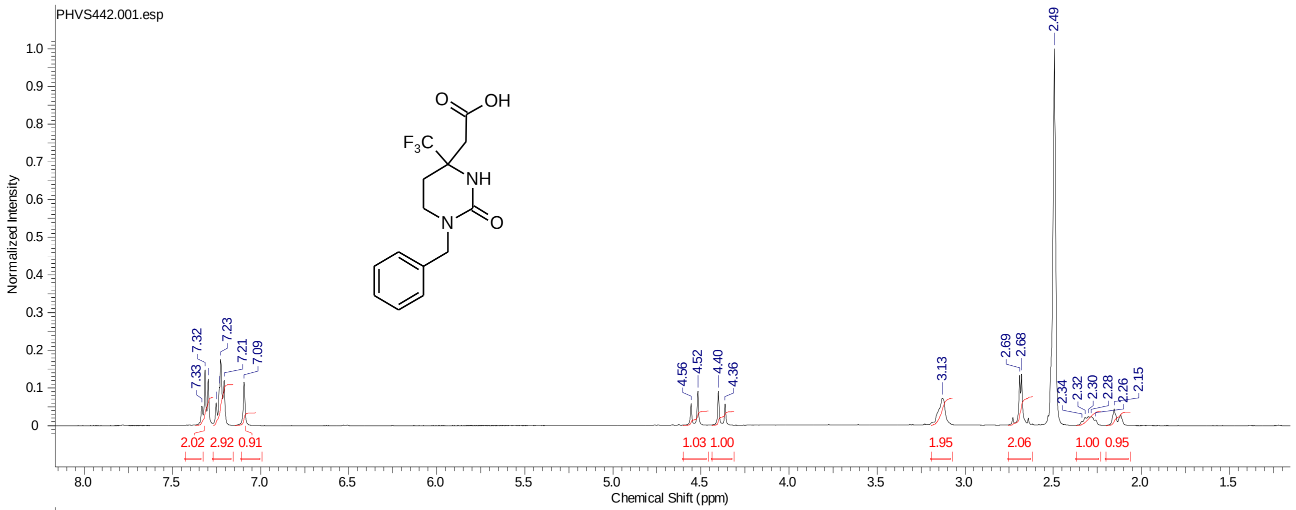
PHVS442.001.esp
Normalized Intensity
Chemical Shift (ppm)
OH
O
F3C
NH
N
O
7.33
7.32
7.23
7.21
7.09
4.56
4.52
4.40
4.36
3.13
2.69
2.68
2.49
2.34
2.32
2.30
2.28
2.26
2.15
2.02
2.92
0.91
1.03
1.00
1.95
2.06
1.00
0.95

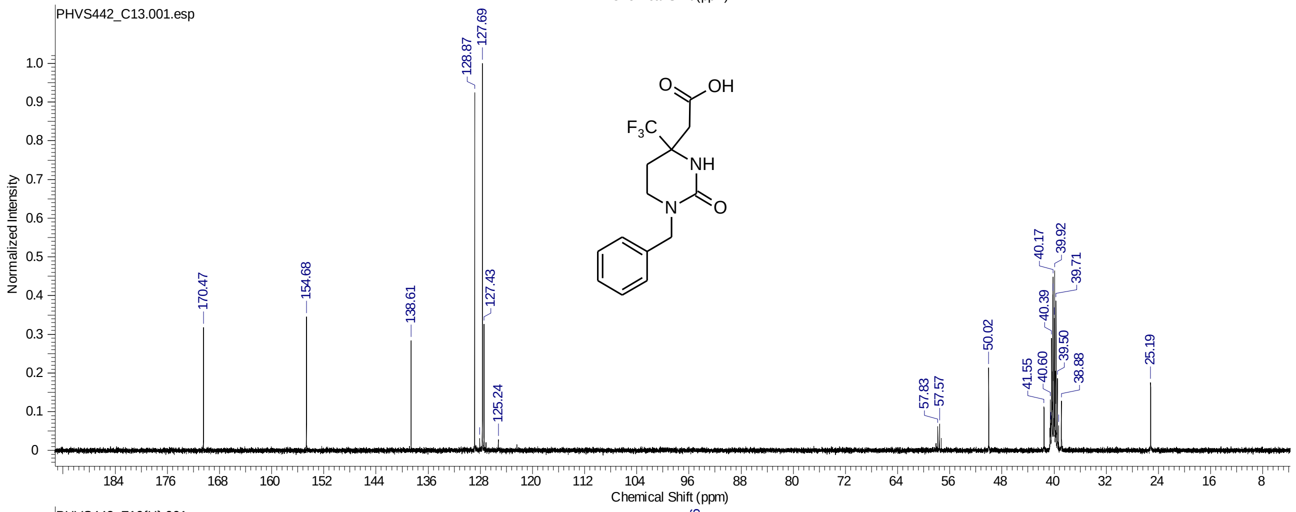
PHVS442_C13.001.esp
Normalized Intensity
Chemical Shift (ppm)
170.47
154.68
138.61
128.87
127.69
127.43
125.24
57.83
57.57
50.02
41.55
40.60
40.39
40.17
39.92
39.71
39.50
38.88
25.19

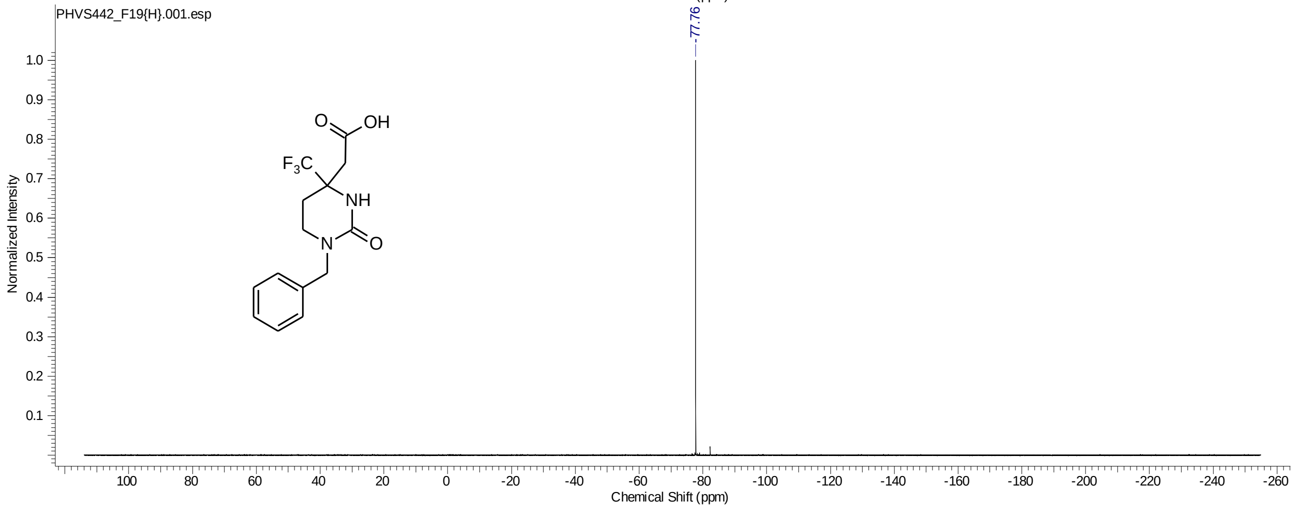
PHVS442_F19{H}.001.esp
Normalized Intensity
Chemical Shift (ppm)
-77.76

Compound **9c**

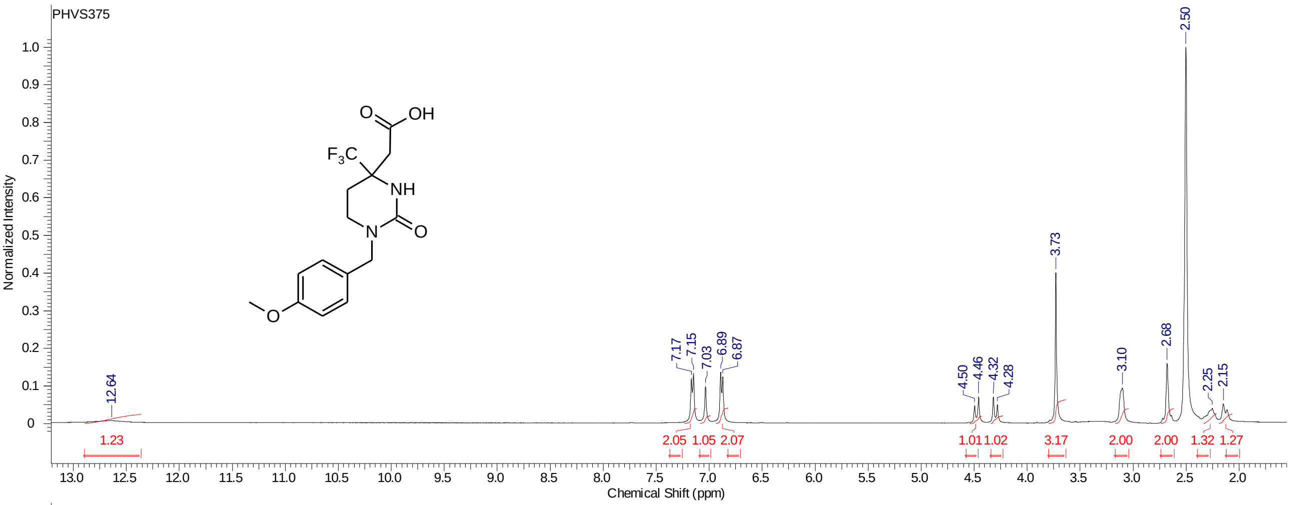

PHVS375
Normalized Intensity
Chemical Shift (ppm)
12.64
7.17
7.15
7.03
6.89
6.87
4.50
4.46
4.32
4.28
3.73
3.10
2.68
2.50
2.25
2.15
1.23
2.05
1.05
2.07
1.01
1.02
3.17
2.00
2.00
1.32
1.27

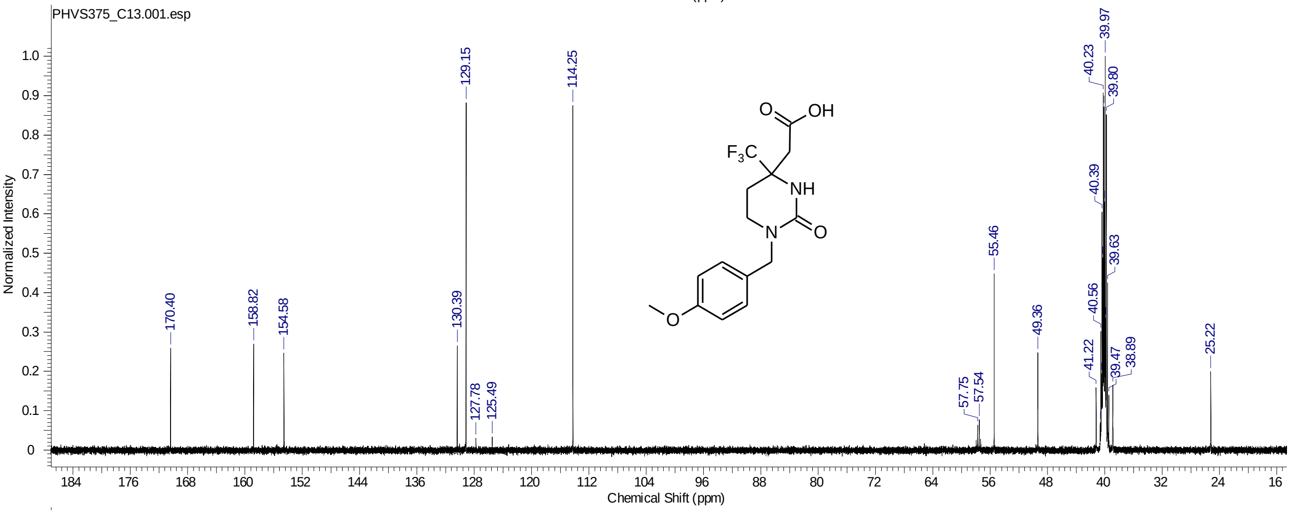

PHVS375_C13.001.esp
Normalized Intensity
Chemical Shift (ppm)
170.40
158.82
154.58
130.39
129.15
127.78
125.49
114.25
57.75
57.54
55.46
49.36
41.22
40.56
40.39
40.23
39.97
39.80
39.63
39.47
38.89
25.22

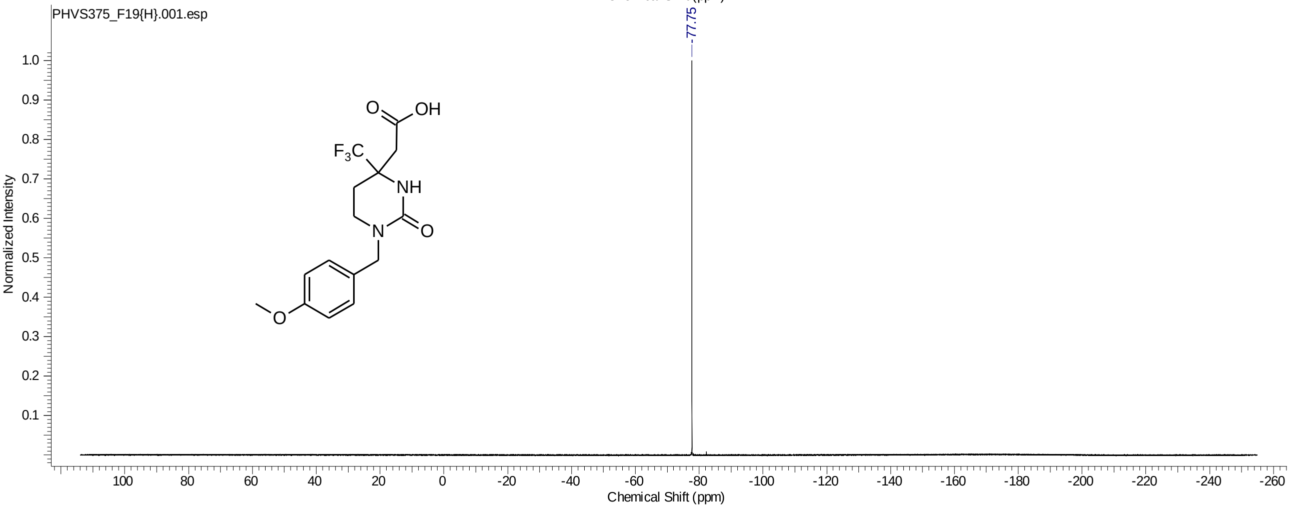

PHVS375_F19{H}.001.esp
Normalized Intensity
Chemical Shift (ppm)
-77.75

Compound **9d**

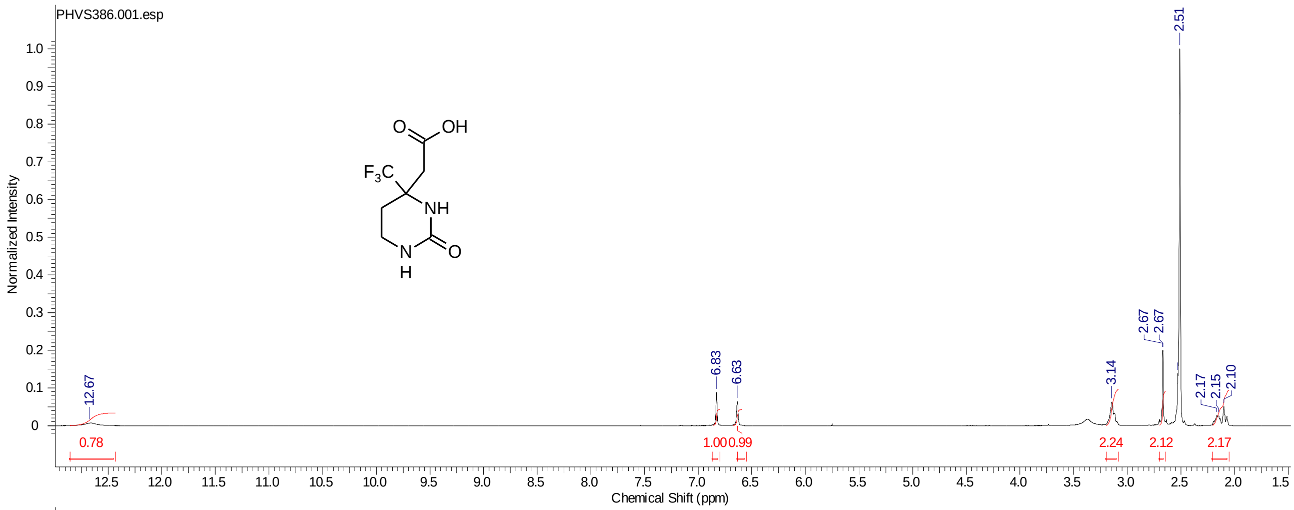
PHVS386.001.esp
Normalized Intensity
Chemical Shift (ppm)
12.67
6.83
6.63
3.14
2.67
2.67
2.51
2.17
2.15
2.10
0.78
1.00
0.99
2.24
2.12
2.17
O
OH
F3C
NH
N
H
O

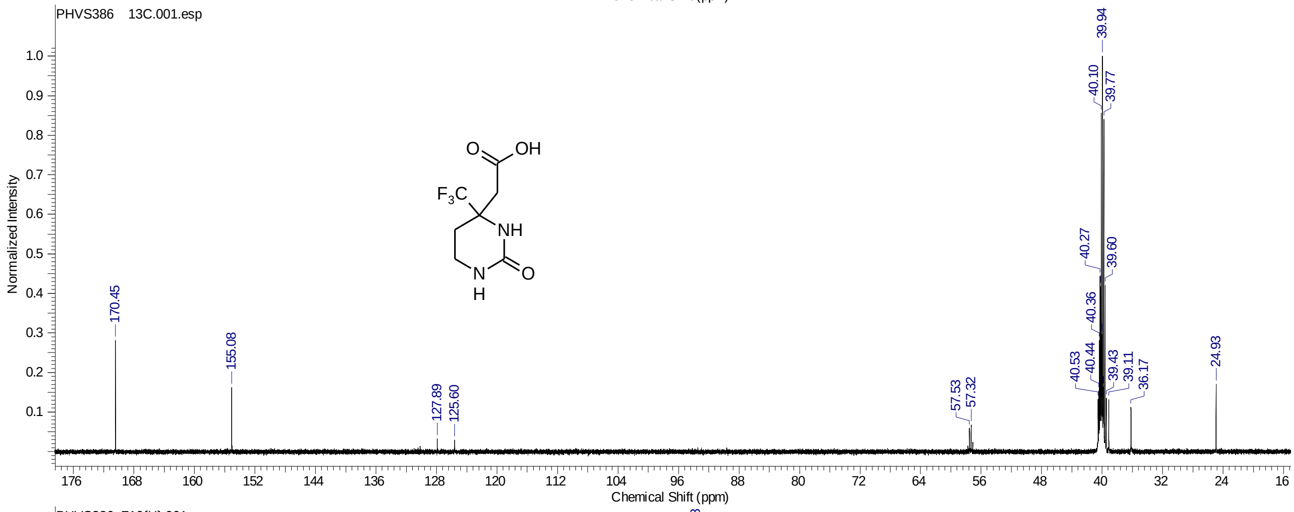
PHVS386 13C.001.esp
Normalized Intensity
Chemical Shift (ppm)
170.45
155.08
127.89
125.60
57.53
57.32
40.53
40.44
40.36
40.27
40.10
39.94
39.77
39.60
39.43
39.11
36.17
24.93
O
OH
F3C
NH
N
H
O

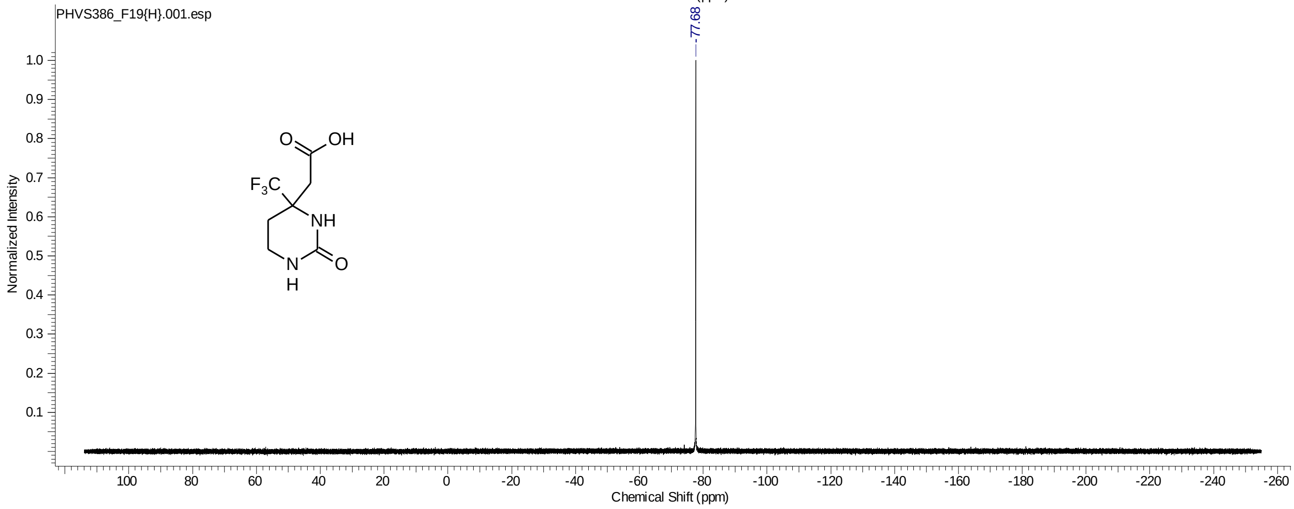
PHVS386_F19{H}.001.esp
Normalized Intensity
Chemical Shift (ppm)
-77.68
O
OH
F3C
NH
N
H
O

Compound **10a**

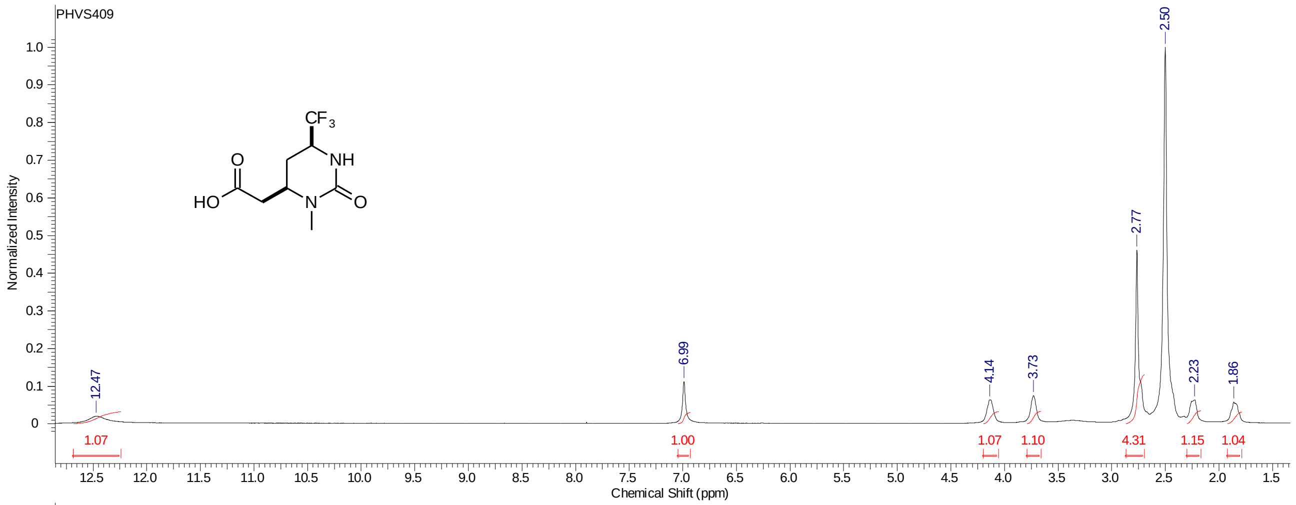

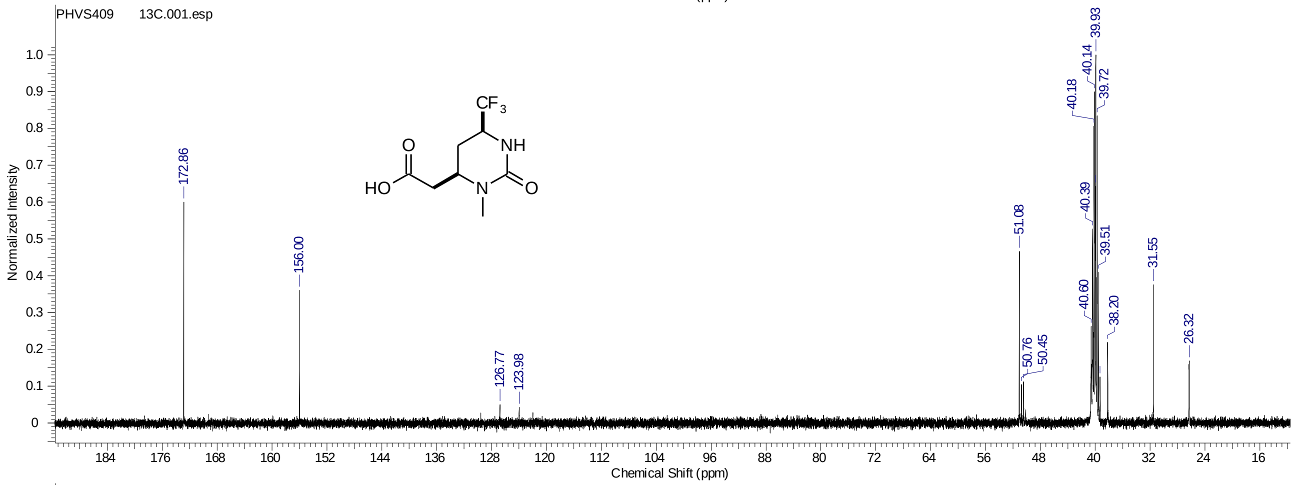

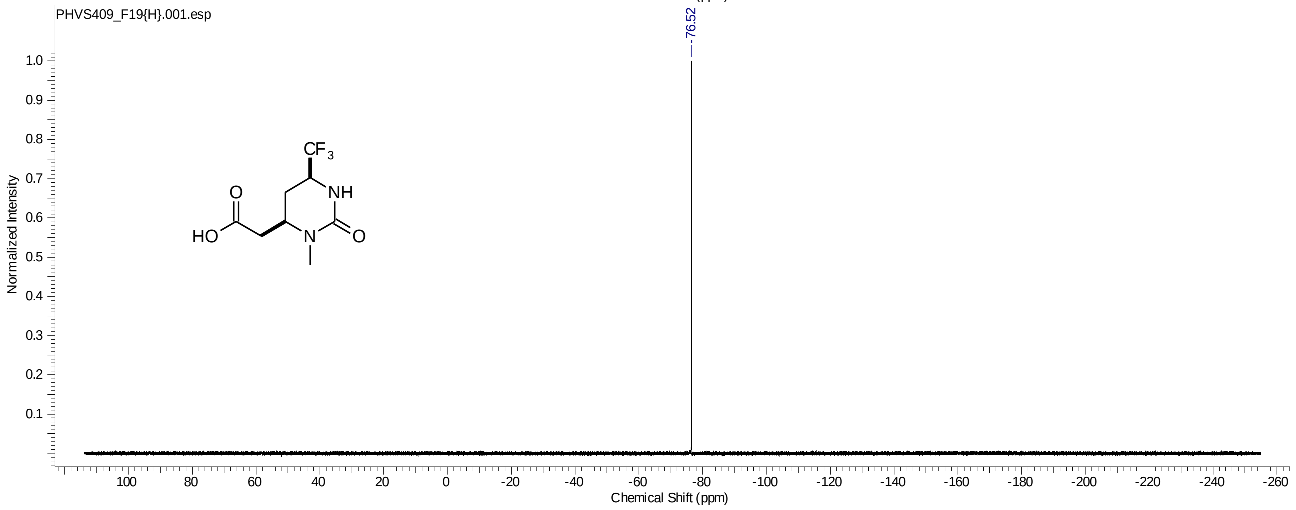

Compound **10b**

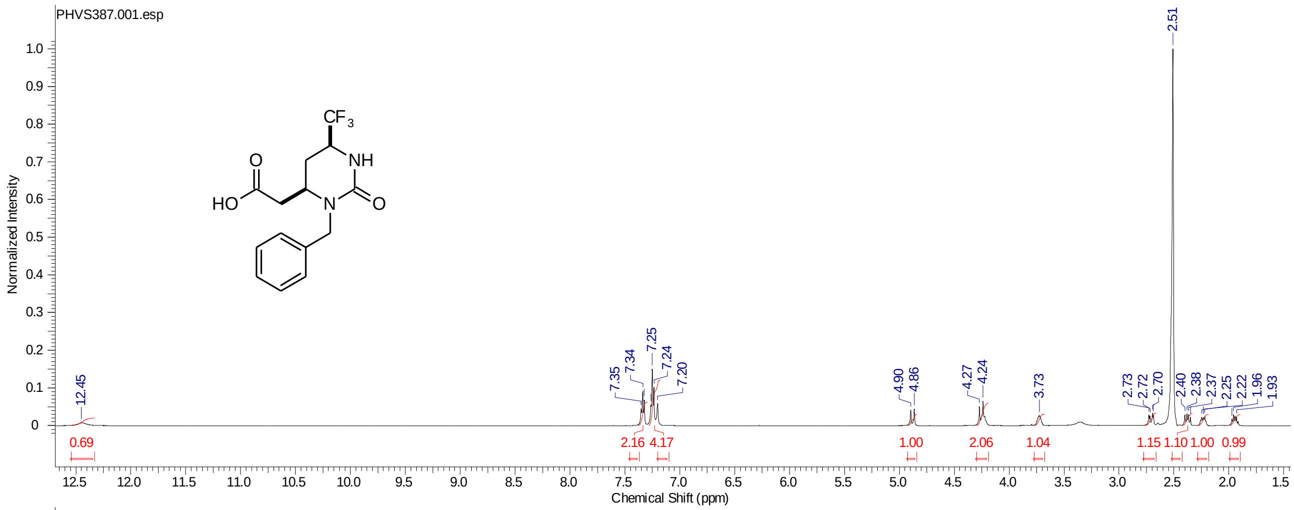

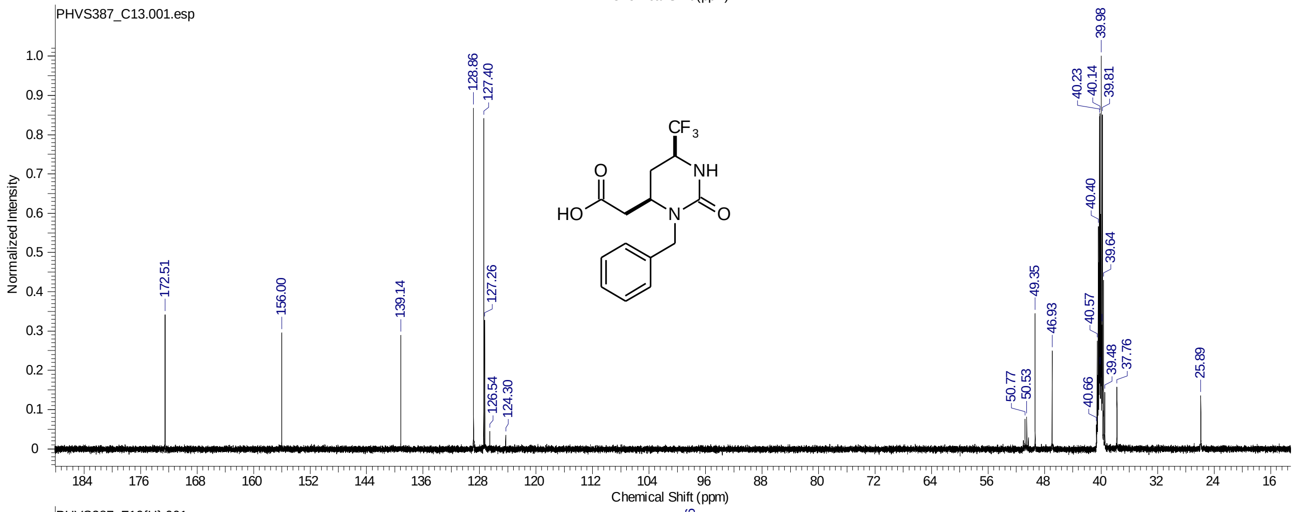

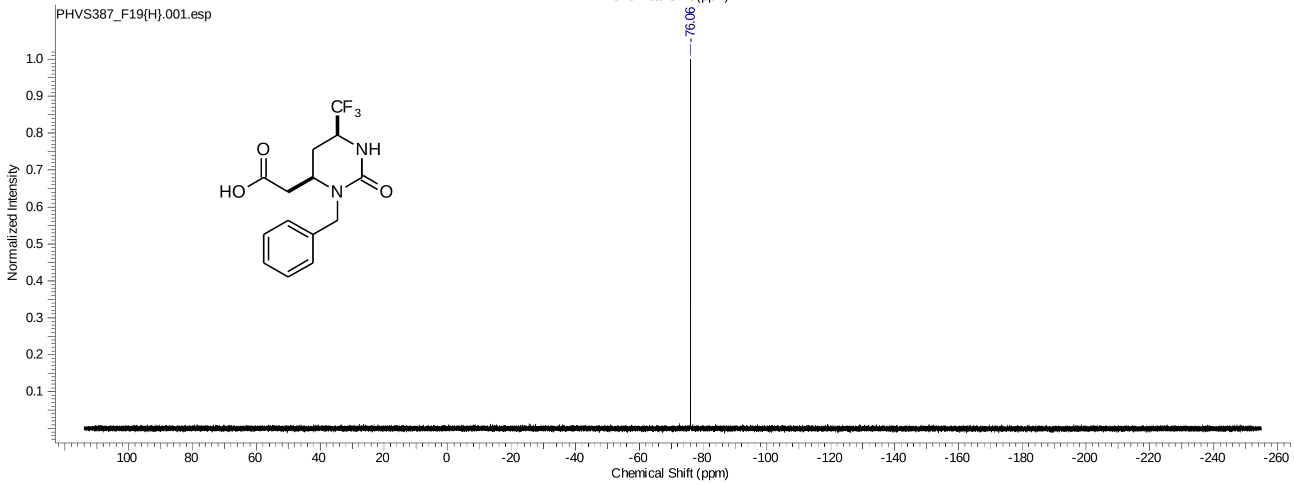

Compound **10c**

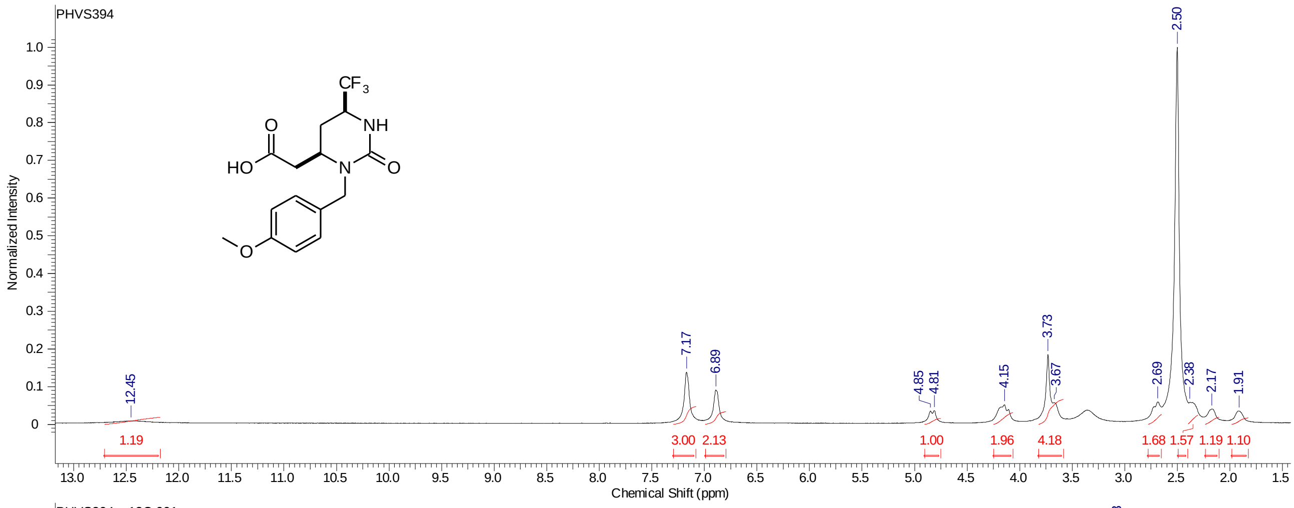

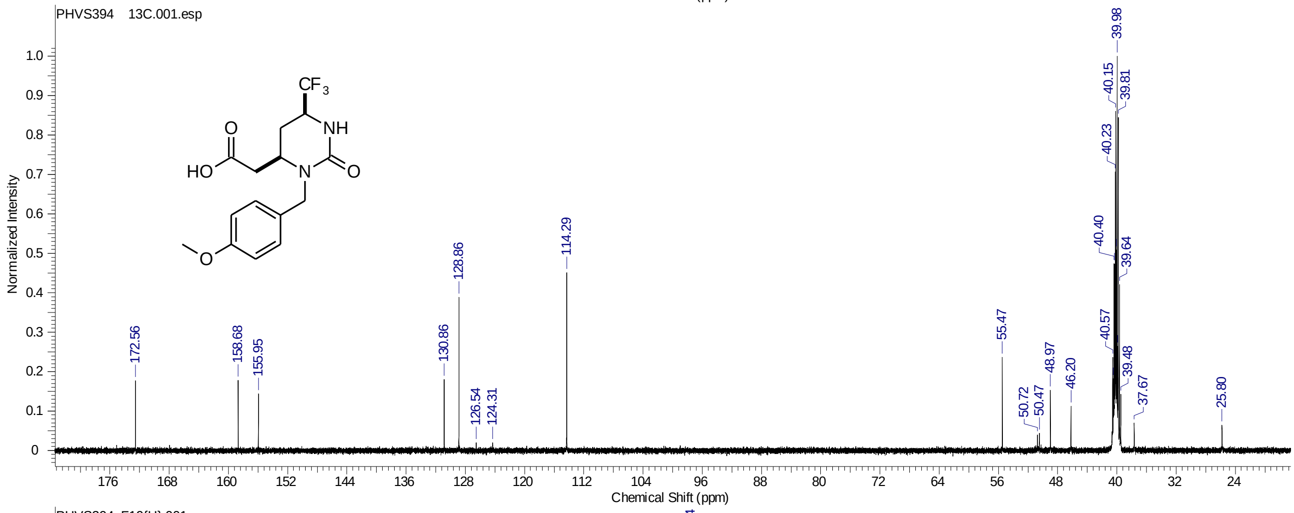

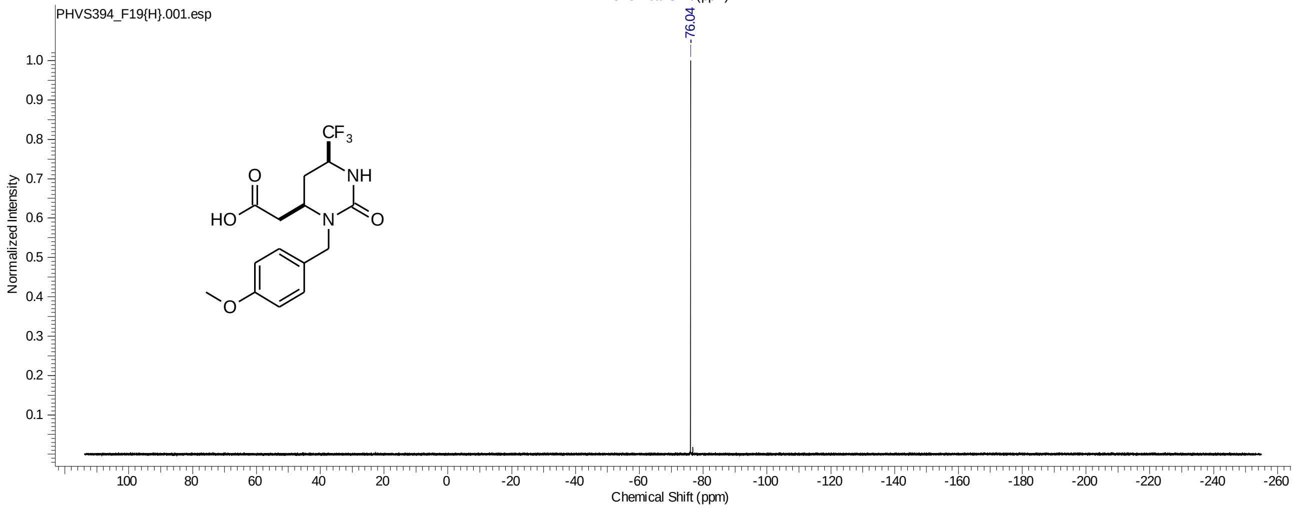

Compound **10d**

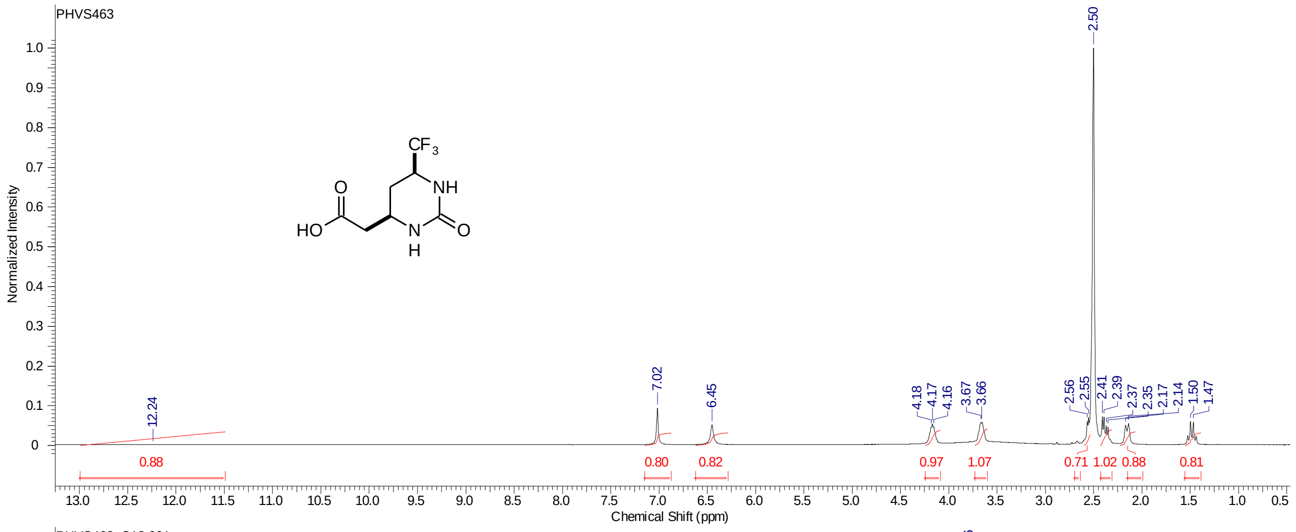

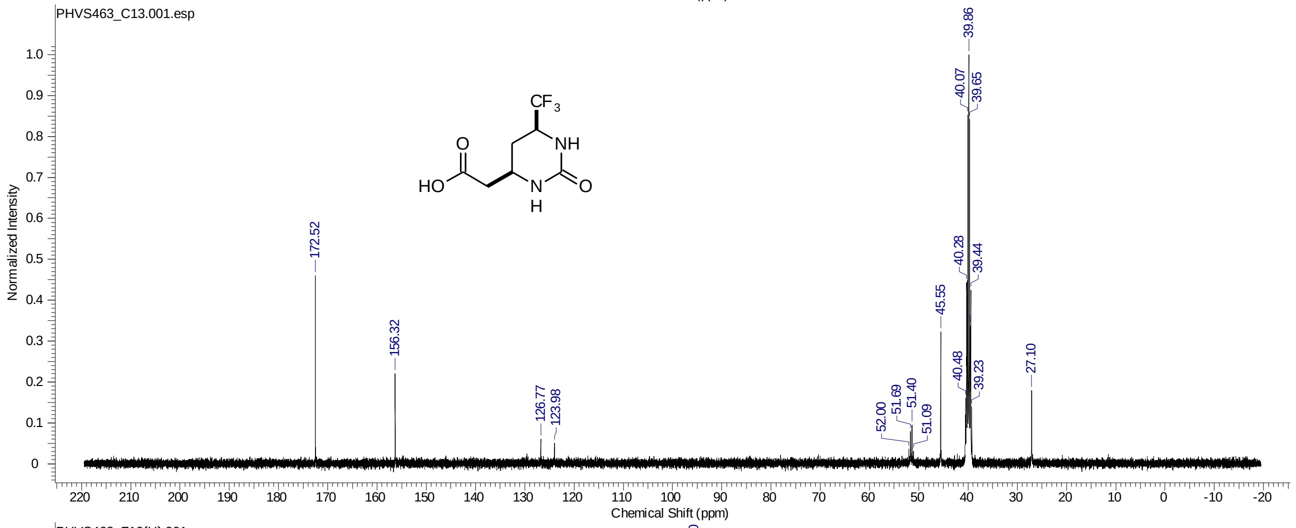

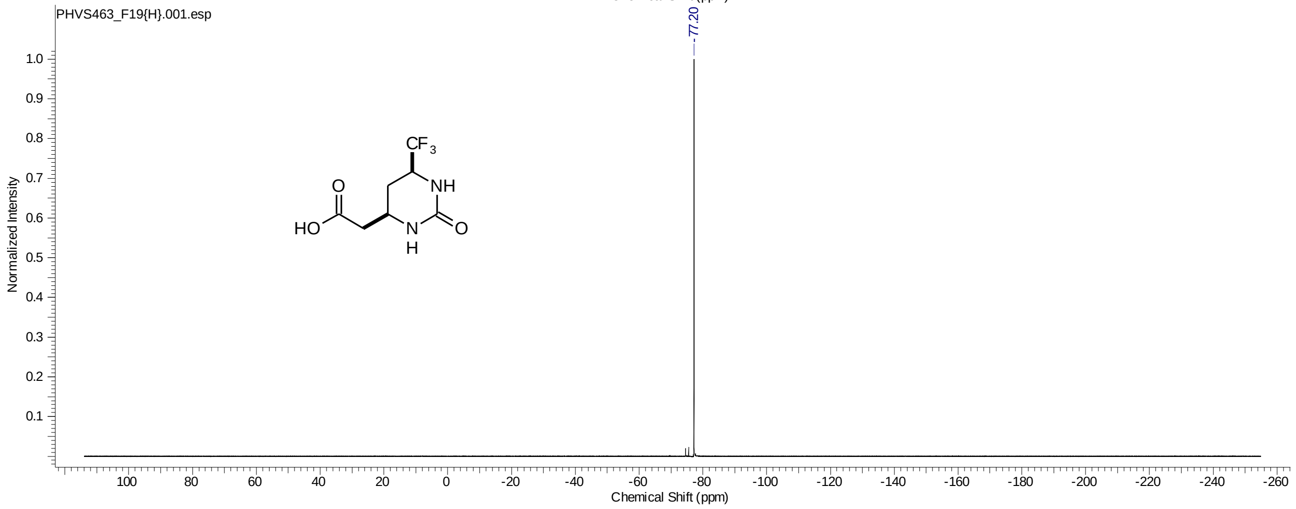

Compound **11a**

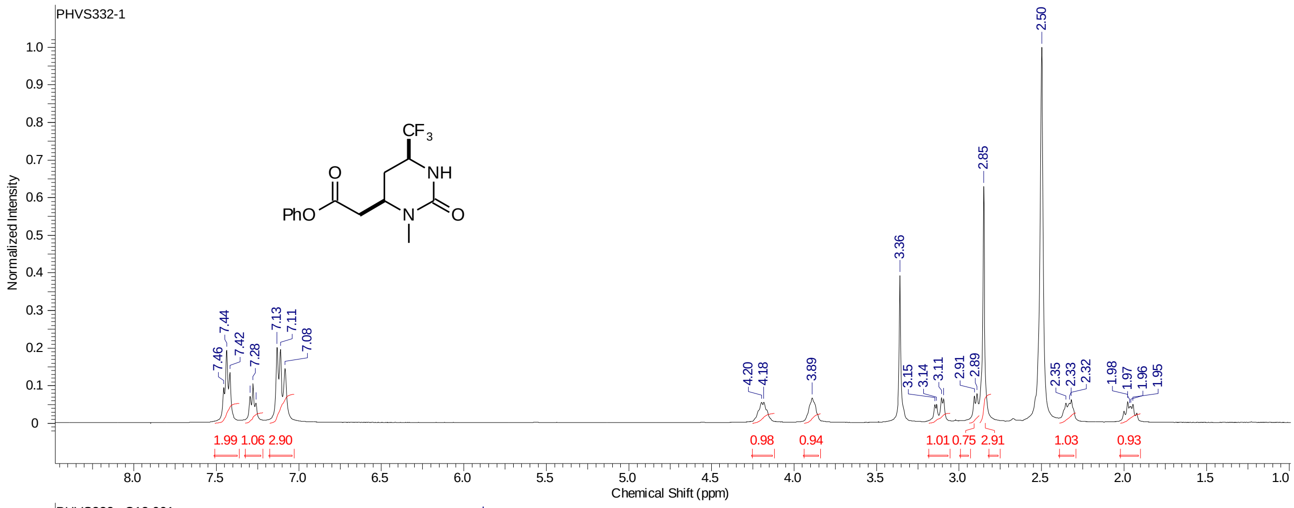

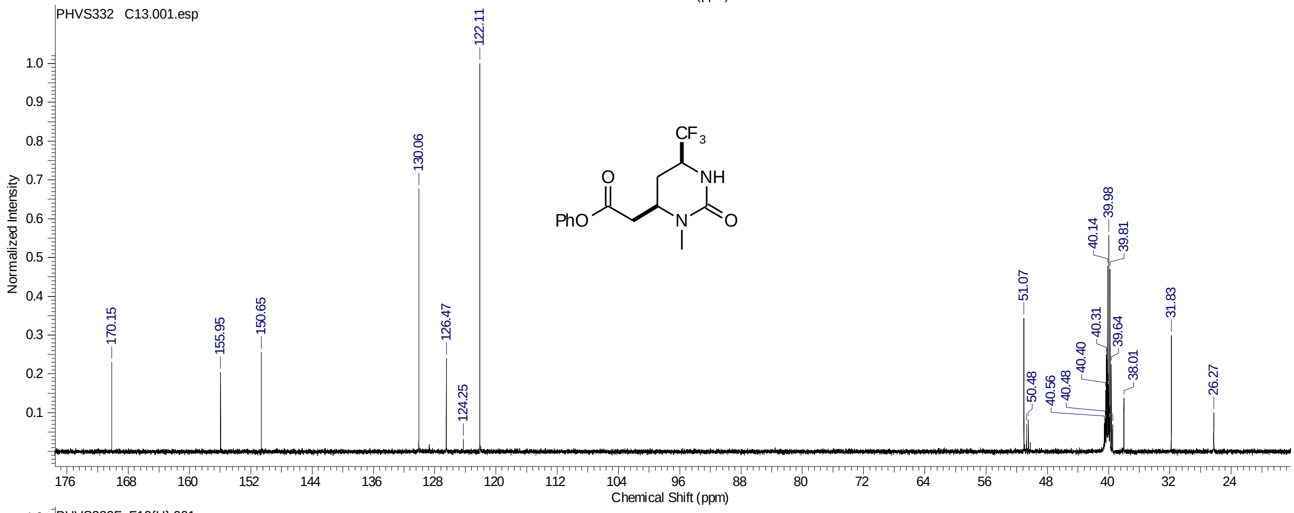

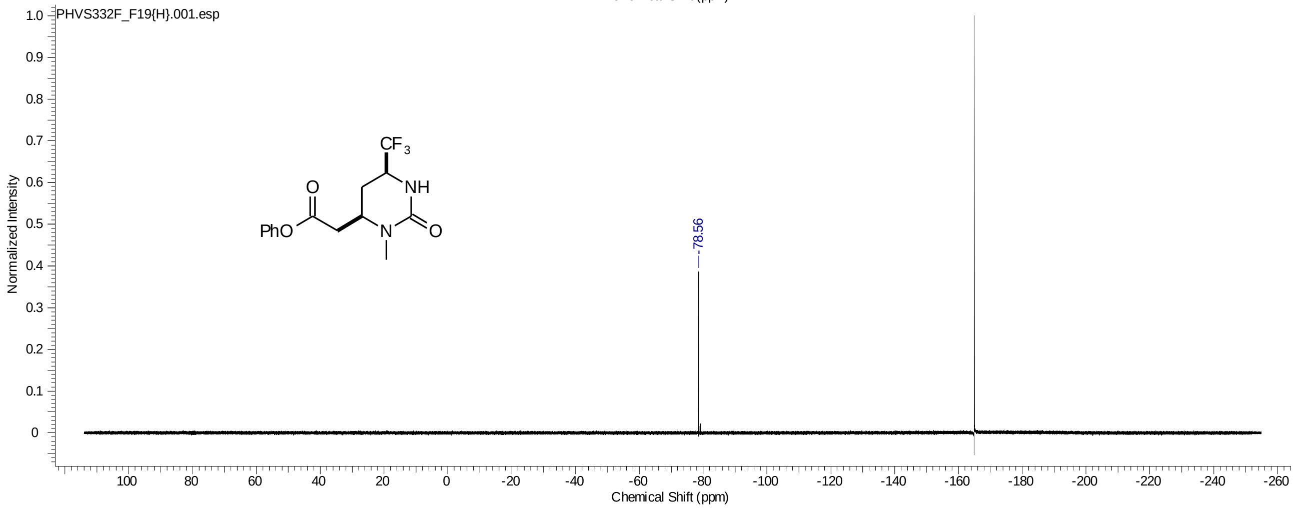

Compound **11b**

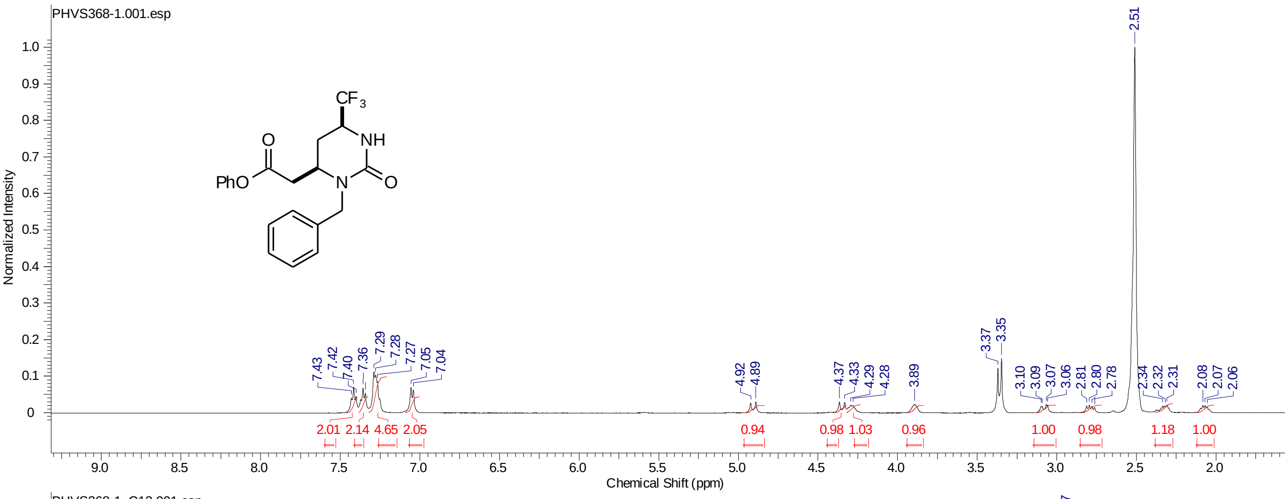

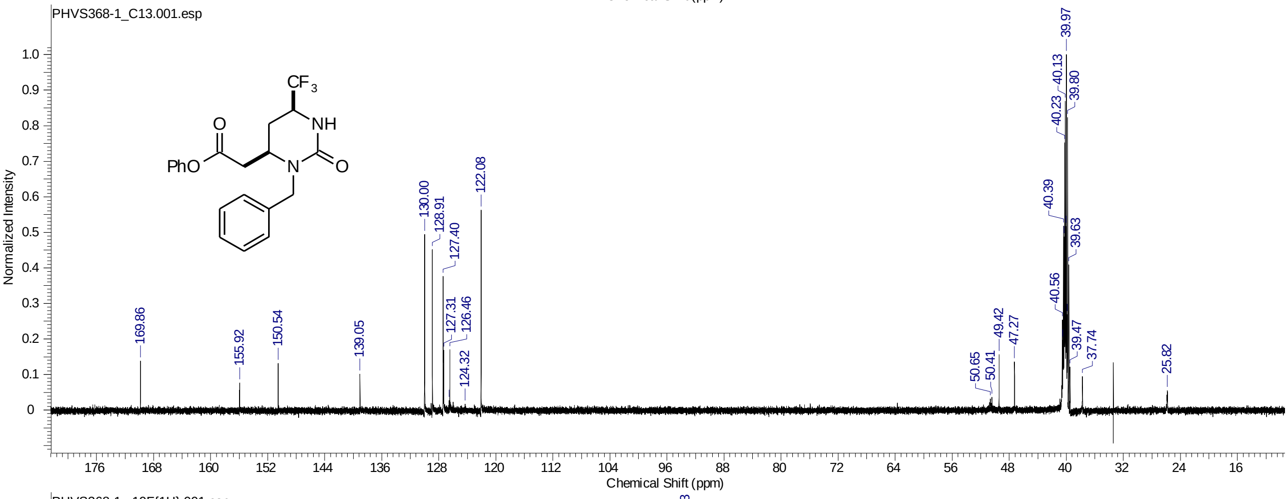

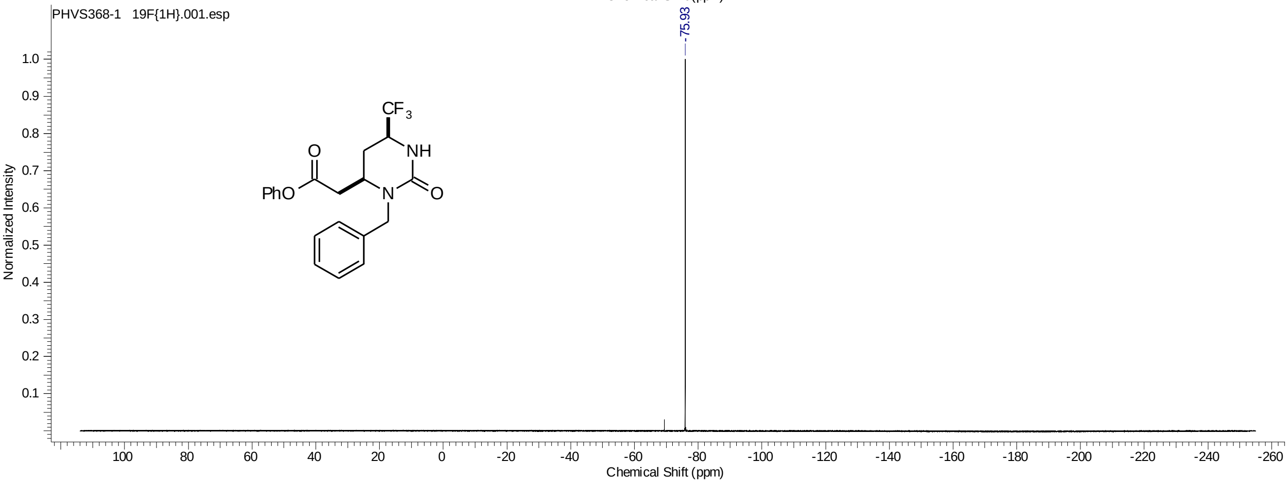

Compound **11c**

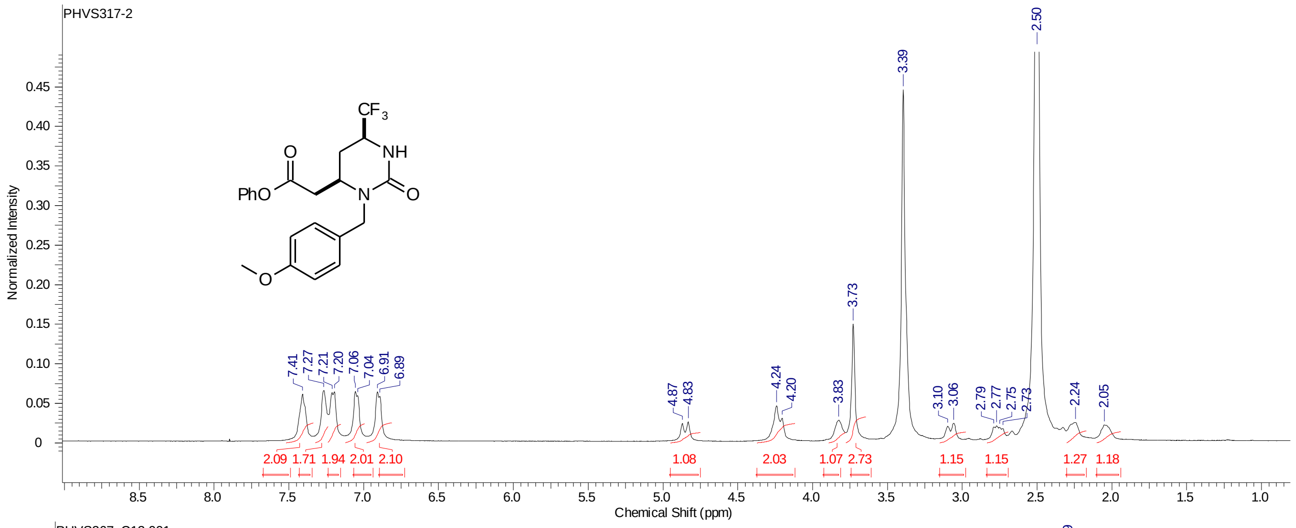

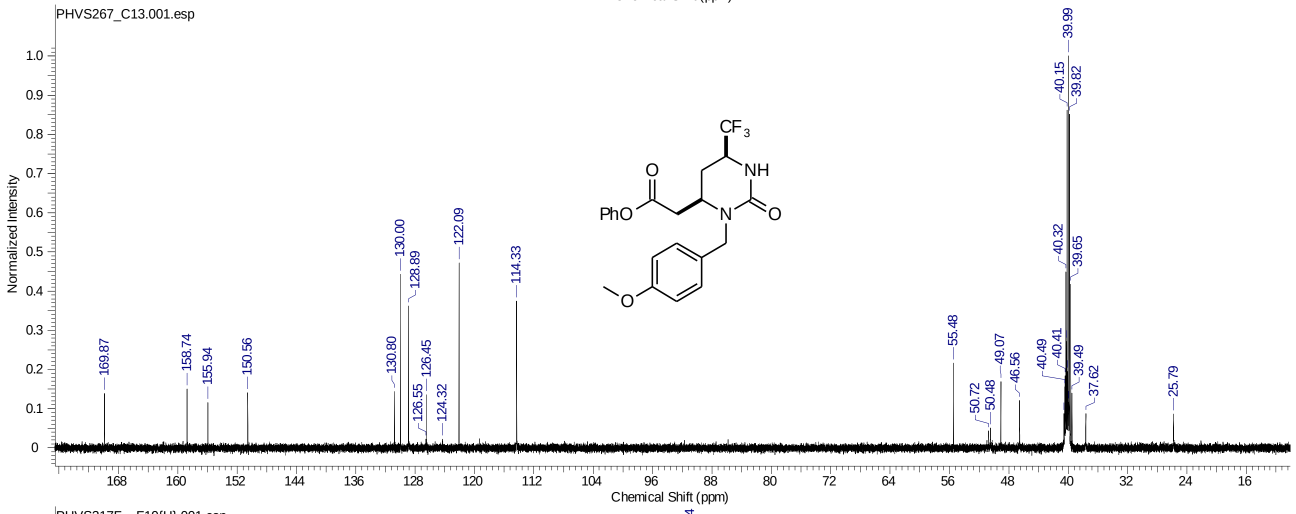

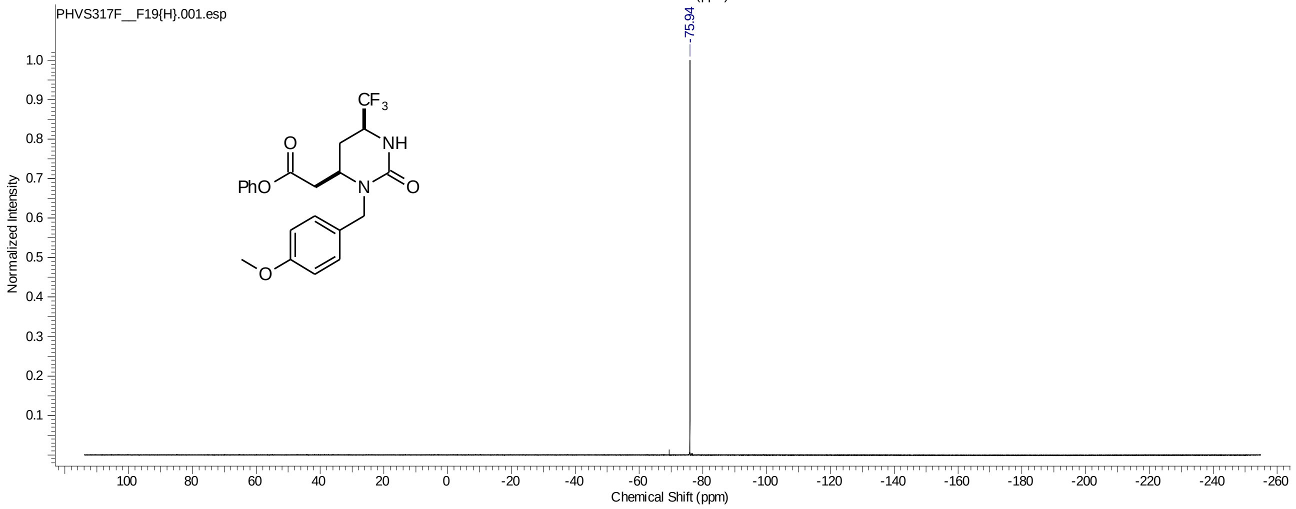

Compound **11d**

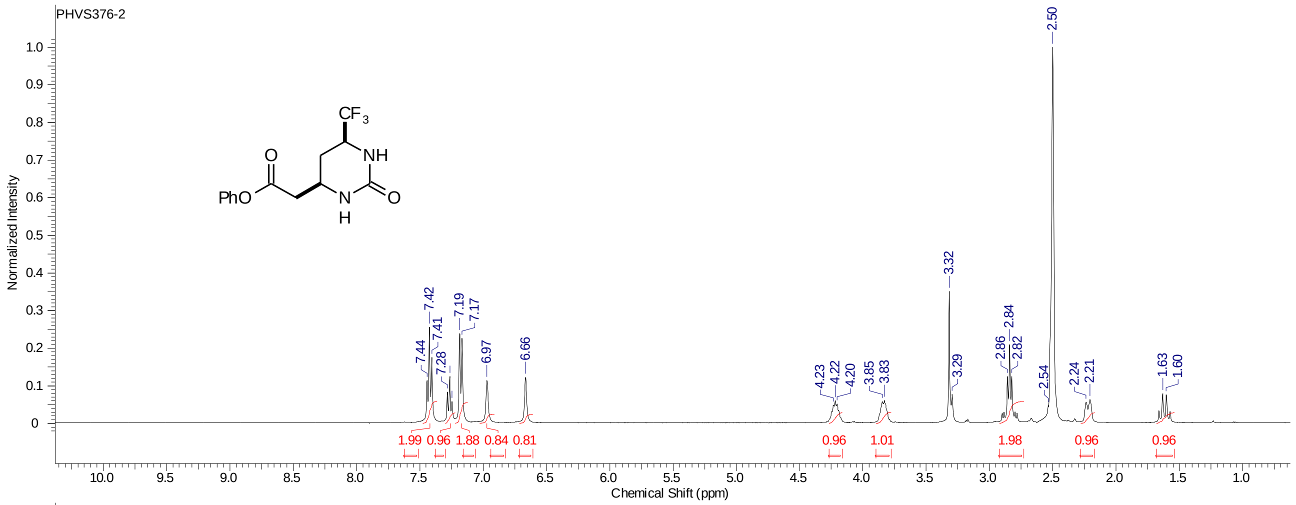

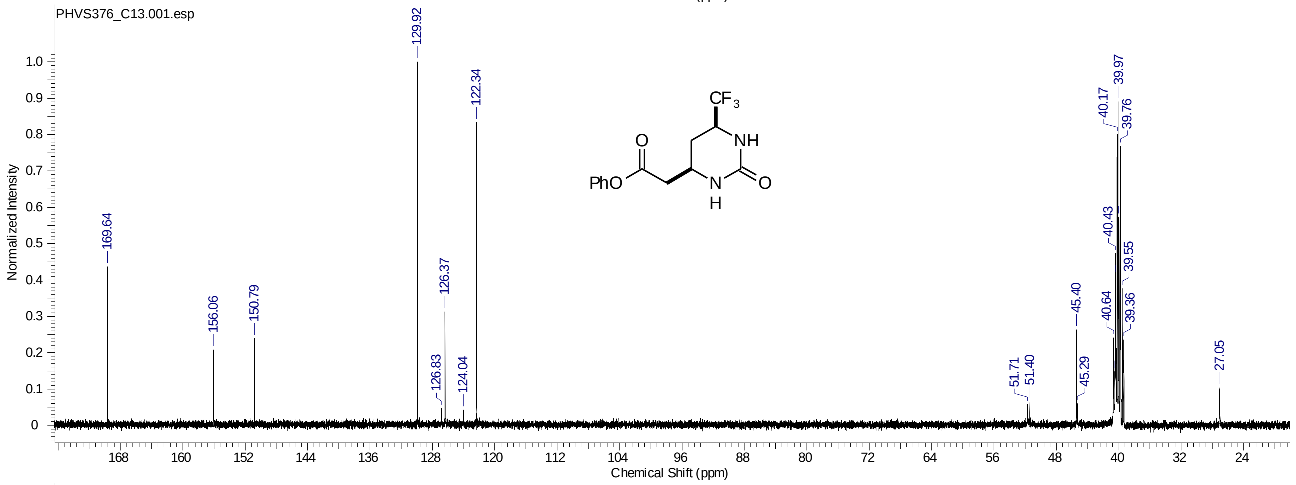

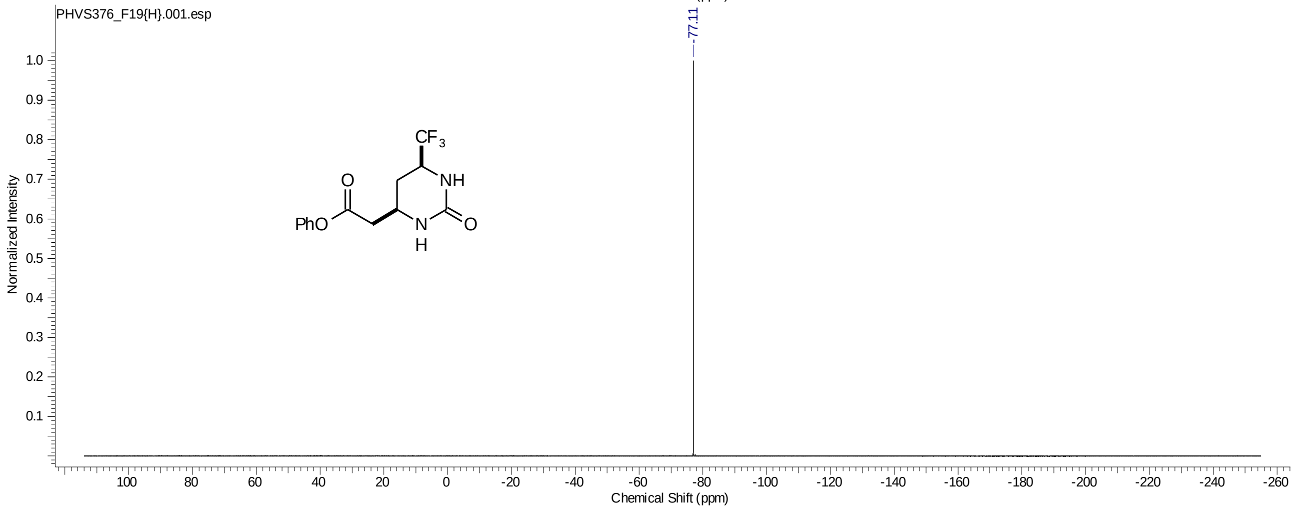

N-Benzyl-2-(3-methyl-2-oxo-6-(trifluoromethyl)-1,2,3,4-tetrahydropyrimidin-4-yl)acetamide

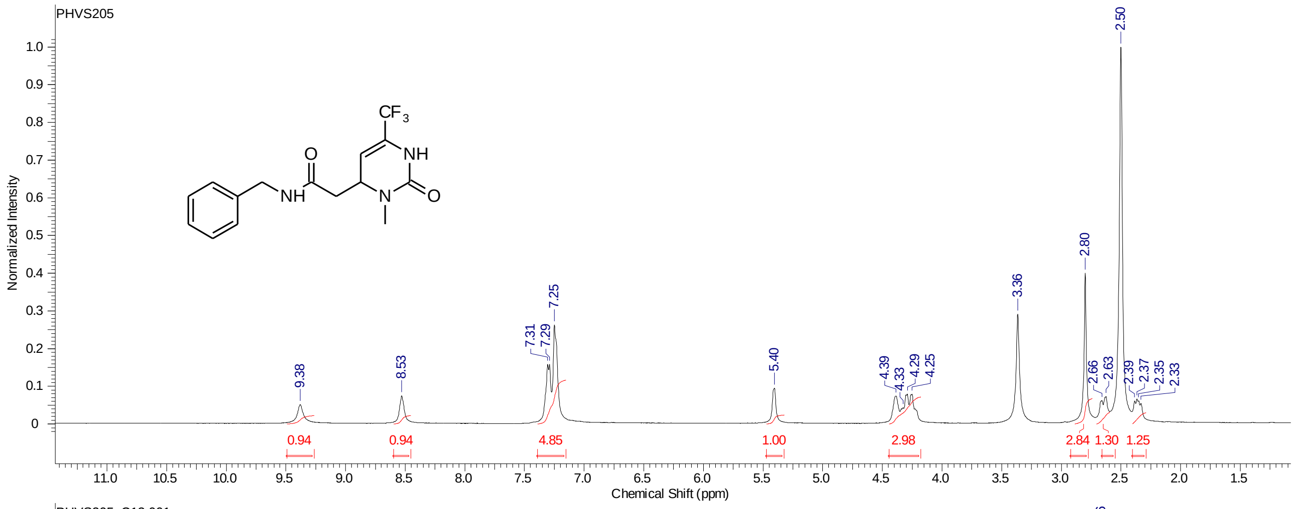

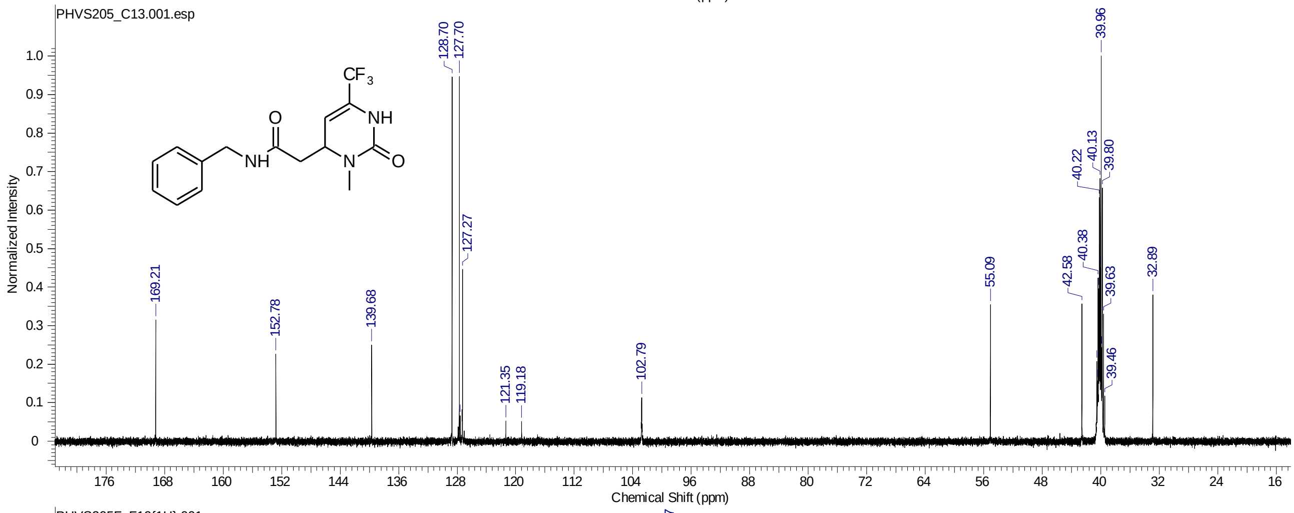

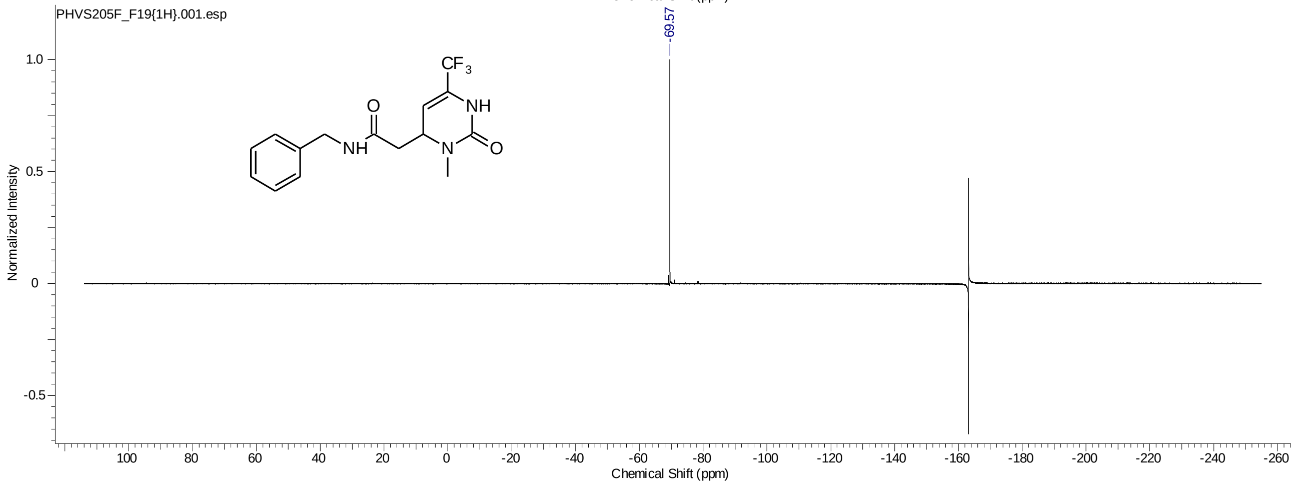

Methyl 4-(2-(benzylamino)-2-oxoethyl)-3-(4-fluorobenzyl)-2-oxo-6-(trifluoromethyl)-1,2,3,4-tetrahydropyrimidine-5-carboxylate

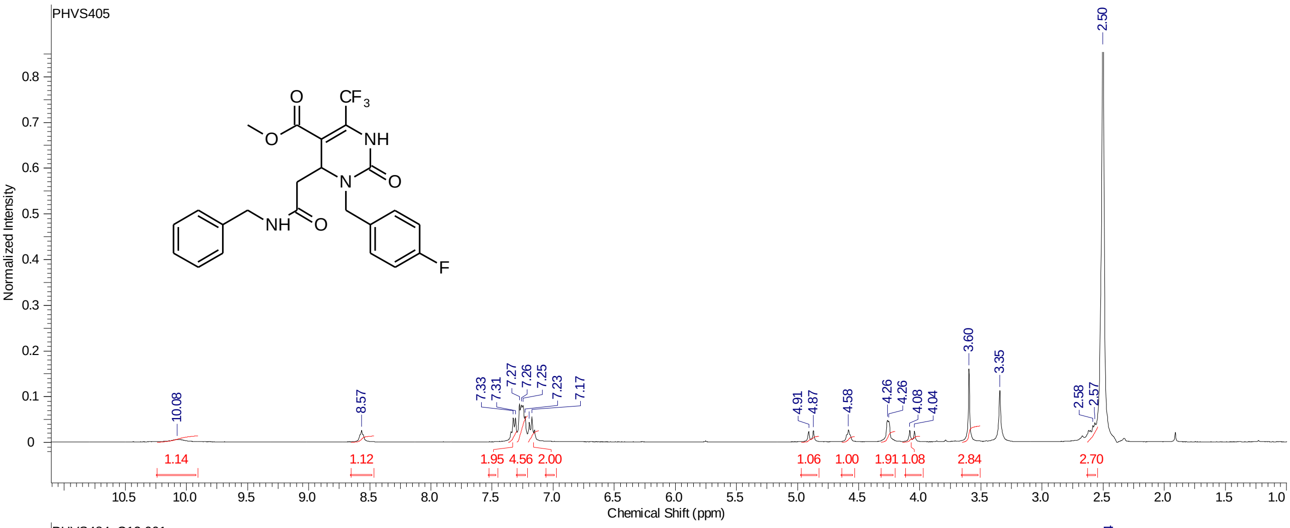

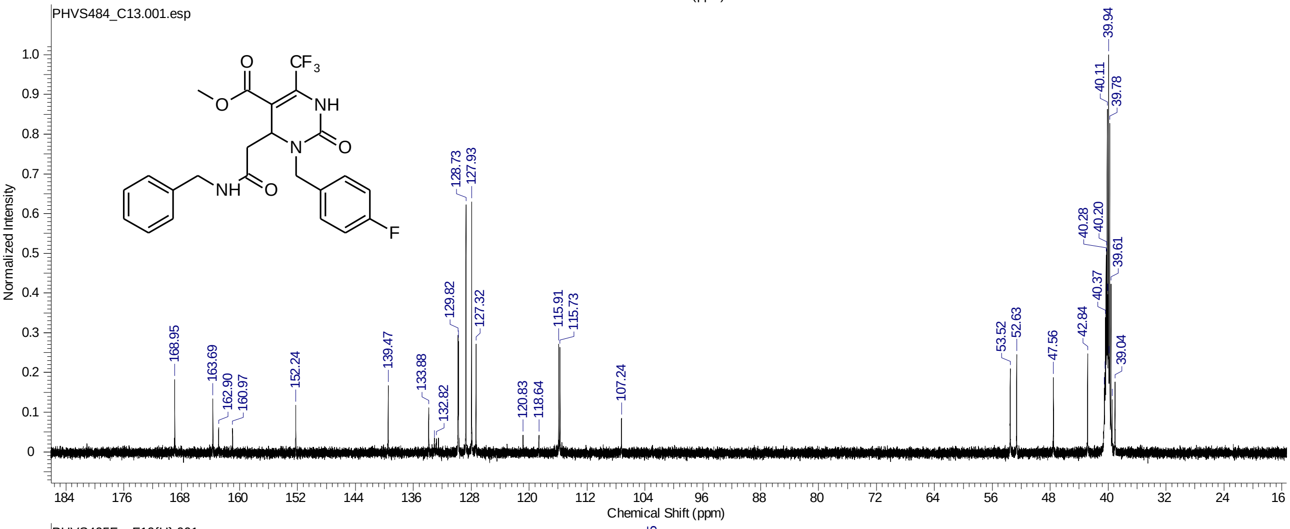

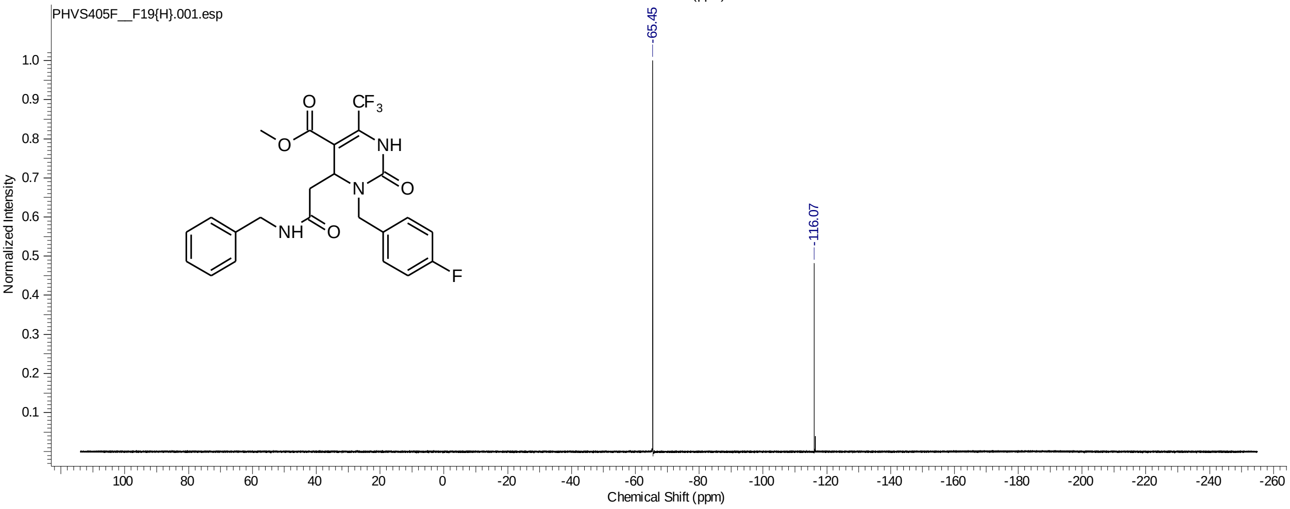

Supplement: File 2 — Copies of the 1H, 13C, and 19F NMR spectra. [file Beilstein_J_Org_Chem-13-2617-s002.pdf]
